# Combining total synthesis and genetic engineering to probe dihydropyran formation in ambruticin biosynthesis†

**DOI:** 10.1039/d4sc00720d

**Published:** 2024-03-12

**Authors:** James I. Bowen, Xiaotong Zhong, Kaining Gao, Benjamin Reed, Matthew P. Crump, Luoyi Wang, Christine L. Willis

**Affiliations:** a School of Chemistry, University of Bristol Bristol BS8 1TS UK chris.Willis@bristol.ac.uk; b Institute of Microbiology, Chinese Academy of Sciences No. 1 Beichen West Road, Chaoyang District Beijing 100101 China wangluoyi@im.ac.cn; c School of Life Sciences, Yunnan University Kunming 650500 China

## Abstract

The ambruticins are a family of potent antifungal polyketide derived natural products isolated from the myxobacterium *Sorangium cellulosum*. Their unusual structures include a trisubstituted cyclopropyl group and two oxygen heterocycles, a tetrahydropyran (THP) and dihydropyran (DHP). Herein we report a flexible modular approach for the total synthesis of ambruticins which is used to prepare ambruticins F and S as well as in the first total synthesis of 20,21-dihydroambruticin F. The flexible strategy unites 3 fragments *via* Julia–Kocienski olefinations and provides important standards for investigation of dihydropyran formation in ambruticin biosynthesis. Cultures of wild-type *S. cellulosum* So ce10 produce mainly ambruticin S and the VS series of metabolites. An efficient electroporation method enabled gene knockout experiments which revealed that the Δ*ambP-S* mutant of *S. cellulosum* accumulated the bisTHP polyketide 20,21-dihydroambruticin F. In contrast, the Δ*ambN-S* mutant gave ambruticin F with the 20,21-alkene as the major metabolite confirming that AmbP and AmbO (a Rieske enzyme and flavin-dependent monooxygenase respectively) are implicated in 20,21-alkene formation. The results of feeding studies to a *Sorangium* strain containing only *ambP* and *ambO* are in accord with formation of the 20,21-alkene occurring prior to generation of the C3 to C7 dihydroxylated tetrahydropyran in ambruticin biosynthesis.

## Abstract 2

Integrating total synthesis with genetic engineering of *Sorangium* strains reveal insights into the enzyme-catalysed formation of the 20,21-alkene in ambruticin biosynthesis.

## Graphical abstract

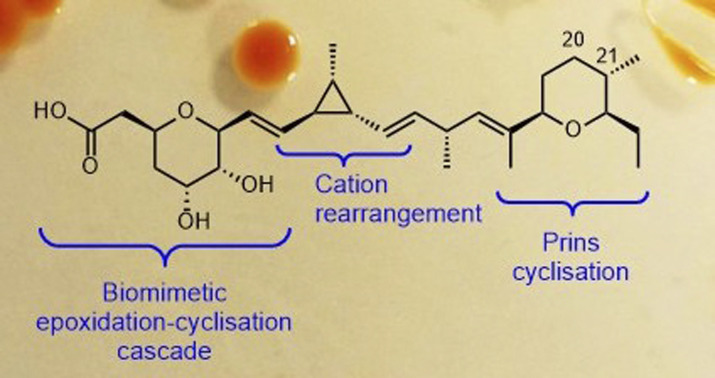

## Introduction

**Scheme 1:**
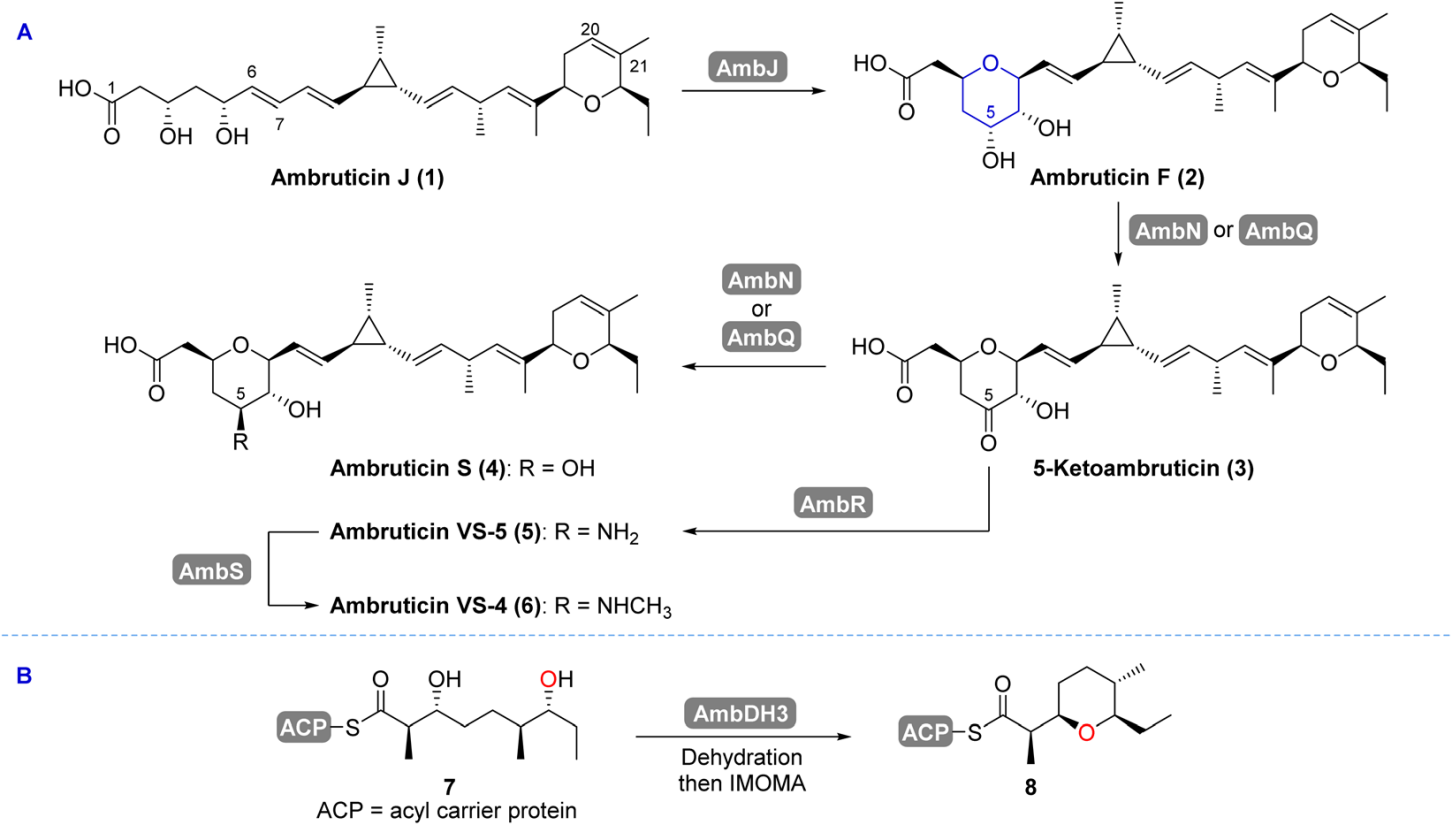
(A) Proposed late stage transformations in ambruticin biosynthesis.^22^ (B) Dehydration and intramolecular oxa-Michael addition (IMOMA) generates an intermediate in the biosynthesis of the DHP.^22,23^

The ambruticins (*e.g.* ambruticins F and S) are a family of polyketide derived natural products, isolated from the myxobacterium *Sorangium (Polyangium) cellulosum* (Scheme 1).^1,2^ They exhibit potent antifungal activity against a range of fungal pathogens including *Coccidioides immitis* and *Blastomyces dermatitidis*.^3,4^ Their antimycotic activity originates from interaction with the high-osmolarity glycerol (HOG) protein kinase signalling pathway.^5^ Importantly, no toxicity was observed in mice dosed with ambruticin S.^6^ Antifungal resistance is an area of growing concern which poses a severe threat to public health and food security.^7–10^ Global warming has exacerbated this by fuelling the worldwide spread of fungi.^11,12^ Research into lead compounds such as the ambruticins has been stimulated by the urgent need to develop antimycotics with novel modes of action to address antifungal resistance.^13^

The ambruticins share several key structural features including a tetrahydropyran (THP) and dihydropyran (DHP) with a hydrocarbon linker incorporating an unusual trisubstituted cyclopropyl ring and 4 olefinic bonds. The structures of the major metabolites from *S. cellulosum* differ at C-5 and include an alcohol (ambruticin S) or amines such as ambruticins VS-5 and VS-4 (Scheme 1A). Ambruticin F (2), the C-5 epimer of ambruticin S (4), has been isolated as a minor metabolite, however, spectral data were not recorded.^14^ Total syntheses of just two members of this family of natural products, ambruticin S and ambruticin J, have been described.^15–21^

In 2006, Reeves reported analysis of the ambruticin biosynthetic gene cluster (BGC) and proposed several unusual features of the modular biosynthetic pathway based on the results from isotopic labelling studies alongside gene knockout experiments.^22^ In the tailoring phase of the polyketide pathway, the THP ring is proposed to be formed by the AmbJ catalysed 6,7-epoxidation of ambruticin J (1), followed by selective intramolecular attack on the oxirane ring to afford ambruticin F (2) (Scheme 1A).^22,24^ AmbQ and AmbN are implicated in oxidation of 2 to 5-ketoambruticin (3), and subsequent reduction to ambruticin S (4). In a parallel pathway reductive amination of ketone 3 (by AmbR) was proposed to give ambruticin VS-5 (5), which undergoes *N*-methylation (by AmbS) to deliver ambruticin VS-4 (6).

The biosynthetic origin of the DHP ring of the ambruticins is unusual. Hahn and co-workers revealed that the oxygen heterocycle is initially formed from linear polyketide 7 by AmbDH3 catalysed dehydration and subsequent intramolecular oxa-Michael addition (IMOMA) giving THP 8 (Scheme 1B).^23^ AmbDH3 has broad substrate specificity as demonstrated in its use in the chemoenzymatic synthesis of the diarylheptanoid centrolobine.^25^ The results of gene knockout experiments led Reeves to propose that dehydrogenation of the THP to a DHP occurs later in the biosynthetic pathway, catalysed by the Rieske oxygenase AmbP and the NAD(P)/FAD-dependent oxidoreductase AmbO.^22^ LC-MS analysis of culture extracts of mutants of *S. cellulosum* where *ambP* or *ambO* were disrupted indicated that a series of 20,21-dihydroambruticins was produced at similar relative levels to each of the corresponding ambruticins (with the 20,21-alkene) found in the wild-type strain.^22^ Hence selective desaturation may occur at various points in the biosynthetic pathway. However, no NMR spectral data for the bisTHP products were reported. Herein we describe the total syntheses of both 20,21-dihydroambruticin F (9) and ambruticin F (2) alongside the results of gene knockout experiments, giving the first definitive experimental evidence for desaturation catalysed by AmbP and AmbO in ambruticin biosynthesis.

## Results and discussion

### Total syntheses of 20,21-dihydroambruticin F, ambruticin F and ambruticin S

**Scheme 2:**
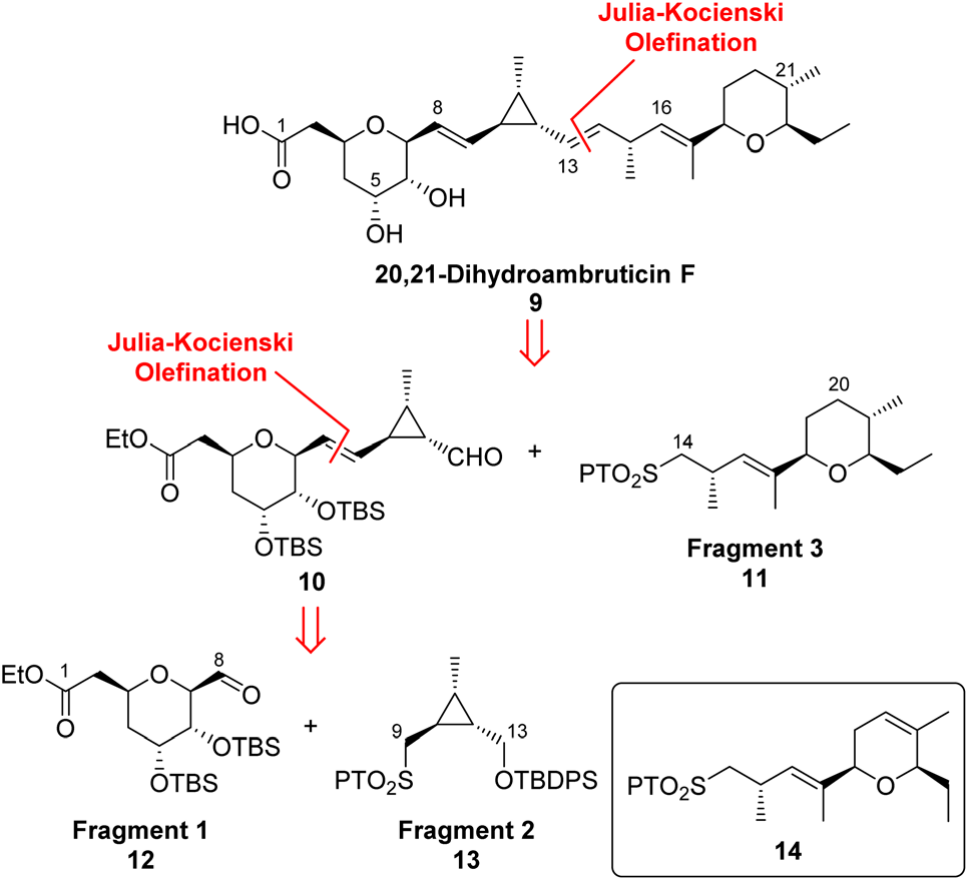
Retrosynthetic analysis of 20,21-dihydroambruticin F (9).

As 20,21-dihydroambruticin F (9) is novel and no literature NMR data are available for ambruticin F (2), we required authentic samples of both to confirm the structures of accumulated products in our proposed gene knockout experiments.^14^ First the total synthesis of 20,21-dihydroambruticin F was investigated based on a flexible modular approach whereby three fragments 11, 12 and 13 were to be united by two Julia–Kocienski olefinations (Scheme 2).^26^ This synthetic strategy would then be readily adapted for the synthesis of ambruticin F by using the DHP containing sulfone 14 rather than the saturated oxygen heterocycle 11 as a coupling partner.

**Scheme 3:**
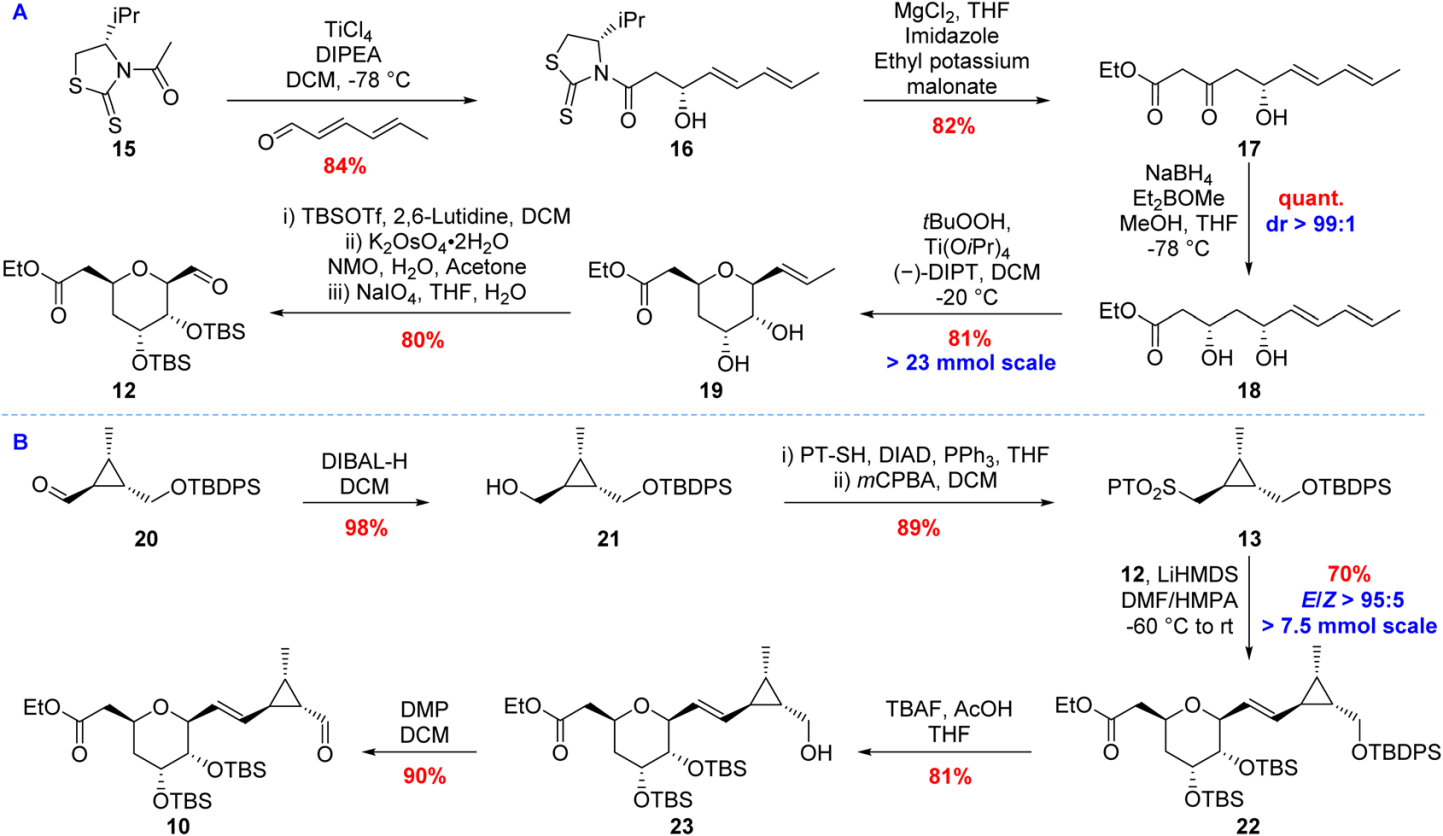
(A) Synthesis of aldehyde 12. (B) Synthesis of aldehyde 10.

The C1–C8 fragment (12) was prepared *via* a bioinspired epoxidation–cyclisation cascade based on the proposed selective THP formation in the AmbJ catalysed conversion of ambruticin J to ambruticin F (Scheme 1A).^20^ Asymmetric aldol condensation between *E*,*E*-hexadienal and acetylthiazolidinethione 15, followed by a decarboxylative Claisen condensation gave β-hydroxy ketone 17 (Scheme 3A).^27^ Stereoselective Narasaka–Prasad reduction of 17 yielded *syn*-diol 18 in 69% yield over the three steps. The *syn*-diol relationship was confirmed by Rychnovsky analysis of the corresponding acetonide (see ESI†).^28–30^ Conversion of 3,5-dihydroxy ester 18 to THP 19 was achieved *via* Sharpless asymmetric epoxidation, with the intermediate epoxide undergoing selective spontaneous cyclisation, providing THP 19 in 81% yield.^24,31^ These reactions could be readily conducted on >23 mmol scale. Protection of the *syn*-diol as silyl ethers followed by oxidative cleavage of the alkene afforded the desired aldehyde fragment 12 in 45% yield over 6 steps from 15.

For the C9–C13 fragment (13), reduction of the known aldehyde 20 (ref. 20) with DIBAL-H followed by a Mitsunobu reaction and oxidation with *m*CPBA provided the phenyl tetrazole sulfone 13 (Scheme 3B).^17^ Oxidation of the intermediate sulfide was also achieved using molybdate and H_2_O_2_ in ethanol giving similar yields of sulfone 13, but the reaction proved less amenable to scale up.^32^ Julia–Kocienski reaction of aldehyde 12 with sulfone 13 afforded the desired *E*-olefin 22 in 70% yield. LiHMDS in a mixture of DMF and HMPA was required to achieve the required *trans*-selectivity in the reaction, which was conducted successfully on >7.5 mmol scale.^17^ Selective deprotection of the primary alcohol with TBAF and acetic acid in THF followed by oxidation with Dess–Martin periodinane (DMP) furnished aldehyde 10 in 73% yield over the two steps.^33^

**Scheme 4:**
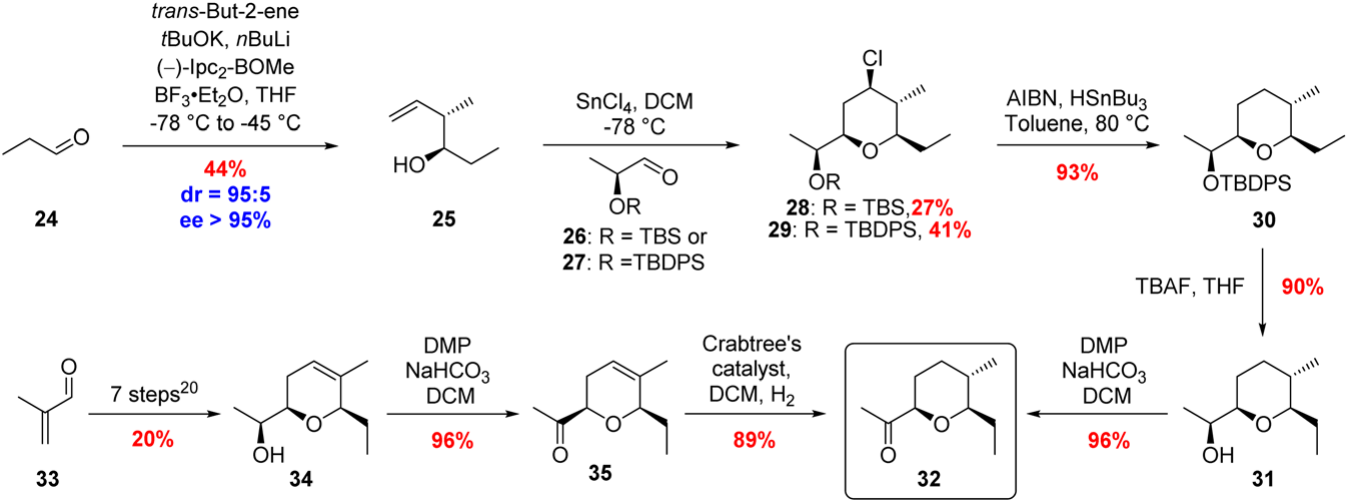
Two approaches to the synthesis of ketone 32.

The final building block required for the total synthesis of 20,21-dihydroambruticin F (9) was tetrahydropyran 11. Prins cyclisations have been widely used in natural product synthesis and we investigated the use of a SnCl_4_ mediated Prins cyclisation for the stereoselective construction of the THP ring.^34,35^ Reaction of homoallylic alcohol 25 with 2-*tert*-butyldimethylsilyloxypropanal 26, gave THP 28 with all four substituents in the required equatorial position but in a disappointing 27% yield due to *in situ* deprotection of the silyl ether (Scheme 4). Using the more robust TBDPS protecting group gave 29 in 41% yield. Radical dechlorination of 29 followed by deprotection of the silyl ether and subsequent oxidation of the resultant alcohol gave ketone 32 in 80% yield over the 3 steps from 29.^35^

An alternative approach to ketone 32 began with the known DHP 34 prepared in 7 steps and 20% overall yield from 33.^20,36^ Following oxidation of 34, hydrogenation of 35 with Crabtree's catalyst afforded the desired THP fragment 32 in 89% yield as a single diastereomer (Scheme 4).^37^ The observed selectivity was rationalised by hydrogenation being directed to the top face of the alkene by the ketone.

**Scheme 5:**
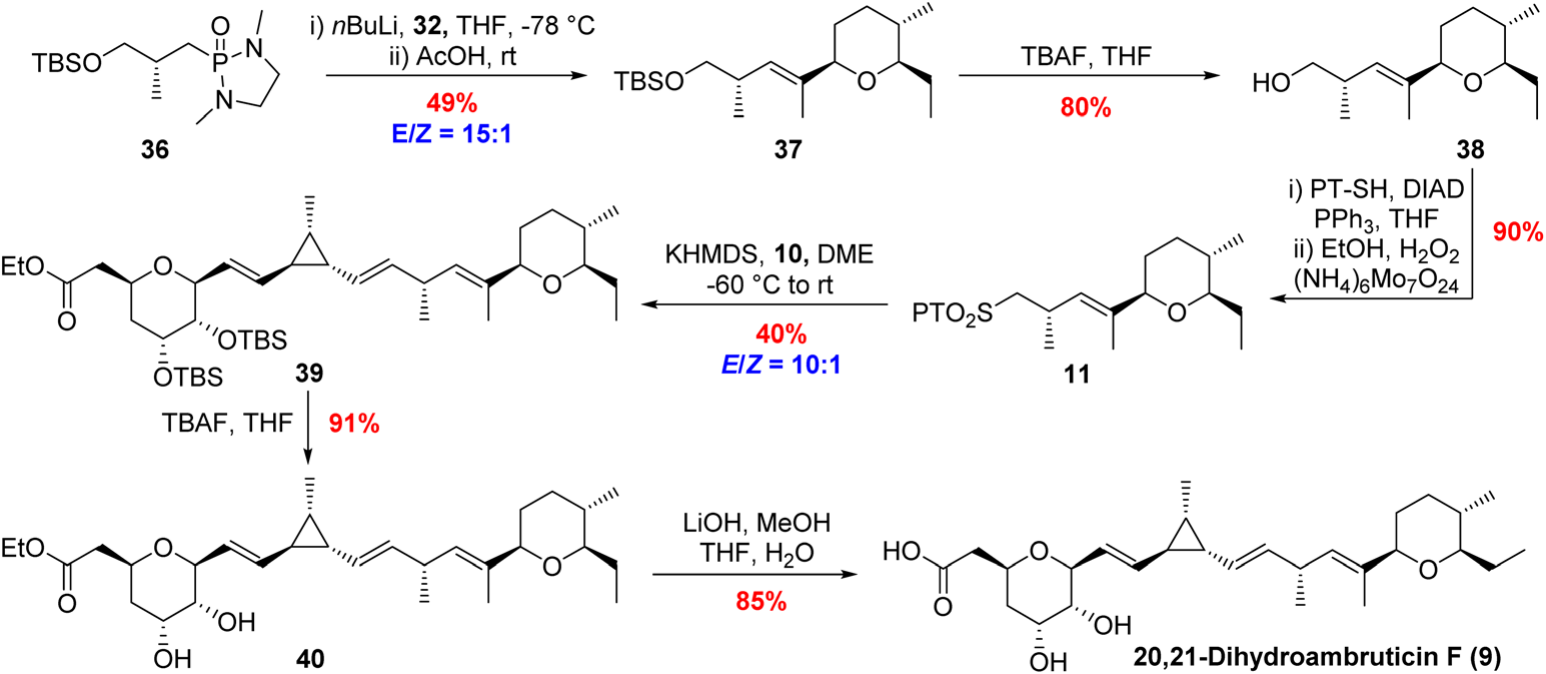
Synthesis of sulfone 11 and completing the total synthesis of 20,21-dihydroambruticin F (9).

Ketone 32 was coupled with known phosphorus diamide 36 under conditions developed by Hanessian in the total synthesis of ambruticin S (Scheme 5).^19^ The olefination provided a 15 : 1 mixture of alkene isomers which, after silyl deprotection, were separated to afford the desired *E*-alkene 38 in 39% yield over the two steps. Finally, conversion of alcohol 38 to the corresponding phenyl tetrazole sulfone 11 (fragment 3) proceeded smoothly through a sequence of Mitsunobu coupling and subsequent oxidation of the intermediate sulfide. The total synthesis of 20,21-dihydroambruticin F (9) was completed as shown in Scheme 5. Deprotonation of sulfone 11 with 1.2 equivalents of KHMDS in DME, followed by treatment with 1.2 equivalents of aldehyde 10 gave an inseparable mixture of alkene isomers (*E*/*Z* = 10 : 1) in 40% yield. The use of NaHMDS in a mixture of THF and HMPA resulted in an improved yield of 78% for the required coupling but reduced the stereoselectivity (*E*/*Z* = 3 : 1). A screen of further solvents and bases failed to improve either the yield or selectivity. Silyl deprotection of 39 with TBAF followed by hydrolysis of the ethyl ester gave the target, 20,21-dihydroambruticin F (9).

**Scheme 6:**
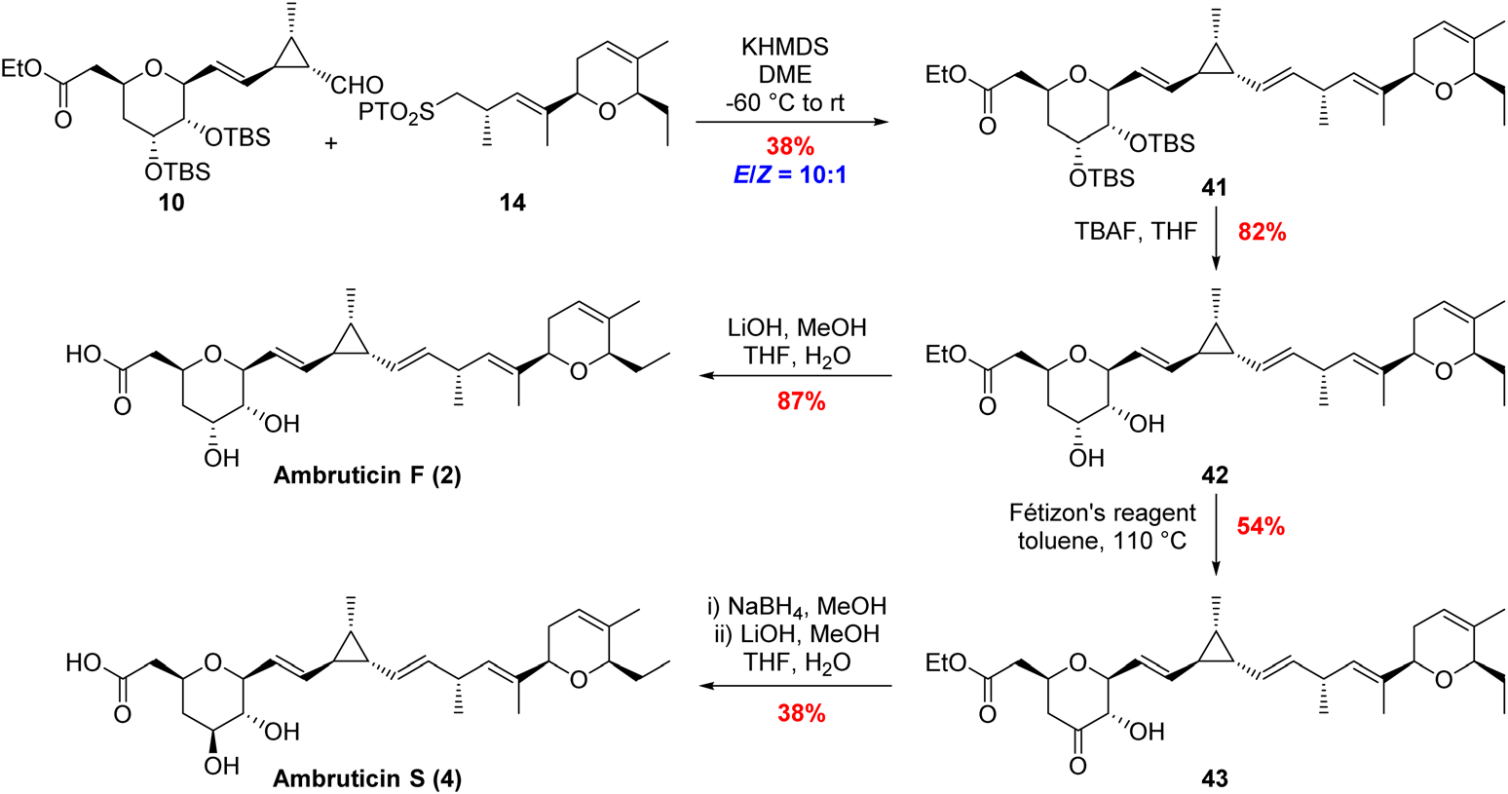
Completing the total syntheses of ambruticins F and S.

The same modular strategy was used for the synthesis of ambruticin F (2). In this case the known sulfone 14 (ref. 20) was coupled to aldehyde 10 to afford alkene 41 in 38% yield as a 10 : 1 mixture of inseparable *E*/*Z* isomers (Scheme 6). Again, it was found that use of NaHMDS in a mixture of THF and HMPA in the coupling reaction gave an improved yield (80%) but the selectivity was 3 : 1 in favour of the *E*-alkene. Deprotection of bissilyl ether 41 with TBAF in THF followed by ester hydrolysis completed the first total synthesis of ambruticin F (2). As NMR data of the natural product 2 were not available for comparison with the synthetic material, we aimed to convert 2 to a fully characterised metabolite.

Further variations in the structures of the ambruticins arise from the presence of different groups at C-5, for example ambruticin S (4) has a 5β-alcohol rather than the 5α-alcohol proposed to be present in ambruticin F (2). Spectral data have been reported for the natural product ambruticin S (4) and it has been a target for total synthesis.^15–19^ To correlate our synthetic ambruticin F with ambruticin S, a biomimetic oxidation/reduction sequence was undertaken (Scheme 6).^22^ Reaction of dihydroxy ester 42 with Fétizon's reagent (Ag_2_CO_3_ on Celite) led to selective oxidation of the 5-axial alcohol to ketone 43 in 54% yield, whereas with DMP in DCM the ketone was isolated in only 33% yield.^14,38^ Reduction of ketone 43 with NaBH_4_ in MeOH gave a separable mixture of 1,2-*syn*-diol (ambruticin F ethyl ester 42) in 35% yield and 1,2-*anti*-diol (ambruticin S ethyl ester) in 47% yield. Finally, hydrolysis of the ethyl ester of ambruticin S completed the total synthesis of ambruticin S (4), and all spectral data were consistent with those reported for the natural product as well as products from previous total syntheses (ESI, Fig. S5 and S6†).^17,19,38^

### Engineering the ambruticin biosynthetic pathway

**Fig. 1:**
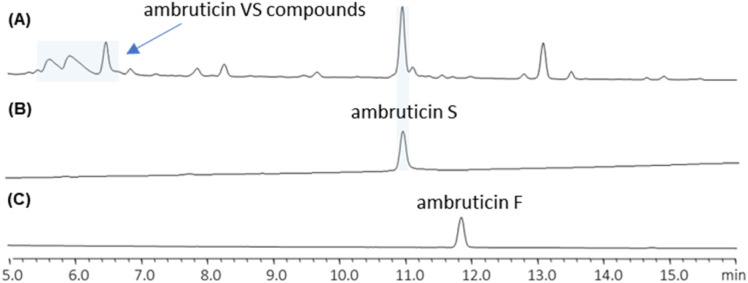
HPLC traces of (A) wild-type *S. cellulosum* So ce10, (B) synthetic standard of ambruticin S (4) and (C) synthetic standard of ambruticin F (2).

We found that cultures of the wild-type strain of *S. cellulosum* So ce10 produce predominantly ambruticin S and the VS series of compounds, and ambruticin F could not be detected by LC-MS in culture extracts (Fig. 1A). Hence, to investigate production of both ambruticin F (2) and 20,21-dihydroambruticin F (9), it was necessary to engineer the biosynthetic pathway in *S. cellulosum* So ce10.

Strains of the genus *Sorangium* are, however, very slow growing, notoriously difficult to ferment and challenging to genetically engineer. Gene disruption of So ce10 has only been successfully achieved by Reeves and co-workers in 2006 and since then no further genetic engineering of this strain has been reported.^22^ In their study, target genes were disrupted through conjugation of a plasmid that could insert into the middle of the gene by a single recombination event. Mutants generated by this method could potentially undergo a second homologous recombination event and revert to the wild-type. To circumvent the challenges of conjugation and lability of mutants obtained, we developed an efficient electroporation method for transferring DNA into So ce10 cells and introduced a plasmid that contains an antibiotic selection marker flanked by upstream and downstream fragments of the target gene. This superior approach led to far more stable mutant strains generated *via* a double crossover recombination event.

**Fig. 2:**
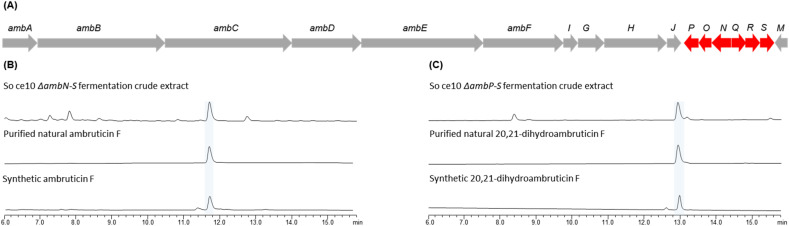
(A) Organisation of the ambruticin biosynthetic gene cluster where genes disrupted in this study are highlighted in red. (B) HPLC traces of the Δ*ambN-S* mutant of *S. cellulosum* So ce10 compared with synthetic standard of ambruticin F (2). (C) HPLC traces of the Δ*ambP-S* mutant of *S. cellulosum* So ce10 compared with synthetic standard of 20,21-dihydroambruticin F (9).

Previous studies had shown that disrupting *ambQ* from the ambruticin biosynthetic gene cluster led to accumulation of ambruticin F but along with ambruticin S and the VS series of compounds found in the wild-type strain, potentially due to the function of AmbQ being complemented by AmbN.^22^ This led us to propose that disruption of both *ambQ* and *ambN*, as well as other downstream genes, would afford ambruticin F as the major metabolite. Therefore, the *ΔambN-S* mutant strain of *S. cellulosum* So ce10 was constructed by replacing the continuous region of *ambN*, *ambQ*, *ambR* and *ambS* genes with a hygromycin selection marker (Fig. 2A). Fermentation of the *ΔambN-S* mutant gave ambruticin F as the major product which was isolated and characterised (Fig. 2B). The NMR data of the purified natural ambruticin F matched perfectly with those of the synthetic sample confirming the structure of the natural product (ESI, Fig. S3 and S4†).

Reeves proposed that 20,21-dehydrogenation of THPs to DHPs in the ambruticins is catalysed by the two-component enzyme system including the Rieske oxygenase AmbP and the NAD(P)/FAD-dependent oxidoreductase AmbO and indicated that this intriguing selective desaturation may occur at various points in the biosynthetic pathway.^22^ To investigate whether these enzymes could indeed be implicated in the conversion of 20,21-dihydroambruticin F to ambruticin F, cultures of *S. cellulosum* So ce10 were grown in which the continuous region of *ambP*, *ambO*, *ambN*, *ambQ*, *ambR* and *ambS* genes (designated as the *ΔambP-S* mutant) were disrupted. Analysis of the crude extract by HPLC (Fig. 2C) revealed one major product which was isolated and NMR analysis and comparison with synthetic material confirmed that 20,21-dihydroambruticin F (9) had accumulated. Similar to the *ΔambP-S* mutant in which the *ambP* and *ambO* genes were deleted together, deletions of *ambP* and *ambO* individually from the *ΔambN-S* mutant, designated as *ΔambN-S*/*ΔambP* and *ΔambO-S* respectively, also yielded 9 as the major metabolite (ESI, Fig. S1 and S2†). These studies fully support the important role of AmbP and AmbO as the key enzymes involved in desaturation of tetrahydropyrans leading to 20,21-alkenes in ambruticin biosynthesis.

**Fig. 3:**
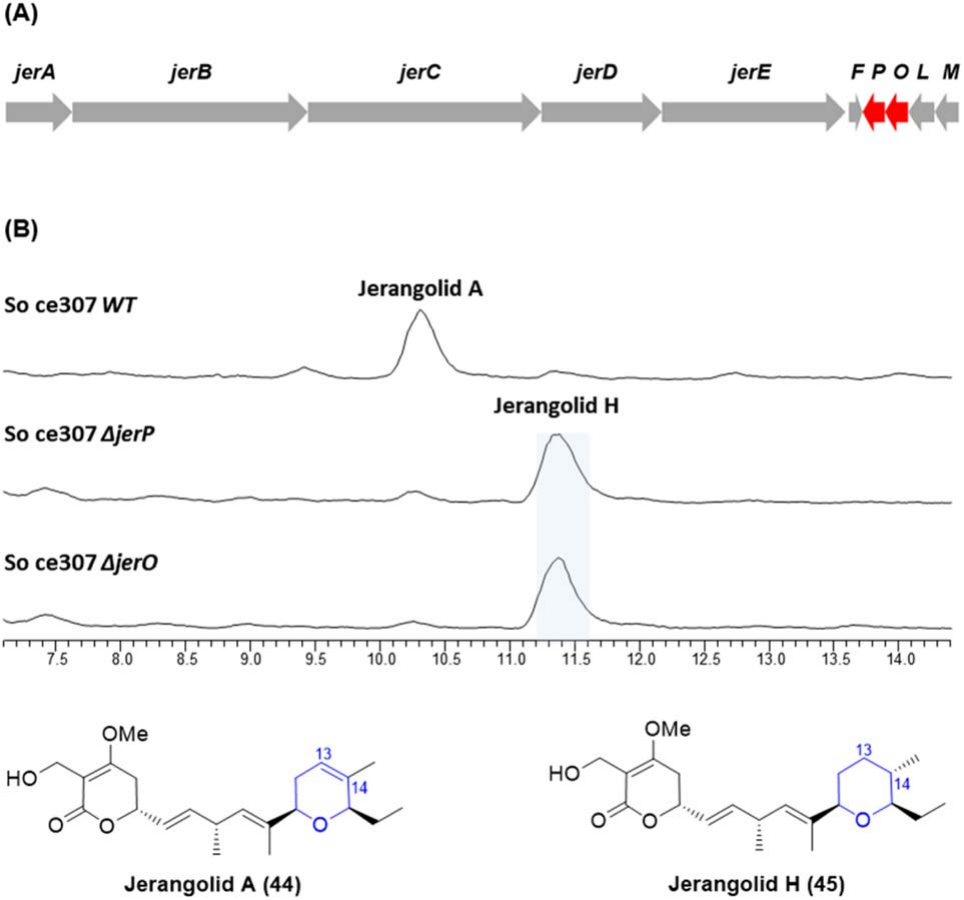
(A) Organisation of the jerangolid biosynthetic gene cluster. (B) HPLC traces of the wild-type, Δ*jerP* and Δ*jerO* mutants of *S. cellulosum* So ce307.

In a parallel experiment, deletions of the *jerP* and *jerO* genes were carried out in the closely related jerangolid biosynthetic pathway in *S. cellulosum* So ce307 (Fig. 3A). Both JerP and JerO share 78% sequence identity to AmbP and AmbO.^22^ Compared with the production of jerangolid A (44) by wild-type *S. cellulosum* So ce307, both the *ΔjerP* and *ΔjerO* mutants produced jerangolid H (45) lacking the 13,14-double bond as the major compound (Fig. 3B).

**Fig. 4:**
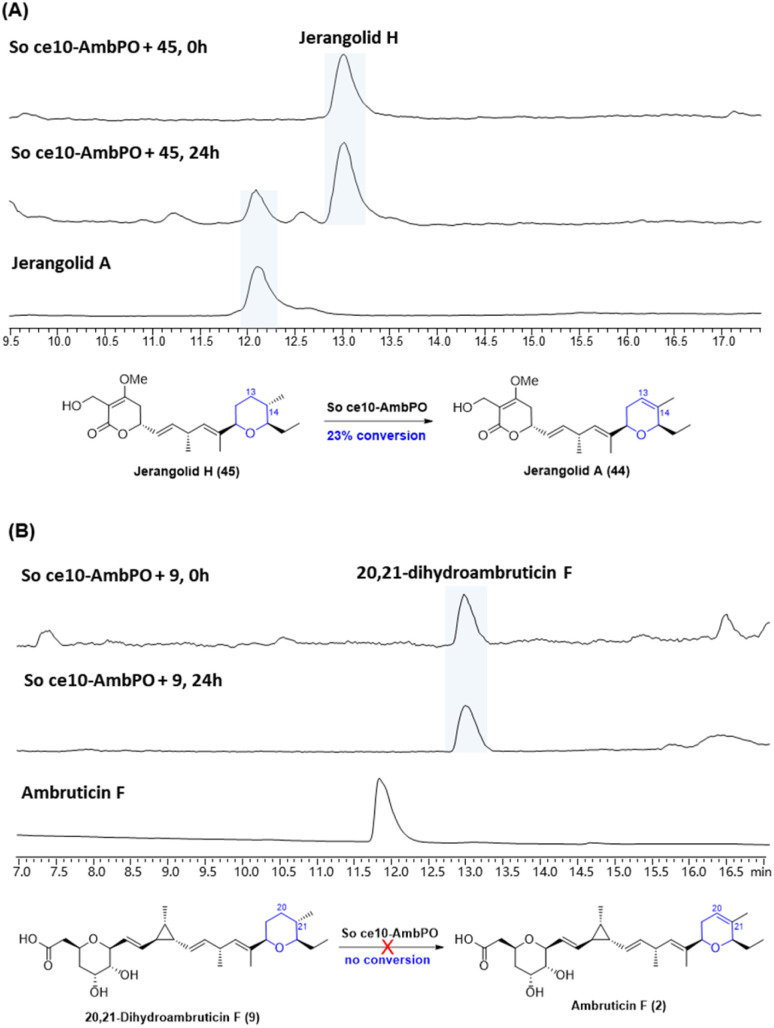
(A) Biotransformation of jerangolid H (45) by So ce10-AmbPO. (B) Biotransformation of 20,21-dihydroambruticin F (9) by So ce10-AmbPO.

Note: During the review process of this manuscript, Guth *et al.* reported reconstitution of the activity of JerP and JerO by extensive optimisation of the whole-cell biotransformation conditions in *E. coli*, similar conversion of jerangolid H to jerangolid A was observed.^39^

With trisubstituted tetrahydropyrans 20,21-dihydroambruticin F (9) and jerangolid H (45) in hand, we next turned to biotransformation assays with AmbP and AmbO. Despite various attempts, no clear turnover was observed with either purified enzymes of AmbP and AmbO or their expressing *E. coli* whole cells. We therefore further engineered the *ΔambN-S* mutant strain and deleted all other ambruticin biosynthetic genes except *ambP* and *ambO*, thus generating a *Sorangium* strain that only contains *ambP* and *ambO* at the *amb* BGC region (designated as So ce10-AmbPO). Interestingly, feeding jerangolid H to this *Sorangium* strain showed conversion to jerangolid A (Fig. 4A), whereas 20,21-dihydroambruticin F (9) was recovered unchanged in an analogous feeding experiment with So ce10-AmbPO (Fig. 4B). These results are inconsistent with the original proposal that the desaturation step may occur late in the biosynthetic pathway.^22^ Bearing in mind that ambruticin J (1) with the 20,21-alkene has been isolated from the *ΔambJ* mutant,^22^ desaturation is now proposed to happen at an earlier stage of the biosynthetic pathway, most likely before the western THP ring is formed.

## Conclusions

In summary, we have completed the first total synthesis of 20,21-dihydroambruticin F (9), whereby three fragments were combined by two Julia–Kocienski olefinations. The two THP rings were constructed on multi-gram scales by a biomimetic epoxidation–cyclisation strategy for the C1–C8 fragment and a Prins cyclisation for the second ring. The modular approach was readily adapted to allow completion of the first total synthesis of ambruticin F (2) which in turn was converted to ambruticin S (4). These synthetic studies provided key standards to enable investigation of dihydropyran formation in ambruticin biosynthesis. Cultures of wild-type *S. cellulosum* So ce10 produced mainly ambruticin S and the VS series of metabolites. A method was developed to carry out gene knockout experiments reliably which revealed that the Δ*ambP-S* mutant of *S. cellulosum* accumulated the novel bisTHP polyketide 20,21-dihydroambruticin F whilst the Δ*ambN-S* mutant gave ambruticin F with the 20,21-alkene as the major metabolite. Similar results were obtained when *ambP* and *ambO* were deleted individually in these strains. These studies confirm that AmbP and AmbO (a Rieske enzyme and flavin-dependent monooxygenase respectively) are implicated in 20,21-alkene formation. Furthermore, the results of feeding studies with *Sorangium* strains containing only *ambP* and *ambO* are in accord with desaturation occurring prior to formation of the C3–C7 tetrahydropyran in ambruticin biosynthesis, laying the foundation for future investigations into the timing and mechanism of this intriguing biotransformation.

## Data availability

All the data supporting this article have been included in the ESI.†

## Author contributions

MPC, LW and CLW co-ordinated the project. JIB and BR conducted all the synthetic work (under the supervision of CLW) and XZ and KG completed all the biosynthetic studies (under the supervision of LW). JIB, MPC, LW and CLW wrote the manuscript.

## Conflicts of interest

There are no conflicts to declare.

## Supplementary Material

SC-015-D4SC00720D-s001
