# Supplementary material for: Combining total synthesis and genetic engineering to probe dihydropyran formation in ambruticin biosynthesis
Source: Chem Sci. 2024 Mar 12;15(14):5319–26. doi: 10.1039/d4sc00720d (PMC10988584; doi:10.1039/d4sc00720d)
Supplement: SC-015-D4SC00720D-s001 [file SC-015-D4SC00720D-s001.pdf]

Electronic Supplementary Material (ESI) for Chemical Science.
This journal is © The Royal Society of Chemistry 2024

Supporting Information

Combining Total Synthesis and Genetic Engineering to Probe Dihydropyran Formation in Ambruticin Biosynthesis

James I. Bowen,[a] Xiaotong Zhong,[b] Kaining Gao,[b,c] Benjamin Reed,[a] Matthew P. Crump,[a] Luoyi Wang[b]* and Christine L. Willis[a]*

[a]School of Chemistry, University of Bristol, Cantock's Close, Bristol, BS8 1TS, United Kingdom

[b]CAS Key Laboratory of Microbial Physiological and Metabolic Engineering, State Key Laboratory of Microbial Resources, Institute of Microbiology, Chinese Academy of Sciences, Beijing 100101, China

[c]School of Life Sciences, Yunnan University, Kunming 650500, China

Contents

1. General Experimental 3

2. Genetic Engineering of *Sorangium* Strains and Biotransformation 4

2.1 Gene disruption in *Sorangium cellulosum* So ce10 4

2.2 General fermentation procedure for mutant strains of *Sorangium cellulosum* So ce10 4

2.3 Isolation and purification of 20,21-dihydroambruticin F and ambruticin F 4

2.4 Isolation and purification of jerangolid H and jerangolid A 4

2.5 Biotransformation of jerangolid H and 20,21-dihydroambruticin F 5

3. Synthetic Procedures 5

3.1 Synthesis of Aldehyde 12 5

3.2 Synthesis of Sulfone 13 13

3.3 Synthesis of Aldehyde 10 15

3.4 Synthesis of Ketone 32 17

3.4.1 Synthesis of Ketone 32 *via* Asymmetric Hydrogenation 17

3.4.2 Synthesis of Ketone 32 *via* Prins Cyclisation 19

3.5 Synthesis of Sulfone 11 24

3.6 Synthesis of 20,21-Dihydroambruticin F 27

3.7 Synthesis of Ambruticin F and S 31

4. Comparison of NMR data for 20,21-Dihydroambruticin F 38

5. Comparison of NMR data for Ambruticin F 41

6. Comparison of NMR data for Ambruticin S 44

7. References 46

8. NMR Appendix 47

1. General Experimental

All reactions were carried out using standard Schlenk syringe-septa techniques in flame dried glassware under a positive pressure of nitrogen in anhydrous solvents unless otherwise stated. Reagents were purchased from commercial suppliers and used without further purification unless reported. Anhydrous THF, Et_2O, hexane, DCM, toluene and MeCN were dried by passing through a modified Grubbs system of alumina columns and stored under nitrogen. MeOH was dried by distillation from calcium hydride and stored under nitrogen and over 3Å molecular sieves. Degassed solvents were prepared by sparging with nitrogen. Analytical thin layer chromatography (TLC) was carried out on Merck silica gel 60 F_{254} analytical plates and were developed using UV fluorescence (254 nm) or $KMnO_4$ / Δ. Flash column chromatography was carried out on Sigma Aldrich silica gel 60 Å (43-63 µm) and an organic solvent system as stated.

Infrared spectra were recorded on a Perkin-Elmer FT-IR spectrometer spectrum 2 with selected peaks of interested reported as absorption maxima (cm^{-1}). Mass spectrometry (MS) and high-resolution mass spectrometry (HRMS) were performed using electrospray ionisation (ESI) on a Bruker microOTOF II (TOF) or atmospheric pressure chemical ionisation (APCI) on a Thermo Scientific Orbitrap Elite (LC-Orbitrap). Optical rotation was measured on a Bellingham and Stanley Ltd. ADP220 polarimeter and is quoted in (° ml)(g dm)$^{-1}$. NMR spectra were recorded on Varian 400-MR (400 MHz), Jeol ECS400 (400 MHz), Jeol ECZ400 (400 MHz), JeolVAR ECZ400 (400 MHz), BrukerNano400 (400 MHz), Bruker Avance III HD 500 Cryo (500 MHz), Bruker Neo 600 Cryo (600 MHz), and Bruker Avance III HD Cryo700 (700 MHz) spectrometers at ambient temperature. Spectra were recorded in deuterochloroform referenced to residual $CHCl_3$ (^1H, 7.26 ppm; ^{13}C, 77.2 ppm), deuterated methanol referenced to residual MeOH (^1H, 3.30 ppm; ^{13}C, 49.0 ppm) or deuterated acetone referenced to residual acetone (^1H, 2.09 ppm; ^{13}C, 30.6 ppm). Chemical shifts (δ) are reported in parts per million (ppm) and coupling constants (*J*) are reported in Hertz (Hz). The following abbreviations are used to describe multiplicity: s (singlet), d (doublet), t (triplet), q (quartet), br. (broad), ap. (apparent). For clarity, the numbering of atoms does not correspond to the compound names.

Size exclusion column chromatographic separations were carried out by using Sephadex LH-20 (Cytiva) as packing materials. LC-MS data were obtained on a SHIMADZU LCMS system comprising SHIMADZU SIL-20A autosampler, SHIMADZU LC-20AD HPLC pump, SHIMADZU SPD-M40 Diode Array detector and SHIMADZU LCMS-2010 mass spectrometer. HPLC grade H_2O and MeCN were added with 0.1% formic acid as solvent system. Analytical LC-MS data were obtained using a Phenomenex Kinetex column (C18, 150 x 4.60 mm, 5 µm) at a flow rate of 1 mL/min. Preparative HPLC purification were carried out using a SilGreen column (C18, 250 x 10 mm, 5 µm) at a flow rate of 4 mL/min.

2. Genetic Engineering of *Sorangium* Strains and Biotransformation

2.1 Gene disruption in *Sorangium cellulosum* So ce10

A plasmid containing the antibiotic selection marker flanked by the upstream and downstream fragments of the target gene or region was constructed and introduced into *Sorangium* cells *via* electroporation. Double crossover clones with the target gene or region replaced by the hygromycin or tetracycline antibiotic selection marker were screened by PCR on HS agar (0.15% Casitone, 0.1% KNO_3, 0.1% $MgSO_4 \cdot 7H_2O$, 0.008% Fe-EDTA, 0.4% glucose, 0.0075% $CaCl_2 \cdot 2H_2O$, 0.00625% K_2HPO_4, 1.5% agar) containing 100 µg/mL of hygromycin or 1.25 µg/mL of tetracycline.

2.2 General fermentation procedure for mutant strains of *Sorangium cellulosum* So ce10

Mutant strains of *Sorangium cellulosum* So ce10 were inoculated on HS agar plates and incubated for 3 days at 30 °C. Seed medium was inoculated in a 500 mL flask with 100 mL of liquid HS medium by scraping colonies from the HS agar plate and incubated for 2-3 days at 220 rpm at 30 °C. Production fermentation was inoculated with 20% of seed culture in SF1-P medium (0.3% soy peptone, 0.6% fructose, 0.1% $MgSO_4 \cdot 7H_2O$, 0.1% $CaCl_2 \cdot 2H_2O$, 0.008% ferric citrate and 0.05 M HEPES, pH 7.6, ferric citrate and HEPES were filter sterilized and added after autoclaving). The culture was incubated at 30°C at 220 rpm for 7 days and supplemented with 0.5 g/L of fructose every 48 hours, and then extracted with EtOAc three times. The combined EtOAc extracts were evaporated *in vacuo* to give a crude extract, which was subjected to LC-MS analysis or further purification.

2.3 Isolation and purification of 20,21-dihydroambruticin F and ambruticin F

A 2.0 L scale fermentation of *ΔambP-S* mutant of *Sorangium cellulosum* So ce10 was carried out as per the general procedure described above. The crude extract was purified by Sephadex LH-20 column chromatography eluting with MeOH to give a crude 20,21-dihydroambruticin F fraction, which was further purified by HPLC eluting with a gradient of 70 to 95% MeCN in water over 20 min to yield 20,21-dihydroambruticin F (7 mg). Ambruticin F was isolated from the fermentation of *ΔambN-S* mutant of *Sorangium cellulosum* So ce10 using a similar purification protocol at a yield of 2 mg/L.

2.4 Isolation and purification of jerangolid H and jerangolid A

Genetic manipulation in *Sorangium cellulosum* So ce307 and its fermentation procedure followed similar protocols for those of *S. cellulosum* So ce10. A 1.0 L scale fermentation of the *ΔjerP* or *ΔjerO* mutant of *Sorangium cellulosum* So ce307 was carried out as per the general procedure. The crude extract was purified by Sephadex LH-20 column chromatography eluting with MeOH to give a crude jerangolid H fraction, which was further purified by HPLC eluting with a gradient of 50 to 95% MeCN in water over 20 min to yield jerangolid H (2 mg). Jerangolid A was isolated from the fermentation of

wild-type *Sorangium cellulosum* So ce307 using a similar purification protocol at a yield of 1.1 mg/L. ^{1}H NMR spectra on page 86.

2.5 Biotransformation of jerangolid H and 20,21-dihydroambruticin F

100 mL of the So ce10-AmbPO strain in SF1-P medium was cultured at 30 °C for 24h. 2 mg of the substrate jerangolid H or 20,21-dihydroambruticin F dissolved in 200 µl of MeOH was then added, and the culture was further incubated at 30 °C for 24h. Equal volumes of EtOAc were used for extraction and then removed *in vacuo*. The samples were redissolved in MeOH and injected for LC-MS analysis.

3. Synthetic Procedures

3.1 Synthesis of Aldehyde 12

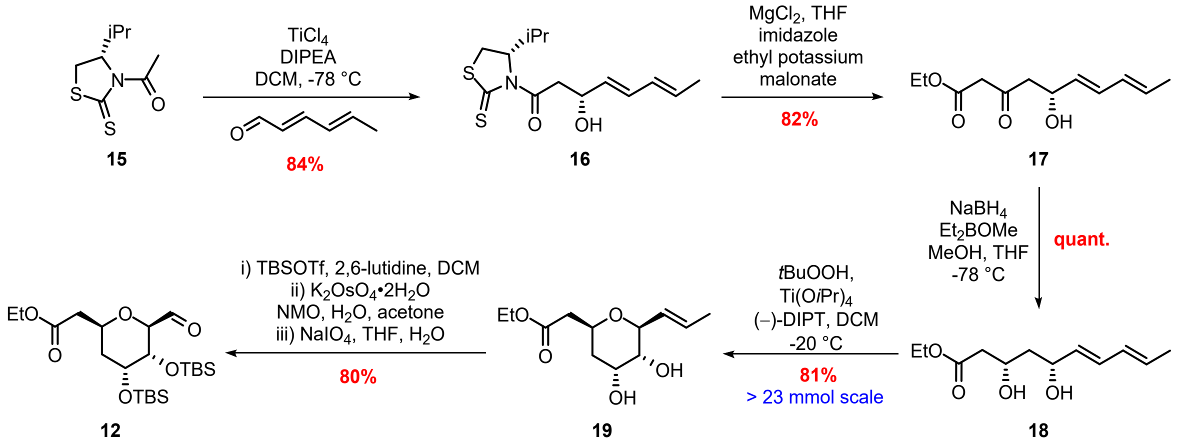

(*S*)-4-Isopropylthiazolidine-2-thioneacid (S1)

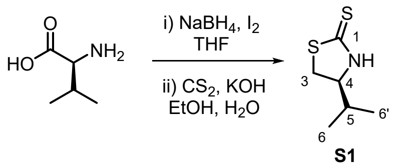

L-Valine (8.03 g, 68.3 mmol) was dissolved in THF (180 mL) under nitrogen and cooled to 0 °C then $NaBH_4$ (6.20 g, 163.9 mmol) was added in one portion. After stirring for 5 minutes, iodine (17.30 g, 68.3 mmol) in THF (20 mL) was added dropwise over 20 minutes. The reaction mixture was warmed slowly to room temperature and then refluxed for 24 hours. The reaction mixture was cooled to room temperature and methanol was added until the solution became clear and the solvent was removed *in vacuo*. The crude material was dissolved in 1 M KOH solution (200 mL) and stirred for 4 hours when the solution was extracted with EtOAc (3 × 200 mL). The combined organic layers were washed with brine (50 mL), dried over $MgSO_4$ and the solvent removed *in vacuo* to afford L-valinol (5.31 g, 75%) as a colourless oil. The crude product was dissolved in ethanol (17 mL) and CS_2 (7.85 mL) was added. KOH

(7.64 g, 136.1 mmol) in water/MeOH, 1:1 (50 mL) was added by an addition funnel over 20 minutes. The reaction mixture was refluxed for 3 days before being cooled to room temperature and the solvent removed *in vacuo*. The solution was dissolved in 2 M HCl (30 mL) and extracted with DCM (3 × 100 mL). The combined organic layers were dried over $MgSO_4$ and the solvent removed *in vacuo* to afford **S1** (6.50 g, 80%) as a yellow solid; $[\alpha]_D^{23}$ = -41.0 (*c* 1, $CHCl_3$), lit.[1] $[\alpha]_D^{20}$ = -35.9 (*c* 1, $CHCl_3$); ν_{max} (UATR) 3176, 2960, 2869, 1661, 1482, 1268, 1029, 977; δ_H (400 MHz, $CDCl_3$) 0.99 (3H, d, *J* 6.8, 6-H_3), 1.03 (3H, d, *J* 6.8, 6′-H_3), 1.92 – 2.02 (1H, m, 5-H), 3.31 (1H, dd, *J* 11.1, 8.4, 3-*H*H), 3.50 (1H, dd, *J* 11.1, 8.2, 3-H*H*), 4.01 – 4.08 (1H, m, 4-H), 8.22 (1H, s, NH); δ_C (101 MHz, $CDCl_3$) 18.4 (C-6), 19.0 (C-6′), 32.2 (C-5), 36.2 (C-3), 70.2 (C-4), 201.3 (C-1); m/z (ESI): $[M+H]^+$ = 175.1. Data consistent with the literature.[1]

(*S*)-1-(4-Isopropyl-2-thioxothiazolidin-3-yl)ethan-1-one (15)

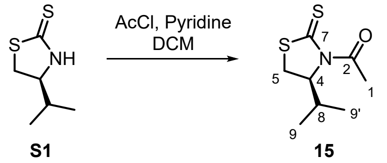

Auxiliary **S1** (1.40 g, 8.70 mmol) was dissolved in DCM (35 mL) under an atmosphere of nitrogen then AcCl (0.92 mL, 13.0 mmol) was added. Pyridine (1.05 mL, 13.0 mmol) was added dropwise and the reaction mixture was stirred for 2 hours. The reaction mixture was filtered, and the solvent removed *in vacuo*. The crude material was purified by flash column chromatography (10% EtOAc in petroleum ether 60:40) to afford acylated auxiliary **15** (1.77 g, quant.) as a yellow oil; $[\alpha]_D^{23}$ = +412.0 (*c* 1, $CHCl_3$), lit.[1] $[\alpha]_D^{20}$ = +442.1 (*c* 1, $CHCl_3$); δ_H (400 MHz, $CDCl_3$) 0.97 (3H, d, *J* 6.9, 9-H_3), 1.05 (3H, d, *J* 6.8, 9′-H_3), 2.29 – 2.43 (1H, m, 8-H), 2.76 (3H, s, 1-H_3), 3.02 (1H, dd, *J* 11.5, 1.1, 5-*H*H), 3.50 (1H, dd, *J* 11.5, 8.0, 5-H*H*), 5.11 – 5.17 (1H, m, 4-H); δ_C (101 MHz, $CDCl_3$) 17.9 (C-9), 19.2 (C-9′), 27.1 (C-1), 30.6 (C-8), 30.9 (C-5), 71.4 (C-4), 170.9 (C-2), 203.4 (C-7); m/z (ESI): $[M+H]^+$ = 204.05. Data consistent with the literature.[1]

(*R*,4*E*,6*E*)-3-Hydroxy-1-((*S*)-4-isopropyl-2-thioxothiazolidin-3-yl)octa-4,6-dien-1-one (16)

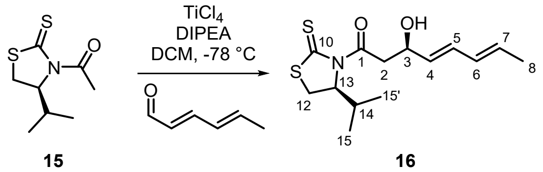

Acylated auxiliary **15** (2.0 g, 9.84 mmol) was dissolved in DCM (100 mL) under nitrogen and cooled to -78 °C when $TiCl_4$ (1 M in DCM, 10 mL, 9.84 mmol) was added dropwise over 15 minutes. The reaction mixture was stirred for 20 minutes then DIPEA (2.1 mL, 11.8 mmol) was added dropwise. The reaction mixture was stirred for 1 hour then *trans*,*trans*-hexadienal (1.1 mL, 9.84 mmol) was added dropwise.

After stirring at -78 °C for 1 hour, aqueous saturated ammonium chloride (25 mL) was added and the reaction mixture was stirred for a further hour at room temperature. The solution was washed with DCM (3 × 50 mL) dried over $MgSO_4$ and the solvent removed *in vacuo*. The crude material was purified by flash column chromatography (18% EtOAc in petroleum ether 60:40) to afford aldol product **16** (2.48 g, 84%) as a yellow oil; $[\alpha]_D^{25}$ = +182.0 (*c* 1, Acetone); ν_{max} (film) 3430, 2962, 1689, 1468, 1162, 725; δ_H (400 MHz, $CDCl_3$) 0.97 (3H, d, *J* 6.9, 15-H_3), 1.05 (3H, d, *J* 6.8, 15'-H_3), 1.75 (3H, d, *J* 6.5, 8-H_3), 2.30 – 2.41 (1H, m, 14-H), 3.02 (1H, d, *J* 11.5, 12-*H*H), 3.33 (1H, dd, *J* 17.5, 8.8, 2-*H*H), 3.51 (1H, dd, *J* 11.5, 7.5, 12-H*H*), 3.61 (1H, dd, *J* 17.5, 3.0, 2-H*H*), 4.64 – 4.78 (1H, m, OH), 5.14 (1H, ap. t, *J* 7.5, 13-H), 5.60 (1H, dd, *J* 15.3, 6.2, 4-H), 5.67 – 5.78 (1H, m, 7-H), 5.98 – 6.09 (1H, m, 6-H), 6.24 (1H, dd, *J* 15.3, 10.5, 5-H); δ_C (101 MHz, $CDCl_3$) 18.0 (C-15), 18.3 (C-8), 19.3 (C-15'), 30.8 (C-12), 31.0 (C-14), 45.5 (C-2), 68.7 (C-3), 71.6 (C-13), 130.7 (C-4), 130.8 (C-6 and C-7), 131.3 (C-5), 172.6 (C-1), 203.2 (C-10); HRMS (ESI) calc. for [$C_{14}H_{21}NO_2S_2Na$] 322.0906 Found 322.0898.

Ethyl (*R*,6*E*,8*E*)-5-hydroxy-3-oxodeca-6,8-dienoate (17)

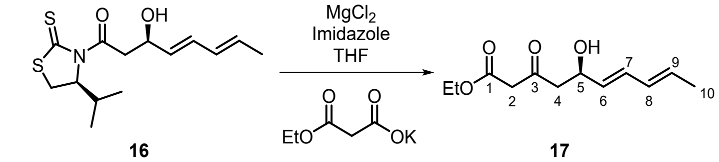

Potassium-3-ethoxy-2-methyl-3-oxopropanoate (5.19 g, 30.49 mmol) and $MgCl_2$ (1.45 g, 15.24 mmol) were added to a solution of aldol product **16** (4.15 g, 13.86 mmol) in THF (50 mL) under nitrogen and stirred for 45 minutes. Imidazole (1.04 g, 15.24 mmol) was added and the reaction mixture was stirred for 72 hours. The mixture was diluted with EtOAc (100 mL) and washed with 1 M HCl (50 mL). The aqueous layer was extracted with EtOAc (3 × 50 mL). The combined organic layers were washed with aqueous saturated sodium hydrogen carbonate (100 mL) and the combined organic layers were dried over Na_2SO_4 and the solvent removed *in vacuo*. The crude product was purified by flash column chromatography (5% Et_2O in DCM) to afford **17** (2.58 g, 82%) as a yellow oil; $[\alpha]_D^{22}$ = +6.0 (*c* 1, Acetone); ν_{max} (film) 3443, 2981, 2914, 1737, 1709, 988; δ_H (400 MHz, $CDCl_3$) 1.27 (3H, t, *J* 7.2, OCH_2CH_3), 1.73 – 1.76 (3H, m, 10-H_3), 2.68 – 2.74 (1H, br. s, OH), 2.74 – 2.80 (2H, m, 4-H_2), 3.47 (2H, s, 2-H_2) , 4.19 (2H, q, *J* 7.2, OCH_2CH_3), 4.61 (1H, ap. q, *J* 6.5, 5-H), 5.53 (1H, ddq, *J* 15.2, 6.4, 0.7, 9-H), 5.71 (1H, dd, *J* 15.0, 6.5, 6-H), 5.97 – 6.04 (1H, m, 8-H), 6.17 – 6.26 (1H, m, 7-H); δ_C (101 MHz, $CDCl_3$) 14.2 (OCH_2CH_3), 18.3 (C-10), 49.8 (C-4), 50.2 (C-2), 61.7 (OCH_2CH_3), 68.4 (C-5), 130.6 (C-9), 130.7 (C-6), 130.9 (C-8), 131.4 (C-7), 167.1 (C-1), 203.0 (C-3); HRMS (ESI) calc. for [$C_{12}H_{18}O_4Na$] 249.1097 Found 249.1093.

Ethyl (3*S*,5*R*,6*E*,8*E*)-3,5-dihydroxydeca-6,8-dienoate (18)

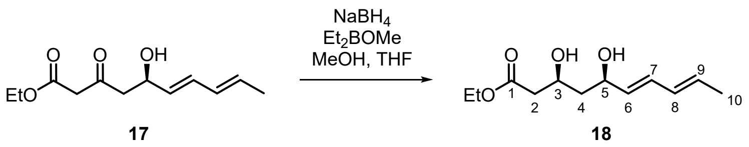

Ketone **17** (1.01 g, 4.42 mmol) was dissolved in THF (25 mL) and MeOH (7 mL) under nitrogen and cooled to -78 °C when Et_2BOMe (0.70 mL, 5.30 mmol) was added dropwise and the reaction mixture was stirred for 15 minutes. $NaBH_4$ (0.19 g, 5.08 mmol) was added in one portion and the reaction mixture was stirred for 3 hours then AcOH (3 mL) was added and the mixture was warmed to room temperature. The reaction mixture was diluted with EtOAc (30 mL) and the resulting solution was washed with aqueous saturated sodium hydrogen carbonate (20 mL). The organic layer was dried over $MgSO_4$ and the solvent removed *in vacuo* to afford diol **18** (1.02 g, quant.) as a yellow oil; $[\alpha]_D^{22}$ = -8.0 (*c* 1, Acetone); ν_{max} (film) 3396, 2981, 2914, 1716, 1164, 987; δ_H (400 MHz, $CDCl_3$) 1.27 (3H, t, *J* 7.1, OCH_2CH_3), 1.57 – 1.72 (2H, m, 4-H_2), 1.73 – 1.77 (3H, m, 10-H_3), 2.42 – 2.52 (2H, m, 2-H_2), 3.07 (1H, br. s, OH), 3.74 (1H, br. s, OH), 4.16 (2H, q, *J* 7.1, OCH_2CH_3), 4.22 – 4.31 (1H, m, 3-H), 4.42 (1H, m, 5-H), 5.54 (1H, dd, *J* 15.2, 6.6, 6-H), 5.71 (1H, dd, *J* 15.0, 6.8, 9-H), 5.97 – 6.08 (1H, m, 8-H), 6.20 (1H, dd, *J* 15.2, 10.4, 7-H); δ_C (101 MHz, $CDCl_3$) 14.3 (OCH_2CH_3), 18.3 (C-10), 41.8 (C-2), 42.9 (C-4), 61.0 (OCH_2CH_3), 68.5 (C-3), 72.6 (C-5), 130.5 (C-9), 130.85 (C-8), 130.93 (C-7), 132.4 (C-6), 172.7 (C-1); HRMS (ESI) calc. for [$C_{12}H_{20}O_4Na$] 251.1254 Found 251.1252.

The relative stereochemistry in diol **18** was confirmed by NMR analysis of the corresponding acetonide (**S2**), where the ^{13}C chemical shifts of 19.9 and 30.3 ppm for the acetonide methyl groups and 99.0 ppm for the acetal carbon are consistent with a *syn*-diol.[2,3]

Ethyl 2-((4*S*,6*R*)-2,2-dimethyl-6-((*E*)-prop-1-en-1-yl)-1,3-dioxan-4-yl)acetate (S2)

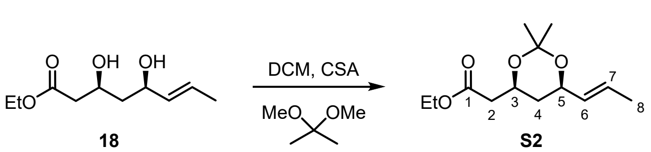

Diol **18** (100 mg, 0.49 mmol) was dissolved in DCM (5 mL) under nitrogen then 2,2-dimethoxypropane (1.5 mL, 12 mmol) and CSA (57 mg, 0.25 mmol) were added and the reaction mixture was stirred at room temperature for 1 hour. The solution was diluted with aqueous saturated $NaHCO_3$ (10 mL), the organic layer separated and the aqueous extracted with DCM (2 × 10 mL). The combined organic layers were washed with brine (10 mL), dried over $MgSO_4$ and the solvent removed *in vacuo* to afford acetonide **S2** (116 mg, 99%) as a yellow oil; $[\alpha]_D^{23}$ = +15.0 (*c* 1, $CHCl_3$); ν_{max} (film) 2997, 1735, 1198, 1164, 965, 949; δ_H (400 MHz, $CDCl_3$) 1.26 (3H, t, *J* 7.1, OCH_2CH_3), 1.29 – 1.36 (1H, m, 4-*H*H), 1.40 (3H, s, $OCCH_3$), 1.49 (3H, s, $OCCH_3$), 1.60 (1H, ap. dt, *J* 12.8, 2.5, 4-H*H*), 1.69 (3H, dd, *J* 6.5, 1.6, 8-H_3), 2.38

(1H, dd, *J* 15.5, 6.1, 2-*H*H), 2.54 (1H, dd, *J* 15.5, 7.0, 2-H*H*), 4.08 – 4.22 (2H, m, O$CH_2$$CH_3$), 4.28 – 4.38 (2H, m, 3-H and 5-H), 5.40 – 5.50 (1H, m, 6-H), 5.72 (1H, dq, *J* 15.5, 6.5, 7-H); δ_C (101 MHz, $CDCl_3$) 14.4 (OCH_2*CH_3*), 18.0 (C-8), 19.9 (CH_3), 30.3 (CH_3), 36.9 (C-4), 41.6 (C-2), 60.7 (O*CH_2*CH_3), 65.9 (C-3), 70.2 (C-5), 99.0 (OCO), 128.2 (C-7), 131.7 (C-6), 171.1 (C-1); HRMS (ESI) calc. for [$C_{13}H_{22}O_4Na$] 265.1410 Found 265.1401.

Ethyl 2-((2*S*,4*R*,5*R*,6*S*)-4,5-dihydroxy-6-((*E*)-prop-1-en-1-yl)tetrahydro-2H-pyran-2-yl)acetate (19)

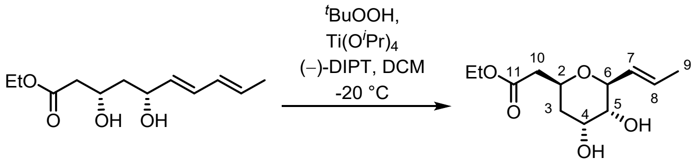

18 **19**

Optimised procedure on < 1 mmol scale of allylic alcohol 18:

In a flame dried flask at -20 °C under nitrogen was added 4 Å molecular sieves (30 mg), allylic alcohol **18** (100 mg, 0.44 mmol) and DCM (2.5 mL) followed by titanium isopropoxide (0.03 mL, 0.09 mmol) and (−)-DIPT (0.03 mL, 0.13 mmol). The reaction mixture was stirred for 30 minutes then 5.5 M ^{t}BuOOH in decane (0.16 mL, 0.88 mmol) was added dropwise and the reaction mixture was stirred at -20 °C for 24 hours. The reaction mixture was quenched with a precooled (0 °C) aqueous solution of $FeSO_4$/citric acid (660 mg of $FeSO_4$ and 220 mg of citric acid in 3 mL of H_2O). The solution was stirred vigorously for 30 minutes at room temperature then extracted with DCM (3 × 30 mL). The combined organic layers were dried over $MgSO_4$ and the solvent removed *in vacuo*. The crude product was purified by flash column chromatography (70% EtOAc in petroleum ether 60:40) to afford tetrahydropyran **19** (87 mg, 81%) as a colourless oil. Data consistent with previously reported.

Optimised scale-up procedure:

In a flame dried flask at -20 °C under nitrogen was added 4 Å powdered molecular sieves (1.60 g), DCM (100 mL) and (−)-DIPT (2.92 mL, 13.93 mmol). The mixture was stirred for 30 minutes then titanium isopropoxide (3.44 mL, 11.61 mmol) was added dropwise. The reaction mixture was stirred for 30 minutes then allylic alcohol **18** (5.30 g, 23.22 mmol) in DCM (15 mL) was added dropwise. The reaction mixture was stirred for a further 30 minutes then 5.5 M ^{t}BuOOH in decane (8.44 mL, 46.44 mmol) was added dropwise and the reaction mixture was stirred at -20 °C for 24 hours. The reaction mixture was poured into a precooled (0 °C) aqueous solution of $FeSO_4$/citric acid (20 g of $FeSO_4$ and 6.4 g of citric acid in 60 mL of H_2O) and the resulting solution was stirred for 10 minutes at room temperature. The mixture was filtered over Celite, washing with EtOAc (500 mL) and water (200 mL). The organic layer was separated and the aqueous extracted with further EtOAc (2 × 500 mL). The combined organic

layers were dried over Na_2SO_4 and the solvent removed *in vacuo*. The crude material was purified by flash column chromatography (50% Et_2O in DCM) to afford tetrahydropyran **19** (4.31 g, 76%, 89% BORSM) as a colourless oil and unreacted allylic alcohol **18** (0.75 g, 14%). Data consistent with previously reported.

Procedure for alternative quench with $Na_2S_2O_3$:

In a flame dried flask at -20 °C under nitrogen was added 4 Å powdered molecular sieves (600 mg), DCM (26 mL) and (−)-DIPT (1.04 mL, 4.97 mmol). The mixture was stirred for 15 minutes then titanium isopropoxide (1.23 mL, 4.14 mmol) was added dropwise. The reaction mixture was stirred for 30 minutes then allylic alcohol **18** (1890 mg, 8.28 mmol) in DCM (15 mL) was added dropwise. The reaction mixture was stirred for a further 30 minutes then 5.5 M ^{t}BuOOH in decane (3.01 mL, 16.56 mmol) was added dropwise and the reaction mixture was stirred at -20 °C for 24 hours. The reaction mixture was quenched with aqueous saturated $Na_2S_2O_3$ (40 mL) then stirred vigorously for 1 hour at 0 °C. The reaction mixture was diluted with water (100 mL) and EtOAc (200 mL) and the layers separated. The aqueous layer was extracted with EtOAc (3 × 150 mL) then the combined organic layers washed with Brine (100 mL), dried over Na_2SO_4 and the solvent removed *in vacuo*. The crude material was purified by flash column chromatography (60% Et_2O in DCM) to afford tetrahydropyran **19** (1167 mg, 58%) as a yellow oil. Data consistent with previously reported.

(2*S*,3*R*,4*R*,6*S*)-6-(2-Ethoxy-2-oxoethyl)-2-((*E*)-prop-1-en-1-yl)tetrahydro-2H-pyran-3,4-diyl diacetate (S3)

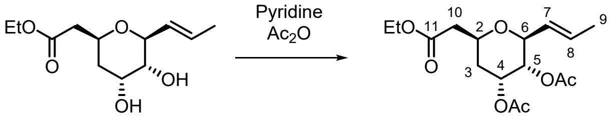

19 **S3**

Diol **19** (30 mg, 0.12 mmol) was dissolved in pyridine (0.25 mL, 3.01 mmol) and Ac_2O (0.11 mL, 1.2 mmol) and stirred under nitrogen for 16 hours. The reaction mixture was diluted with water (10 mL) and extracted with DCM (2 × 15 mL). The combined organic layers were washed sequentially with 2 M HCl (2 × 5 mL), sodium hydrogen carbonate (2 × 10 mL) and brine (25 mL). The combined organics were dried over $MgSO_4$ and the solvent removed *in vacuo*. The crude product was purified by flash column chromatography (40% EtOAc in petroleum ether 60:40) to afford diacetate **S3** (32 mg, 82%) as a colourless oil; $[\alpha]_D^{18}$ = -20.0 (*c* 1, $CHCl_3$); ν_{max} (film) 2981, 2938, 1736, 1370, 1243, 1055; δ_H (400 MHz, $CDCl_3$) 1.24 (3H, t, *J* 7.1, OCH_2CH_3), 1.67 (3H, dd, *J* 6.6, 1.7, 9-H_3), 1.70 – 1.77 (1H, m, 3-*H*H), 1.91 – 1.95 (1H, m, 3-H*H*), 1.96 (3H, s, OAc), 2.13 (3H, s, OAc), 2.38 (1H, dd, *J* 15.5, 5.8, 10-*H*H), 2.58 (1H, dd, *J* 15.5, 7.3, 10-H*H*), 4.11 – 4.17 (3H, m, CH_3CH_2O and 6-H), 4.18 – 4.25 (1H, m, 2-H), 4.66 (1H, dd, *J* 10.1, 3.1, 5-H), 5.34 (1H, ddq, *J* 15.3, 7.2, 1.7, 7-H), 5.40 (1H, ap. q, *J* 3.1, 4-H), 5.72 – 5.82 (1H, m, 8-

H); δ_C (101 MHz, $CDCl_3$) 14.3 (CH_3CH_2O), 18.1 (C-9), 21.0 (OAc), 21.3 (OAc), 35.6 (C-3), 40.6 (C-10), 60.8 (CH_3CH_2O), 67.4 (C-4), 68.8 (C-2), 70.9 (C-5), 75.0 (C-6), 127.8 (C-7), 131.1 (C-8), 170.1 (OAc), 170.4 (OAc), 170.9 (C-11); HRMS (ESI) calc. for [$C_{16}H_{24}O_7$ Na] 315.1414 Found 315.1402.

Upon acetylation of diol **19**, protons 4-H and 5-H exhibited significant downfield shifts, whilst 6-H showed little change in chemical shift (Figure **S1**). This is consistent with the assigned structure of THP **19**.

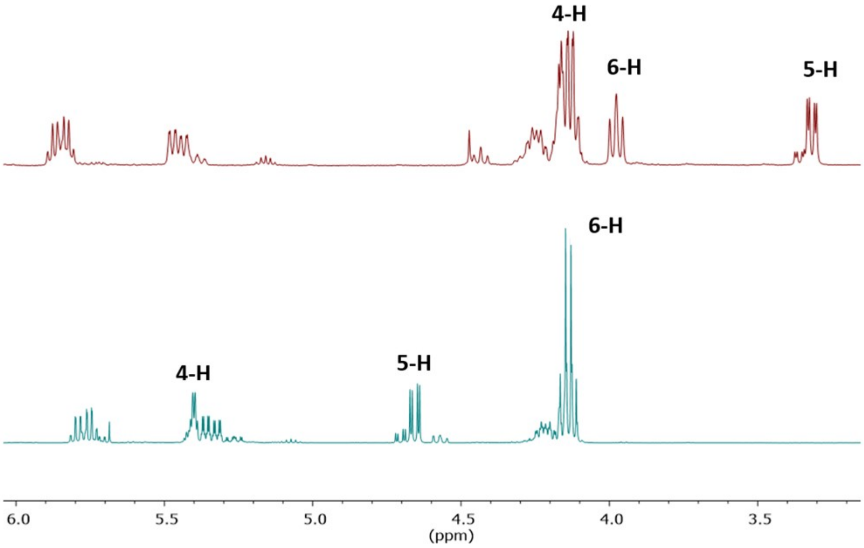

Figure S1. Comparison of key ^{1}H-NMR shifts of diol **19** (Red) and acetylated diol **S3** (Blue) used to confirm product.

Ethyl 2-((2*S*,4*R*,5*S*,6*S*)-4,5-bis(*tert*-butyldimethylsiloxy)-6-((*E*)-prop-1-en-1-yl)tetrahydro-2H-pyran-2-yl)acetate (S4)

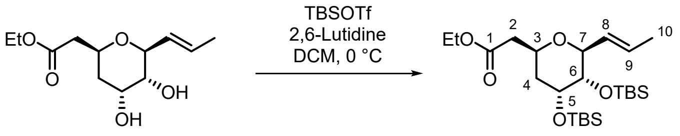

19 **S4**

Diol **19** (3.60 g, 14.74 mmol) was dissolved in DCM (150 mL) under nitrogen and cooled to 0 °C when 2,6-lutidine (10.30 mL, 88.44 mmol) was added and the reaction mixture was stirred for 5 minutes. TBSOTf (13.54 mL, 58.96 mmol) was added dropwise over 15 minutes. The reaction mixture was stirred for 45 minutes at 0 °C then quenched with aqueous saturated sodium hydrogen carbonate solution (50 mL). The resulting solution was diluted with water (100 mL) and DCM (200 mL). The organic layer was separated and the aqueous extracted with DCM (2 × 100 mL). The combined organic layers were washed sequentially with 2 M HCl (100 mL) then brine (100 mL), dried over $MgSO_4$ and the solvent removed *in vacuo*. The crude material was purified by flash column chromatography (8% Et_2O in petroleum ether 60:40) to afford silyl ether **S4** (6.28 g, 90%) as a colourless oil; $[\alpha]_D^{21}$ = -46.0 (*c* 1, $CHCl_3$); ν_{max} (film) 3020, 2954, 2929, 2887, 2857, 1739, 1473, 1214; δ_H (400 MHz, $CDCl_3$) δ 0.00 (3H,

s, $SiCH_3$), 0.02 (3H, s, $SiCH_3$), 0.06 (3H, s, $SiCH_3$), 0.08 (3H, s, $SiCH_3$), 0.86 (9H, s, $SiC(CH_3)_3$), 0.91 (9H, s, $SiC(CH_3)_3$), 1.24 (3H, t, *J* 7.1, $OCH_2\mathit{CH_3}$), 1.53 (1H, ddd, *J* 13.4, 11.4, 2.1, 4-H_{ax}), 1.68 (3H, dd, *J* 6.5, 1.6, 10-H_3), 1.82 (1H, ddd, *J* 13.4, 4.1, 2.1, 4-H_{eq}), 2.34 (1H, dd, *J* 14.8, 7.6, 2-*H*H), 2.60 (1H, dd, *J* 14.8, 6.2, 2-H*H*), 3.28 (1H, dd, *J* 9.2, 2.1, 6-H), 4.00 (1H, ap. dt, *J* 4.1, 2.1, 5-H), 4.07 – 4.18 (3H, m, $O\mathit{CH_2}CH_3$ and 7-H), 4.27 (1H, dddd, *J* 11.4, 7.6, 6.2, 2.1, 3-H), 5.38 (1H, ddq, *J* 15.4, 7.3, 1.6, 8-H), 5.69 (1H, dqd, *J* 15.4, 6.5, 1.0, 9-H); δ_C (101 MHz, $CDCl_3$) -4.4 (2 x $SiCH_3$), -4.2 ($SiCH_3$), -3.4 ($SiCH_3$), 14.4 ($OCH_2\mathit{CH_3}$), 18.2 (C-10), 18.3 ($Si\mathit{C}(CH_3)_3$), 18.4 ($Si\mathit{C}(CH_3)_3$), 26.1 ($SiC(\mathit{C}H_3)_3$), 26.3 ($SiC(\mathit{C}H_3)_3$), 39.7 (C-4), 41.2 (C-2), 60.6 ($O\mathit{C}H_2CH_3$), 67.9 (C-3), 69.6 (C-5), 73.9 (C-6), 76.7 (C-7), 129.2 (C-9), 130.4 (C-8), 171.2 (C-1); HRMS (ESI) calc. for [$C_{24}H_{48}O_5Si_2$] 473.3113 Found 473.3114.

Ethyl 2-((2*S*,4*R*,5*S*,6*R*)-4,5-bis(*tert*-butyldimethylsiloxy)-6-formyltetrahydro-2H-pyran-2-yl)acetate (12)

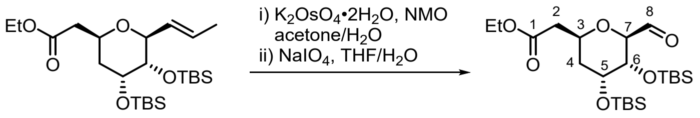

S4 **12**

Alkene **S4** (5.99 g, 12.67 mmol) was dissolved in acetone (50 mL) and water (6.5 mL) then potassium osmate dihydrate (47 mg, 0.13 mmol) and NMO (2.23 g, 19.04 mmol) were added sequentially, and the reaction mixture was stirred at room temperature. After 18 hours the reaction mixture was diluted with EtOAc (200 mL), water (100 mL) and brine (50 mL). The organic layer was separated and the aqueous was extracted with further EtOAc (2 × 150 mL). The combined organics were dried over $MgSO_4$ and the solvent removed *in vacuo*. The crude diol was dissolved in THF (42 mL) and water (32 mL) and $NaIO_4$ (3.80 g, 17.77 mmol) was added portion wise. The reaction mixture was stirred at room temperature for 3 hours then further $NaIO_4$ (3.80 g, 17.77 mmol) was added and the reaction mixture was stirred for 3 hours. The solution was filtered through a pad of Celite and washed with DCM (300 mL). The organic phase was washed with aqueous saturated $Na_2S_2O_3$ (200 mL) and the aqueous extracted with DCM (2 × 200 mL). The combined organic layers were dried over $MgSO_4$ and the solvent removed *in vacuo*. The crude material was purified by flash column chromatography (50% Et_2O in petroleum ether 60:40) to afford aldehyde **12** (5.23 g, 89%) as a colourless oil; $[\alpha]_D^{22}$ = -76.0 (*c* 1, $CHCl_3$); ν_{max} (film) 3019, 2953, 2929, 2887, 2858, 1739, 1214; δ_H (400 MHz, $CDCl_3$) 0.01 (3H, s, $SiCH_3$), 0.05 (3H, s, $SiCH_3$), 0.09 (3H, s, $SiCH_3$), 0.11 (3H, s, $SiCH_3$), 0.89 (9H, s, $SiC(CH_3)_3$), 0.93 (9H, s, $SiC(CH_3)_3$), 1.25 (3H, t, *J* 7.1, $OCH_2\mathit{CH_3}$), 1.57 (1H, ddd, *J* 13.6, 11.5, 2.0, 4-H_{ax}), 1.86 (1H, ddd, *J* 13.6, 4.2, 2.1, 4-H_{eq}), 2.40 (1H, dd, *J* 15.2, 6.8, 2-*H*H), 2.67 (1H, dd, *J* 15.2, 6.8, 2-H*H*), 3.67 (1H, dd, *J* 9.6, 2.0, 6-H), 4.06 (1H, ap. dt, *J* 4.2, 2.0, 5-H), 4.13 (2H, ap. qd, *J* 7.1, 2.2, $O\mathit{CH_2}CH_3$), 4.29 (1H, ap. dtd, *J* 11.5, 6.8, 2.1, 3-H), 4.39 (1H, dd, *J* 9.6, 1.4, 7-H), 9.72 (1H, d, *J* 1.4, 8-H); δ_C (101 MHz, $CDCl_3$) -4.9 ($SiCH_3$), -4.4 ($SiCH_3$), -4.1

(SiCH$_3$), -3.5 (SiCH$_3$), 14.4 (OCH$_2$*C*H$_3$), 18.2 (Si*C*(CH$_3$)$_3$), 18.3 (Si*C*(CH$_3$)$_3$), 26.0 (SiC(*C*H$_3$)$_3$), 26.1 (SiC(*C*H$_3$)$_3$), 39.0 (C-4), 40.6 (C-2), 60.8 (O*C*H$_2$CH$_3$), 68.2 (C-3), 69.4 (C-5), 71.4 (C-6), 79.5 (C-7), 170.8 (C-1), 200.5 (C-8); HRMS (ESI) calc. for [$C_{22}H_{44}O_6Si_2$] 461.2749 Found 461.2761.

3.2 Synthesis of Sulfone 13

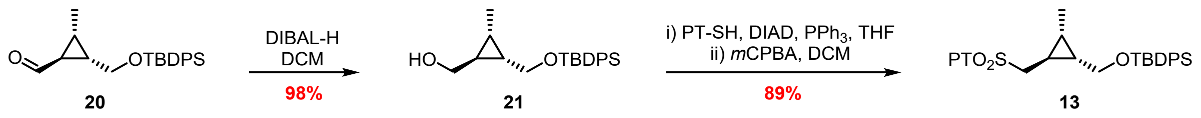

((1*S*,2*S*,3*S*)-2-((*tert*-Butyldiphenylsiloxy)methyl)-3-methylcyclopropyl)methanol (21)

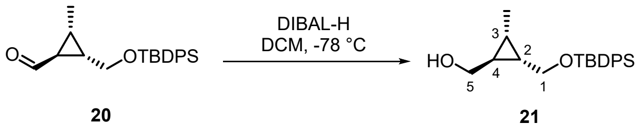

Aldehyde **20**[4] (5.90 g, 16.74 mmol) was dissolved in DCM (170 mL) under nitrogen and cooled to -78 °C then 1 M DIBAL-H in hexanes (18.41 mL, 18.41 mmol) was added dropwise. The reaction mixture was stirred at room temperature for 1 hour, cooled to 0 °C, then quenched with dropwise addition of aqueous saturated sodium potassium tartrate solution (75 mL). The reaction mixture was warmed to room temperature, diluted with water (75 mL) and DCM (50 mL) and stirred vigorously for 1 hour. The organic layer was separated and the aqueous extracted with DCM (2 x 200 mL). The combined organic layers were washed with brine (100 mL), dried over $MgSO_4$ and the solvent removed *in vacuo*. The crude material was purified by flash column chromatography (45% Et_2O in petroleum ether 60:40) to afford alcohol **21** (5.83 g, 98%) as a colourless oil; $[\alpha]_D^{24}$ = +4.0 (*c* 1, CHCl$_3$); ν_{max} (film) 3347, 3018, 2957, 2931, 2858, 1428, 1214; δ_H (400 MHz, CDCl$_3$) 0.66 (1H, m, 4-H), 0.79 – 0.89 (1H, m, 3-H), 0.97 (1H, dddd, *J* 9.1, 7.6, 6.8, 4.7, 2-H), 1.03 – 1.08 (12H, m, 3-CH$_3$ and SiC(CH$_3$)$_3$), 1.23 (1H, m, OH), 3.37 (1H, ddd, *J* 11.7, 7.1, 4.5, 5-*H*H), 3.50 (1H, ap. dt, *J* 11.7, 6.2, 5-H*H*), 3.66 (1H, dd, *J* 11.0, 7.6, 1-*H*H), 3.72 (1H, dd, *J* 11.0, 6.8, 1-H*H*), 7.34 – 7.45 (6H, m, 6 x ArH), 7.65 – 7.71 (4H, m, 4 x ArH); δ_C (101 MHz, CDCl$_3$) 12.9 (CH$_3$-3), 15.8 (C-3), 19.4 (Si*C*(CH$_3$)$_3$), 23.8 (C-2), 27.0 (SiC(*C*H$_3$)$_3$), 27.5 (C-4), 63.3 (C-1), 66.8 (C-5), 127.8 (2 x ArCH), 129.8 (2 x ArCH), 134.1 (ArC), 134.2 (ArC), 135.8 (2 x ArCH); HRMS (ESI) calc. for [$C_{22}H_{30}O_2Si$] 366.1907 Found 377.1911. Data consistent with the literature.[5]

5-((((1*S*,2*R*,3*R*)-2-((*tert*-Butyldiphenylsiloxy)methyl)-3-methylcyclopropyl)methyl)sulfonyl)-1-phenyl-1H-tetrazole (13)

<u>Method 1:</u>

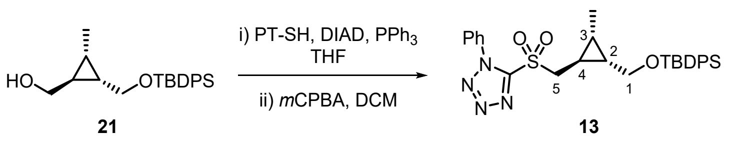

Alcohol **21** (5.31 g, 15.00 mmol), PT-SH (3.48 g, 19.50 mmol) and PPh_3 (5.11 g, 19.50 mmol) were dissolved in THF (150 mL) under nitrogen and cooled the 0 °C then DIAD (3.84 mL, 19.50 mmol) was added dropwise over 10 minutes. The reaction mixture was stirred for 3 hours at room temperature then quenched with aqueous saturated NH_4Cl (150 mL). The resulting solution was extracted with EtOAc (3 x 200 mL), and the combined organic layers were washed with brine (100 mL), dried over Na_2SO_4 and the solvent removed *in vacuo*. The crude material was filtered through a small plug of silica eluting with 50% Et_2O in petroleum ether 60:40 and the solvent removed *in vacuo*. The crude material was dissolved in DCM (75 mL) and cooled to 0 °C then *m*CPBA (70wt%, 12.94 g, 52.50 mmol) was added over 15 minutes. The reaction mixture was stirred at room temperature for 18 hours then quenched with aqueous saturated $Na_2S_2O_3$ (50 mL) and aqueous saturated $NaHCO_3$ (50 mL) then stirred vigorously for 1 hour. The resulting solution was diluted with water (100 mL) and extracted with EtOAc (3 x 200 mL). The combined organic layers were washed with brine (100 mL), dried over $MgSO_4$ and the solvent removed *in vacuo*. The crude material was purified by flash column chromatography (30% Et_2O in petroleum ether 60:40) to afford sulfone **13** (7.34 g, 89%) as a yellow oil; $[\alpha]_D^{22}$ = +4.0 (*c* 1, $CHCl_3$); ν_{max} (film) 3019, 2932, 2858, 1214; δ_H (400 MHz, $CDCl_3$) 0.76 – 0.84 (1H, m, 2-H or 4-H), 0.99 – 1.05 (1H, m, $SiC(CH_3)_3$ and 3-CH_3), 1.05 – 1.08 (1H, m, 3-H), 1.17 – 1.24 (1H, m, 2-H or 4-H), 3.52 – 3.61 (2H, m, 1-*H*H and 5-*H*H), 3.73 (1H, dd, *J* 6.3, 3.4, 1-H*H* or 5-H*H*), 3.77 (1H, d, *J* 6.3 1-H*H* or 5-H*H*), 7.34 – 7.44 (6H, m, ArH), 7.53 – 7.69 (9H, m, ArH); δ_C (101 MHz, $CDCl_3$) 12.4 (CH_3-3), 15.9 (C-2/4), 17.9 (C-3), 19.3 (Si*C*(CH_3)$_3$), 25.3 (C-2/4), 27.0 (SiC(*C*H_3)$_3$), 60.8 (C-1/5), 62.4 (C-1/5), 125.3 (2 x ArCH), 127.9 (2 x ArCH), 129.9 (2 x ArCH), 131.6 (ArCH), 133.2 (ArC), 133.8 (ArC), 133.8 (ArC), 135.7 (2 x ArCH), 153.9 (NCN); HRMS (ESI) calc. for [$C_{29}H_{34}N_4O_3SSiNa$] 569.2019 Found 569.2018. Data consistent with the literature.[5]

Method 2:

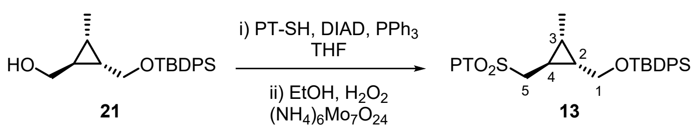

Alcohol **21** (250 mg, 0.71 mmol), PT-SH (164 mg, 0.92 mmol) and PPh_3 (241 mg, 0.92 mmol) were dissolved in THF (7 mL) under nitrogen and cooled the 0 °C then DIAD (0.18 mL, 0.92 mmol) was added dropwise. The reaction mixture was stirred for 3 hours at room temperature then quenched with aqueous saturated NH_4Cl (20 mL). The resulting solution was extracted with EtOAc (3 x 50 mL), and the combined organic layers were washed with brine (50 mL), dried over Na_2SO_4 and the solvent

removed *in vacuo*. The crude material was filtered over a small plug of silica eluting with 50% Et_2O in petroleum ether 60:40 and the solvent removed *in vacuo*. The crude material was dissolved in EtOH (24 mL) and cooled to 0 °C then a premixed solution of molybdate (175 mg, 0.14 mmol) in a 30% aqueous solution of H_2O_2 (0.73 mL, 7.1 mmoL) was added dropwise. The reaction mixture was stirred at room temperature for 18 hours then quenched with water (100 mL). The resulting solution was extracted with DCM (3 x 150 mL) and the combined organic layers dried over Na_2SO_4 and the solvent removed *in vacuo*. The crude material was purified by flash column chromatography (30% Et_2O in petroleum ether 60:40) to afford sulfone **13** (351 mg, 90%) as a colourless oil. Data consistent with previously reported.

3.3 Synthesis of Aldehyde 10

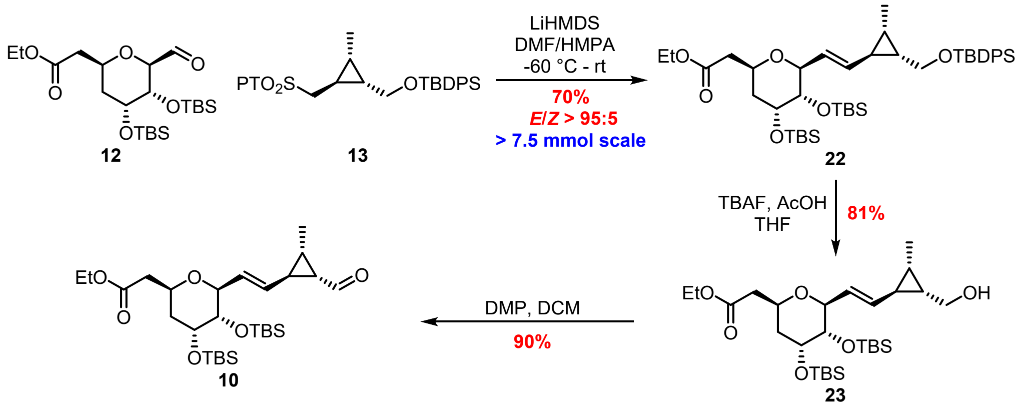

Ethyl 2-((2*S*,4*R*,5*S*,6*S*)-4,5-bis(*tert*-butyldimethylsiloxy)-6-((*E*)-2-((1*S*,2*S*,3*S*)-2-((*tert*-butyldiphenylsiloxy)methyl)-3-methylcyclopropyl)vinyl)tetrahydro-2H-pyran-2-yl)acetate (22)

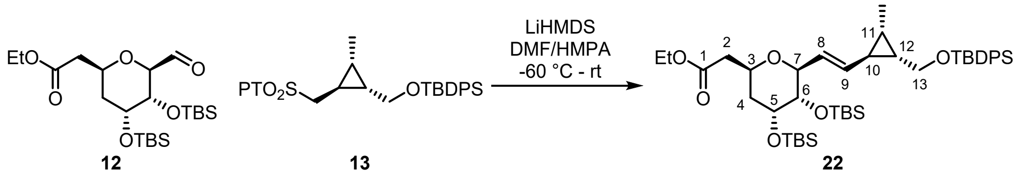

Sulfone **13** (5.28 g, 9.65 mmol) was dissolved in DMF (48 mL) and HMPA (12 mL) and cooled to -60 °C then 1 M LiHMDS in THF (9.65 mL, 9.65 mmol) was added dropwise over 15 minutes. The reaction mixture was stirred for 5 minutes then aldehyde **12** (3.60 g, 7.82 mmol) in DMF (16 mL) and HMPA (4 mL) was added dropwise. The orange solution was stirred at -60 °C for 3 hours then allowed to warm to room temperature and stirred for a further 2 hours. The reaction mixture was quenched with dropwise addition of water (25 mL) then was diluted with water (75 mL), brine (100 mL) and Et_2O (250 mL). The organic layer was separated, and the aqueous layer was extracted with Et_2O (2 x 250 mL). The combined organic layers were washed with brine (2 x 250 mL), dried over $MgSO_4$ and the solvent

removed *in vacuo*. The crude material was purified by flash column chromatography (8% Et_2O in petroleum ether 60:40) to afford alkene **22** (4.28 g, 70%, *E*/*Z* > 95:5) as a colourless oil; $[\alpha]_D^{21}$ = -20.0 (*c* 1, $CHCl_3$); ν_{max} (film) 2954, 2929, 2857, 1738, 1472, 1253, 1214; δ_H (400 MHz, $CDCl_3$) 0.00 (3H, s, $SiCH_3$), 0.02 (3H, s, $SiCH_3$), 0.06 (3H, s, $SiCH_3$), 0.07 (3H, s, $SiCH_3$), 0.87 (9H, s, $SiC(CH_3)_3$), 0.88 – 0.90 (1H, m, 10-H), 0.91 (9H, s, $SiC(CH_3)_3$), 0.92 – 0.93 (1H, m, 11-H), 1.04 (9H, s, $SiC(CH_3)_3$), 1.08 (3H, d, *J* 6.2, 11-CH_3), 1.17 (1H, m, 12-H), 1.23 (3H, t, *J* 7.1, OCH_2CH_3), 1.53 (1H, ddd, *J* 13.5, 11.6, 2.1, 4-H_{ax}), 1.82 (1H, ddd, *J* 13.5, 4.2, 2.1, 4-H_{eq}), 2.34 (1H, dd, *J* 14.8, 7.5, 2-*H*H), 2.61 (1H, dd, *J* 14.8, 6.1, 2-H*H*), 3.26 (1H, dd, *J* 9.1, 2.4, 6-H), 3.47 (1H, dd, *J* 11.0, 8.9, 13-*H*H), 3.88 (1H, dd, *J* 11.0, 5.4, 13-H*H*), 3.99 (1H, m, 5-H), 4.04 – 4.16 (3H, m, OCH_2CH_3 and 7-H), 4.24 (1H, m, 3-H), 5.22 (1H, dd, *J* 15.3, 8.6, 9-H), 5.33 (1H, dd, *J* 15.3, 7.3, 8-H), 7.34 – 7.45 (6H, m, 4 x ArH), 7.66 – 7.70 (4H, m, 4 x ArH); δ_C (101 MHz, $CDCl_3$) -4.4 ($SiCH_3$), -4.3 ($SiCH_3$), -4.2 ($SiCH_3$), -3.5 ($SiCH_3$), 12.7 (CH_3-11), 14.4 (OCH_2CH_3), 18.2 ($SiC(CH_3)_3$), 18.3 ($SiC(CH_3)_3$), 19.4 ($SiC(CH_3)_3$), 19.6 (C-11), 26.0 ($SiC(CH_3)_3$), 26.3 ($SiC(CH_3)_3$), 27.0 ($SiC(CH_3)_3$), 27.4 (C-12), 27.6 (C-10), 39.7 (C-4), 41.2 (C-2), 60.6 (OCH_2CH_3), 63.2 (C-13), 67.8 (C-3), 69.6 (C-5), 73.9 (C-6), 76.7 (C-7), 126.6 (C-8), 127.8 (2 x ArCH), 129.7 (2 x ArCH), 134.17 (2 x ArC), 135.8 (2 x ArCH), 137.1 (C-9), 171.2 (C-1); HRMS (ESI) calc. for $[C_{44}H_{72}O_6Si_3Na]$ 803.4529 Found 803.4524.

Ethyl 2-((2*S*,4*R*,5*S*,6*S*)-4,5-bis(*tert*-butyldimethylsiloxy)-6-((*E*)-2-((1*S*,2*S*,3*S*)-2-(hydroxymethyl)-3-methylcyclopropyl)vinyl)tetrahydro-2H-pyran-2-yl)acetate (23)

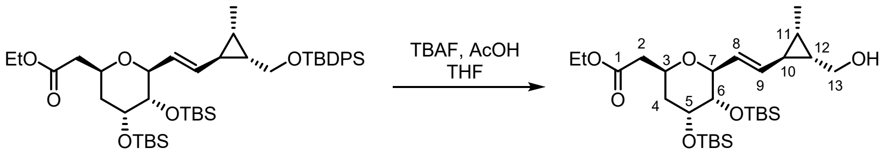

Silyl ether **22** (4.68 g, 6.00 mmol) was dissolved in THF (60 mL) under nitrogen and cooled to 0 °C then AcOH (0.343 mL, 6.00 mmol) and 1 M TBAF in THF (6.00 mL, 6.00 mmol) were added sequentially. The reaction mixture was stirred for 18 hours at room temperature then quenched with aqueous saturated $NaHCO_3$ (20 mL). The resulting solution was diluted with water (30 mL) and extracted with EtOAc (3 x 70 mL). The combined organic layers were washed with brine (100 mL), dried over $MgSO_4$ and the solvent removed *in vacuo*. The crude material was purified by flash column chromatography (50% Et_2O in petroleum ether 60:40) to afford alcohol **23** (2.63 mg, 81%) as a colourless oil; $[\alpha]_D^{21}$ = -38.0 (*c* 1, $CHCl_3$); ν_{max} (film) 3441, 2953, 2929, 2885, 2857, 1738, 1472, 1252, 1115; δ_H (400 MHz, $CDCl_3$) 0.03 (6H, s, 2 x $SiCH_3$), 0.05 (3H, s, $SiCH_3$), 0.07 (3H, s, $SiCH_3$), 0.88 (9H, s, $SiC(CH_3)_3$), 0.91 (9H, s, $SiC(CH_3)_3$), 0.95 – 1.02 (2H, m, 10-H and 11-H), 1.12 (3H, d, *J* 5.7, 11-CH_3), 1.15 – 1.20 (1H, m, 12-H), 1.23 (3H, t, *J* 7.1, OCH_2CH_3), 1.43 (1H, s, OH), 1.53 (1H, ddd, *J* 13.4, 11.4, 1.9, 4-H_{ax}), 1.82 (1H, ddd, *J* 13.5, 4.2, 2.1, 4-H_{eq}), 2.34 (1H, dd, *J* 14.8, 7.5, 2-*H*H), 2.60 (1H, dd, *J* 14.8, 6.0, 2-H*H*), 3.27 (1H, dd, *J* 9.1, 2.4, 6-H),

3.51 (1H, dd, *J* 11.4, 8.6, 13-*H*H), 3.77 (1H, dd, *J* 11.4, 6.3, 13-H*H*), 3.97 – 4.02 (1H, m, 5-H), 4.05 – 4.16 (3H, m, O$CH_2$$CH_3$ and 7-H), 4.20 – 4.30 (1H, m, 3-H), 5.24 (1H, dd, *J* 15.3, 8.3, 9-H), 5.39 (1H, dd, *J* 15.3, 7.4, 8-H); δ_C (101 MHz, $CDCl_3$) -4.4 ($SiCH_3$), -4.3 ($SiCH_3$), -4.2 ($SiCH_3$), -3.4 ($SiCH_3$), 12.6 (CH_3-11), 14.4 (OCH_2CH_3), 18.26 ($SiC(CH_3)_3$), 18.32 ($SiC(CH_3)_3$), 19.4 (C-11), 26.0 ($SiC(CH_3)_3$), 26.3 ($SiC(CH_3)_3$), 27.6 (C-12), 28.0 (C-10), 39.6 (C-4), 41.1 (C-2), 60.6 (OCH_2CH_3), 62.3 (C-13), 67.8 (C-3), 69.5 (C-5), 74.0 (C-6), 76.6 (C-7), 127.1 (C-8), 136.6 (C-9), 171.1 (C-1); HRMS (ESI) calc. for [$C_{28}H_{54}O_6Si_2Na$] 565.3351 Found 565.3350.

Ethyl 2-((2*S*,4*R*,5*S*,6*S*)-4,5-bis(*tert*-butyldimethylsiloxy)-6-((*E*)-2-((1S,2S,3S)-2-formyl-3-methylcyclopropyl)vinyl)tetrahydro-2H-pyran-2-yl)acetate (10)

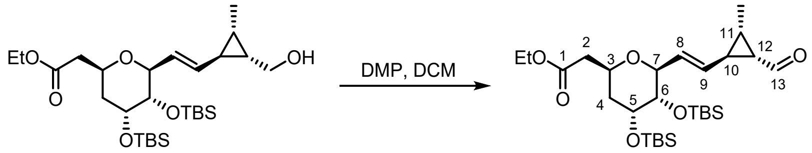

Alcohol **23** (2.60 g, 4.79 mmol) was dissolved in DCM (50 mL) under nitrogen and cooled to 0 °C, then DMP (2.64 g, 6.23 mmol) was added. The reaction mixture was stirred for 1 hour at room temperature then quenched with aqueous saturated $Na_2S_2O_3$ (30 mL) and aqueous saturated $NaHCO_3$ (30 mL) and stirred vigorously for 30 minutes. The aqueous phase was extracted with DCM (3 x 75 mL) and the combined organic layers were washed with brine (50 mL), dried over $MgSO_4$ and the solvent removed *in vacuo*. The crude material was purified by flash column chromatography (20% Et_2O in petroleum ether 60:40) to afford aldehyde **10** (2.33 g, 90%) as a white solid; $[\alpha]_D^{23}$ = -6.0 (*c* 1, $CHCl_3$); ν_{max} (film) 2954, 2020, 2887, 2857, 1738, 1700, 1472; δ_H (400 MHz, $CDCl_3$) 0.02 (3H, s, $SiCH_3$), 0.03 (3H, s, $SiCH_3$), 0.06 (3H, s, $SiCH_3$), 0.07 (3H, s, $SiCH_3$), 0.88 (9H, s, $SiC(CH_3)_3$), 0.91 (9H, s, $SiC(CH_3)_3$), 1.23 (3H, t, *J* 7.1, OCH_2CH_3), 1.27 (3H, d, *J* 6.4, 11-CH_3), 1.51 – 1.61 (2H, m, 4-H_{ax} and 11-H), 1.82 (1H, ddd, *J* 13.5, 4.1, 2.0, 4-H_{eq}), 1.96 (1H, ap. dt, *J* 9.1, 4.6, 12-H), 2.12 (1H, ddd, *J* 8.8, 6.4, 4.6, 10-H), 2.34 (1H, dd, *J* 14.9, 7.2, 2-*H*H), 2.58 (1H, dd, *J* 14.9, 6.4, 2-H*H*), 3.27 (1H, dd, *J* 9.1, 2.0, 6-H), 4.00 (1H, ap. dt, *J* 4.1, 2.0, 5-H), 4.07 – 4.17 (3H, m, O$CH_2$$CH_3$ and 7-H), 4.25 (1H, ap. dtd, *J* 11.4, 6.8, 2.0, 3-H), 5.25 (1H, ddd, *J* 15.3, 8.8, 1.1, 9-H), 5.58 (1H, dd, *J* 15.3, 7.0, 8-H), 9.47 (1H, d, *J* 4.6, 13-H); δ_C (101 MHz, $CDCl_3$) -4.4 ($SiCH_3$), -4.3 ($SiCH_3$), -4.2 ($SiCH_3$), -3.4 ($SiCH_3$), 12.7 (CH_3-11), 14.4 (OCH_2CH_3), 18.27 ($SiC(CH_3)_3$), 18.32 ($SiC(CH_3)_3$), 26.0 ($SiC(CH_3)_3$), 26.2 ($SiC(CH_3)_3$), 26.7 (C-11), 33.2 (C-10), 36.9 (C-12), 39.6 (C-4), 41.1 (C-2), 60.6 (OCH_2CH_3), 68.0 (C-3), 69.5 (C-5), 74.0 (C-6), 76.0 (C-7), 129.9 (C-8), 132.6 (C-9), 171.1 (C-1), 200.0 (C-13); HRMS (ESI) calc. for [$C_{28}H_{52}O_6Si_2$] 541.3375 Found 541.3369.

3.4 Synthesis of Ketone 32

3.4.1 Synthesis of Ketone 32 *via* Asymmetric Hydrogenation

1-((2*R*,6*R*)-6-Ethyl-5-methyl-3,6-dihydro-2H-pyran-2-yl)ethan-1-one (35)

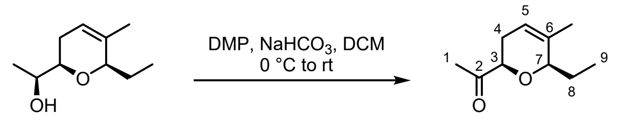

Alcohol **34**[4] (1.20 g, 7.05 mmol) was dissolved in DCM (70 mL) under nitrogen and cooled to 0 °C, then $NaHCO_3$ (2.37 g, 28.20 mmol) and DMP (3.59 g, 8.46 mmol) were added sequentially. The reaction mixture was stirred for 2 hours at room temperature, filtered over Celite eluting with Et_2O (100 mL), and the solvent removed *in vacuo*. The crude material was purified by flash column chromatography (7% Et_2O in pentane) to afford the volatile ketone **35** (1.15 g, 97%) as a colourless oil; $[\alpha]_D^{20}$ = +166 (*c* 0.25, $CHCl_3$) lit.[6] $[\alpha]_D^{20}$ = +181 (*c* 0.257, $CHCl_3$); ν_{max} (film) 2966, 2936, 1720, 1353, 1115, 1057; δ_H (400 MHz, $CDCl_3$) 0.94 (3H, t, *J* 7.3, 9-H_3), 1.44 – 1.58 (1H, m, 8-*H*H), 1.56 – 1.62 (3H, m, 6-CH_3), 1.81 (1H, dqd, *J* 14.8, 7.3, 3.5, 8-H*H*), 1.98 – 2.20 (2H, m, 4-H_2), 2.24 (3H, s, 1-H_3), 3.91 (1H, dd, *J* 10.5, 4.2, 3-H), 4.06 – 4.12 (1H, m, 7-H), 5.46 – 5.64 (1H, m, 5-H); δ_C (101 MHz, $CDCl_3$) 8.8 (C-9), 19.1 (CH_3-6), 25.8 (C-8), 25.9 (C-1), 27.5 (C-4), 78.5 (C-7), 78.9 (C-3), 119.7 (C-5), 135.8 (C-6), 210.2 (C-2); HRMS (ESI) calc. for $[C_{10}H_{16}O_2Na]$ 191.1043 Found 191.1045. Data consistent with the literature.[6]

1-((2*R*,5*S*,6*R*)-6-Ethyl-5-methyltetrahydro-2H-pyran-2-yl)ethan-1-one (32)

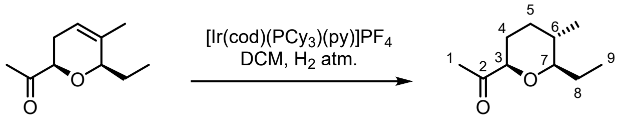

Ketone **35** (73 mg, 0.45 mmol) was dissolved in degassed DCM (2 mL) under nitrogen at room temperature then Crabtree's catalyst (17 mg, 0.022 mmol) was added. Using a balloon, H_2 was bubbled through the reaction mixture for 5 minutes then the reaction mixture was stirred under H_2 at atmospheric pressure for 3 hours. The solvent was removed *in vacuo* and the resulting material dissolved in Et_2O (5 mL), filtered over celite and the solvent removed *in vacuo*. The crude material was purified by flash column chromatography (8% Et_2O in pentane) to afford ketone **32** (65 mg, 89%) as a single diastereoisomer and a colourless oil; $[\alpha]_D^{22}$ = +172 (*c* 1, $CHCl_3$); ν_{max} (film) 2961, 2930, 2877, 1721, 1215, 1103, 746; δ_H (400 MHz, $CDCl_3$) 0.82 (3H, d, *J* 6.5, 6-CH_3), 0.97 (3H, t, *J* 7.5, 9-H_3), 1.17 – 1.27 (1H, m, 5-*H*H), 1.30 – 1.49 (3H, m, 4-*H*H, 6-H and 8-*H*H), 1.73 (1H, dqd, *J* 14.0, 7.5, 2.8, 8-H*H*), 1.79 – 1.89 (2H, m, 4-H*H* and 5-H*H*), 2.20 (3H, s, 1-H_3), 2.89 (1H, ddd, *J* 9.5, 8.4, 2.8, 7-H), 3.70 (1H, dd, *J* 11.6, 2.5,

3-H); δ_C (101 MHz, $CDCl_3$) 9.9 (C-9), 17.8 (CH_3-6), 26.02 (C-8), 26.04 (C-1), 28.5 (C-4), 32.7 (C-5), 34.5 (C-6), 83.2 (C-3), 84.8 (C-7), 210.6 (C-2); HRMS (ESI) calc. for $[C_{10}H_{18}O_2Na]$ 193.1199 Found 193.1199. Data consistent with the literature.[7]

Scale-up procedure:

Ketone **35** (1100 mg, 6.45 mmol) was dissolved in degassed DCM (26 mL) under nitrogen at room temperature. Using a balloon, H_2 was bubbled through the solution was 1 minute then Crabtree's catalyst (264 mg, 0.33 mmol) was added. Using a balloon, H_2 was bubbled through the reaction mixture for 20 minutes then the reaction mixture was stirred under H_2 at atmospheric pressure for 3 hours. The solvent was removed *in vacuo* and the resulting material dissolved in Et_2O (25 mL), filtered over Celite and the solvent removed *in vacuo*. The crude material was purified by flash column chromatography (8% Et_2O in pentane) to afford ketone **32** (889 mg, 81%) as a single diastereoisomer and a colourless oil. Data consistent with previously reported.

3.4.2 Synthesis of Ketone 32 *via* Prins Cyclisation

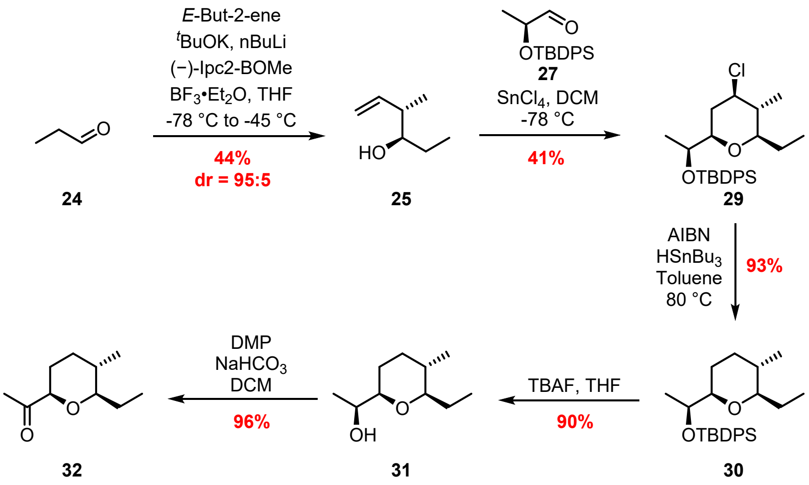

(3*R*,4*S*)-4-Methylhex-5-en-3-ol (25)

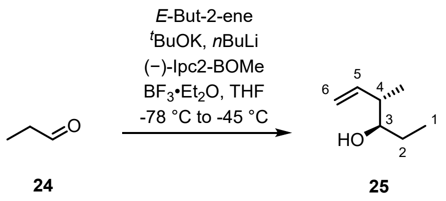

*t*BuOK (561 mg, 5.0 mmol) was dissolved in THF (15 mL) under nitrogen and cooled to -78 °C then condensed *trans*-but-2-ene (0.90 mL, 10.0 mmol) was added *via* cannula. A solution of 2.5 M *n*BuLi in hexane (2.00 mL, 5.0 mmol) was added dropwise over 15 minutes and the reaction mixture was stirred for 10 minutes at -45 °C then cooled to -78 °C. A 0.92 M solution of (−)-Ipc_2BOMe in THF (6.52 mL, 6.0

mmol) was added dropwise over 20 minutes. The reaction mixture was stirred at -78 °C for 30 minutes then $BF_3.Et_2O$ (0.83 mL, 6.7 mmol) was added dropwise over 5 minutes followed by propionaldehyde **24** (0.51 mL, 7.0 mmol) over 5 minutes. The reaction mixture was stirred for 4 hours, quenched with 3 M NaOH (4 mL) and 30% H_2O_2 (2 mL), then stirred at room temperature for 16 hours. The resulting solution was diluted with Et_2O (150 mL) and water (100 mL). The organic layer was separated and the aqueous extracted with further Et_2O (2 x 100 mL). The combined organic layers were washed with brine (100 mL), dried over $MgSO_4$ and the solvent removed *in vacuo*. The crude material was purified by flash column chromatography (10% Et_2O in pentane) to afford alcohol **25** (252 mg, 44%, 95:5 dr) as a colourless oil. ^{1}H NMR analysis of the Mosher's ester indicated a >95% ee; $[\alpha]_D^{21}$ -8.0 (*c* 1, $CHCl_3$); lit.[8] $[\alpha]_D^{25}$ = -8.7 (*c* 0.3, $CHCl_3$); ν_{max} (film) 3385, 3077, 2964, 2935, 2877, 1639, 1457, 1215; δ_H (400 MHz, $CDCl_3$) 0.96 (3H, t, *J* 7.4, 1-H_3), 1.03 (3H, d, *J* 6.8, 4-CH_3), 1.35 – 1.44 (1H, m, 2-*H*H), 1.52 – 1.64 (2H, m, OH and 2-H*H*), 2.14 – 2.29 (1H, m, 4-H), 3.22 – 3.40 (1H, m, 3-H), 5.09 (1H, m, 6-*H*H), 5.12 (1H, m, 6-H*H*), 5.69 – 5.84 (1H, m, 5-H); δ_C (101 MHz, $CDCl_3$) 10.1 (C-1), 16.5 (CH_3-4), 27.1 (C-2), 43.9 (C-4), 76.2 (C-3), 116.4 (C-6), 140.6 (C-5); HRMS (APCI) calc. for [$C_7H_{14}O$] 97.1012 Found 97.1008. Data consistent with the literature.[8]

Ethyl (*S*)-2-(*tert*-butyldiphenylsiloxy)propanoate (S6)

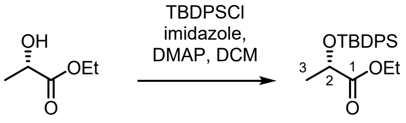

S5 S6

To a solution of alcohol **S5** (2.36 g, 20.00 mmol) in DCM (70 mL) under nitrogen was added imidazole (1.63 g, 24 mmol), TBDPSCl (5.72 mL, 24 mmol) and a spatula tip of DMAP and the reaction mixture was stirred for 24 hours. The reaction was quenched with 2 M HCl (20 mL) and water (50 mL). The organic layer was separated and the aqueous extracted with DCM (2 × 60 mL). The combined organic layers were washed with brine (100 mL), dried over $MgSO_4$ and the solvent removed *in vacuo* to afford silyl alcohol **S6** (7.42 g, quant.) as a colourless oil; $[\alpha]_D^{23}$ = -69.0 (*c* 1, $CHCl_3$); ν_{max} (film) 3072, 2933, 2894, 2858, 1752, 1733, 1428, 1133, 1106; δ_H (400 MHz, $CDCl_3$) 1.10 (9H, s, SiC(CH_3)$_3$), 1.15 (3H, t, *J* 7.0, OCH_2*CH_3*), 1.37 (3H, d, *J* 6.8, 3-H_3), 4.02 (2H, q, *J* 7.0, O*CH_2*CH_3), 4.27 (1H, q, *J* 6.8, 2-H), 7.31 – 7.48 (6H, m, ArH), 7.62 – 7.73 (4H, m, ArH); δ_C (101 MHz, $CDCl_3$) 14.2 (OCH_2*C*H_3), 19.4 (Si*C*(CH_3)$_3$), 21.4 (C-3), 27.0 (SiC(*C*H_3)$_3$), 66.0 (O*C*H_2CH_3), 69.1 (C-2), 127.7 (ArCH), 127.8 (ArCH), 129.9 (ArCH), 133.4 (ArC), 133.8 (ArC), 135.9 (ArCH), 136.1 (ArCH), 173.9 (C-1); HRMS (ESI) calc. for [$C_{21}H_{28}NaSiO_3$] 379.1700 Found 379.1689. Data consistent with the literature.[4]

(*S*)-2-(*tert*-Butyldiphenylsiloxy)propanal (27)

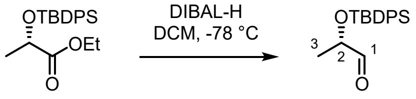

Ester **S6** (2.00 g, 5.60 mmol) was dissolved in DCM (20 mL) under nitrogen and cooled to -78 °C then 1 M DIBAL-H in hexanes (6.20 mL, 6.20 mmol) was added dropwise. The reaction mixture was stirred at -78 °C for 1 hour, then quenched with MeOH (1 mL) dropwise followed by aqueous saturated sodium potassium tartrate solution (50 mL). The reaction mixture was warmed to room temperature and stirred vigorously for 1 hour. The organic layer was separated and the aqueous extracted with DCM (2 × 50 mL). The combined organic layers were washed with brine (100 mL), dried over $MgSO_4$ and the solvent removed *in vacuo*. The crude material was purified by flash column chromatography (3% Et_2O in petroleum ether 60:40) to afford aldehyde **27** (1.45 g, 83%) as a colourless oil; $[\alpha]_D^{23}$ = -26.0 (*c* 1, $CHCl_3$); ν_{max} (film) 3072, 2959, 2932, 2858, 1738, 1428, 1110, 699; δ_H (400 MHz, $CDCl_3$) 1.11 (9H, s, $SiC(CH_3)_3$), 1.22 (3H, d, *J* 6.8, 3-H_3), 4.09 (1H, qd, *J* 6.8, 1.2, 2-H), 7.34 – 7.47 (6H, m, ArH), 7.62 – 7.68 (4H, m, ArH), 9.64 (1H, d, *J* 1.2, C-1); δ_C (101 MHz, $CDCl_3$) 18.6 (C-3), 19.4 (Si*C*($CH_3)_3$), 27.0 (SiC(*C*$H_3)_3$), 74.6 (C-2), 127.95 (ArCH), 128.03 (ArCH), 130.16 (ArCH), 130.23 (ArCH), 133.1 (ArC), 133.5 (ArC), 135.89 (ArCH), 135.92 (ArCH), 204.0 (C-1); HRMS (ESI) calc. for [$C_{19}H_{24}NaO_2Si$] 335.1438 Found 335.1436. Data consistent with the literature.[4]

Ethyl (*S*)-2-((*tert*-butyldimethylsilyl)oxy)propanoate (S5)

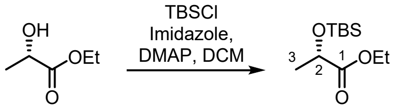

To a solution of alcohol **S5** (5.91 g, 50.00 mmol) in DCM (150 mL) was added imidazole (4.08 g, 68.08 mmol), TBSCl (8.29 g, 55 mmol) and a spatula tip of DMAP and the reaction mixture was stirred for 24 hours. The reaction was quenched with 2 M HCl (20 mL) and water (50 mL). The organic layer was separated and the aqueous extracted with DCM (2 × 60 mL). The combined organic layers were washed with brine (100 mL), dried over $MgSO_4$ and the solvent removed *in vacuo* to afford silyl alcohol **S7** (11.64 g, quant.) as a colourless oil; $[\alpha]_D^{24}$ = -40.0 (*c* 1, $CHCl_3$); ν_{max} (film) 2983, 2955, 2931, 2888, 2858, 1754, 1736, 1252, 1142, 829; δ_H (400 MHz, $CDCl_3$) 0.06 (3H, s, $Si(CH_3)$), 0.09 (3H, s, $Si(CH_3)$), 0.89 (9H, s, $SiC(CH_3)_3$), 1.27 (3H, t, *J* 7.1, OCH_2*CH_3*), 1.38 (3H, d, *J* 6.7, 3-H_3), 4.12 – 4.22 (2H, m, O*CH_2*CH_3), 4.30 (1H, q, *J* 6.7, 2-H); δ_C (101 MHz, $CDCl_3$) -5.1 ($SiCH_3$), -4.8 ($SiCH_3$), 14.4 (OCH_2*C*H_3), 18.5 (Si*C*($CH_3)_3$), 21.5 (C-3), 25.9 (SiC(*C*$H_3)_3$), 60.9 (O*C*H_2CH_3), 68.6 (C-2), 174.3 (C-1); HRMS (ESI) calc. for [$C_{11}H_{24}NaSiO_3$] 255.1387 Found 255.1396. Data consistent with the literature.[9]

(*S*)-2-(*tert*-Butyldimethylsilyloxy)propanal (26)

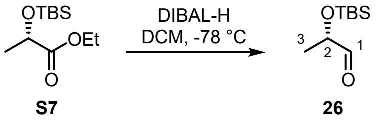

Ester **S7** (1.00 g, 4.30 mmol) was dissolved in DCM (15 mL) under nitrogen and cooled to -78 °C then 1 M DIBAL-H in hexanes (4.73 mL, 4.73 mmol) was added dropwise. The reaction mixture was stirred at -78 °C for 1 hour, then quenched with dropwise addition of MeOH (1 mL) followed by aqueous saturated sodium potassium tartrate solution (50 mL). The reaction mixture was warmed to room temperature and stirred vigorously for 1 hour. The organic layer was separated and the aqueous extracted with DCM (2 × 50 mL). The combined organic layers were washed with brine (100 mL), dried over $MgSO_4$ and the solvent removed *in vacuo*. The crude material was purified by flash column chromatography (5% Et_2O in petroleum ether 60:40) to afford aldehyde **26** (673 mg, 83%) as a colourless oil; $[\alpha]_D^{25}$ = -19.0 (*c* 1, $CHCl_3$); ν_{max} (film) 2955, 2930, 2857, 2886, 1739, 1253, 832, 776; δ_H (400 MHz, $CDCl_3$) 0.09 (3H, s, $SiCH_3$), 0.10 (3H, s, $SiCH_3$), 0.92 (9H, s, $SiC(CH_3)_3$), 1.28 (3H, d, *J* 6.8, 3-H_3), 4.09 (1H, qd, *J* 6.8, 1.3, 2-H), 9.61 (1H, d, *J* 1.3, 1-H); δ_C (101 MHz, $CDCl_3$) -4.63 ($SiCH_3$), -4.57 ($SiCH_3$), 18.4 ($Si\mathit{C}(CH_3)_3$), 18.7 (C-3), 25.9 ($SiC(\mathit{C}H_3)_3$), 74.0 (C-2), 204.4 (C-1); HRMS (ESI) calc. for [$C_9H_{20}O_2Si$] 189.1305 Found 189.1304. Data consistent with the literature.[9]

***tert*-Butyl((*S*)-1-((2*R*,4*R*,5*R*,6*R*)-4-chloro-6-ethyl-5-methyltetrahydro-2H-pyran-2-yl)ethoxy)diphenylsilane (29)**

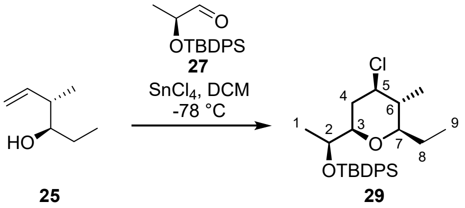

Homoallylic alcohol **25** (387 mg, 3.39 mmol) and aldehyde **27** (1060 mg, 3.39 mmol) were dissolved in DCM (40 ml) at -78 °C under nitrogen and $SnCl_4$ (1 M in DCM, 6.78 mL, 6.78 mmol) was added dropwise. The reaction mixture was stirred for 3 hours then quenched with aqueous saturated sodium hydrogen carbonate (20 mL) and warmed to room temperature. The resultant solution was diluted with DCM (50 mL) and water (30 mL), and the organic layer separated. The aqueous layer was extracted with further DCM (2 x 40 mL) and the combined organic layers were washed with brine (50 mL), dried over $MgSO_4$ and the solvent removed *in vacuo*. The crude material was purified by flash column chromatography (12% DCM in petroleum ether 60:40) to afford tetrahydropyran **29** (614 mg, 41%) as a white crystalline solid; $[\alpha]_D^{23}$ = -12.0 (*c* 1, $CHCl_3$); ν_{max} (film) 2965, 2931, 2857, 1473, 1428, 1376, 1111; δ_H (400 MHz, $CDCl_3$) 0.94 (3H, t, *J* 7.3, 9-H_3), 1.00 – 1.05 (6H, m 1-H_3 and, 6-CH_3), 1.06 (9H,

s, $SiC(CH_3)_3$), 1.33 – 1.46 (1H, m, 8-*H*H), 1.47 – 1.60 (1H, m, 6-H), 1.70 – 1.76 (1H, m, 8-H*H*), 1.82 (1H, ap. q, *J* 12.0, 4-H_{ax}), 2.33 (1H, ddd, *J* 12.8, 4.6, 2.0, 4-H_{eq}), 2.88 (1H, ap. td, *J* 9.1, 2.5, 7-H), 3.15 (1H, ddd, *J* 11.4, 4.5, 2.0, 3-H), 3.69 (1H, ap. td, *J* 11.3, 4.6, 5-H), 3.86 (1H, qd, *J* 6.3, 4.5, 2-H), 7.32 – 7.47 (6H, m, 6 x ArH), 7.68 – 7.75 (4H, m, 4 x ArH); δ_C (101 MHz, $CDCl_3$) 10.1 (C-9), 14.7 (CH_3-6), 19.6 (Si*C*$(CH_3)_3$), 20.2 (C-1), 26.4 (C-8), 27.2 (SiC(*C*$H_3)_3$), 38.3 (C-4), 44.7 (C-6), 65.3 (C-5), 71.8 (C-2), 81.0 (C-3), 83.4 (C-7), 127.66 (ArCH), 127.70 (ArCH), 129.7 (ArCH), 129.80 (ArCH), 134.0 (ArC), 135.0 (ArC), 136.2 (ArCH), 136.45 (ArCH); HRMS (EI) calc. for [$C_{26}H_{37}O_2Si^{35}Cl$] 387.1542 Found 387.1546.

tert-Butyl((*S*)-1-((2*R*,5*S*,6*R*)-6-ethyl-5-methyltetrahydro-2H-pyran-2-yl)ethoxy)diphenylsilane (30)

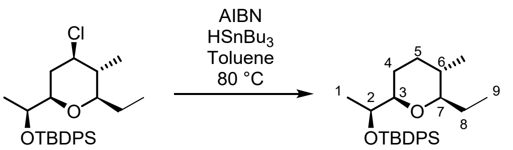

29 **30**

Chloride **29** (430 mg, 0.97 mmol) was dissolved in degassed toluene (10 mL) under nitrogen and heated to 50 °C then tributyltin hydride (0.78 mL, 2.90 mmol) was added followed by AIBN (32 mg). The reaction mixture was heated at 80 °C for 1 hour, cooled to room temperature and diluted with aqueous saturated potassium fluoride (25 mL) and EtOAc (50 mL). The organic layer was separated and washed with aqueous saturated potassium fluoride (25 mL), then brine (25 mL), dried over $MgSO_4$ and the solvent removed *in vacuo*. The crude material was purified by flash column chromatography (13% DCM in petroleum ether 60:40) to afford tetrahydropyran **30** (372 mg, 93%) as a colourless oil; $[\alpha]_D^{22}$ = +12.0 (*c* 1, $CHCl_3$); ν_{max} (film) 2960, 2930, 2856, 1463, 1427, 1105; δ_H (400 MHz, $CDCl_3$) 0.81 (3H, d, *J* 6.5, 6-CH_3), 0.94 (3H, ap. t, *J* 7.4, 9-H_3), 1.04 (3H, d, *J* 6.2, 1-H_3), 1.06 (9H, s, $SiC(CH_3)_3$), 1.10 – 1.22 (1H, m, 5-*H*H), 1.24 – 1.45 (3H, m, 4-*H*H, 6-H and 8-*H*H), 1.63 – 1.75 (1H, m, 8-H*H*), 1.76 – 1.86 (2H, m, 4-H*H* and 5-H*H*), 2.79 (1H, ap. td, *J* 9.2, 2.6, 7-H), 3.09 (1H, ddd, *J* 11.1, 5.4, 2.0, 3-H), 3.75 – 3.88 (1H, m, 2-H), 7.31 – 7.47 (6H, m, ArH), 7.62 – 7.81 (4H, m, ArH); δ_C (101 MHz, $CDCl_3$) 10.2 (C-9), 18.0 (CH_3-6), 19.6 (Si*C*$(CH_3)_3$), 20.4 (C-1), 26.3 (C-8), 27.2 (SiC(*C*$H_3)_3$), 27.6 (C-4), 33.1 (C-5), 35.4 (C-6), 72.6 (C-2), 82.4 (C-3), 85.0 (C-7), 127.5 (ArCH), 127.6 (ArCH), 129.5 (ArCH), 129.6 (ArCH), 134.4 (ArC), 135.3 (ArC), 136.2 (ArCH), 136.3 (ArCH); HRMS (EI) calc. for [$C_{26}H_{38}O_2Si$] 353.1931 Found 353.1929.

(*S*)-1-((2*R*,5*S*,6*R*)-6-Ethyl-5-methyltetrahydro-2H-pyran-2-yl)ethan-1-ol (31)

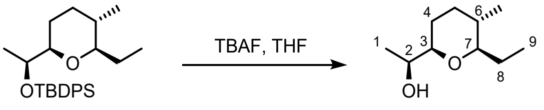

30 **31**

Silyl ether **30** (350 mg, 0.79 mmol) was cooled to 0 °C under nitrogen then 1 M TBAF in THF (1.57 mL, 1.57 mmol) was added dropwise. The reaction mixture was stirred under nitrogen at room temperature for 24 hours and then quenched with water (20 mL) and Et_2O (50 mL). The organic layer was separated and the aqueous extracted with Et_2O (2 x 50 mL). The combined organic layers were washed with brine (50 mL), dried over $MgSO_4$ and the solvent removed *in vacuo*. The crude material was purified by flash column chromatography (30% Et_2O in petroleum ether 60:40) to afford alcohol **31** (122 mg, 90%) as a colourless oil; $[\alpha]_D^{22}$ = +36.0 (*c* 1, $CHCl_3$); ν_{max} (film) 3398, 2929, 2875, 1457, 1379, 1214, 747; δ_H (500 MHz, $CDCl_3$) 0.80 (3H, d, *J* 6.6, 6-CH_3), 0.92 (3H, ap. t, *J* 7.5, 9-H_3), 1.13 (3H, d, *J* 6.5, 1-H_3), 1.14 – 1.19 (1H, m, 5-*H*H), 1.26 – 1.33 (1H, m, 6-H), 1.33 – 1.43 (2H, m, 4-*H*H and 8-*H*H), 1.55 (1H, ap. ddt, *J* 13.1, 5.1, 2.5, 4-H*H*), 1.68 (1H, dqd, *J* 14.9, 7.5, 2.7, 8-H*H*), 1.79 (1H, ap. dq, *J* 13.2, 3.6, 5-H*H*), 2.20 – 2.28 (1H, m, OH), 2.87 (1H, ddd, *J* 9.4, 8.2, 2.7, 7-H), 3.19 (1H, ddd, *J* 11.4, 4.0, 2.5, 3-H), 3.73 – 3.83 (m, 1H); δ_C (101 MHz, $CDCl_3$) 9.9 (C-9), 17.9 (CH_3-6), 18.0 (C-1), 25.6 (C-4), 26.1 (C-8), 32.7 (C-5), 35.0 (C-6), 69.8 (C-2), 80.7 (C-3), 84.7 (C-7); HRMS (ESI) calc. for [$C_{10}H_{20}O_2Na$] 195.1356 Found 195.1363.

1-((2*R*,5*S*,6*R*)-6-Ethyl-5-methyltetrahydro-2H-pyran-2-yl)ethan-1-one (32)

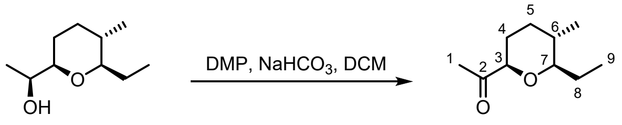

Alcohol **31** (88 mg, 0.51 mmol) was dissolved in DCM (5 mL) under nitrogen and cooled to 0 °C, then $NaHCO_3$ (171 mg, 2.04 mmol) and DMP (260 mg, 0.61 mmol) were added sequentially. The reaction mixture was stirred for 2 hours at room temperature, filtered over celite eluting with Et_2O (100 mL) and the solvent removed *in vacuo*. The crude material was purified by flash column chromatography (8% Et_2O in pentane) to afford ketone **32** (83 mg, 96%) as a colourless oil. Data consistent with previously reported.

3.5 Synthesis of Sulfone 11

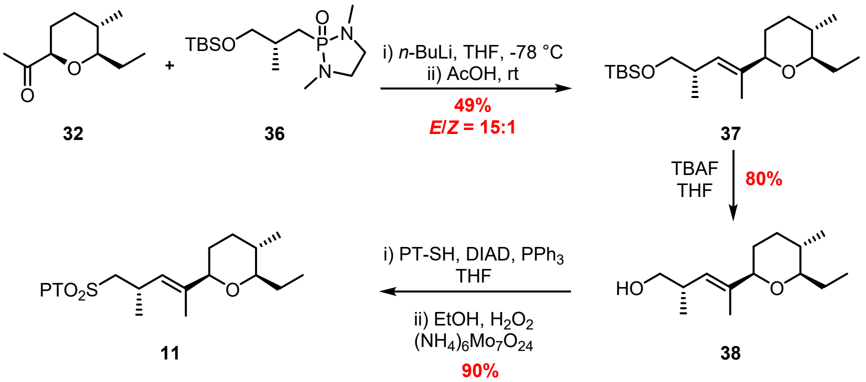

***tert*-Butyl(((*S*,*E*)-4-((2*R*,5*S*,6*R*)-6-ethyl-5-methyltetrahydro-2H-pyran-2-yl)-2-methylpent-3-en-1-yl)oxy)dimethylsilane (37)**

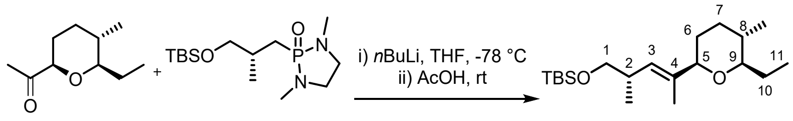

Phosphonamide **36**[10] (3.15 g, 9.82 mmol) was dissolved in THF (30 mL) under nitrogen and cooled to -78 °C then 2.5 M *n*-BuLi in hexane (3.39 mL, 8.47 mmol) was added dropwise and the reaction mixture was stirred for 2 hours. Neat ketone **32** (0.76 g, 4.46 mmol) was added dropwise and the reaction mixture was stirred at -78 °C for 1 hour. The reaction mixture was warmed to room temperature and quenched with AcOH (2.55 mL, 44.6 mmol) and stirred for an additional 20 minutes at room temperature. Aqueous saturated $NaHCO_3$ (50 mL) was added and the resulting solution was extracted with DCM (3 x 100 mL). The combined organic layers were dried over $MgSO_4$ and the solvent removed *in vacuo*. The crude material was purified by flash column chromatography (3% Et_2O in petroleum ether 60:40) to afford alkene **37** (0.75 g, 49%, *E*/*Z* = 15:1) as a colourless oil and ketone **32** (212 mg, 28%); $[\alpha]_D^{24}$ = +22 (*c* 1, $CHCl_3$); ν_{max} (film) 2956, 2929, 2856, 1215, 753; δ_H (400 MHz, $CDCl_3$) 0.81 (3H, d, *J* 6.6, 8-CH_3), 0.88 (9H, s, $SiC(CH_3)_3$), 0.95 (3H, d, *J* 1.1, 2-CH_3), 0.95 – 0.98 (3H, m, 11-H_3), 1.17 – 1.25 (1H, m, 7-*H*H), 1.30 – 1.38 (1H, m, 8-H), 1.38 – 1.48 (2H, m, 6-*H*H and 10-*H*H), 1.56 – 1.72 (5H, m, 4-CH_3, 6-H*H* and 10-H*H*), 1.74 – 1.82 (1H, m, 7-H*H*), 2.51 – 2.60 (1H, m, 2-H), 2.90 (1H, ddd, *J* 9.5, 7.5, 3.0, 9-H), 3.26 – 3.36 (1H, m, 1-*H*H), 3.42 – 3.52 (1H, m, 1-H*H*), 3.54 – 3.62 (1H, m, 5-H), 5.14 – 5.18 (1H, m, 3-H); δ_C (101 MHz, $CDCl_3$) -6.16 ($SiCH_3$), -6.09 ($SiCH_3$), 8.6 (C-11), 12.6 (CH_3-4), 16.5 (CH_3-2), 17.0 (CH_3-8), 17.5 ($Si\mathit{C}(CH_3)_3$), 25.08 (C-10), 25.14 ($SiC(\mathit{C}H_3)_3$), 30.0 (C-6), 32.4 (C-7), 33.4 (C-8), 34.2 (C-2), 67.1 (C-1), 81.2 (C-5), 83.5 (C-9), 126.3 (C-3), 136.1 (C-4); HRMS (ESI) calc. for $[C_{20}H_{40}O_2SiNa]$ 363.2701 Found 363.2690.

(*S*,*E*)-4-((2*R*,5*S*,6*R*)-6-Ethyl-5-methyltetrahydro-2H-pyran-2-yl)-2-methylpent-3-en-1-ol (38)

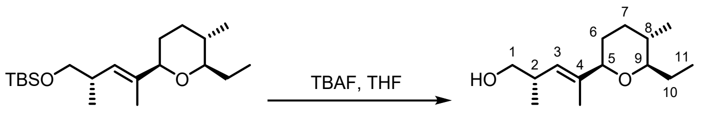

Silyl ether **37** (737 mg, 2.16 mmol, *E*/*Z* = 15:1) was dissolved in THF (20 mL) under nitrogen and cooled to 0 °C then 1 M TBAF in THF (3.25 mL, 3.25 mmol) was added dropwise. The reaction mixture was stirred under nitrogen at room temperature for 3 hours and then quenched with aqueous saturated NH_4Cl (30 mL) and the organic solvent removed *in vacuo*. The aqueous layer was extracted with EtOAc (3 x 50 mL) and the combined organic layers were dried over Na_2SO_4 and the solvent removed *in vacuo*. The crude material was purified by flash column chromatography (35% Et_2O in petroleum ether 60:40) to afford alcohol **38** (391 mg, 80%) as a colourless oil; $[\alpha]_D^{23}$ = +20.0 (*c* 1, $CHCl_3$); ν_{max} (film) 3403, 2956, 2928, 2873, 2850, 1457, 1380, 1084, 1031; δ_H (500 MHz, $CDCl_3$) 0.81 (3H, d, *J* 6.5, 8-CH_3), 0.92 – 0.98 (6H, m, 2-CH_3 and 11-H_3), 1.15 – 1.26 (1H, m, 7-*H*H), 1.30 – 1.46 (3H, m, 6-*H*H, 8-H and 10-*H*H), 1.52 (1H, s, OH), 1.62 – 1.74 (5H, m, 4-CH_3, 6-H*H* and 10-H*H*), 1.80 (1H, ap. dq, *J* 13.0, 3.6, 7-H*H*), 2.60 – 2.70 (1H, m, 2-H), 2.90 (1H, ddd, *J* 9.5, 7.6, 3.0, 9-H), 3.36 (1H, dd, *J* 10.5, 7.8, 1-*H*H), 3.43 – 3.49 (1H, m, 1-H*H*), 3.61 (1H, m, 5-H), 5.13 – 5.20 (1H, m, 3-H); δ_C (126 MHz, $CDCl_3$) 9.7 (C-11), 13.9 (CH_3-4), 17.1 (CH_3-2), 17.9 (CH_3-8), 26.1 (C-10), 31.1 (C-6), 33.4 (C-7), 34.5 (C-8), 35.2 (C-2), 68.0 (C-1), 81.9 (C-5), 84.6 (C-9), 126.6 (C-3), 139.3 (C-4); HRMS (ESI) calc. for [$C_{14}H_{26}O_2Na$] 249.1825 Found 249.1820.

5-(((*S*,*E*)-4-((2*R*,5*S*,6*R*)-6-Ethyl-5-methyltetrahydro-2H-pyran-2-yl)-2-methylpent-3-en-1-yl)sulfonyl)-1-phenyl-1H-tetrazole (11)

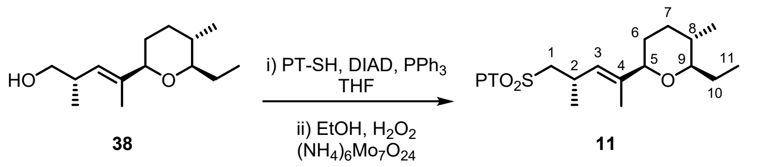

Alcohol **38** (340 mg, 1.50 mmol), PT-SH (348 mg, 1.96 mmol) and PPh_3 (514 mg, 1.96 mmol) were dissolved in THF (15 mL) under nitrogen and cooled the 0 °C then DIAD (0.39 mL, 1.96 mmol) was added dropwise. The reaction mixture was stirred for 3 hours at room temperature then quenched with aqueous saturated $NHCl_4$ (20 mL) and brine (20 mL). The resulting solution was extracted with EtOAc (3 x 75 mL) and the combined organic layers were washed with brine (50 mL), dried over Na_2SO_4 and the solvent removed *in vacuo*. The crude material was filtered over a small plug of silica eluting with 20% Et_2O in petroleum ether 60:40 and the solvent removed *in vacuo* to afford sulfide (552 mg, 95%) as a yellow oil.

The sulfide was dissolved in EtOH (47 mL) and cooled to 0 °C then a premixed solution of molybdenate (347 mg, 0.28 mmol) in a 30% aqueous solution of H_2O_2 (1.43 mL, 14.1 mmol) was added dropwise.

The reaction mixture was stirred at room temperature for 18 hours then quenched with water (50 mL). The resulting solution was extracted with DCM (3 x 100 mL) and the combined organic layers dried over $MgSO_4$ and the solvent removed *in vacuo*. The crude material was purified by flash column chromatography (10% EtOAc in petroleum ether 60:40) to afford sulfone **11** (542 mg, 90% over two steps) as a yellow oil; $[\alpha]_D^{24}$ = -16.0 (*c* 1, $CHCl_3$); ν_{max} (film) 3019, 2927, 1498, 1459, 1347, 1214; δ_H (400 MHz, $CDCl_3$) 0.80 (3H, d, *J* 6.6, 8-CH_3), 0.95 (3H, t, *J* 7.4, 11-H_3), 1.16 (3H, d, *J* 6.8, 2-CH_3), 1.18 – 1.20 (1H, m, 7-*H*H), 1.20 – 1.23 (1H, m, 6-*H*H), 1.28 – 1.35 (1H, m, 8-H), 1.42 (ap. dp, *J* 14.5, 7.4, 10-*H*H), 1.52 – 1.57 (1H, m, 6-H*H*), 1.61 (3H, d, *J* 1.4, 4-CH_3), 1.70 (1H, dqd, *J* 14.5, 7.4, 2.9, 10-H*H*), 1.74 – 1.80 (1H, m, 7-H*H*), 2.87 (1H, ddd, *J* 9.9, 7.4, 2.9, 9-H), 3.27 (1H, m, 2-H), 3.48 (1H, d, *J* 9.7, 5-H), 3.57 (1H, dd, *J* 14.7, 6.3, 1-*H*H), 3.83 (1H, dd, *J* 14.7, 7.5, 1-H*H*), 5.13 (1H, dt, *J* 9.7, 1.4, 3-H), 7.54 – 7.68 (5H, m, ArH); δ_C (101 MHz, $CDCl_3$) 9.7 (C-11), 13.7 (CH_3-4), 17.9 (CH_3-8), 21.0 (CH_3-2), 26.0 (C-10), 27.9 (C-2), 30.6 (C-6), 33.2 (C-7), 34.3 (C-8), 61.9 (C-1), 81.1 (C-5), 84.7 (C-9), 125.1 (C-3), 125.7 (2 x ArCH), 129.7 (2 x ArCH), 131.6 (ArCH), 133.3 (ArC), 138.9 (C-4), 154.2 (NCN); HRMS (ESI) calc. for $[C_{21}H_{30}N_4O_3S]$ 419.2111 Found 419.2113.

3.6 Synthesis of 20,21-Dihydroambruticin F

Ethyl 2-((2*S*,4*R*,5*S*,6*S*)-4,5-bis(*tert*-butyldimethylsiloxy)-6-((*E*)-2-((1*S*,2*S*,3*R*)-2-((*R*,1*E*,4*E*)-5-((2*R*,5*S*,6*R*)-6-ethyl-5-methyltetrahydro-2H-pyran-2-yl)-3-methylhexa-1,4-dien-1-yl)-3-methylcyclopropyl)vinyl)tetrahydro-2H-pyran-2-yl)acetate (39)

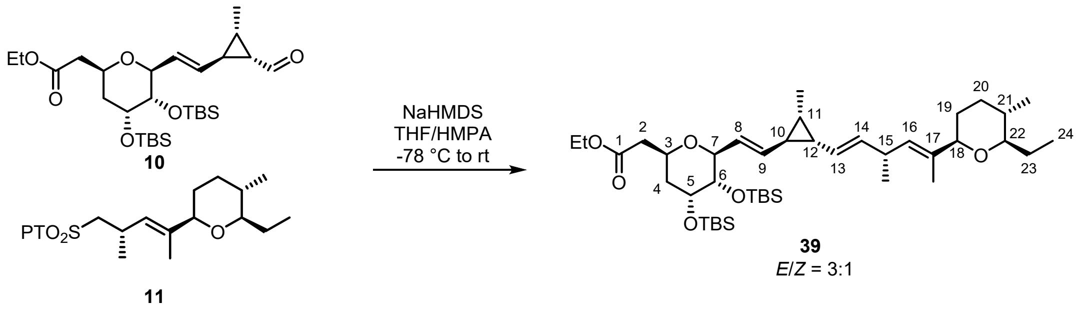

Sulfone **11** (20 mg, 0.048 mmol) was dissolved in THF (0.67 mL) and HMPA (0.13 mL) and cooled to -78 °C then 1 M NaHMDS in THF (0.06 mL, 0.06 mmol) was added dropwise over 1 minutes. The reaction mixture was stirred for 15 minutes then aldehyde **10** (32 mg, 0.06 mmol) in THF (0.28 mL) and HMPA (0.07 mL) was added dropwise. The orange solution was stirred at -78 °C for 2 hours then allowed to warm to room temperature and stirred for a further 2 hours. The reaction mixture was quenched with dropwise addition of water (1 mL) then was diluted with water (25 mL), brine (10 mL) and Et_2O (40 mL). The organic layer was separated, and the aqueous layer was extracted with Et_2O (2 x 40 mL). The combined organic layers were washed with brine (2 x 50 mL), dried over $MgSO_4$ and the

solvent removed *in vacuo*. The crude material was purified by flash column chromatography (8% Et_2O in petroleum ether 60:40) to afford alkene **39** (27 mg, 78%, *E*/*Z* = 3:1) as a colourless oil; $[\alpha]_D^{24}$ = -10.0 (*c* 1, $CHCl_3$); ν_{max} (film) 2955, 2928, 2856, 1739, 1463, 1214; δ_H (500 MHz, $CDCl_3$) 0.03 (3H, s, $SiCH_3$), 0.03 (3H, s, $SiCH_3$), 0.05 (3H, s, $SiCH_3$), 0.07 (3H, s, $SiCH_3$), 0.80 (3H, d, *J* 6.6, 21-CH_3), 0.88 (9H, s, $SiC(CH_3)_3$), 0.91 (9H, s, $SiC(CH_3)_3$), 0.94 (3H, t, *J* 7.4, 24-H_3), 1.00 – 1.04 (7H, m, 11-H, 11-CH_3 and 15-CH_3), 1.05 – 1.07 (1H, m, 10-H), 1.16 – 1.21 (1H, m, 20-*H*H), 1.24 (3H, t, *J* 7.3, OCH_2CH_3), 1.31 – 1.36 (1H, m, 21-H), 1.39 – 1.48 (3H, m, 12-H, 19-*H*H and 23-*H*H), 1.52 – 1.54 (1H, m, 4-H_{ax}), 1.59 – 1.61 (1H, m, 19-H*H*), 1.62 (3H, d, *J* 1.3, 17-CH_3), 1.68 – 1.72 (1H, m, 23-H*H*), 1.75 – 1.84 (2H, m, 4-H_{eq} and 20-H*H*), 2.33 (1H, dd, *J* 15.0, 7.7, 2-*H*H), 2.61 (1H, dd, *J* 15.0, 5.9, 2-H*H*), 2.90 (1H, ddd, *J* 9.9, 7.2, 2.9, 22-H), 2.98 – 3.08 (1H, m, 15-H), 3.27 (1H, dd, *J* 9.1, 2.4, 6-H), 3.61 (1H, d, *J* 10.8, 18-H), 3.98 – 4.01 (1H, m, 5-H), 4.07 – 4.15 (3H, m, O$CH_2$$CH_3$ and 7-H), 4.20 – 4.29 (1H, m, 3-H), 5.07 (1H, ddd, *J* 15.3, 8.8, 1.4, 13-H), 5.21 (1H, dt, *J* 8.8, 1.3, 16-H), 5.25 (1H, dd, *J* 15.4, 8.6, 9-H), 5.36 (dd, *J* 15.4, 7.5, 8-H), 5.42 (1H, dd, *J* 15.3, 6.5, 14-H); δ_C (126 MHz, $CDCl_3$) -4.4 ($SiCH_3$), -4.24 ($SiCH_3$), -4.20 ($SiCH_3$), -3.4 ($SiCH_3$), 9.5 (C-24), 13.0 (CH_3-17), 13.3 (CH_3-11), 14.4 (OCH_2CH_3), 18.0 (CH_3-21), 18.28 (Si*C*$(CH_3)_3$), 18.34 (Si*C*$(CH_3)_3$), 21.40 (CH_3-15), 21.44 (C-11) 26.1 ($SiC(CH_3)_3$ and C-23), 26.3 ($SiC(CH_3)_3$), 28.8 (C-12), 31.0 (C-10), 31.1 (C-19), 33.4 (C-20), 34.2 (C-21), 35.1 (C-15), 39.7 (C-4), 41.2 (C-2), 60.6 (O*C*H_2CH_3), 67.8 (C-3), 69.6 (C-5), 74.0 (C-6), 77.4 (C-7), 82.5 (C-18), 84.3 (C-22), 125.6 (C-13), 126.5 (C-8), 129.2 (C-16), 135.5 (C-9), 135.8 (C-17), 137.2 (C-9), 171.1 (C-1); HRMS (MALDI) calc. for $[C_{42}H_{76}O_6Si_2Na]$ 755.5073 Found 755.5081.

Ethyl 2-((2*S*,4*R*,5*S*,6*S*)-4,5-bis(*tert*-butyldimethylsiloxy)-6-((*E*)-2-((1*S*,2*S*,3*R*)-2-((*R*,1*E*,4*E*)-5-((2*R*,5*S*,6*R*)-6-ethyl-5-methyltetrahydro-2H-pyran-2-yl)-3-methylhexa-1,4-dien-1-yl)-3-methylcyclopropyl)vinyl)tetrahydro-2H-pyran-2-yl)acetate (39)

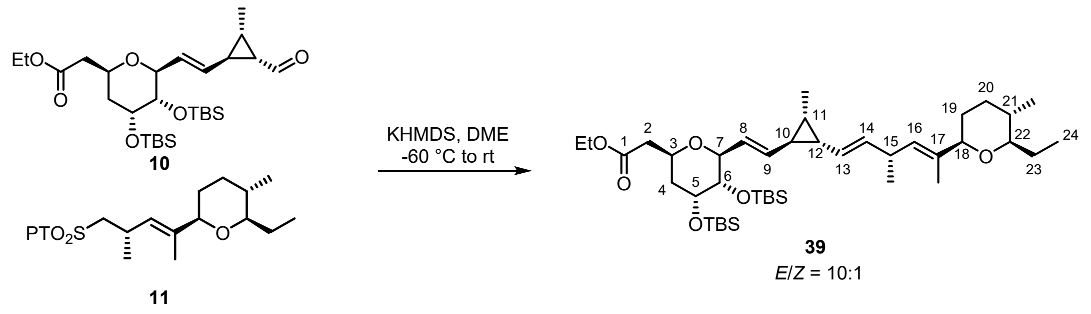

Sulfone **11** (350 mg, 0.84 mmol) was dissolved in DME (7 mL) and cooled to -60 °C then 1 M KHMDS in THF (1.00 mL, 1.00 mmol) was added dropwise over 5 minutes. The reaction mixture was stirred for 10 minutes then aldehyde **10** (543 mg, 1.00 mmol) in DME (4 mL) was added dropwise over 10 minutes. The deep red solution was stirred at -60 °C for 3 hours then allowed to warm to room temperature and stirred for a further 2 hours. The reaction mixture was quenched with dropwise addition of water (5 mL) then was diluted with water (30 mL), brine (40 mL) and Et_2O (100 mL). The

organic layer was separated, and the aqueous layer was extracted with Et_2O (2 x 100 mL). The combined organic layers were washed with brine (100 mL), dried over $MgSO_4$ and the solvent removed *in vacuo*. The crude material was purified by flash column chromatography (8% Et_2O in petroleum ether 60:40) to afford alkene **39** (244 mg, 40%, *E*/*Z* = 10:1) as a colourless oil; $[\alpha]_D^{24}$ = -10.0 (*c* 1, $CHCl_3$); ν_{max} (film) 2955, 2928, 2856, 1739, 1463, 1214; δ_H (500 MHz, $CDCl_3$) 0.03 (3H, s, $SiCH_3$), 0.03 (3H, s, $SiCH_3$), 0.05 (3H, s, $SiCH_3$), 0.07 (3H, s, $SiCH_3$), 0.80 (3H, d, *J* 6.5, 21-CH_3), 0.88 (9H, s, $SiC(CH_3)_3$), 0.91 (9H, s, $SiC(CH_3)_3$), 0.94 (3H, t, *J* 7.4, 24-H_3), 1.00 – 1.04 (7H, m, 11-H, 11-CH_3 and 15-CH_3), 1.05 – 1.08 (1H, m, 10-H), 1.17 – 1.21 (1H, m, 20-*H*H), 1.24 (3H, t, *J* 7.1, OCH_2CH_3), 1.30 – 1.37 (1H, m, 21-H), 1.43 – 1.48 (3H, m, 12-H, 19-*H*H and 23-*H*H), 1.50 – 1.54 (1H, m, 4-H_{ax}), 1.57 – 1.61 (1H, m, 19-H*H*), 1.62 (3H, d, *J* 1.4, 17-CH_3), 1.67 – 1.72 (1H, m, 23-*H*H), 1.75 – 1.84 (2H, m, 4-H_{eq} and 20-H*H*), 2.33 (1H, dd, *J* 14.8, 7.7, 2-*H*H), 2.61 (1H, dd, *J* 14.8, 5.9, 2-H*H*), 2.90 (1H, ddd, *J* 9.9, 7.3, 3.0, 22-H), 3.04 (1H, m, 15-H), 3.27 (1H, dd, *J* 9.1, 2.2, 6-H), 3.61 (1H, dt, *J* 10.7, 1.7, 18-H), 4.00 (1H, dt, *J* 4.2, 2.2, 5-H), 4.06 – 4.16 (3H, m, OCH_2CH_3 and 7-H), 4.21 – 4.27 (1H, m, 3-H), 5.07 (1H, ddd, *J* 15.4, 8.8, 1.4, 13-H), 5.21 (1H, m, 16-H), 5.25 (1H, dd, *J* 15.3, 8.7, 9-H), 5.36 (1H, dd, *J* 15.3, 7.5, 8-H), 5.42 (1H, ddd, *J* 15.4, 6.4, 0.7, 14-H); δ_C (126 MHz, $CDCl_3$) -4.4 ($SiCH_3$), -4.23 ($SiCH_3$), -4.20 ($SiCH_3$), -3.4 ($SiCH_3$), 9.5 (C-24), 13.0 (CH_3-17), 13.3 (CH_3-11), 14.4 (OCH_2CH_3), 18.0 (CH_3-21), 18.29 ($SiC(CH_3)_3$), 18.34 ($SiC(CH_3)_3$), 21.40 (CH_3-15), 21.44 (C-11) 26.1 ($SiC(CH_3)_3$ and C-23), 26.3 ($SiC(CH_3)_3$), 28.8 (C-12), 31.0 (C-10), 31.1 (C-19), 33.4 (C-20), 34.2 (C-21), 35.1 (C-15), 39.7 (C-4), 41.2 (C-2), 60.6 (OCH_2CH_3), 67.8 (C-3), 69.6 (C-5), 74.0 (C-6), 76.8 (C-7), 82.5 (C-18), 84.3 (C-22), 125.6 (C-13), 126.5 (C-8), 129.2 (C-16), 135.5 (C-14), 135.8 (C-17), 137.2 (C-9), 171.1 (C-1); HRMS (MALDI) calc. for $[C_{42}H_{76}O_6Si_2Na]$ 755.5073 Found 755.5081.

20,21-Dihydroambruticin F ethyl ester (40)

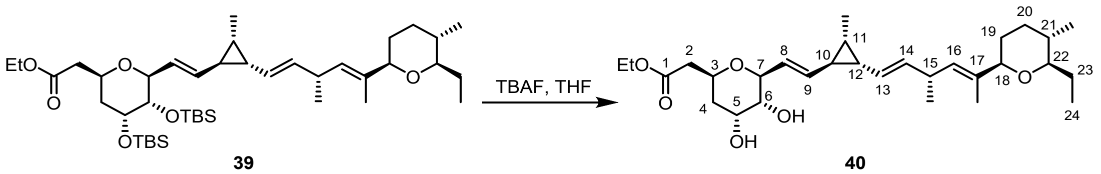

39 40

Silyl ether **39** (175 mg, 0.24 mmol, *E*/*Z* = 10:1) was dissolved in THF (2.50 mL) under nitrogen and cooled to 0 °C then 1 M TBAF in THF (1.20 mL, 1.20 mmol) was added dropwise. The reaction mixture was stirred at room temperature for 18 hours and then quenched with aqueous saturated NH_4Cl (3 mL) and water (20 mL). The aqueous layer was extracted with EtOAc (3 x 25 mL) and the combined organic layers were washed with brine (30 mL), dried over Na_2SO_4 and the solvent removed *in vacuo*. The crude material was purified by flash column chromatography (50% EtOAc in petroleum ether 60:40) to afford alcohol **40** (110 mg, 91%, *E*/*Z* = 10:1) as a colourless oil; $[\alpha]_D^{22}$ = +28.0 (*c* 0.5, $CHCl_3$); ν_{max} (film) 3439, 2961, 2926, 2874, 1736; δ_H (600 MHz, $CDCl_3$) 0.81 (3H, d, *J* 6.5, 21-CH_3), 0.94 (3H, t, *J*

7.4, 24-H_3), 1.03 (3H, d, *J* 6.8, 15-CH_3), 1.04 – 1.06 (4H, m, 11-H and 11-CH_3), 1.09 – 1.14 (1H, m, 10-H), 1.18 – 1.21 (1H, m, 20-*H*H), 1.22 – 1.27 (3H, m, OCH_2CH_3), 1.31 – 1.37 (1H, m, 21-H), 1.39 – 1.47 (2H, m, 19-*H*H and 23-*H*H), 1.47 – 1.52 (1H, m, 12-H), 1.57 – 1.65 (5H, m, 17-CH_3, 19-H*H* and 4-H_{ax}), 1.67 – 1.71 (1H, m, 23-H*H*), 1.78 (1H, ap. dq, *J* 13.0, 3.6, 20-H*H*), 1.94 (1H, s, OH), 1.99 (1H, ddd, *J* 14.1, 3.6, 2.1, 4-H_{eq}), 2.37 (1H, dd, *J* 15.1, 5.9, 2-*H*H), 2.40 (1H, s, OH), 2.55 (1H, dd, *J* 15.1, 7.2, 2-H*H*), 2.90 (1H, ddd, *J* 9.9, 7.4, 2.9, 22-H), 3.05 (1H, m, 15-H), 3.32 (1H, dd, *J* 9.5, 3.1, 6-H), 3.60 (1H, d, *J* 11.3, 18-H), 3.95 (1H, dd, *J* 9.5, 6.8, 7-H), 4.10 – 4.19 (3H, m, OCH_2CH_3 and 5-H), 4.20 – 4.27 (1H, m, 3-H), 5.07 (1H, ddd, *J* 15.3, 8.8, 1.4, 13-H), 5.21 (1H, dt, *J* 8.8, 1.4, 16-H), 5.41 – 5.49 (3H, m, 8-H, 9-H and 14-H); δ_C (126 MHz, $CDCl_3$) 9.6 (C-24), 13.1 (CH_3-17), 13.2 (CH_3-11), 14.4 (OCH_2CH_3), 18.0 (CH_3-21), 21.4 (CH_3-15), 21.8 (C-11), 26.1 (C-23), 29.3 (C-12), 30.7 (C-10), 31.1 (C-19), 33.4 (C-20), 34.3 (C-21), 35.2 (C-15), 37.1 (C-4), 40.9 (C-2), 60.7 (OCH_2CH_3), 66.6 (C-5), 68.2 (C-3), 71.2 (C-6), 77.0 (C-7), 82.3 (C-18), 84.4 (C-22), 124.5 (C-8), 125.2 (C-13), 129.0 (C-16), 135.9 (C-17), 136.0 (C-14), 139.9 (C-9), 171.0 (C-1); HRMS (ESI) calc. for [$C_{30}H_{48}O_6$] 505.3524 Found 505.3527.

20,21-Dihydroambruticin F (9)

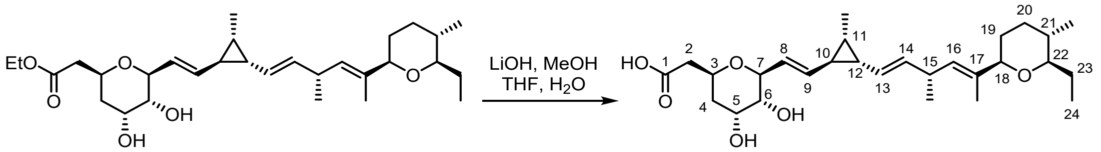

Ester **40** (30 mg, 0.06 mmol, *E*/*Z* = 10:1) was dissolved in THF (1 mL) and MeOH (0.1 mL) then a solution of LiOH (71 mg, 2.97 mmol) in water (0.4 mL) was added. The reaction mixture was stirred at room temperature for 3 hours then quenched with 1 M HCl (2 mL). The resulting solution was diluted with water (10 mL) and extracted with EtOAc (3 × 30 mL). The combined organic layers were washed with brine (20 mL), dried over Na_2SO_4 and the solvent removed *in vacuo*. The crude material was purified by flash column chromatography (10% MeOH in DCM) to afford 20,21-dihydroambruticin F **9** (24 mg, 85%, *E*/*Z* = 10:1) as a colourless oil; $[\alpha]_D^{21}$ = +20.0 (*c* 0.5, $CHCl_3$); ν_{max} (film) 3420, 2961, 2925, 2858, 1715, 1214; δ_H (400 MHz, $CDCl_3$) 0.80 (3H, d, *J* 6.5, 21-CH_3), 0.94 (3H, t, *J* 7.4, 24-H_3), 1.03 (3H, d, *J* 6.8, 15-CH_3), 1.04 – 1.07 (4H, m, 11-H and 11-CH_3), 1.09 – 1.16 (1H, m, 10-H), 1.17 – 1.23 (1H, m, 20-*H*H), 1.31 – 1.37 (1H, m, 21-H), 1.38 – 1.47 (2H, m, 19-*H*H and 23-*H*H), 1.48 – 1.54 (1H, m, 12-H), 1.56 – 1.61 (2H, m, 19-H*H* and 4-H_{ax}), 1.63 (3H, d, *J* 1.3, 17-CH_3), 1.67 – 1.73 (1H, m, 23-*H*H), 1.78 (1H, ap. dq, *J*

12.8, 3.5, 20-H*H*), 2.02 (1H, d, *J* 14.0, 4-H_{eq}), 2.44 (1H, dd, *J* 15.7, 5.8, 2-*H*H), 2.60 (1H, dd, *J* 15.7, 7.0, 2-H*H*), 2.91 (1H, ddd, *J* 10.0, 7.3, 3.1, 22-H), 2.98 – 3.11 (1H, m, 15-H), 3.33 (1H, dd, *J* 9.0, 3.0, 6-H), 3.60 (1H, d, *J* 10.9, 18-H), 3.98 (1H, dd, *J* 9.0, 7.1, 7-H), 4.18 (1H, m, 5-H), 4.22 (1H, m, 3-H), 5.07 (1H, ddd, *J* 15.3, 8.8, 1.4, 13-H), 5.21 (1H, dq, *J* 8.9, 1.3, 16-H), 5.40 – 5.52 (3H, m, 8-H, 9-H and 14-H); δ_C (126 MHz, $CDCl_3$) 9.6 (C-24), 13.18 (CH_3-17), 13.21 (CH_3-11), 17.9 (CH_3-21), 21.3 (CH_3-15), 21.8 (C-11), 26.0 (C-23), 29.3 (C-12), 30.6 (C-10), 31.0 (C-19), 33.4 (C-20), 34.3 (C-21), 35.2 (C-15), 37.0 (C-4), 40.4 (C-2), 66.6 (C-5), 68.0 (C-3), 71.2 (C-6), 77.1 (C-7), 82.3 (C-18), 84.4 (C-22), 124.1 (C-8), 125.1 (C-13), 129.0 (C-16), 135.8 (C-17), 136.1 (C-14), 140.1 (C-9), 174.7 (C-1); HRMS (MALDI) calc. for [$C_{28}H_{44}O_6Na$] 499.3030 Found 499.3036.

3.7 Synthesis of Ambruticin F and S

Ethyl 2-((2*S*,4*R*,5*S*,6*S*)-4,5-bis(*tert*-butyldimethylsiloxy)-6-((*E*)-2-((1*S*,2*S*,3*R*)-2-((*R*,1*E*,4*E*)-5-((2*R*,6*R*)-6-ethyl-5-methyl-3,6-dihydro-2H-pyran-2-yl)-3-methylhexa-1,4-dien-1-yl)-3-methylcyclopropyl)vinyl)tetrahydro-2H-pyran-2-yl)acetate (41)

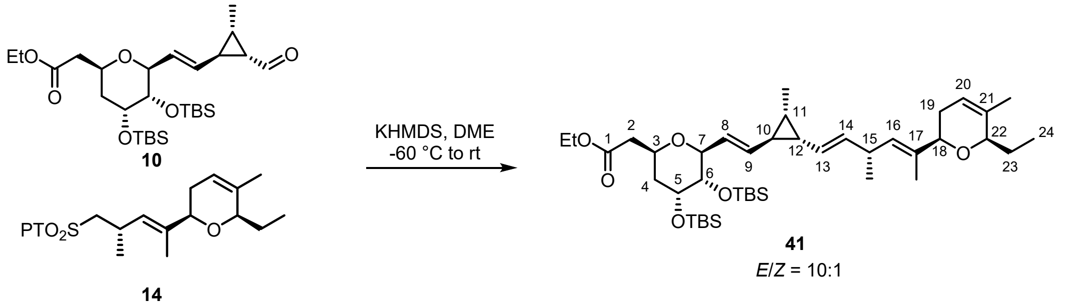

Sulfone **14**[4] (551 mg, 1.32 mmol) was dissolved in DME (13 mL) and cooled to -60 °C then 1 M KHMDS in THF (1.98 mL, 1.98 mmol) was added dropwise over 8 minutes. The reaction mixture was stirred for 10 minutes then aldehyde **10** (1070 mg, 1.98 mmol) in DME (5 mL) was added dropwise over 10 minutes. The deep red solution was stirred at -60 °C for 3 hours then allowed to warm to room temperature and stirred for a further 2 hours. The reaction mixture was quenched with dropwise addition of water (10 mL) then was diluted with water (30 mL), brine (40 mL) and Et_2O (100 mL). The organic layer was separated, and the aqueous layer was extracted with Et_2O (2 x 100 mL). The combined organic layers were washed with brine (100 mL), dried over $MgSO_4$ and the solvent removed *in vacuo*. The crude material was purified by flash column chromatography (8% Et_2O in petroleum

ether 60:40) to afford alkene **41** (367 mg, 38%, *E/Z* = 10:1) as a colourless oil; $[\alpha]_D^{27}$ = +14.0 (*c* 1, CHCl$_3$); ν_{max} (film) 2958, 2929, 2857, 1738, 1215; δ_H (400 MHz, CDCl$_3$) 0.03 (3H, s, SiCH$_3$), 0.03 (3H, s, SiCH$_3$), 0.05 (3H, s, SiCH$_3$), 0.07 (3H, s, SiCH$_3$), 0.86 – 0.89 (12H, m, SiC(CH$_3$)$_3$ and 24-H$_3$), 0.91 (9H, s, SiC(CH$_3$)$_3$), 0.96 – 1.01 (1H, m, 11-H), 1.02 – 1.03 (3H, m, 15-CH$_3$), 1.04 (3H, s, 11-CH$_3$), 1.05 – 1.08 (1H, m, 10-H), 1.23 (3H, t, *J* 7.1, OCH$_2$*CH$_3$*), 1.46 (1H, ap. dt, *J* 8.5, 4.3, 12-H), 1.49 – 1.56 (2H, m, 4-H$_{ax}$ and 23-H*H*), 1.57 – 1.60 (3H, m, 21-CH$_3$), 1.63 (3H, d, *J* 1.4, 17-CH$_3$), 1.73 – 1.88 (3H, m, 4-H$_{eq}$, 19-*H*H and 23-H*H*), 2.06 – 2.18 (1H, m, 19-H*H*), 2.33 (1H, dd, *J* 15.0, 7.7, 2-*H*H), 2.61 (1H, dd, *J* 15.0, 5.9, 2-H*H*), 3.05 (1H, ap. h, *J* 7.0, 15-H), 3.27 (1H, dd, *J* 9.1, 2.5, 6-H), 3.84 (1H, dd, *J* 10.7, 3.0, 18-H), 3.97 – 4.01 (1H, m, 5-H), 4.06 – 4.16 (4H, m, O*CH$_2$*CH$_3$, 7-H and 22-H), 4.20 – 4.29 (1H, m, 3-H), 5.07 (1H, ddd, *J* 15.2, 8.8, 1.3, 13-H), 5.20 – 5.28 (2H, m, 9-H and 16-H), 5.32 – 5.45 (2H, m, 8-H and 14-H), 5.54 – 5.59 (1H, m, 20-H); δ_C (101 MHz, CDCl$_3$) -4.4 (SiCH$_3$), -4.3 (SiCH$_3$), -4.2 (SiCH$_3$), -3.4 (SiCH$_3$), 8.3 (C-24), 12.2 (CH$_3$-17), 13.3 (CH$_3$-11), 14.4 (OCH$_2$*C*H$_3$), 18.27 (Si*C*(CH$_3$)$_3$), 18.32 (Si*C*(CH$_3$)$_3$), 19.2 (CH$_3$-21), 21.38 (CH$_3$-15), 21.43 (C-11), 25.8 (C-23), 26.0 (SiC(*C*H$_3$)$_3$), 26.3 (SiC(*C*H$_3$)$_3$), 28.8 (C-12), 30.3 (C-19), 31.1 (C-10), 35.2 (C-15), 39.6 (C-4), 41.2 (C-2), 60.6 (O*C*H$_2$CH$_3$), 67.8 (C-3), 69.6 (C-5), 74.0 (C-6), 76.8 (C-7), 77.9 (C-22), 78.4 (C-18), 121.2 (C-20), 125.7 (C-13), 126.5 (C-8), 130.0 (C-16), 135.2 (C-17 and C-21), 135.4 (C-14), 137.2 (C-9), 171.1 (C-1); HRMS (ESI) calc. for [$C_{42}H_{74}O_6Si_2Na$] 753.4916 Found 753.1919.

Ethyl 2-((2*S*,4*R*,5*S*,6*S*)-4,5-bis(*tert*-butyldimethylsiloxy)-6-((*E*)-2-((1*S*,2*S*,3*R*)-2-((*R*,1*E*,4*E*)-5-((2*R*,6*R*)-6-ethyl-5-methyl-3,6-dihydro-2H-pyran-2-yl)-3-methylhexa-1,4-dien-1-yl)-3-methylcyclopropyl)vinyl)tetrahydro-2H-pyran-2-yl)acetate (41)

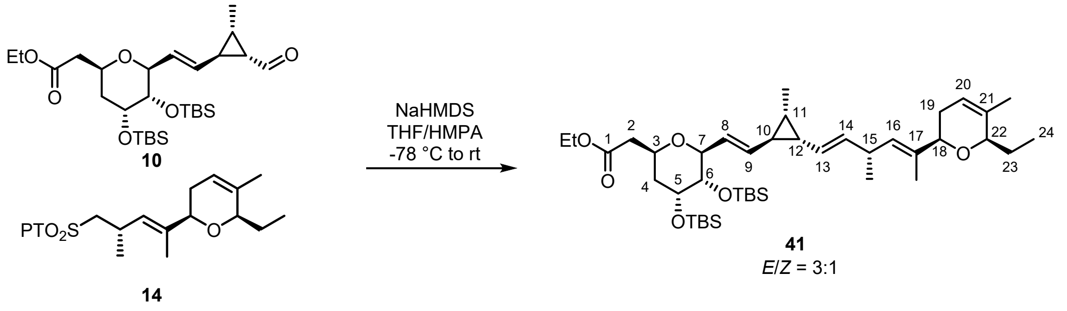

Sulfone **14**[4] (25 mg, 0.06 mmol) was dissolved in THF (0.70 mL) and HMPA (0.18 mL) and cooled to -78 °C then 1 M NaHMDS in THF (0.08 mL, 0.08 mmol) was added dropwise over 1 minute. The reaction mixture was stirred for 15 minutes then aldehyde **10** (42 mg, 0.08 mmol) in THF (0.30 mL) and HMPA (0.08 mL) was added dropwise. The orange solution was stirred at -78 °C for 2 hours then allowed to warm to room temperature and stirred for a further 2 hours. The reaction mixture was quenched with dropwise addition of water (1 mL) then was diluted with water (25 mL), brine (10 mL) and Et$_2$O (40 mL). The organic layer was separated, and the aqueous layer was extracted with Et$_2$O (2 x 40 mL). The combined organic layers were washed with brine (2 x 50 mL), dried over MgSO$_4$ and the solvent removed *in vacuo*. The crude material was purified by flash column chromatography (8% Et$_2$O in

petroleum ether 60:40) to afford alkene **41** (35 mg, 80%, *E/Z* = 3:1) as a colourless oil. Data consistent with previously reported.

Ambruticin F ethyl ester (42)

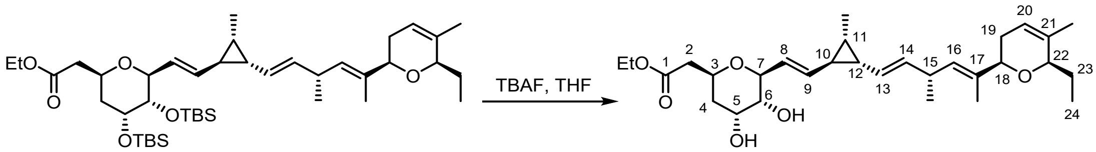

41 **42**

Silyl ether **41** (367 mg, 0.50 mmol, *E/Z* = 10:1) was dissolved in THF (5 mL) under nitrogen and cooled to 0 °C then 1 M TBAF in THF (4.0 mL, 4.0 mmol) was added dropwise. The reaction mixture was stirred at room temperature for 18 hours and then quenched with aqueous saturated NH_4Cl (10 mL) and water (40 mL). The aqueous layer was extracted with EtOAc (3 x 50 mL) and the combined organic layers were washed with brine (50 mL), dried over Na_2SO_4 and the solvent removed *in vacuo*. The crude material was purified by flash column chromatography (50% EtOAc in petroleum ether 60:40) to afford diol **42** (206 mg, 82%, *E/Z* = 10:1) as a yellow oil; $[\alpha]_D^{22}$ = +54.0 (*c* 1, $CHCl_3$); ν_{max} (film) 3438, 2963, 2923, 1735, 1214; δ_H (500 MHz, $CDCl_3$) 0.89 (3H, t, *J* 7.3, 24-H_3), 1.02 – 1.07 (7H, m, 11-H, 11-CH_3 and 15-CH_3), 1.10 – 1.14 (1H, m, 10-H), 1.24 (3H, t, *J* 7.1, OCH_2CH_3), 1.46 – 1.51 (1H, m, 12-H), 1.52 – 1.57 (1H, m, 23-*H*H), 1.59 (3H, ap. dq, *J* 2.4, 1.2, 21-CH_3), 1.60 – 1.62 (1H, m, 4-H_{ax}), 1.64 (3H, d, *J* 1.3, 17-CH_3), 1.77 (1H, m, 23-H*H*), 1.83 – 1.90 (1H, m, 19-*H*H), 1.94 – 2.01 (2H, m, 4-H_{eq} and OH), 2.08 – 2.16 (1H, m, 19-H*H*), 2.32 – 2.42 (2H, m, 2-*H*H and OH), 2.55 (1H, dd, *J* 15.2, 7.5, 2-H*H*), 3.06 (1H, dpd, *J* 8.2, 6.7, 1.4, 15-H), 3.31 (1H, dd, *J* 9.5, 3.1, 6-H), 3.84 (1H, dd, *J* 10.4, 2.7, 18-H), 3.95 (1H, dd, *J* 9.5, 6.6, 7-H), 4.07 – 4.11 (1H, m, 22-H), 4.13 (2H, ap. qd, *J* 7.1, 1.1, OCH_2CH_3), 4.16 – 4.18 (1H, m, 5-H), 4.23 (1H, dddd, *J* 11.7, 7.5, 6.1, 2.0, 3-H), 5.07 (1H, ddd, *J* 15.3, 8.7, 1.4, 13-H), 5.25 (1H, dq, *J* 8.9, 1.3, 16-H), 5.40 – 5.44 (2H, m, 8-H and 9-H), 5.44 – 5.48 (1H, m, 14-H), 5.53 – 5.59 (1H, m, 20-H); δ_C (126 MHz, $CDCl_3$) 8.4 (C-24), 12.4 (CH_3-17), 13.2 (CH_3-11), 14.4 (OCH_2CH_3), 19.2 (CH_3-21), 21.3 (CH_3-15), 21.8 (C-11), 25.8 (C-23), 29.3 (C-12), 30.3 (C-19), 30.7 (C-10), 35.2 (C-15), 37.1 (C-4), 40.9 (C-2), 60.7 (OCH_2CH_3), 66.6 (C-5), 68.2 (C-3), 71.2 (C-6), 77.0 (C-7), 78.0 (C-22), 78.2 (C-18), 121.1 (C-20), 124.6 (C-8), 125.3 (C-13), 129.7 (C-16), 135.26 (C-21), 135.31 (C-17), 135.9 (C-14), 139.9 (C-9), 171.0 (C-1); HRMS (ESI) calc. for $[C_{30}H_{46}O_6]$ 503.3367 Found 503.3383.

Ambruticin F (2)

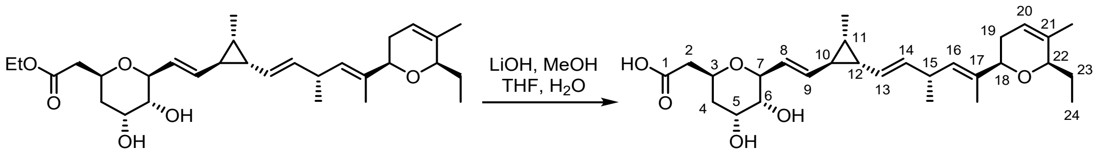

42 **2**

Ester **42** (15.0 mg, 0.03 mmol, *E/Z* = 10:1) was dissolved in THF (0.8 mL) and MeOH (0.2 mL) then a solution of LiOH (36 mg, 1.50 mmol) in water (0.4 mL) was added. The reaction mixture was stirred at room temperature for 1 hour then quenched with 1 M HCl (2 mL). The resulting solution was diluted with water (10 mL) and extracted with EtOAc (3 x 30 mL). The combined organic layers were washed with brine (20 mL), dried over Na_2SO_4 and the solvent removed *in vacuo*. The crude material was purified by flash column chromatography (10% MeOH in DCM) to afford ambruticin F **2** (12.7 mg, 87%, *E/Z* = 10:1) as a colourless oil; $[\alpha]_D^{23}$ = +50.0 (*c* 1, $CHCl_3$); ν_{max} (film) 3411, 2962, 2925, 1714, 1214; δ_H (500 MHz, $CDCl_3$) 0.89 (3H, t, *J* 7.3, 24-H_3), 1.02 – 1.07 (7H, m, 11-H, 11-CH_3 and 15-CH_3), 1.10 – 1.16 (1H, m, 10-H), 1.48 – 1.57 (2H, m, 12-H and 23-*H*H), 1.59 (3H, d, *J* 1.5, 21-CH_3), 1.60 – 1.62 (1H, m, 4-H_{ax}), 1.64 (3H, d, *J* 1.4, 17-CH_3), 1.77 (1H, dqd, *J* 14.2, 7.3, 3.7, 23-H*H*), 1.81 – 1.92 (1H, m, 19-*H*H), 1.97 – 2.06 (1H, m, 4-H_{eq}), 2.07 – 2.17 (1H, m, 19-H*H*), 2.46 (1H, dd, *J* 15.7, 5.6, 2-*H*H), 2.59 (1H, dd, *J* 15.7, 7.2, 2-H*H*), 3.02 – 3.13 (1H, m, 15-H), 3.33 (1H, dd, *J* 9.5, 3.0, 6-H), 3.84 (1H, dd, *J* 10.7, 3.1, 18-H), 3.99 (1H, dd, *J* 9.5, 7.0, 7-H), 4.07 – 4.15 (1H, m, 22-H), 4.18 (1H, ap. q, *J* 3.0, 5-H), 4.20 – 4.28 (1H, m, 3-H), 5.07 (1H, ddd, *J* 15.3, 8.8, 1.4, 13-H), 5.25 (1H, dq, *J* 8.9, 1.4, 16-H), 5.41 – 5.50 (3H, m, 8-H, 9-H and 14-H), 5.57 (1H, dq, *J* 6.4, 1.5, 20-H); δ_C (126 MHz, $CDCl_3$) 8.4 (C-24), 12.5 (CH_3-17), 13.2 (CH_3-11), 19.2 (CH_3-21), 21.3 (CH_3-15), 21.9 (C-11), 25.8 (C-23), 29.4 (C-12), 30.3 (C-19), 30.6 (C-10), 35.2 (C-15), 36.9 (C-4), 40.3 (C-2), 66.5 (C-5), 68.0 (C-3), 71.1 (C-6), 77.2 (C-7), 78.1 (C-22), 78.2 (C-18), 121.1 (C-20), 124.1 (C-8), 125.2 (C-13), 129.7 (C-16), 135.2 (C-21), 135.3 (C-17), 136.0 (C-14), 140.4 (C-9), 174.3 (C-1); HRMS (ESI) calc. for [$C_{28}H_{42}O_6Na$] 497.2874 Found 497.2970.

δ_H (600 MHz, MeOD) δ 0.91 (3H, ap. t, *J* 7.3, 24-H_3), 1.06 (3H, d, *J* 6.9, 15-CH_3), 1.07 (4H, m, 11-H and 11-CH_3), 1.13 (1H, m, 10-H), 1.48 (1H, m, 12-H), 1.52 – 1.57 (1H, m, 23-*H*H), 1.57 – 1.60 (1H, m, 4-H_{ax}), 1.60 – 1.63 (3H, m, 21-CH_3), 1.66 (3H, d, *J* 1.4, 17-CH_3), 1.78 (1H, dqd, *J* 14.8, 7.3, 3.6, 23-H*H*), 1.85 – 1.90 (1H, m, 19-*H*H), 1.92 (1H, ddd, *J* 13.9, 3.5, 2.1, 4-H_{eq}), 2.09 – 2.16 (1H, m, 19-H*H*), 2.37 (1H, dd, *J* 15.2, 5.4, 2-*H*H), 2.44 (1H, dd, *J* 15.2, 7.9, 2-H*H*), 3.07 – 3.14 (1H, m, 15-H), 3.22 (1H, dd, *J* 9.7, 3.5, 6-H), 3.85 (1H, dd, *J* 10.8, 3.0, 18-H), 3.99 (1H, dd, *J* 9.7, 6.5, 7-H), 4.04 (1H, ap. q, *J* 3.5, 5-H), 4.10 (1H, m, 22-H), 4.19 – 4.26 (1H, m, 3-H), 5.17 (1H, ddd, *J* 15.3, 8.7, 1.3, 13-H), 5.27 (1H, dq, *J* 9.0, 1.4, 16-H), 5.40 (1H, dd, *J* 15.4, 8.6, 9-H), 5.47 (1H, dd, *J* 15.3, 6.6, 14-H), 5.50 (1H, dd, *J* 15.4, 6.5, 8-H), 5.59 – 5.62 (1H, m, 20-H); δ_C (151 MHz, $CDCl_3$) 8.7 (C-24), 12.6 (CH_3-17), 13.4 (CH_3-11), 19.1 (CH_3-21), 21.7 (CH_3-15), 22.3 (C-11), 26.6 (C-23), 29.8 (C-12), 31.1 (C-19), 31.8 (C-10), 36.3 (C-15), 39.2 (C-4), 41.8 (C-2), 68.5 (C-5), 69.4 (C-3), 73.0 (C-6), 77.4 (C-7), 79.5 (C-18), 79.6 (C-22), 122.1 (C-20), 126.9 (C-8), 127.0 (C-13), 131.0 (C-16), 136.1 (C-21), 136.21 (C-14), 136.22 (C-17), 137.9 (C-9), 175.2 (C-1).

5-Ketoambruticin F ethyl ester (43)

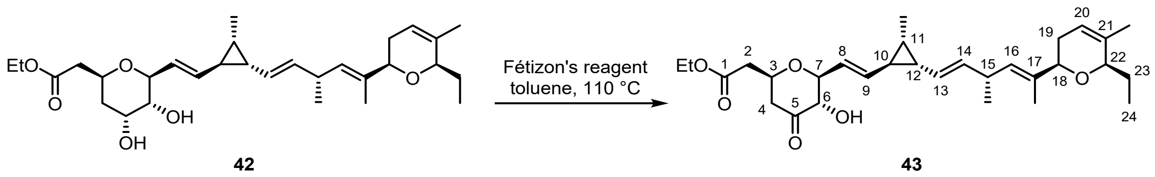

Diol **42** (99 mg, 0.20 mmol) was dissolved in toluene (40 mL) under nitrogen and Fetizon's reagents (~50 wt. % Ag_2CO_3 on celite, 1000 mg) was added. The reaction mixture was heated at 110 °C with vigorous stirring for 2 hours by which time the reaction mixture had turned black. Further Fetizon's reagent (1000 mg) was added and the reaction mixture was stirred for a further 2 hours. Further Fetizon's reagent (1000 mg) was added and the reaction mixture was heated at 110 °C for 2 hours. The reaction mixture was filtered over celite and the solvent removed *in vacuo*. The crude material was purified by flash column chromatography (25% EtOAc in petroleum ether 60:40) to afford ketone **43** (53 mg, 54%, *E/Z* = 10:1) as a colourless oil; $[\alpha]_D^{25}$ = +48.0 (*c* 0.5, $CHCl_3$); ν_{max} (film) 3486, 2963, 2929, 2876, 1725, 1214; δ_H (500 MHz, $CDCl_3$) 0.89 (3H, t, *J* 7.3, 24-H_3), 1.01 – 1.09 (7H, m, 11-H, 11-CH_3 and 15-CH_3), 1.16 (1H, ap. dt, *J* 9.0, 4.4, 10-H), 1.26 (3H, t, *J* 7.1, OCH_2CH_3), 1.46 – 1.55 (2H, m, 12-H and 23-*H*H), 1.59 (3H, ap. dq, *J* 2.6, 1.3, 21-CH_3), 1.64 (3H, d, *J* 1.3, 17-CH_3), 1.77 (1H, dqd, *J* 14.2, 7.3, 3.6, 23-H*H*), 1.83 – 1.89 (1H, m, 19-*H*H), 2.08 – 2.17 (1H, m, 19-H*H*), 2.52 – 2.61 (2H, m, 2-*H*H and 4-H_{ax}), 2.71 (1H, dd, *J* 13.7, 2.4, 4-H_{eq}), 2.76 (1H, dd, *J* 15.6, 6.6, 2-H*H*), 3.03 – 3.12 (1H, m, 15-H), 3.54 (1H, d, *J* 3.7, 6-OH), 3.70 (1H, ddd, *J* 9.6, 6.4, 1.0, 7-H), 3.85 (1H, dd, *J* 10.5, 3.0, 18-H), 3.93 (1H, ddd, *J* 9.6, 3.7, 1.5, 6-H), 4.05 – 4.12 (2H, m, 3-H and 22-H), 4.16 (2H, q, *J* 7.1, OCH_2CH_3), 5.08 (1H, ddd, *J* 15.3, 8.8, 1.4, 13-H), 5.25 (1H, dq, *J* 8.8, 1.3, 16-H), 5.39 – 5.50 (2H, m, 9-H and 14-H), 5.55 – 5.59 (1H, m, 20-H), 5.61 (1H, dd, *J* 15.3, 6.4, 8-H); δ_C (126 MHz, $CDCl_3$) 8.4 (C-24), 12.4 (CH_3-17), 13.2 (CH_3-11), 14.4 (OCH_2CH_3), 19.2 (CH_3-21), 21.4 (CH_3-15), 21.7 (C-11), 25.8 (C-23), 29.2 (C-12), 30.4 (C-19), 30.8 (C-10), 35.2 (C-15), 41.1 (C-2), 45.8 (C-4), 61.1 (OCH_2CH_3), 73.9 (C-3), 77.0 (C-6), 78.0 (C-22), 78.2 (C-18), 83.1 (C-7), 121.1 (C-20), 123.7 (C-8), 125.4 (C-13), 129.8 (C-16), 135.28 (C-21), 135.30 (C-17), 135.8 (C-14), 138.7 (C-9), 170.0 (C-1), 206.4 (C-5); HRMS (ESI) calc. for $[C_{30}H_{44}O_6Na]$ 523.3030 Found 523.3026.

Ambruticin S ethyl ester (S8)

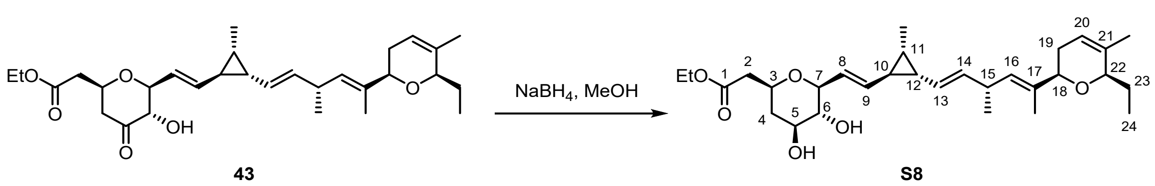

Ketone **43** (25 mg, 0.05 mmol) was dissolved in MeOH (5 mL) under nitrogen and cooled to 0 °C then $NaBH_4$ (19 mg, 0.5 mmol) was added. The reaction mixture was stirred at room temperature for 2 hours then quenched with aqueous saturated NH_4Cl (30 mL). The resulting solution was extracted with EtOAc (3 x 50 mL) and the combined organic layers were washed with brine (30 mL), dried over Na_2OS_4

and the solvent removed *in vacuo*. The crude material was purified by flash column chromatography (60% EtOAc in petroleum ether 60:40) to afford 1,2-*anti*-diol **S8** (11.8 mg, 47%, *E*/*Z* = 10:1) and 1,2-*syn*-diol **42** (8.8 mg, 35%, *E*/*Z* = 10:1) as colourless oils. Data for 1,2-*syn*-diol **42** consistent with previously reported.

1,2-*anti*-diol **S8**:

$[\alpha]_D^{24}$ = +66.0 (*c* 1, $CHCl_3$); ν_{max} (film) 3395, 2962, 2925, 2872, 1736, 1214; δ_H (500 MHz, $CDCl_3$) 0.89 (3H, t, *J* 7.3, 24-H_3), 1.01 – 1.08 (7H, m, 11-H, 11-CH_3 and 15-CH_3), 1.10 – 1.16 (1H, m, 10-H), 1.25 (3H, t, *J* 7.1, OCH_2CH_3), 1.42 – 1.50 (2H, m, 4-H_{ax} and 12-H), 1.52 – 1.57 (1H, m, 23-*H*H), 1.58 – 1.60 (3H, m, 21-CH_3), 1.64 (3H, d, *J* 1.3, 17-CH_3), 1.77 (1H, dqd, *J* 14.3, 7.3, 3.7, 23-H*H*), 1.86 (1H, dddq, *J* 16.6, 6.5, 3.0, 1.4, 19-*H*H), 2.02 (1H, s, OH), 2.06 – 2.13 (2H, m, 4-H_{eq} and 19-H*H*), 2.39 – 2.48 (2H, m, OH and 2-*H*H), 2.63 (1H, dd, *J* 15.5, 6.5, 2-H*H*), 3.03 – 3.10 (1H, m, 15-H), 3.13 (1H, ap. t, *J* 9.0, 6-H), 3.53 (1H, ddd, *J* 9.0, 4.6, 2.2, 7-H), 3.66 – 3.73 (1H, m, 5-H), 3.84 (1H, dd, *J* 10.6, 3.0, 18-H), 3.91 (1H, ap. dtd, *J* 11.5, 6.5, 1.9, 3-H), 4.07 – 4.11 (1H, m, 22-H), 4.14 (2H, q, *J* 7.1, OCH_2CH_3), 5.07 (1H, ddd, *J* 15.3, 8.8, 1.4, 13-H), 5.25 (1H, dq, *J* 8.9, 1.3, 16-H), 5.39 – 5.49 (3H, m, 8-H, 9-H and 14-H), 5.57 (1H, dq, *J* 6.5, 1.7, 20-H); δ_C (126 MHz, $CDCl_3$) 8.4 (C-24), 12.4 (CH_3-17), 13.2 (CH_3-11), 14.4 (OCH_2CH_3), 19.2 (CH_3-21), 21.3 (CH_3-15), 21.8 (C-11), 25.8 (C-23), 29.3 (C-12), 30.4 (C-19), 30.7 (C-10), 35.2 (C-15), 38.3 (C-4), 40.9 (C-2), 60.8 (OCH_2CH_3), 72.0 (C-3), 72.4 (C-5), 75.9 (C-6), 78.0 (C-22), 78.2 (C-18), 80.9 (C-7), 121.1 (C-20), 123.8 (C-8), 125.2 (C-13), 129.7 (C-16), 135.27 (C-21), 135.32 (C-17), 135.9 (C-14), 139.9 (C-9), 170.9 (C-1); HRMS (ESI) calc. for $[C_{30}H_{46}O_6Na]$ 525.3187 Found 525.3182.

Ambruticin S (4)

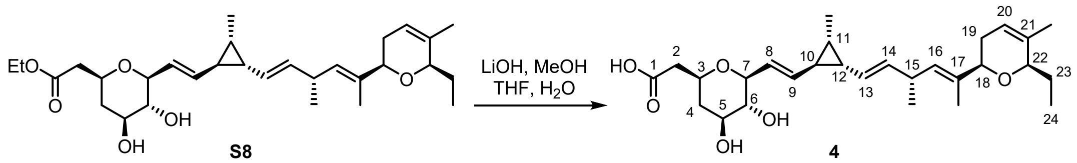

Ester **S8** (10.0 mg, 0.02 mmol, *E*/*Z* = 10:1) was dissolved in THF (0.8 mL) and MeOH (0.2 mL) then a solution of LiOH (24 mg, 1.00 mmol) in water (0.4 mL) was added. The reaction mixture was stirred at room temperature for 1 hour then quenched with 1 M HCl (2 mL). The resulting solution was diluted with water (10 mL) and extracted with EtOAc (3 x 30 mL). The combined organic layers were washed with brine (20 mL), dried over Na_2SO_4 and the solvent removed *in vacuo*. The crude material was purified by flash column chromatography (10% MeOH in DCM) to afford ambruticin S **4** (7.7 mg, 81%, *E*/*Z* = 10:1) as a colourless oil; $[\alpha]_D^{21}$ = +64.0 (*c* 0.5, $CHCl_3$), lit.[11] $[\alpha]_D^{23}$ = +58.0 (*c* 0.1, $CHCl_3$); ν_{max} (film) 3398, 2960, 2926, 1715, 1064; δ_H (500 MHz, $CDCl_3$) 0.89 (3H, t, *J* 7.3, 24-H_3), 1.02 – 1.08 (7H, m, 11-H, 11-CH_3 and 15-CH_3), 1.10 – 1.16 (1H, m, 10-H), 1.44 – 1.49 (1H, m, 4-H_{ax}), 1.49 – 1.53 (1H, m, 12-H),

1.53 – 1.57 (1H, m, 23-*H*H), 1.59 (3H, dd, *J* 2.5, 1.3, 21-CH_3), 1.64 (3H, d, *J* 1.4, 17-CH_3), 1.77 (1H, dqd, *J* 14.2, 7.3, 3.7, 23-H*H*), 1.86 (1H, m, 19-H*H*), 2.07 – 2.16 (2H, m, 4-H_{eq} and 19-H*H*), 2.51 (1H, dd, *J* 16.1, 5.9, 2-*H*H), 2.67 (1H, dd, *J* 16.1, 7.1, 2-H*H*), 3.03 – 3.12 (1H, m, 15-H), 3.15 (1H, ap. t, *J* 8.9, 6-H), 3.52 – 3.61 (1H, m, 7-H), 3.67 – 3.75 (1H, m, 5-H), 3.85 (1H, dd, *J* 10.7, 3.0, 18-H), 3.87 – 3.93 (1H, m, 3-H), 4.07 – 4.12 (1H, m, 22-H), 5.07 (1H, ddd, *J* 15.3, 8.8, 1.4, 13-H), 5.25 (1H, dq, *J* 8.9, 1.4, 16-H), 5.40 – 5.52 (3H, m, 8-H, 9-H and 14-H), 5.56 – 5.58 (1H, m, 20-H); δ_C (126 MHz, $CDCl_3$) 8.4 (C-24), 12.5 (CH_3-17), 13.2 (CH_3-11), 19.2 (CH_3-21), 21.3 (CH_3-15), 21.8 (C-11), 25.8 (C-23), 29.3 (C-12), 30.3 (C-19), 30.7 (C-10), 35.2 (C-15), 38.2 (C-4), 40.3 (C-2), 71.7 (C-3), 72.1 (C-5), 75.8 (C-6), 78.1 (C-22), 78.2 (C-18), 81.1 (C-7), 121.1 (C-20), 123.5 (C-8), 125.2 (C-13), 129.7 (C-16), 135.26 (C-21), 135.32 (C-17), 136.0 (C-14), 140.2 (C-9), 174.0 (C-1); HRMS (MALDI) calc. for [$C_{28}H_{42}O_6Na$] 497.2874 Found 497.2869.

δ_H (500 MHz, CD_3OD) 0.89 (3H, t, *J* 7.3, 24-H_3), 1.04 (3H, d, *J* 6.8, 15-CH_3), 1.05 – 1.08 (4H, m, 11-H and 11-CH_3), 1.10 – 1.14 (1H, m, 10-H), 1.35 – 1.39 (1H, m, 4-*H*H), 1.45 – 1.50 (1H, m, 12-H), 1.52 – 1.57 (1H, m, 23-*H*H), 1.60 (3H, d, *J* 1.1, 21-CH_3), 1.64 (3H, d, *J* 1.3, 17-CH_3), 1.74 – 1.80 (1H, m, 23-H*H*), 1.84 – 1.90 (1H, m, 19-*H*H), 2.05 (1H, ddd, *J* 12.6, 5.0, 1.6, 4-H*H*), 2.08 – 2.15 (1H, m, 19-H*H*), 2.44 (1H, dd, *J* 15.6, 5.4, 2-*H*H), 2.50 (1H, dd, *J* 15.6, 7.6, 2-H*H*), 2.97 (1H, ap. t, *J* 9.1, 6-H), 3.07 – 3.13 (1H, m, 15-H), 3.51 (1H, dd, *J* 9.1, 6.6, 7-H), 3.53 – 3.57 (1H, m, 5-H), 3.83 (1H, dd, *J* 11.1, 3.3, 18-H), 3.85 – 3.89 (1H, m, 3-H), 4.08 – 4.11 (1H, m, 22-H), 5.16 (1H, ddd, *J* 15.2, 8.7, 1.3, 13-H), 5.26 (1H, dq, *J* 9.0, 1.3, 16-H), 5.38 (1H, dd, *J* 15.4, 8.6, 9-H), 5.46 (1H, dd, *J* 15.2, 6.5, 14-H), 5.50 (1H, dd, *J* 15.4, 6.6, 8-H), 5.57 – 5.61 (1H, m, 20-H); δ_C (126 MHz, CD_3OD) 8.7 (C-24), 12.6 (CH_3-17), 13.4 (CH_3-11), 19.1 (CH_3-21), 21.7 (CH_3-15), 22.3 (C-11), 26.6 (C-23), 29.8 (C-12), 31.1 (C-19), 31.7 (C-10), 36.3 (C-15), 40.2 (C-4), 41.6 (C-2), 73.3 (C-5), 73.4 (C-5), 77.1 (C-6), 79.5 (C-18), 79.6 (C-22), 81.8 (C-7), 122.1 (C-20), 126.2 (C-8), 126.9 (C-13), 130.9, (C-16) 136.1 (C-21), 136.2 (C-17), 136.3 (C-14), 138.2 (C-9), 174.7 (C-1). Data consistent with the literature.[10–12]

4. Comparison of NMR data for 20,21-Dihydroambruticin F

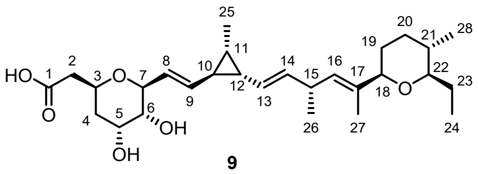

9

Table S1. Comparison of ^{1}H-NMR data between synthetic and natural 20,21-dihydroambruticin F.

**Position**	**Synthetic 20,21-dihydroambruticin F 400 MHz ($CDCl_3$): δ_H (multiplet, *J* (Hz))**	**Natural 20,21-dihydroambruticin F 500 MHz ($CDCl_3$): δ_H (multiplet, *J* (Hz))**
28-H	0.80 (d, 6.5)	0.81 (d, 6.5)
24-H	0.94 (t, 7.4)	0.94 (t, 7.4)
26-H	1.03 (d, 6.8)	1.04 (d, 6.8)
11-H	1.04 – 1.07 (m)	1.05 – 1.07 (m)
25-H	1.04 – 1.07 (m)	1.05 – 1.07 (m)
10-H	1.09 – 1.16 (m)	1.10 – 1.15 (m)
20-*H*H	1.17 – 1.23 (m)	1.16 – 1.24 (m)
21-H	1.31 – 1.37 (m)	1.31 – 1.38 (m)
19-*H*H	1.38 – 1.47 (m)	1.40 – 1.46 (m)
23-*H*H	1.38 – 1.47 (m)	1.40 – 1.46 (m)
12-H	1.48 – 1.54 (m)	1.48 – 1.54 (m)
19-*H*H	1.56 – 1.61 (m)	1.56 – 1.61 (m)
4-*H*H	1.56 – 1.61 (m)	1.56 – 1.61 (m)

27-H	1.63 (d, 1.3)	1.63 (d, 1.4)
23-H*H*	1.67 – 1.73 (m)	1.70 (dqd, 14.9, 7.4, 3.0)
20-H*H*	1.78 (ap. dq, 12.8, 3.5)	1.79 (dq, 13.0, 3.5)
4-H*H*	2.02 (d, 14.0)	2.02 (d, 13.9)
2-*H*H	2.44 (dd, 15.7, 5.8)	2.45 (d, 15.1)
2-H*H*	2.60 (dd, 15.7, 7.0)	2.55 – 2.63 (m)
22-H	2.91 (ddd, 10.0, 7.3, 3.1)	2.90 (ddd, 10.0, 7.4, 3.0)
15-H	2.98 – 3.11 (m)	3.00 – 3.10 (m)
6-H	3.33 (dd, 9.5, 3.0)	3.33 (d, 9.3)
18-H	3.60 (d, 10.9)	3.61 (dd, 11.1, 2.0)
7-H	3.98 (dd, 9.0, 7.1)	3.99 (ap. t, 8.1)
5-H	4.18 (m)	4.17 (m)
3-H	4.22 (m)	4.21 (m)
13-H	5.07 (ddd, 15.3, 8.8, 1.4)	5.07 (dd, 15.1, 8.6)
16-H	5.21 (dq, 8.9, 1.3)	5.21 (d, 8.9)
8-H	5.40 – 5.52 (m)	5.39 – 5.51 (m)
9-H	5.40 – 5.52 (m)	5.39 – 5.51 (m)
14-H	5.40 – 5.52 (m)	5.39 – 5.51 (m)

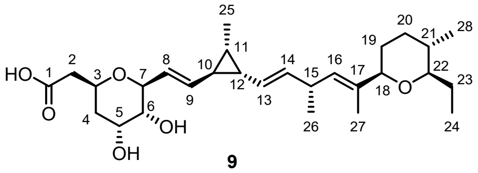

9

Table S2. Comparison of ^{13}C-NMR data between synthetic and natural 20,21-dihydroambruticin F.

**Position**	**Synthetic 20,21-dihydroambruticin F 126 MHz (CDCl$_3$): δ$_C$**	**Natural 20,21-dihydroambruticin F 126 MHz (CDCl$_3$): δ$_C$**
C-24	9.6	9.6
C-27	13.18	13.19
C-25	13.21	13.23
C-28	17.9	18.0
C-26	21.3	21.3
C-11	21.8	21.8
C-23	26.0	26.0
C-12	29.3	29.3
C-10	30.6	30.6
C-19	31.0	31.0
C-20	33.4	33.4
C-21	34.3	34.3
C-15	35.2	35.2
C-4	37.0	37.0
C-2	40.4	40.5

C-5	66.6	66.6
C-3	68.0	68.0
C-6	71.2	71.2
C-7	77.1	77.1
C-18	82.3	82.3
C-22	84.4	84.4
C-8	124.1	124.2
C-13	125.1	125.1
C-16	129.0	129.0
C-17	135.8	135.8
C-14	136.1	136.0
C-9	140.1	140.1
C-1	174.7	174.7

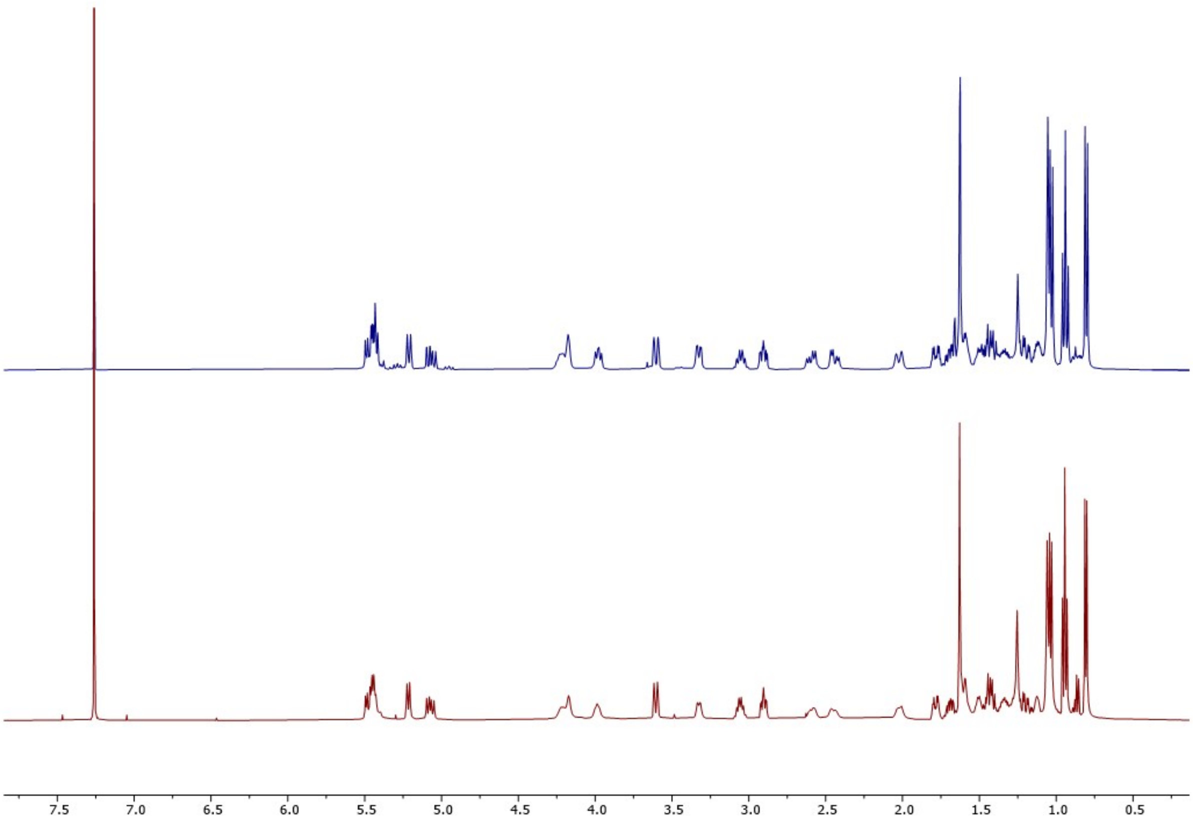

Figure S2. ^{1}H-NMR spectra of synthetic (blue) and natural (red) 20,21-dihydroambruticin F.

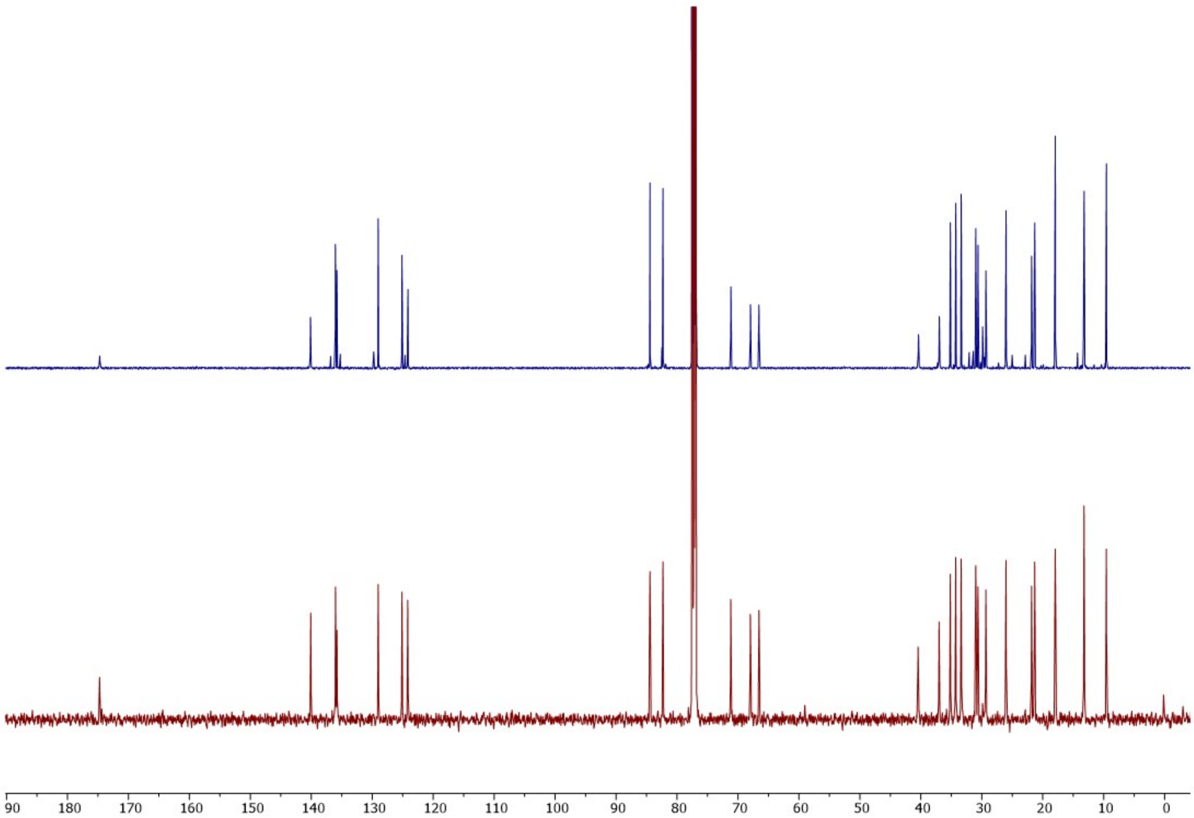

Figure S3. ^{13}C-NMR spectra of synthetic (blue) and natural (red) 20,21-dihydroambruticin F.

5. Comparison of NMR data for Ambruticin F

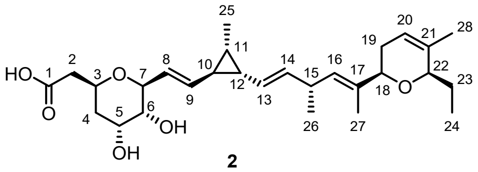

2

Table S3. Comparison of ^{1}H-NMR data between synthetic and natural ambruticin F.

Position	Synthetic Ambruticin F 600 MHz (CD_3OD): δ_H (multiplet, *J* (Hz))	Natural Ambruticin F 500 MHz (CD_3OD): δ_H (multiplet, *J* (Hz))
24-H	0.91 (ap. t, 7.3)	0.90 (ap. t, 7.3)
26-H	1.06 (d. 6.9)	1.05 (d, 6.9)
11-H	1.07 (m)	1.07 (m)
25-H	1.07 (m)	1.07 (m)
10-H	1.13 (m)	1.12 (ddd, 8.6, 6.4, 3.2)
12-H	1.48 (m)	1.47 (m)
23-*H*H	1.52 – 1.57 (m)	1.51 – 1.55 (m)
4-*H*H	1.57 – 1.60 (m)	1.55 – 1.59 (m)
28-H	1.60 – 1.63 (m)	1.60 (ap. dq, 2.4, 1.2)
27-H	1.66 (d, 1.4)	1.65 (d, 1.4)
23-H*H*	1.78 (dqd, 14.8, 7.3, 3.6)	1.77 (dqd, 14.8, 7.3, 3.6)

19-*H*H	1.85 – 1.90 (m)	1.84 – 1.89 (m)
4-*H*H	1.92 (ddd, 13.8, 3.5, 2.1)	1.91 (ddd, 14.0, 3.5, 1.9)
19-H*H*	2.09 – 2.16 (m)	2.07 – 2.17 (m)
2-*H*H	2.37 (dd, 15.2, 5.4)	2.35 (dd, 15.2, 5.4)
2-H*H*	2.44 (dd, 15.2, 7.9)	2.43 (dd, 15.2, 7.8)
15-H	3.07 – 3.14 (m)	3.05 – 3.15 (m)
6-H	3.22 (dd, 9.7, 3.5)	3.21 (dd, 9.7, 3.5)
18-H	3.85 (dd, 10.8, 3.0)	3.84 (dd, 10.7, 3.0)
7-H	3.99 (dd, 9.7, 6.5)	3.98 (dd, 9.7, 6.6)
5-H	4.04 (ap. q, 3.5)	4.04 (ap. q, 3.5)
22-H	4.10 (m)	4.10 (m)
3-H	4.19 – 4.26 (m)	4.16 – 4.25 (m)
13-H	5.17 (ddd, 15.3, 8.7, 1.3)	5.17 (ddd, 15.3, 8.8, 1.3)
16-H	5.27 (dq, 9.0, 1.4)	5.26 (dq, 9.1, 1.4)
9-H	5.40 (dd, 15.4, 8.6)	5.39 (dd, 15.4, 8.6)
14-H	5.47 (dd, 15.3, 6.6)	5.46 (dd, 15.3, 6.9)
8-H	5.50 (dd, 15.4, 6.5)	5.49 (dd, 15.4, 6.6)
20-H	5.59 – 5.62 (m)	5.56 – 5.61 (m)

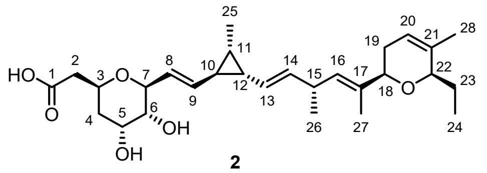

2

Table S4. Comparison of ^{13}C-NMR data between synthetic and natural ambruticin F.

**Position**	**Synthetic Ambruticin F 151 MHz (CD_3OD): δ_C**	**Natural Ambruticin F 126 MHz (CD_3OD): δ_C**
C-24	8.7	8.7
C-27	12.6	12.6
C-25	13.4	13.4
C-28	19.1	19.1
C-26	21.7	21.7
C-11	22.3	22.3
C-23	26.6	26.6
C-12	29.8	29.8
C-19	31.1	31.1
C-10	31.8	31.8
C-15	36.3	36.3
C-4	39.2	39.2
C-2	41.8	42.1
C-5	68.5	68.5
C-3	69.4	69.5

C-6	73.0	73.0
C-7	77.4	77.4
C-18	79.5	79.5
C-22	79.6	79.6
C-20	122.1	122.1
C-8	126.9	126.9
C-13	127.0	127.0
C-16	131.0	131.0
C-21	136.1	136.0
C-17	136.21	136.20
C-14	136.22	136.22
C-9	137.9	138.0
C-1	175.2	175.3

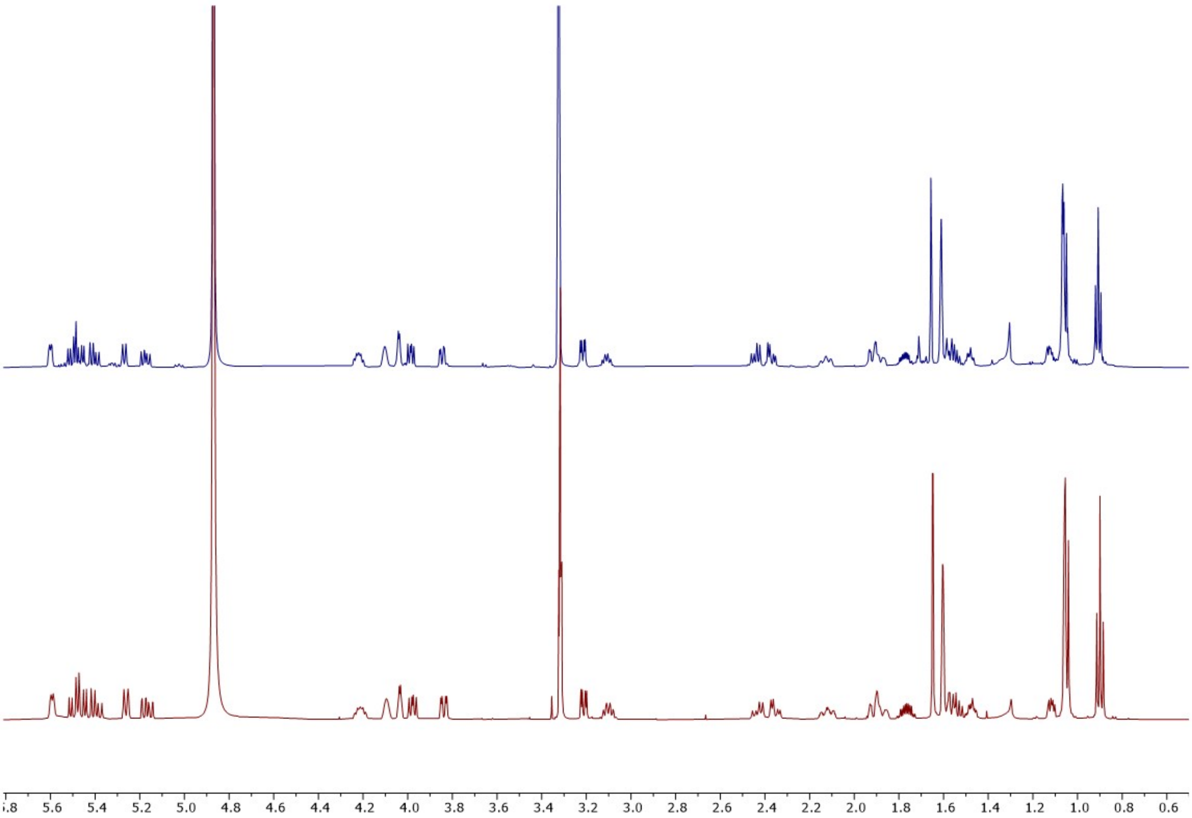

Figure S4. ^{1}H-NMR spectra of synthetic ambruticin F (blue) and natural ambruticin F (red).

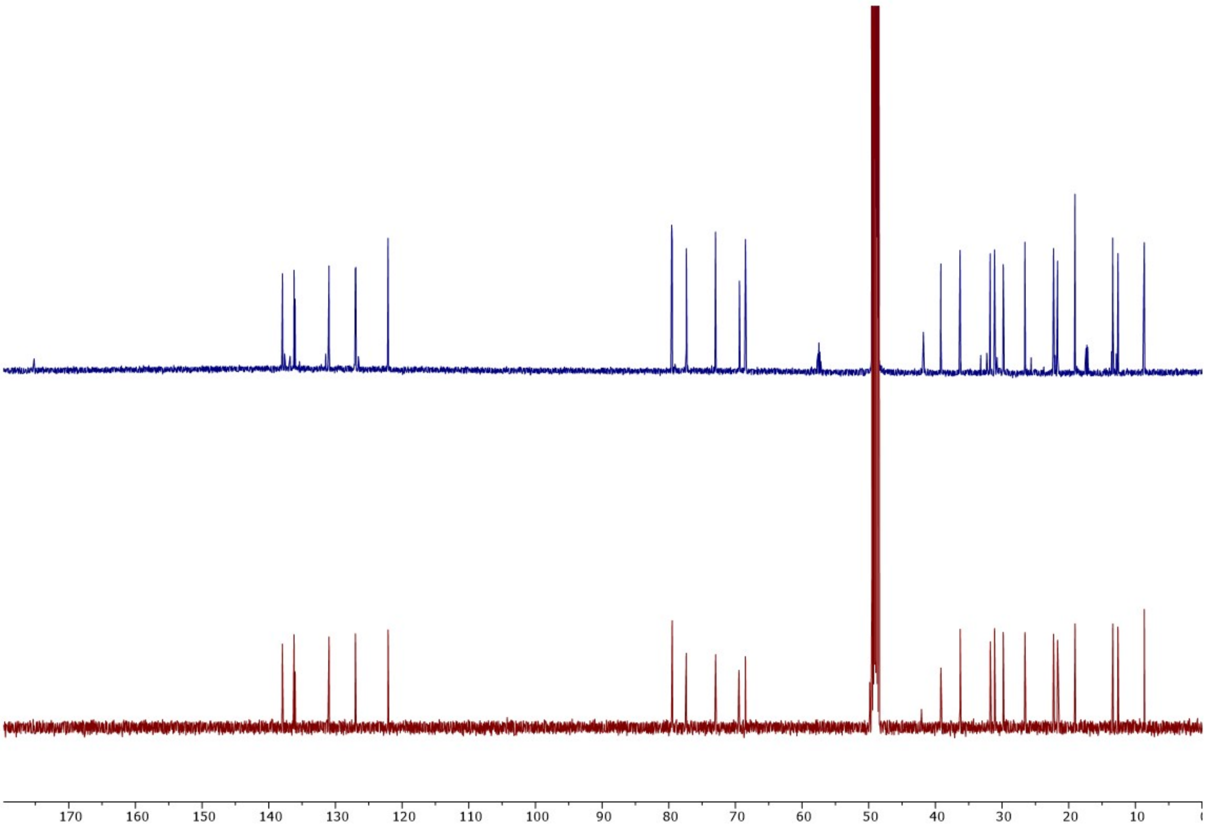

Figure S5. ^{13}C-NMR spectra of synthetic ambruticin F (blue) and natural ambruticin F (red).

6. Comparison of NMR data for Ambruticin S

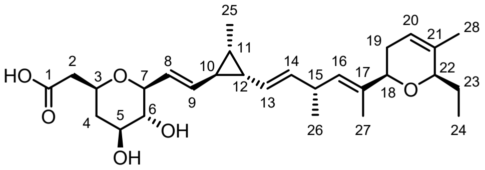

4

Table S5. Comparison of ^{1}H-NMR data between synthetic and natural ambruticin S.[10–12]

Position	Synthetic Ambruticin S 500 MHz (CD_3OD): δ_H (multiplet, *J* (Hz))	Natural Ambruticin S 400 MHz (CD_3OD): δ_H (multiplet, *J* (Hz))	Hanessian's Synthetic Ambruticin S 500 MHz (CD_3OD): δ_H (multiplet, *J* (Hz))
24-H	0.89 (t, 7.3)	0.93 (t, 7.3)	0.91 (t, 7.3)
26-H	1.04 (d, 6.8)	1.08 (d, 7.0)	1.06 (d, 6.8)
11-H	1.05 – 1.08 (m)	1.13 (m)	1.04 – 1.06 (m)
25-H	1.05 – 1.08 (m)	1.09 (s)	1.06 – 1.07 (m)
10-H	1.10 – 1.14 (m)	1.16 (m)	1.13 – 1.15 (m)
4-*H*H	1.35 – 1.39 (m)	1.38 (m)	1.36 (q, 12.0)
12-H	1.45 – 1.50 (m)	1.54 (m)	1.48 – 1.51 (m)
23-*H*H	1.52 – 1.57 (m)	1.58 (m)	1.52 – 1.59 (m)
28-H	1.60 (d, 1.1)	1.64 (m)	1.62 (d, 1.1)
27-H	1.64 (d, 1.3)	1.68 (d, 1.3)	1.66 (d, 1.3)
23-H*H*	1.74 – 1.80 (m)	1.80 (m)	1.75 – 1.81 (m)

19-*H*H	1.84 – 1.90 (m)	1.90 (m)	1.86 – 1.91 (m)
4-*H*H	2.05 (ddd, 12.6, 5.0, 1.6)	2.08 (ddd, 12.6, 5.1, 1.9)	2.06 (ddd, 12.6, 5.0, 1.6)
19-H*H*	2.08 – 2.15 (m)	2.13 (m)	2.11 – 2.16 (m)
2-*H*H	2.44 (dd, 15.6, 5.4)	2.47 (dd, 15.6, 5.5)	2.46 (dd, 15.5, 5.3)
2-H*H*	2.50 (dd, 15.6, 7.6)	2.55 (dd, 15.6, 7.4)	2.52 (dd, 15.3, 7.6)
6-H	2.97 (ap.t, 9.1)	3.01 (dd, 9.1, 9.0)	2.99 (dd, 9.0, 9.0)
15-H	3.07 – 3.13 (m)	3.14 (m)	3.09 – 3.13 (m)
7-H	3.51 (dd, 9.1, 6.6)	3.55 (dd, 9.1, 6.5)	3.53 (dd, 9.2, 7.0)
5-H	3.53 – 3.57 (m)	3.57 (m)	3.56 (ddd, 11.5, 8.8, 5.0)
18-H	3.83 (dd, 11.1, 3.3)	3.87 (dd, 10.6, 3.0)	3.85 (dd, 10.8, 2.9)
3-H	3.85 – 3.89 (m)	3.91 (m)	3.87 – 3.90 (m)
22-H	4.08 – 4.11 (m)	4.13 (m)	4.11 (br s)
13-H	5.16 (ddd, 15.2, 8.7, 1.3)	5.20 (ddd, 15.3 8.8,1.2)	5.18 (ddd, 15.3, 8.8, 1.1)
16-H	5.26 (dq, 9.0, 1.3)	5.29 (dq, 9.0, 1.3)	5.27 (dq, 9.0, 1.1)
9-H	5.38 (dd, 15.4, 8.6)	5.42 (dd, 15.4, 8.4)	5.39 (dd. 15.4, 8.7)
14-H	5.46 (dd, 15.2, 6.5)	5.50 (dd, 15.3, 6.4)	5.48 (dd, 15.2, 6.5)
8-H	5.50 (dd, 15.4, 6.6)	5.54 (dd, 15.4, 6.5)	5.52 (dd, 15.4, 6.6)
20-H	5.57 – 5.61 (m)	5.63 (m)	5.60, 5.61 (m)

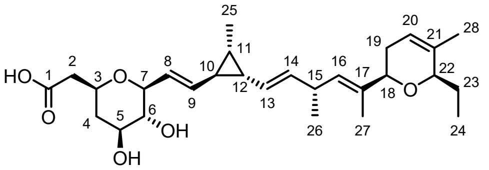

4

Table S6. Comparison of ^{13}C-NMR data between synthetic and natural ambruticin S.[10–12]

Position	Synthetic Ambruticin S 126 MHz (CD_3OD): δ_C	Natural Ambruticin S 75.5 MHz (CD_3OD): δ_C	Hanessian's Synthetic Ambrutícin S 125 MHz (CD_3OD): δ_C
C-24	8.7	8.6	8.7
C-27	12.6	12.6	12.6
C-25	13.4	13.3	13.4
C-28	19.1	19.0	19.1
C-26	21.7	21.6	21.7
C-11	22.3	22.2	22.3
C-23	26.6	26.5	26.6
C-12	29.8	29.7	29.8
C-19	31.1	31.0	31.1
C-10	31.7	31.6	31.7
C-15	36.3	36.2	36.3
C-4	40.2	40.1	40.2
C-2	41.6	41.5	41.6
C-3	73.3	73.2	73.3

C-5	73.4	73.3	73.3
C-6	77.1	77.0	77.0
C-18	79.5	79.4	79.4
C-22	79.6	79.4	79.5
C-7	81.8	81.4	81.8
C-20	122.1	122.0	122.1
C-8	126.2	126.1	126.1
C-13	126.9	126.8	126.9
C-16	130.9	130.8	130.9
C-21	136.1	136.0	136.0
C-17	136.2	136.1	136.2
C-14	136.3	136.2	136.2
C-9	138.2	138.0	138.2
C-1	174.7	174.6	174.8

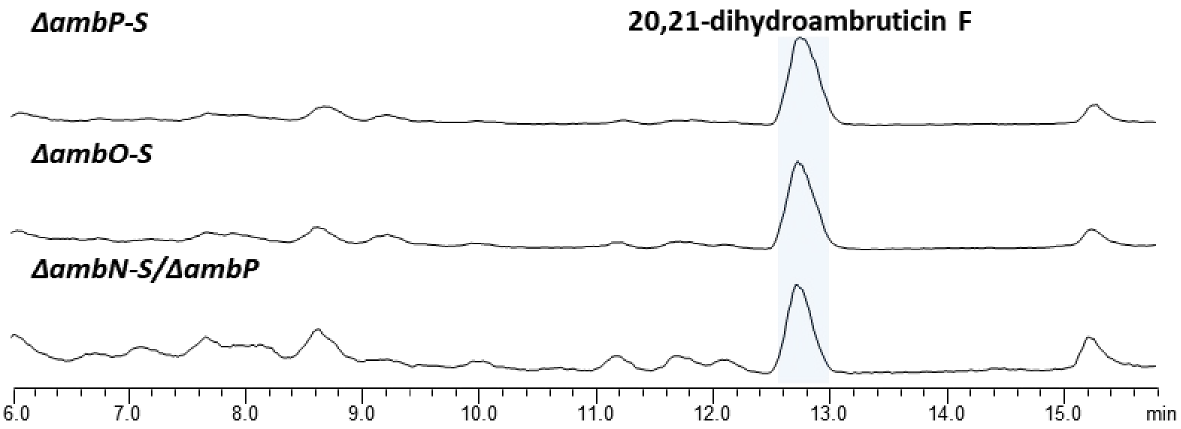

Figure S6. HPLC traces of the *ΔambP-S*, *ΔambO-S* and *ΔambN-S/ΔambP* mutants of *S. cellulosum* So ce10.

7. References

1. Tautz, T.; Hoffmann, J.; Hoffmann, T.; Steinmetz, H.; Washausen, P.; Kunze, B.; Huch, V.; Kitsche, A.; Reichenbach, H.; Höfle, G.; Müller, R.; Kalesse, M. *Org. Lett.* **2016**, *18*, 2560–2563.
2. Rychnovsky, S. D.; Rogers, B.; Yang, G. *J. Org. Chem.* **1993**, *58*, 3511–3515.
3. Evans, D. A.; Rieger, D. L.; Gage, J. R. *Tetrahedron Lett.* **1990**, *31*, 7099–7100.
4. Bowen, J. I.; Wang, L.; Crump, M. P.; Willis, C. L. *Org. Biomol. Chem.* **2021**, *19*, 6210–6215.

5. Lee, E.; Choi, S. J.; Kim, H.; Han, H. O.; Kim, Y. K.; Min, S. J.; Son, S. H.; Lim, S. M.; Jang, W. S. *Angew. Chemie* **2002**, *41*, 176-178.

6. Pospíšil, J.; Markó, I. E. *J. Am. Chem. Soc.* **2007**, *129*, 3516–3517

7. Lindner, F.; Friedrich, S.; Hahn, F. *J. Org. Chem.* **2018**, *83*, 14091–14101.

8. Bates, R. B.; Gangwar, S. *Tetrahedron: Asymmetry* **1993**, *4*, 69–72.

9. Fields, A. M.; Jones, S. *Tetrahedron* **2019**, *75*, 3413–3420.

10. Hanessian, S.; Focken, T.; Mi, X.; Oza, R.; Chen, B.; Ritson, D.; Beaudegnies, R. *J. Org. Chem.* **2010**, *75*, 5601–5618.

11. Liu, P.; Jacobsen, E. N. *J. Am. Chem. Soc.* **2001**, *123*, 10772–10773.

12. Connor, D. T.; Greenough, R. C.; Von Strandtmann, M. *J. Org. Chem.* **1977**, *42*, 3664–3669.

8. NMR Appendix

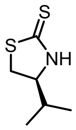

S1

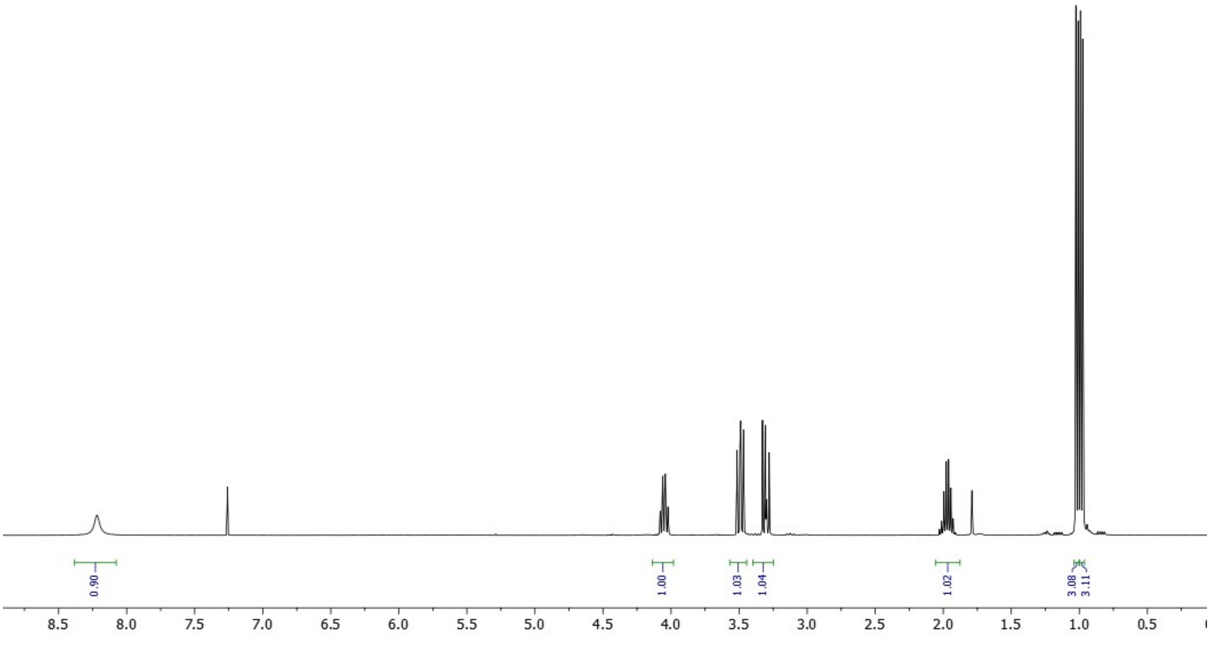

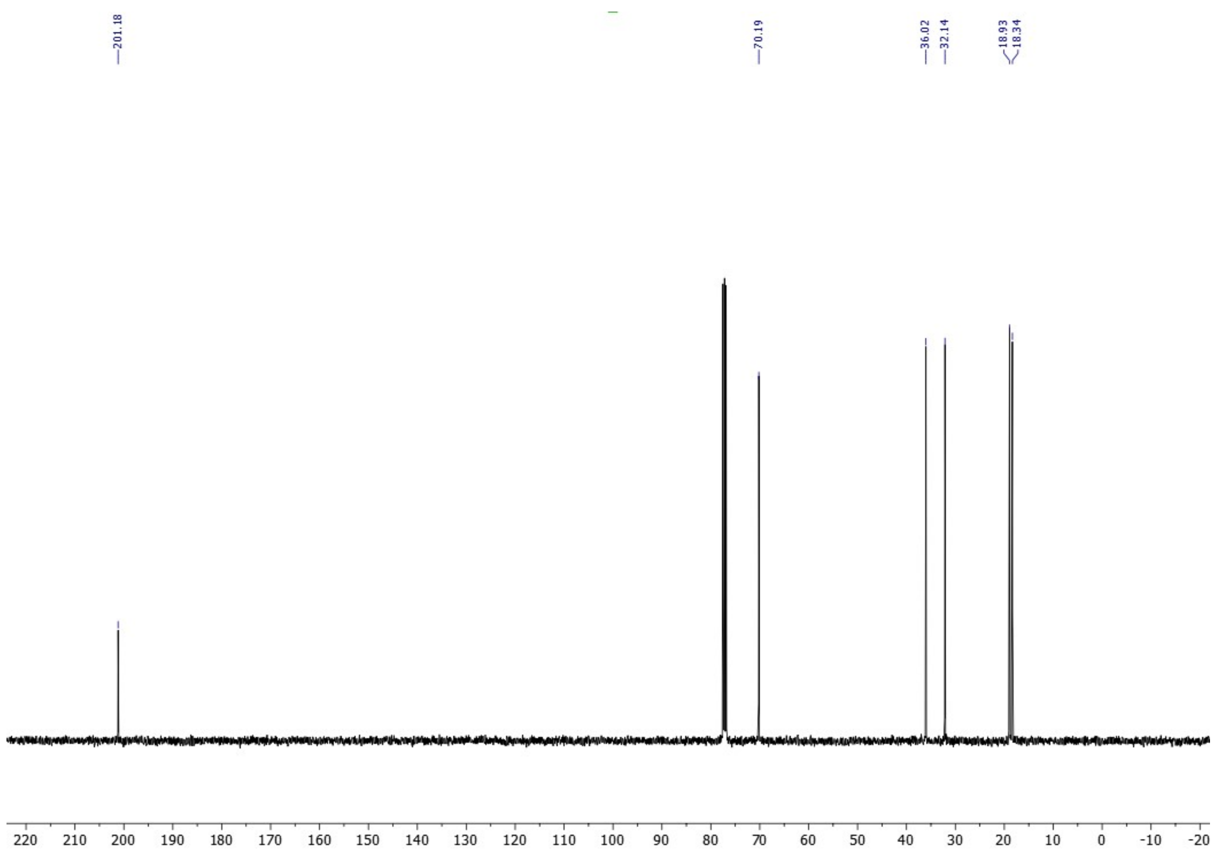
201.18
70.19
36.02
32.14
18.93
18.34
220 210 200 190 180 170 160 150 140 130 120 110 100 90 80 70 60 50 40 30 20 10 0 -10 -20

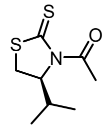
S
S
N
O
15

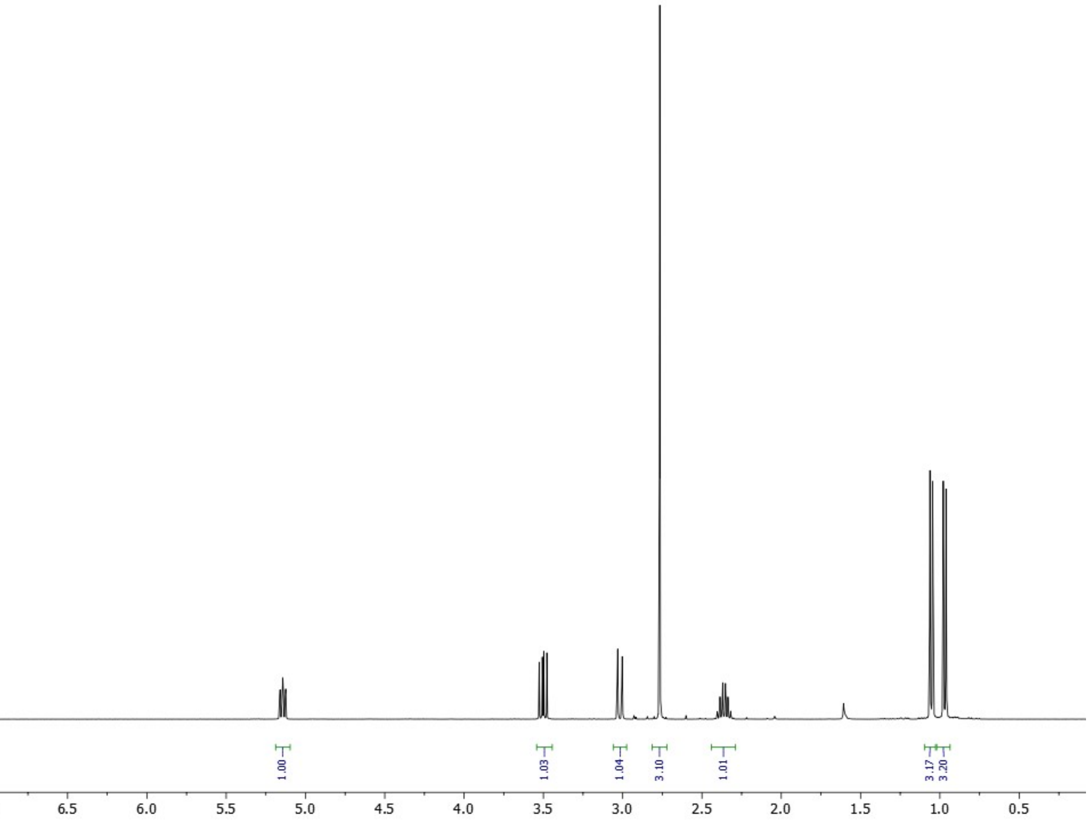
1.00
1.03
1.04
3.10
1.01
3.17
3.20
7.5 7.0 6.5 6.0 5.5 5.0 4.5 4.0 3.5 3.0 2.5 2.0 1.5 1.0 0.5

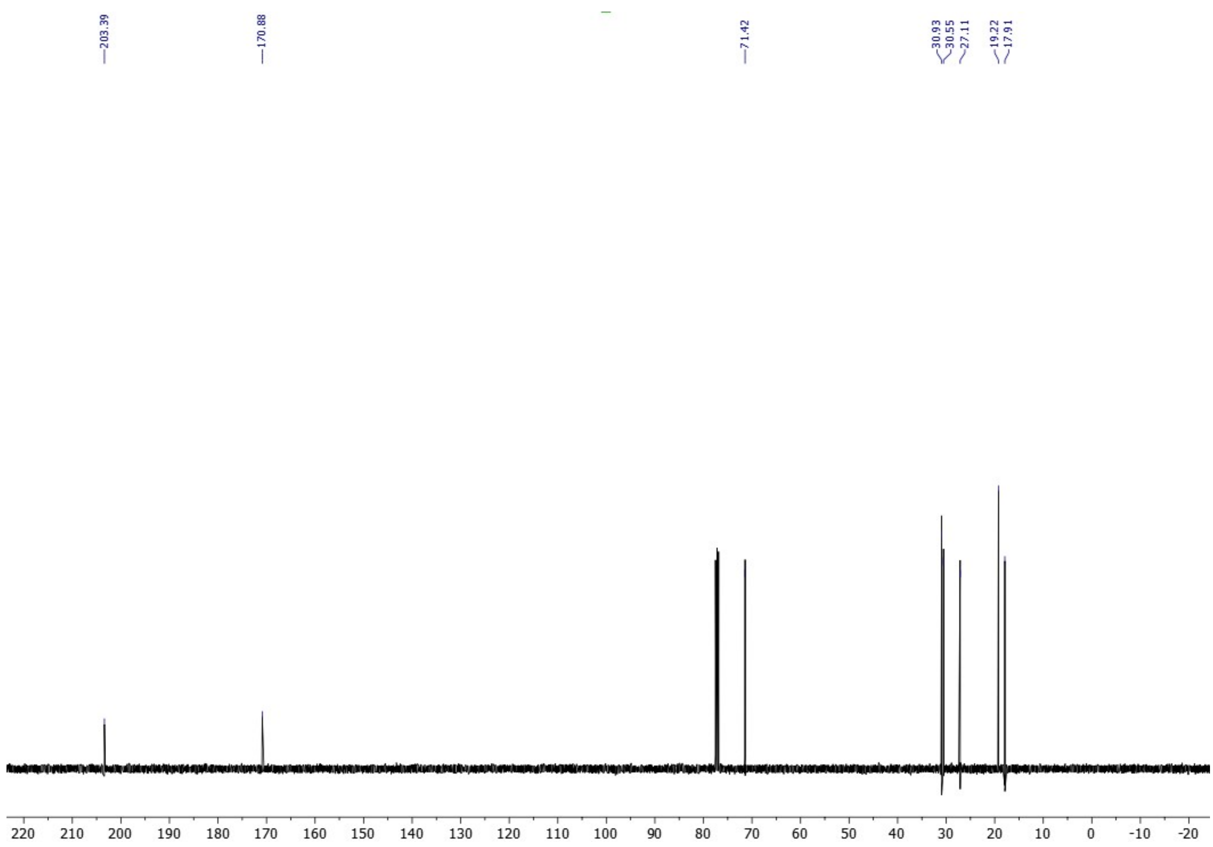

203.39
170.88
71.42
30.93
30.55
27.11
19.22
17.91
220 210 200 190 180 170 160 150 140 130 120 110 100 90 80 70 60 50 40 30 20 10 0 -10 -20

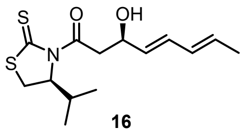
S
O
OH
N
S

16

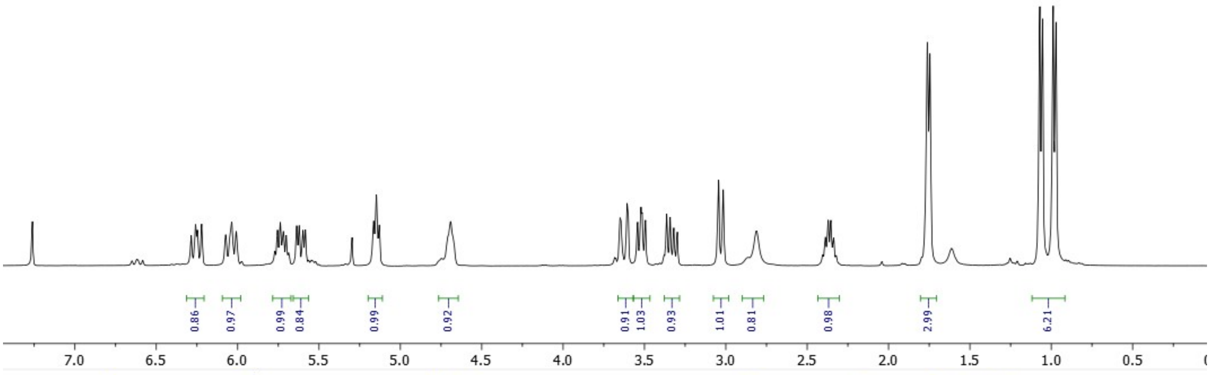
0.86
0.97
0.99
0.84
0.99
0.92
0.91
1.03
0.93
1.01
0.81
0.98
2.99
6.21
7.0
6.5
6.0
5.5
5.0
4.5
4.0
3.5
3.0
2.5
2.0
1.5
1.0
0.5

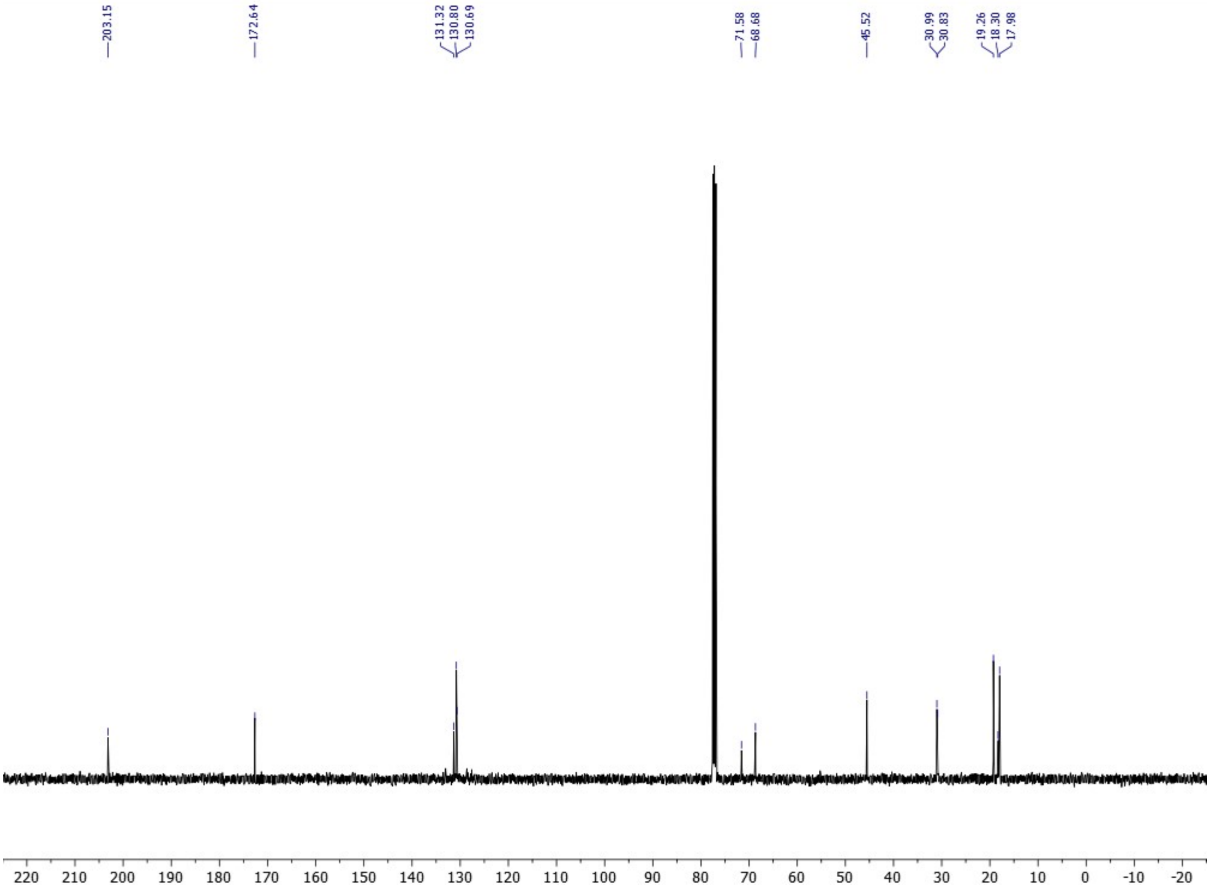
203.15
172.64
131.32
130.80
130.69
71.58
68.68
45.52
30.99
30.83
19.26
18.30
17.98
220
210
200
190
180
170
160
150
140
130
120
110
100
90
80
70
60
50
40
30
20
10
0
-10
-20

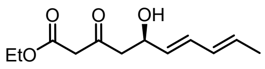
O
O
OH
EtO

17

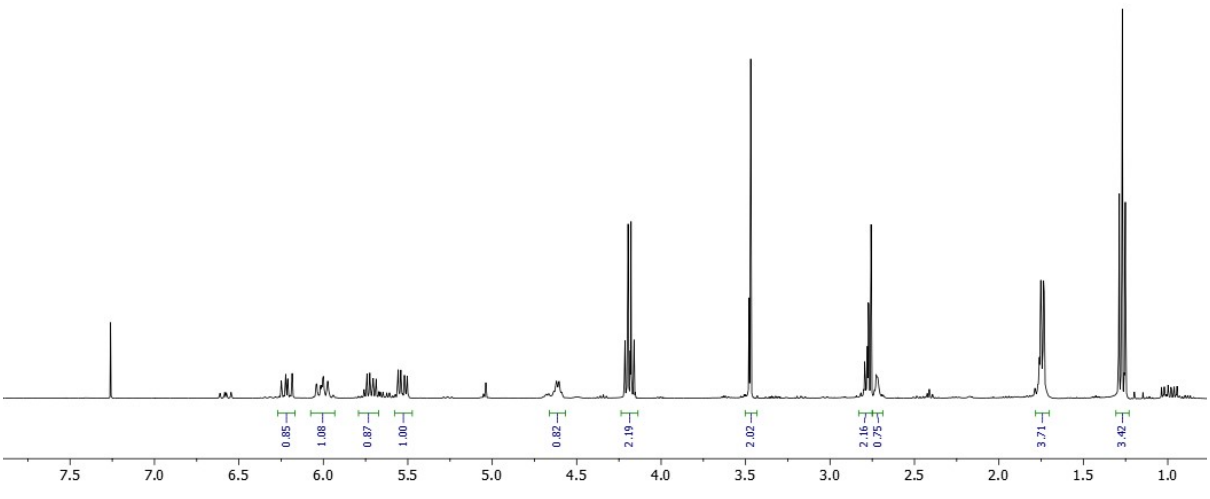
0.85
1.08
0.87
1.00
0.82
2.19
2.02
2.16
0.75
3.71
3.42
7.5 7.0 6.5 6.0 5.5 5.0 4.5 4.0 3.5 3.0 2.5 2.0 1.5 1.0

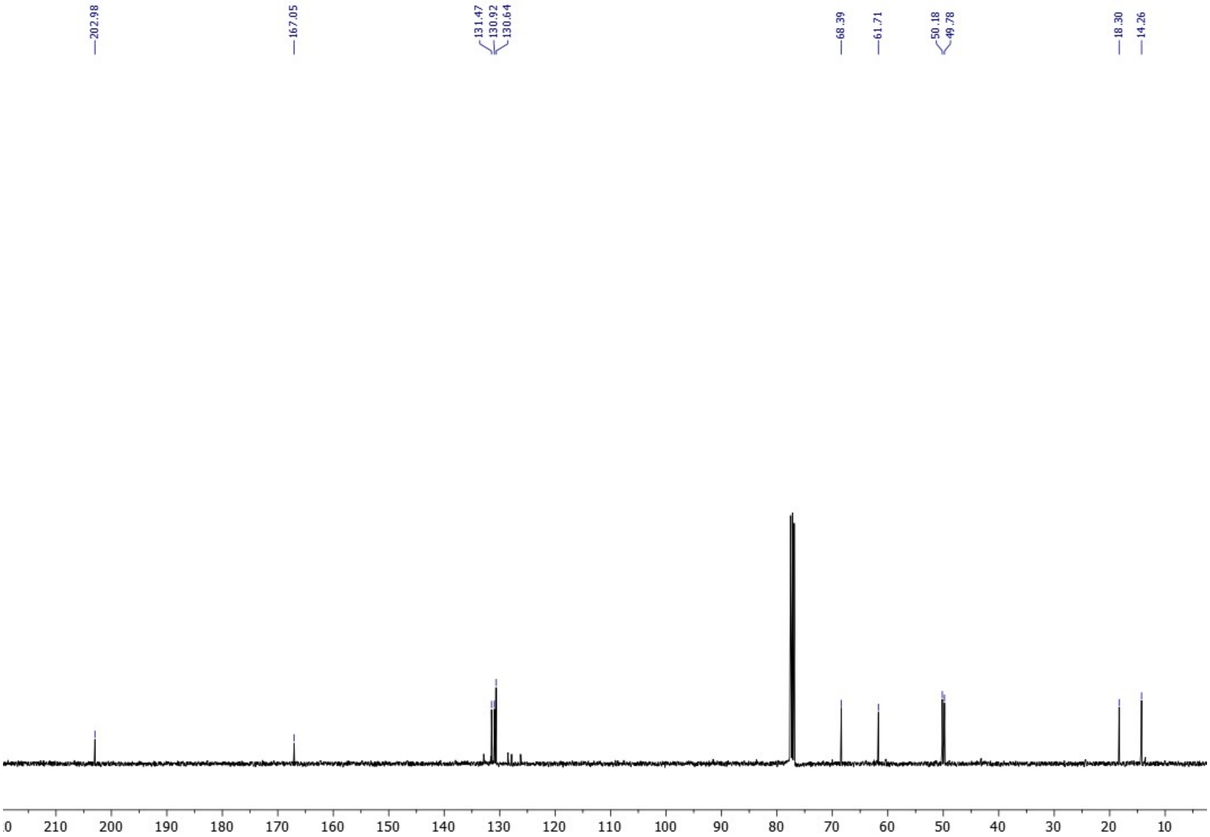
202.98
167.05
131.47
130.92
130.64
68.39
61.71
50.18
49.78
18.30
14.26
0 210 200 190 180 170 160 150 140 130 120 110 100 90 80 70 60 50 40 30 20 10

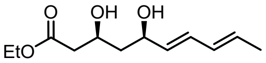

O
OH
OH
EtO

18

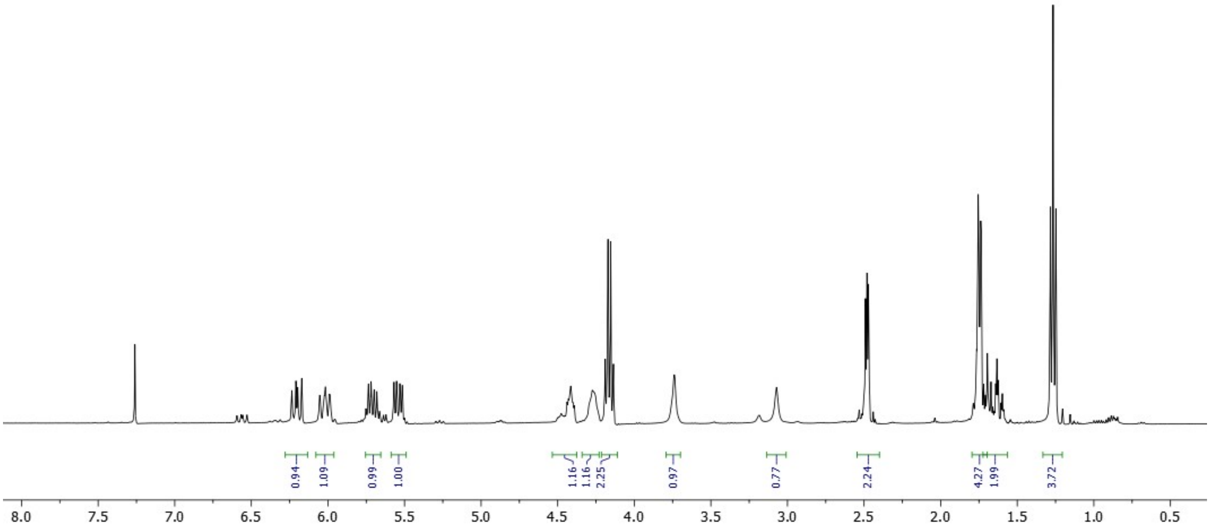

0.94
1.09
0.99
1.00
1.16
1.16
2.25
0.97
0.77
2.24
4.27
1.99
3.72
8.0
7.5
7.0
6.5
6.0
5.5
5.0
4.5
4.0
3.5
3.0
2.5
2.0
1.5
1.0
0.5

Z

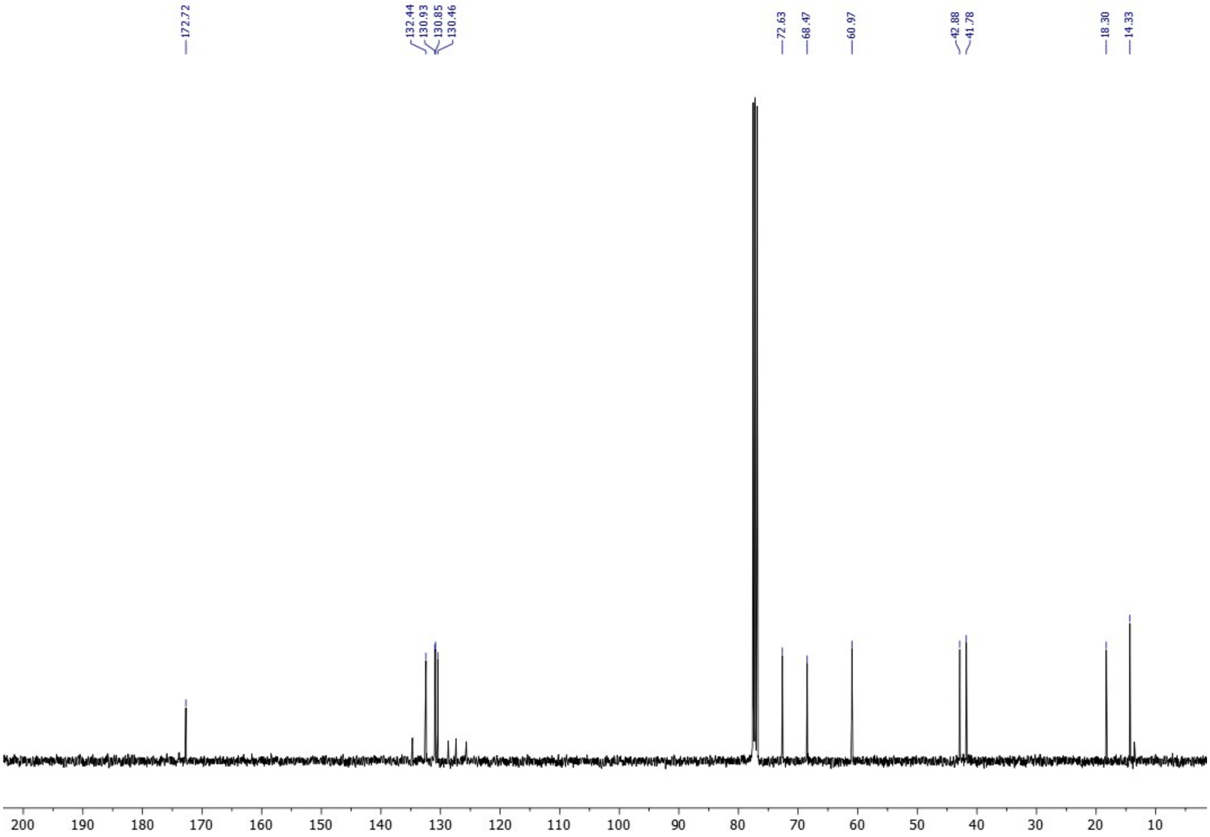

172.72
132.44
130.93
130.85
130.46
72.63
68.47
60.97
42.88
41.78
18.30
14.33
200
190
180
170
160
150
140
130
120
110
100
90
80
70
60
50
40
30
20
10

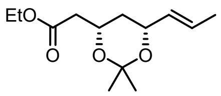

S2

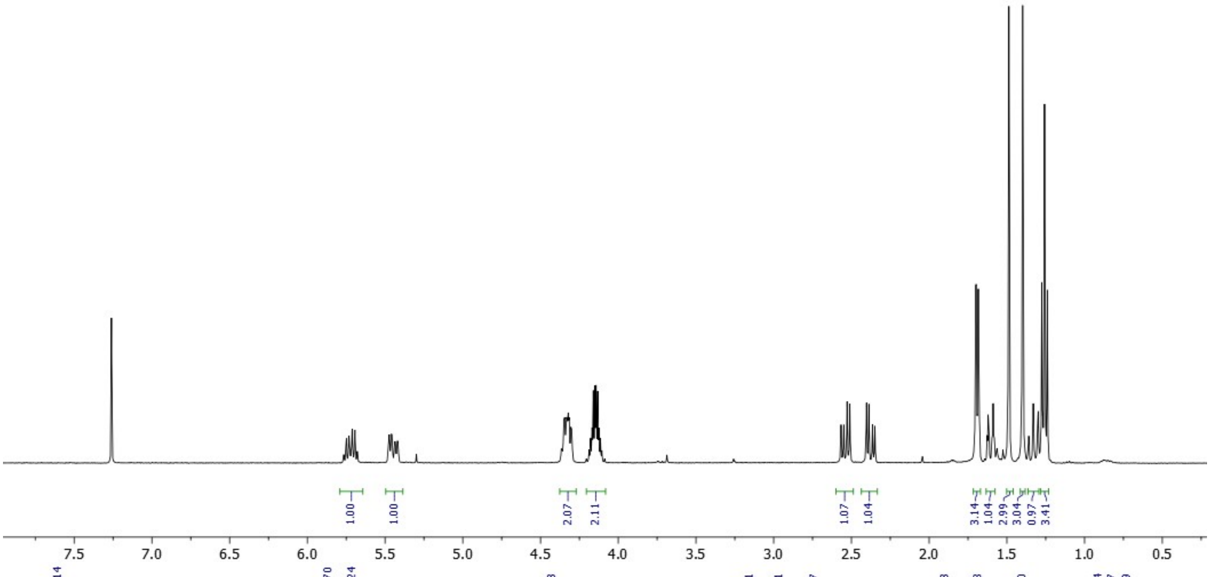

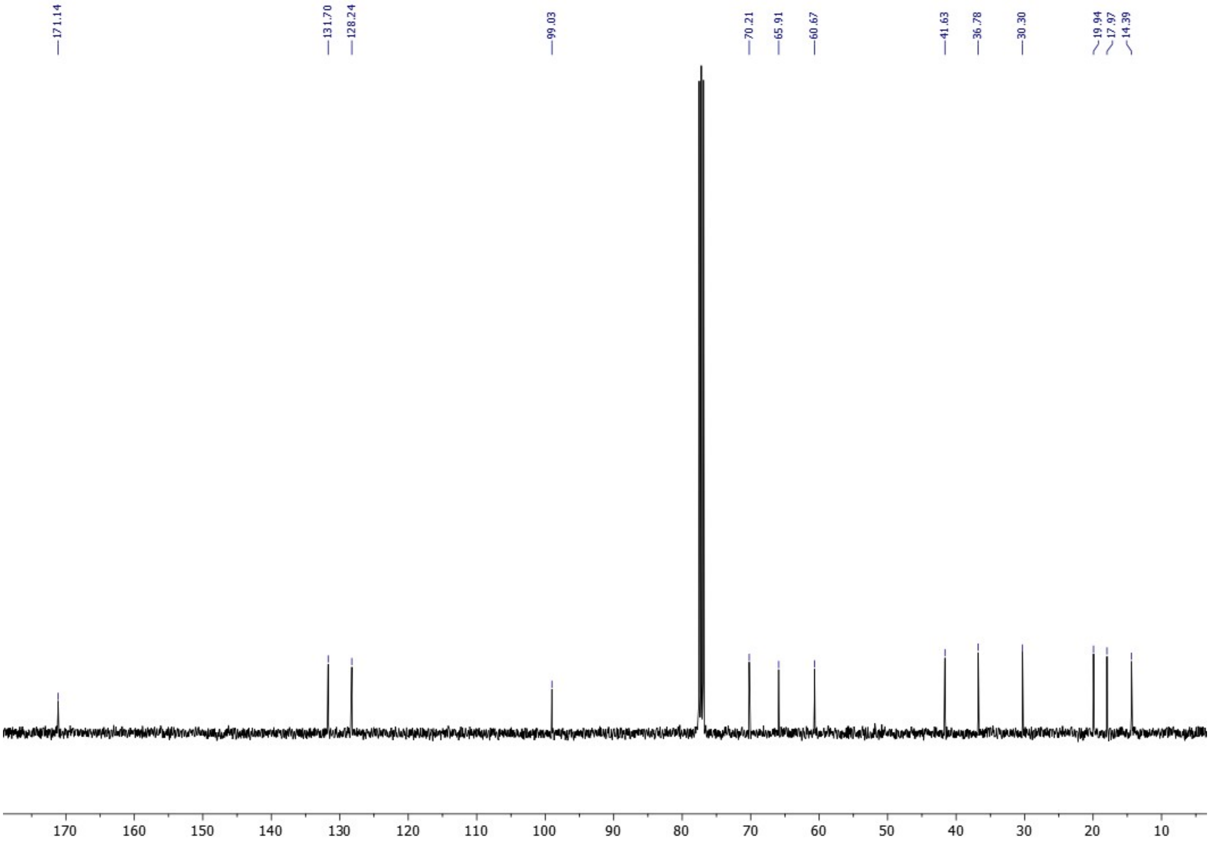

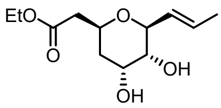
EtO
O
OH
OH

19

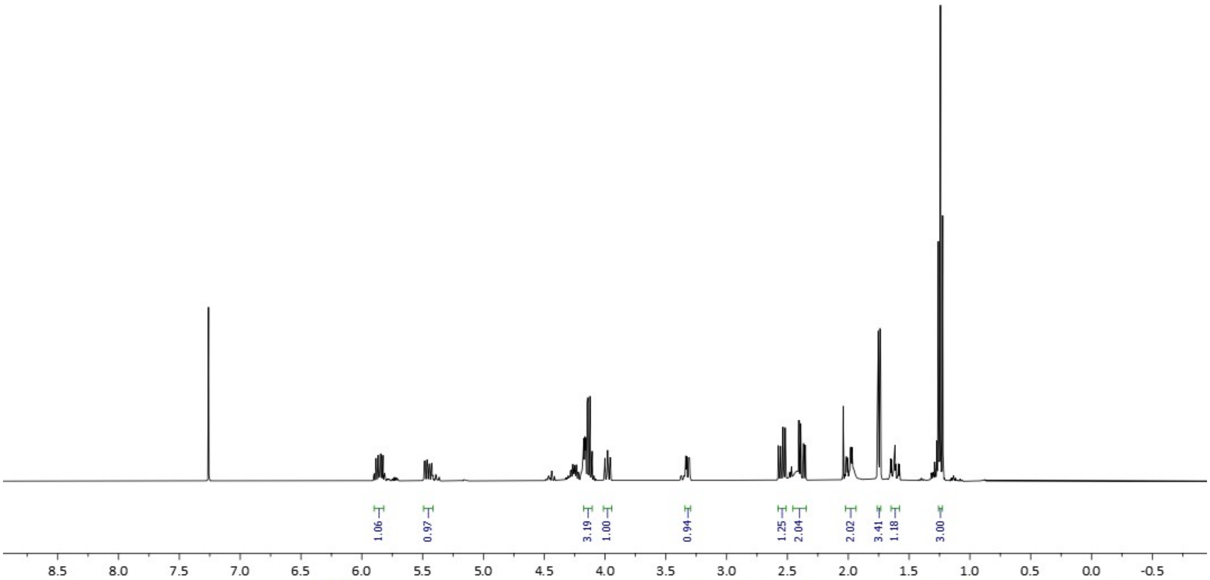
1.06
0.97
3.19
1.00
0.94
1.25
2.04
2.02
3.41
1.18
3.00
8.5
8.0
7.5
7.0
6.5
6.0
5.5
5.0
4.5
4.0
3.5
3.0
2.5
2.0
1.5
1.0
0.5
0.0
-0.5

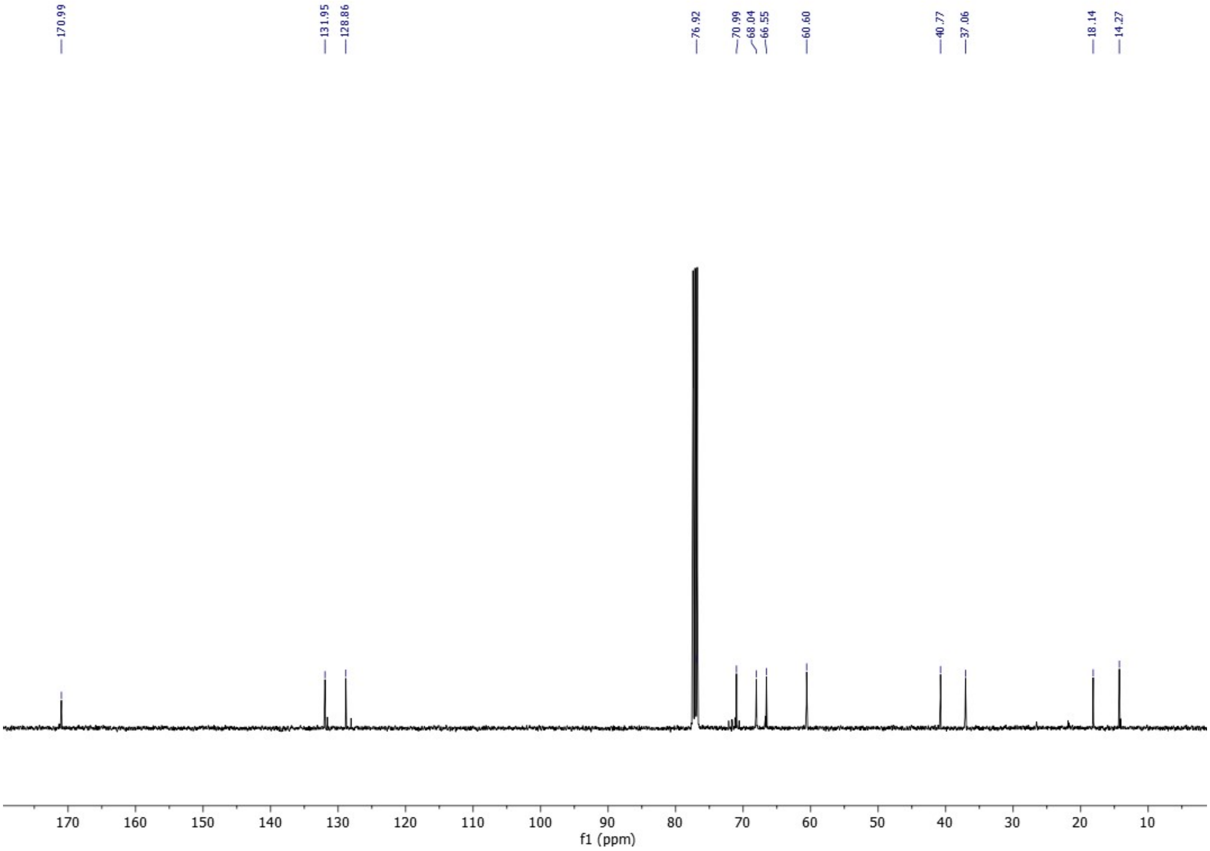
170.99
131.95
128.86
76.92
70.99
68.04
66.55
60.60
40.77
37.06
18.14
14.27
170
160
150
140
130
120
110
100
90
80
70
60
50
40
30
20
10
f1 (ppm)

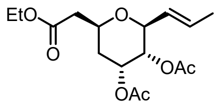
EtO
O
O
OAc
OAc

S3

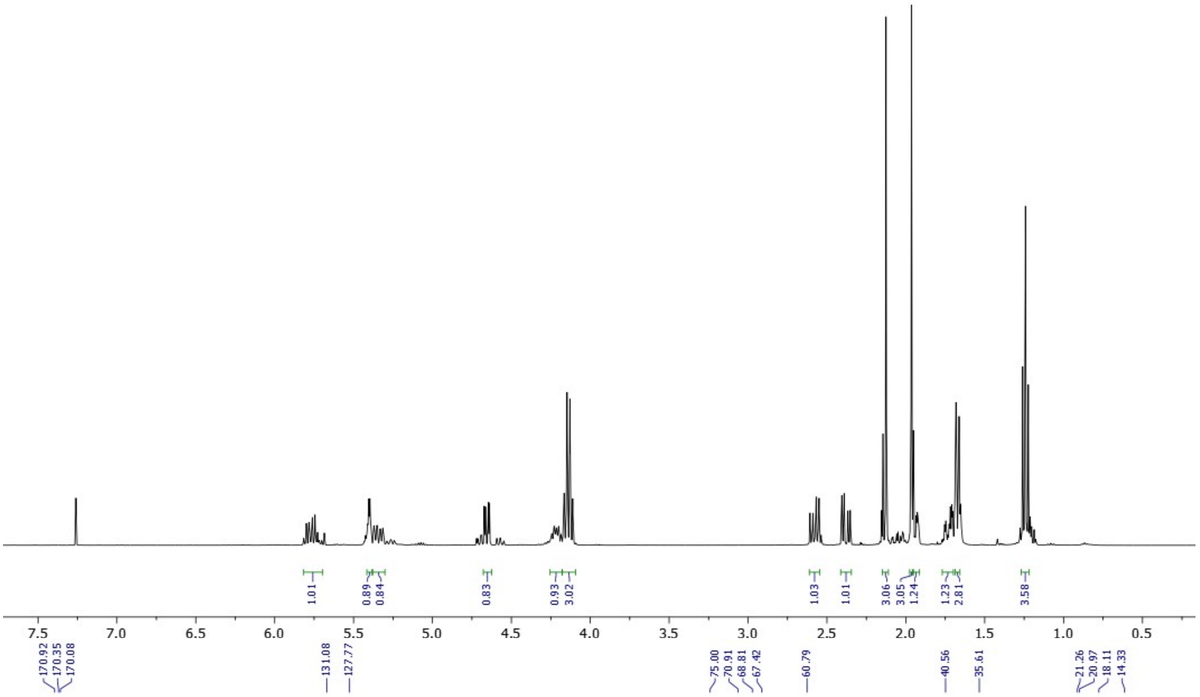
1.01
0.89
0.84
0.83
0.93
3.02
1.03
1.01
3.06
3.05
1.24
1.23
2.81
3.58
7.5 7.0 6.5 6.0 5.5 5.0 4.5 4.0 3.5 3.0 2.5 2.0 1.5 1.0 0.5

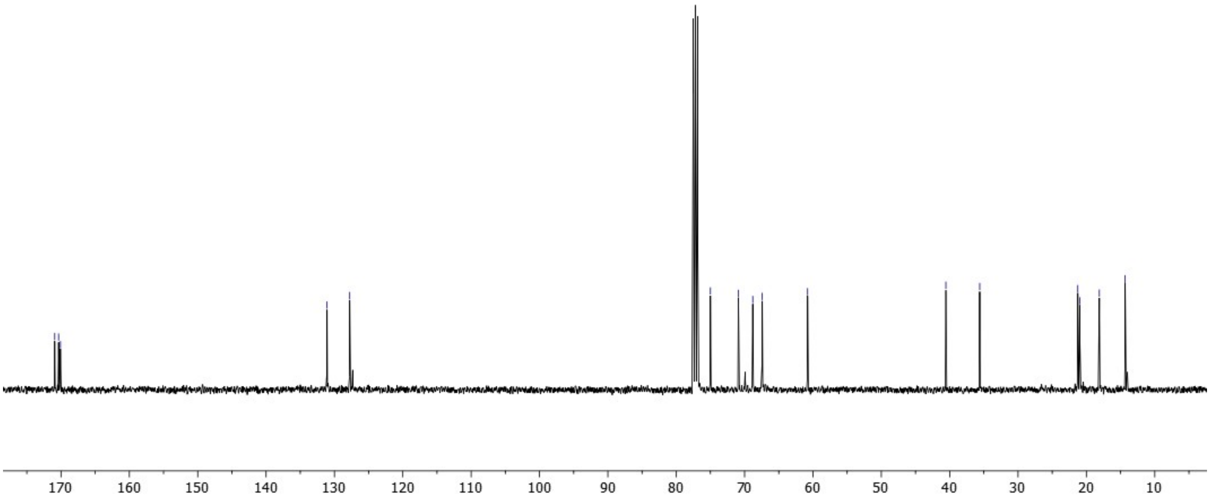
170.92
170.35
170.08
131.08
127.77
75.00
70.91
68.81
67.42
60.79
40.56
35.61
21.26
20.97
18.11
14.33
170 160 150 140 130 120 110 100 90 80 70 60 50 40 30 20 10

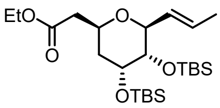
EtO
O
O
OTBS
OTBS

S4

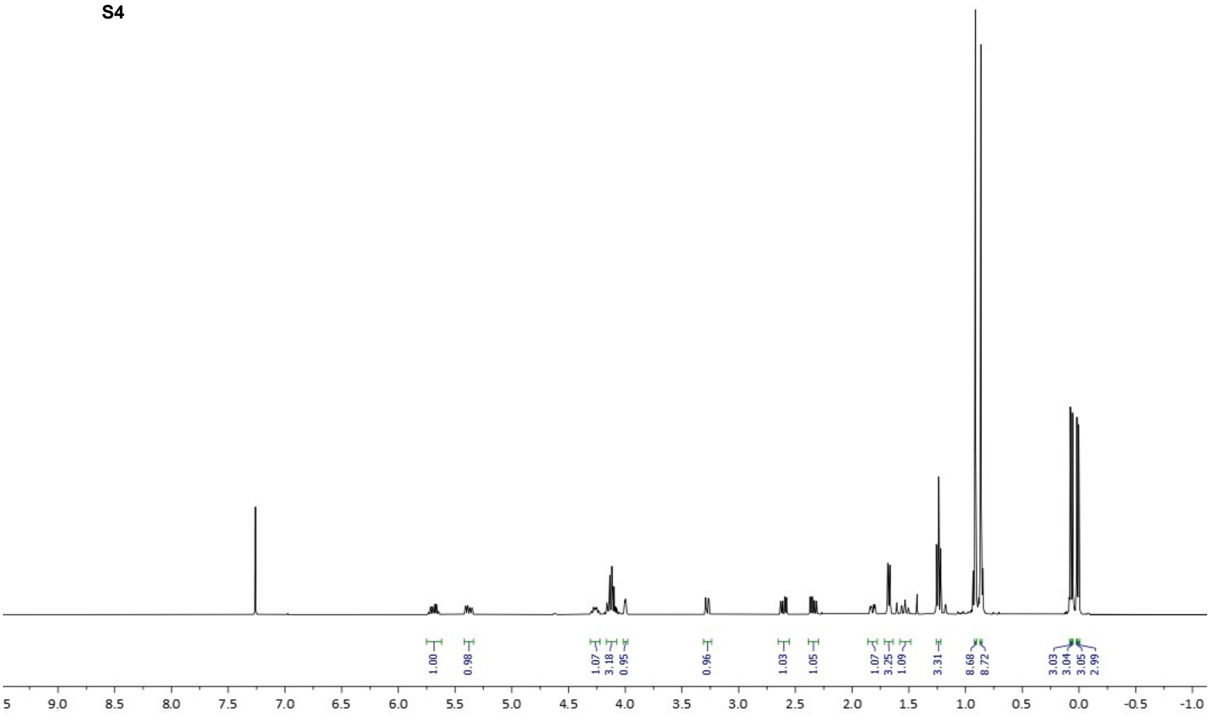
1.00
0.98
1.07
3.18
0.95
0.96
1.03
1.05
1.07
3.25
1.09
3.31
8.68
8.72
3.03
3.04
3.05
2.99
9.0
8.5
8.0
7.5
7.0
6.5
6.0
5.5
5.0
4.5
4.0
3.5
3.0
2.5
2.0
1.5
1.0
0.5
0.0
-0.5
-1.0

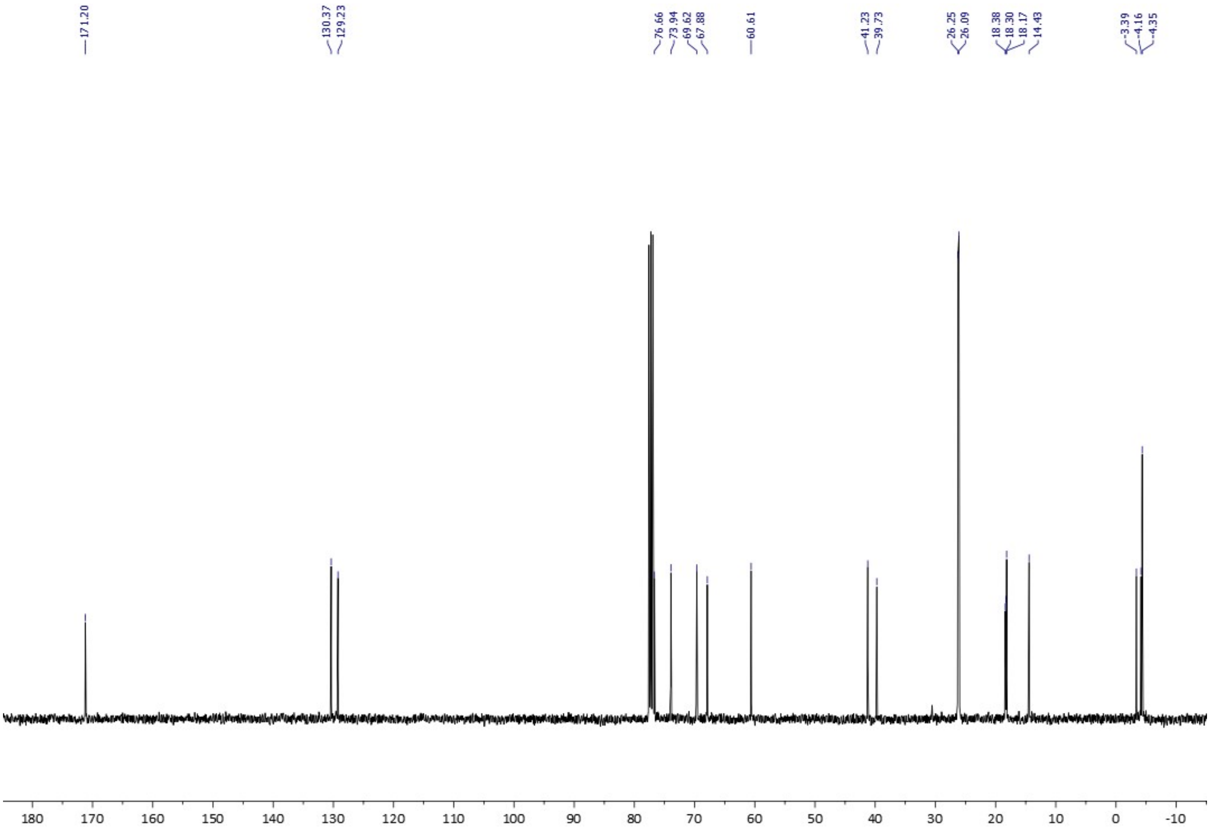
171.20
130.37
129.23
76.66
73.94
69.62
67.88
60.61
41.23
39.73
26.25
26.09
18.38
18.30
18.17
14.43
-3.39
-4.16
-4.35
180
170
160
150
140
130
120
110
100
90
80
70
60
50
40
30
20
10
0
-10

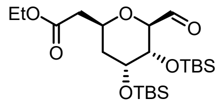
EtO
O
O
O
OTBS
OTBS

12

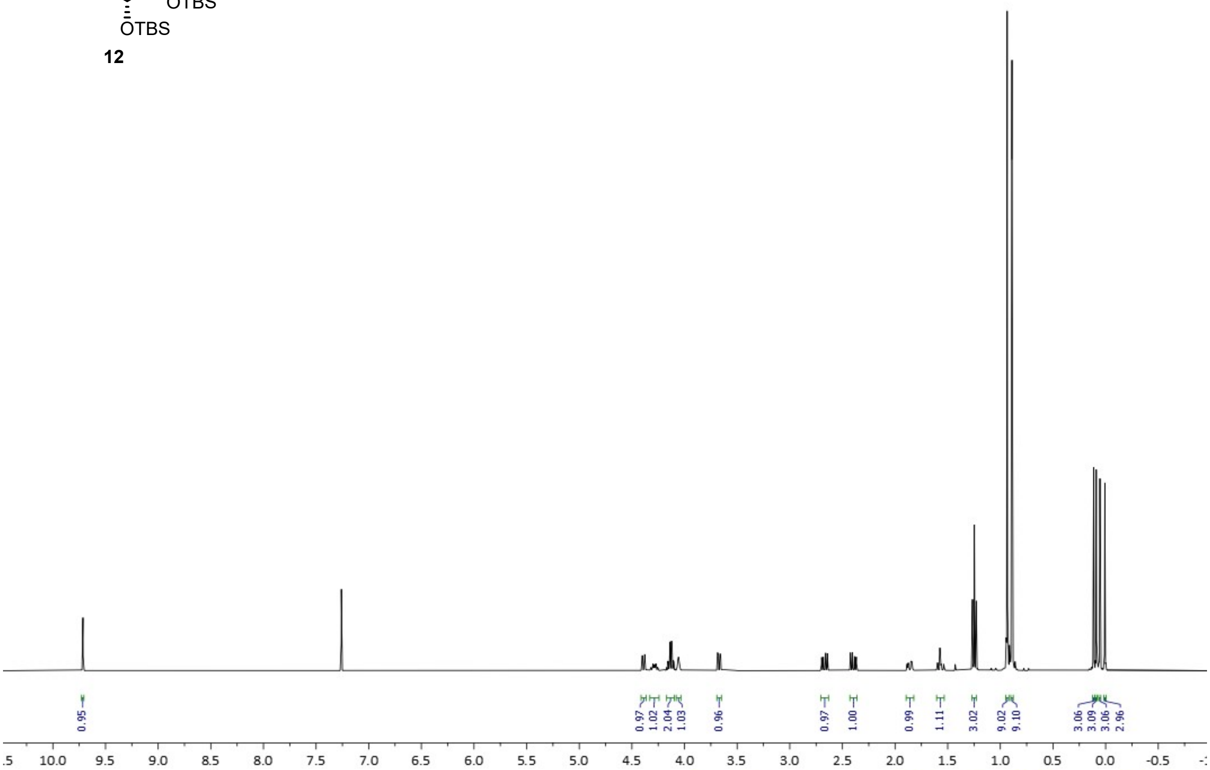
0.95
0.97
1.02
2.04
1.03
0.96
0.97
1.00
0.99
1.11
3.02
9.02
9.10
3.06
3.09
3.06
2.96
10.0 9.5 9.0 8.5 8.0 7.5 7.0 6.5 6.0 5.5 5.0 4.5 4.0 3.5 3.0 2.5 2.0 1.5 1.0 0.5 0.0 -0.5

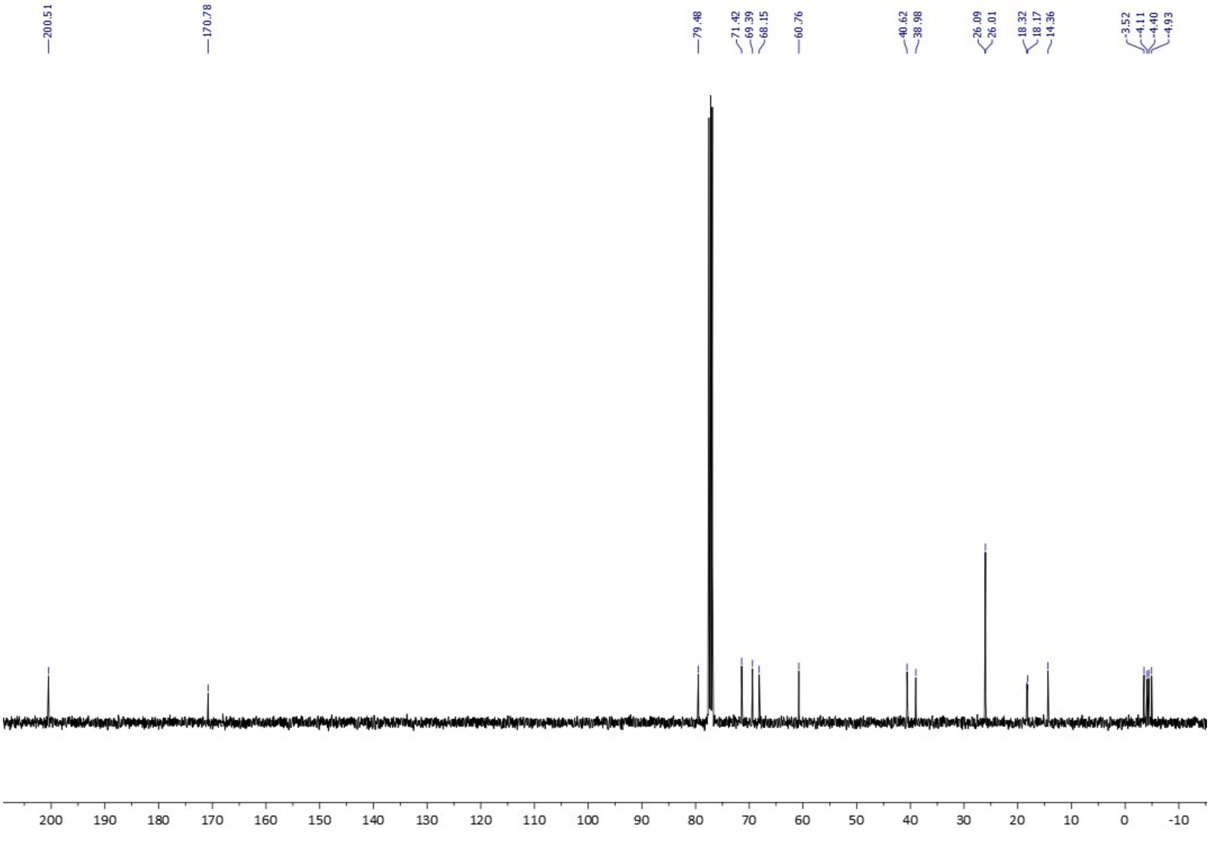
200.51
170.78
79.48
71.42
69.39
68.15
60.76
40.62
38.98
26.09
26.01
18.32
18.17
14.36
-3.52
-4.11
-4.40
-4.93
200 190 180 170 160 150 140 130 120 110 100 90 80 70 60 50 40 30 20 10 0 -10

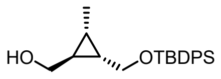
HO
OTBDPS

21

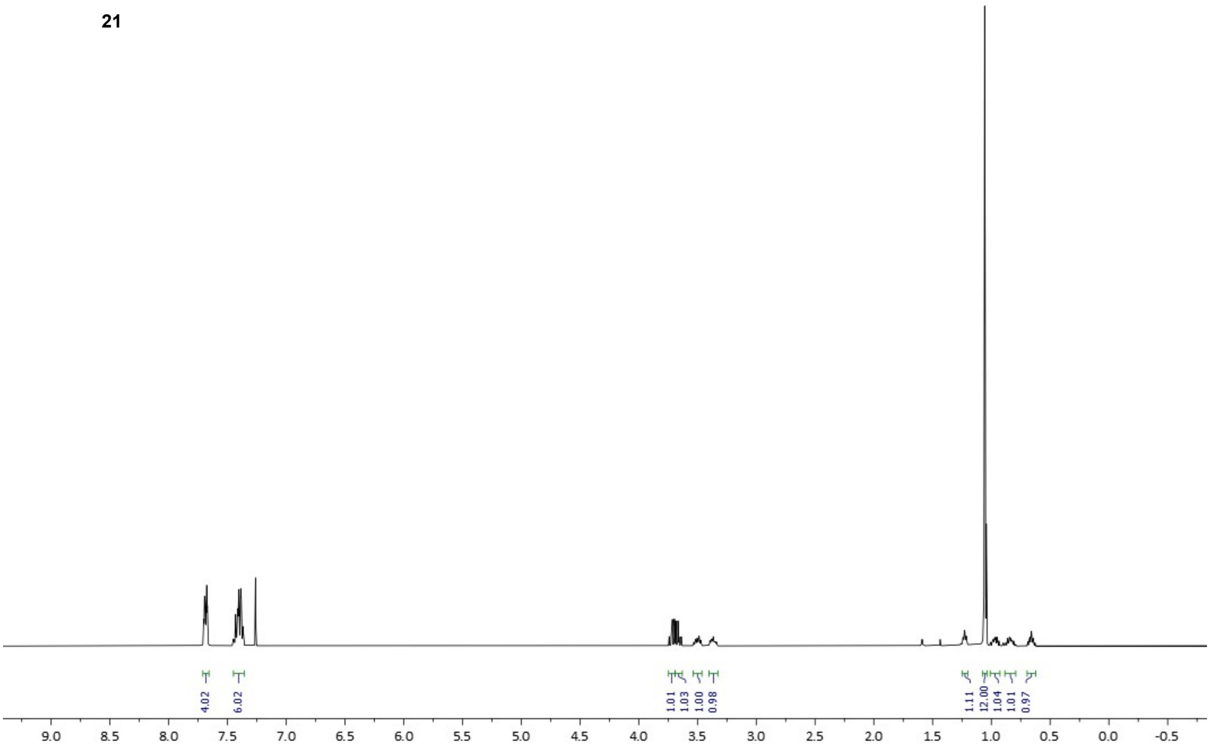
4.02
6.02
1.01
1.03
1.00
0.98
1.11
12.00
1.04
1.01
0.97
9.0 8.5 8.0 7.5 7.0 6.5 6.0 5.5 5.0 4.5 4.0 3.5 3.0 2.5 2.0 1.5 1.0 0.5 0.0 -0.5

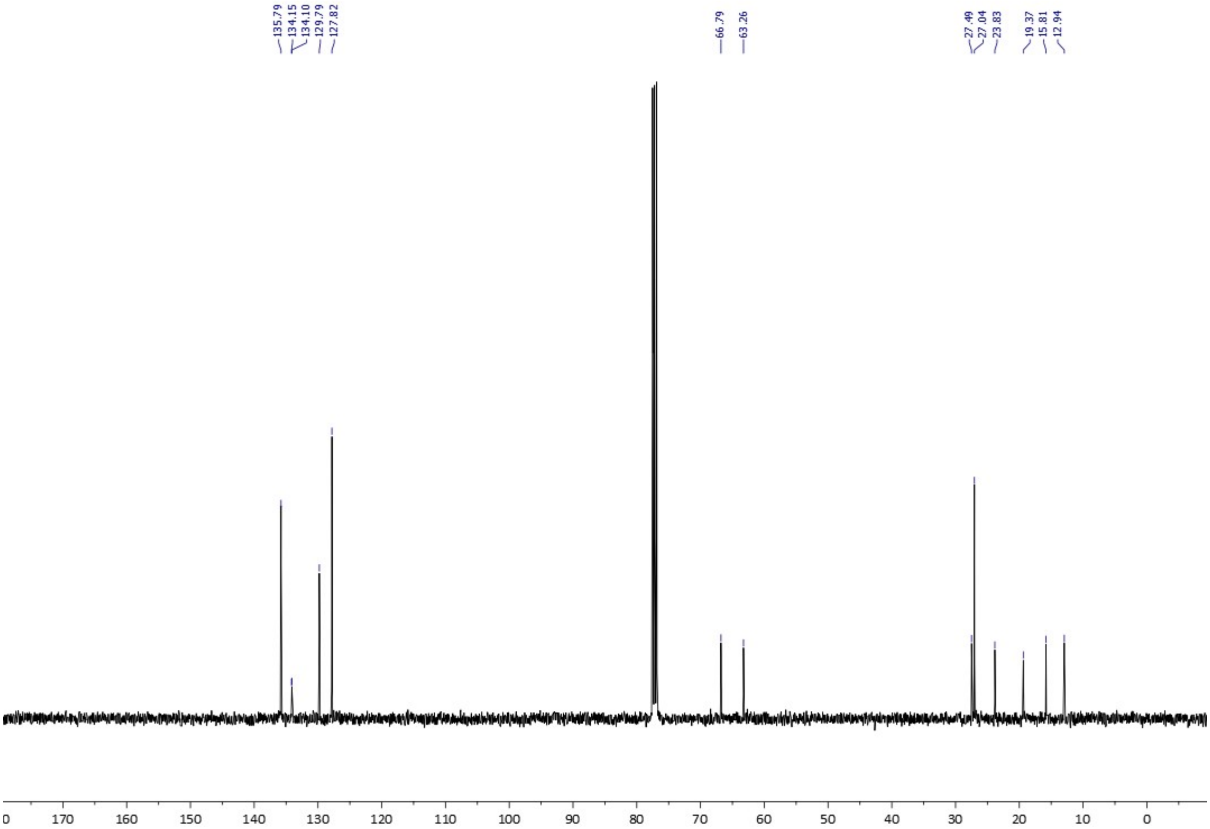
135.79
134.15
134.10
129.79
127.82
66.79
63.26
27.49
27.04
23.83
19.37
15.81
12.94
170 160 150 140 130 120 110 100 90 80 70 60 50 40 30 20 10 0

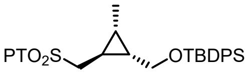
PTO2S
OTBDPS

13

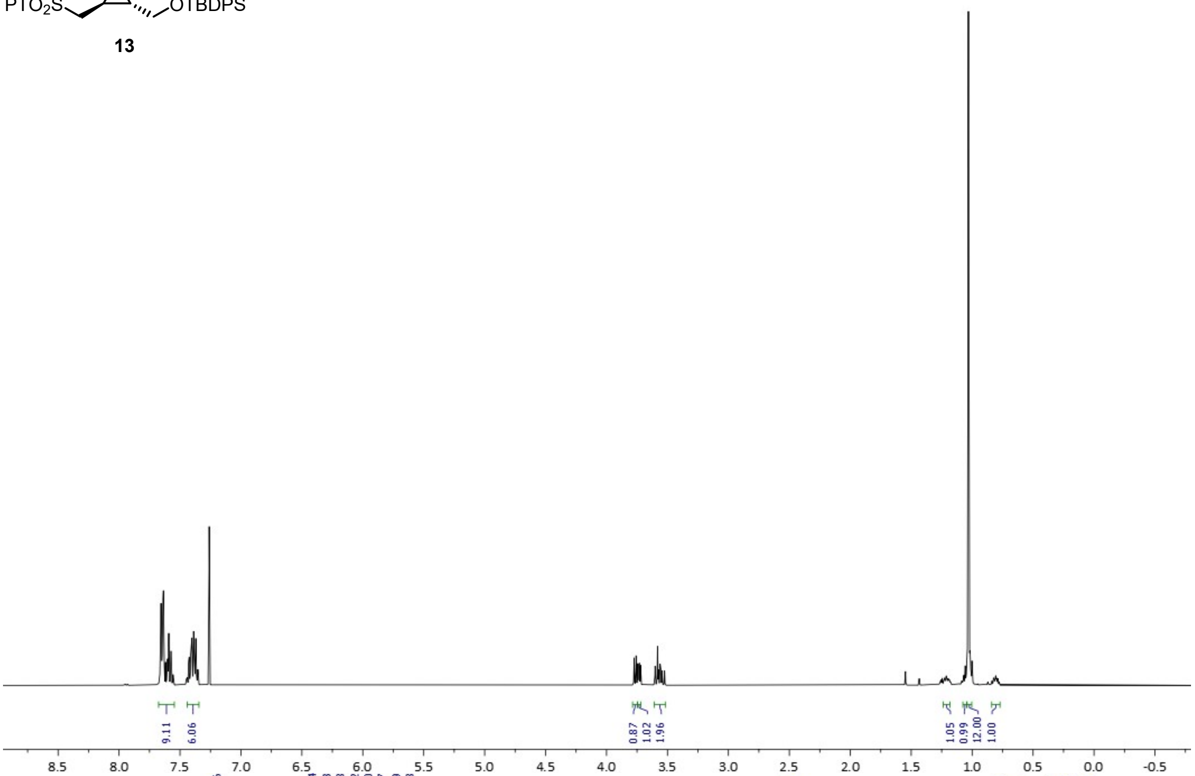
9.11
6.06
0.87
1.02
1.96
1.05
0.99
12.00
1.00
8.5
8.0
7.5
7.0
6.5
6.0
5.5
5.0
4.5
4.0
3.5
3.0
2.5
2.0
1.5
1.0
0.5
0.0
-0.5

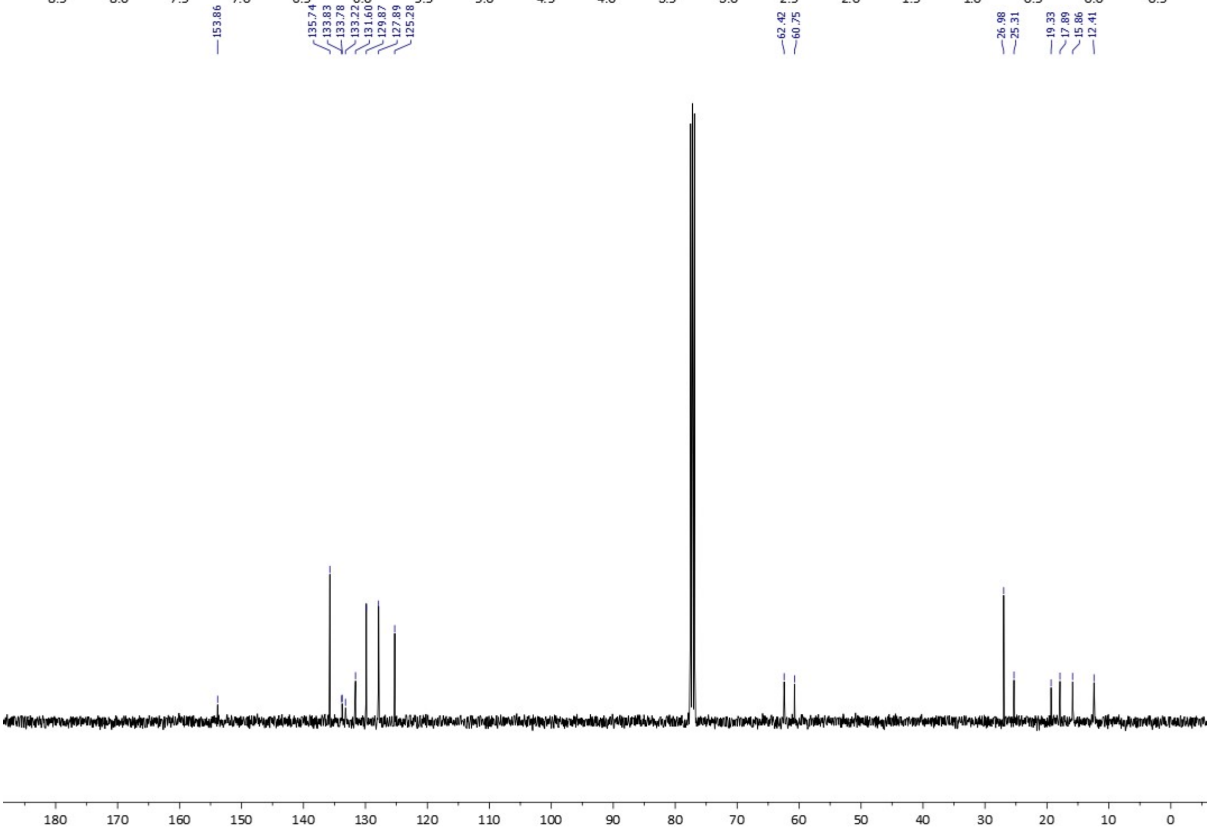
153.86
135.74
133.83
133.78
133.22
131.60
129.87
127.89
125.28
62.42
60.75
26.98
25.31
19.33
17.89
15.86
12.41
180
170
160
150
140
130
120
110
100
90
80
70
60
50
40
30
20
10
0

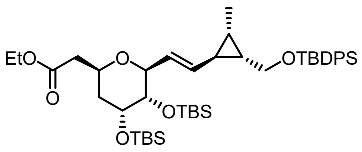

22

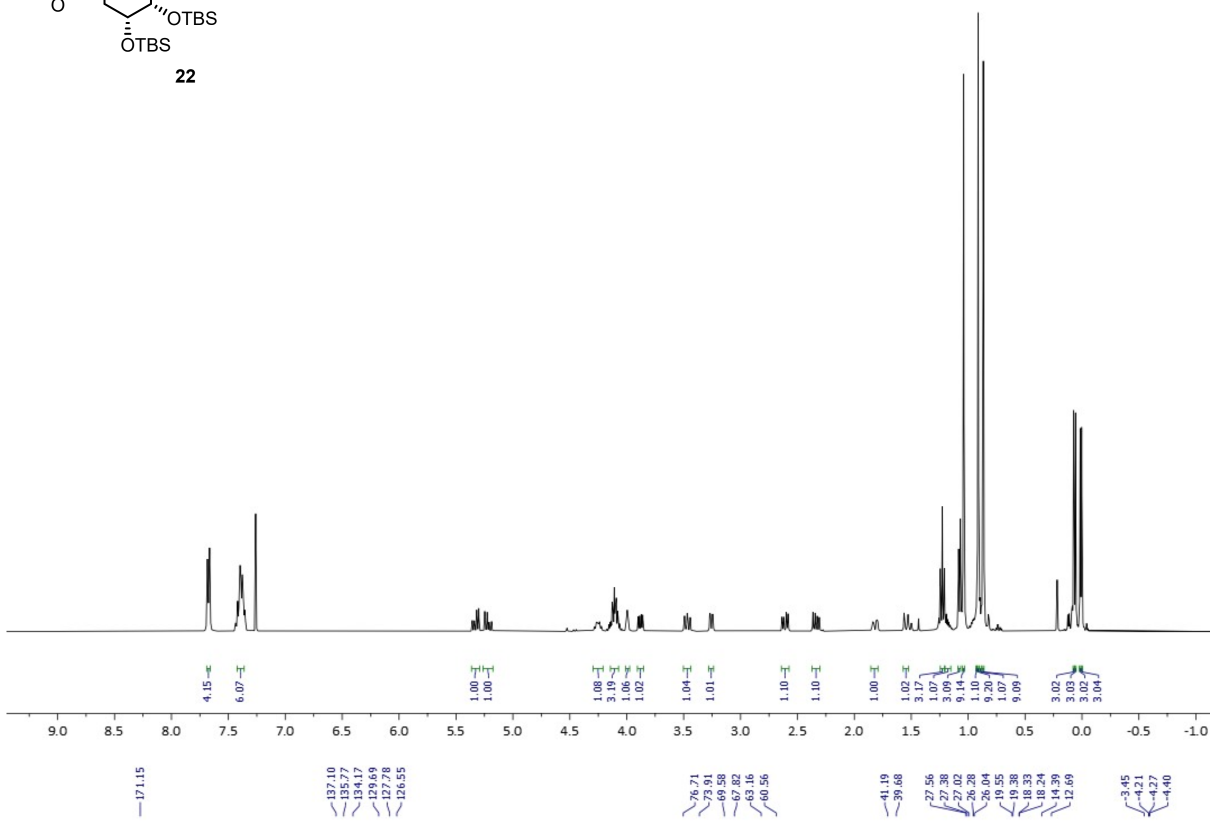

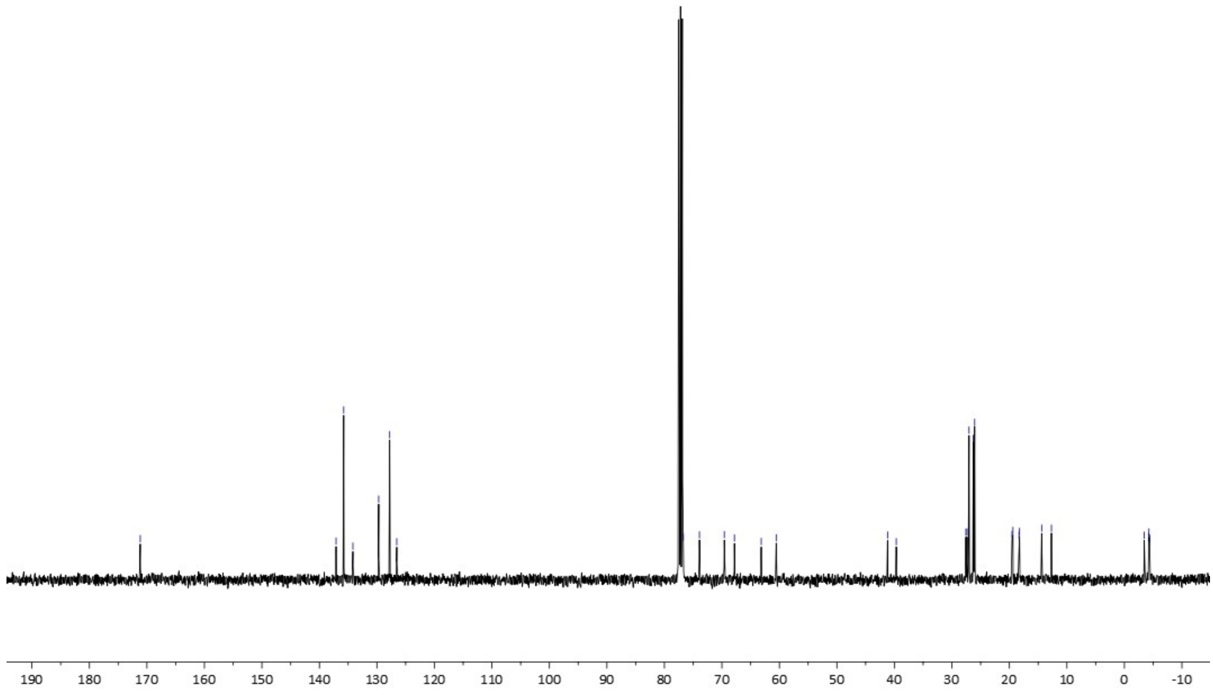

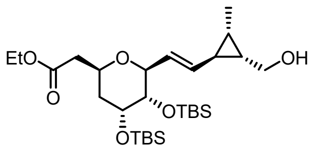

23

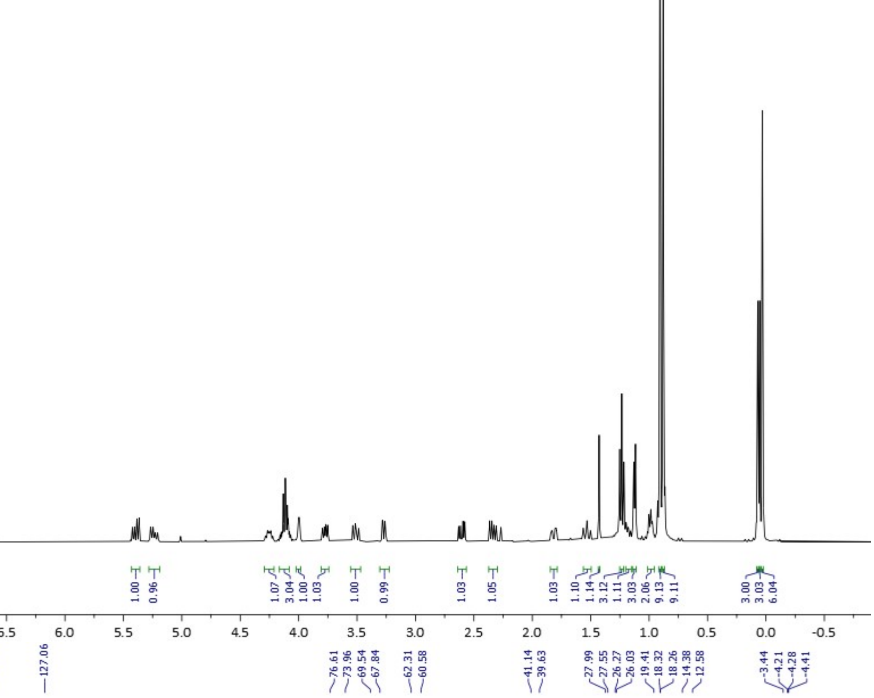

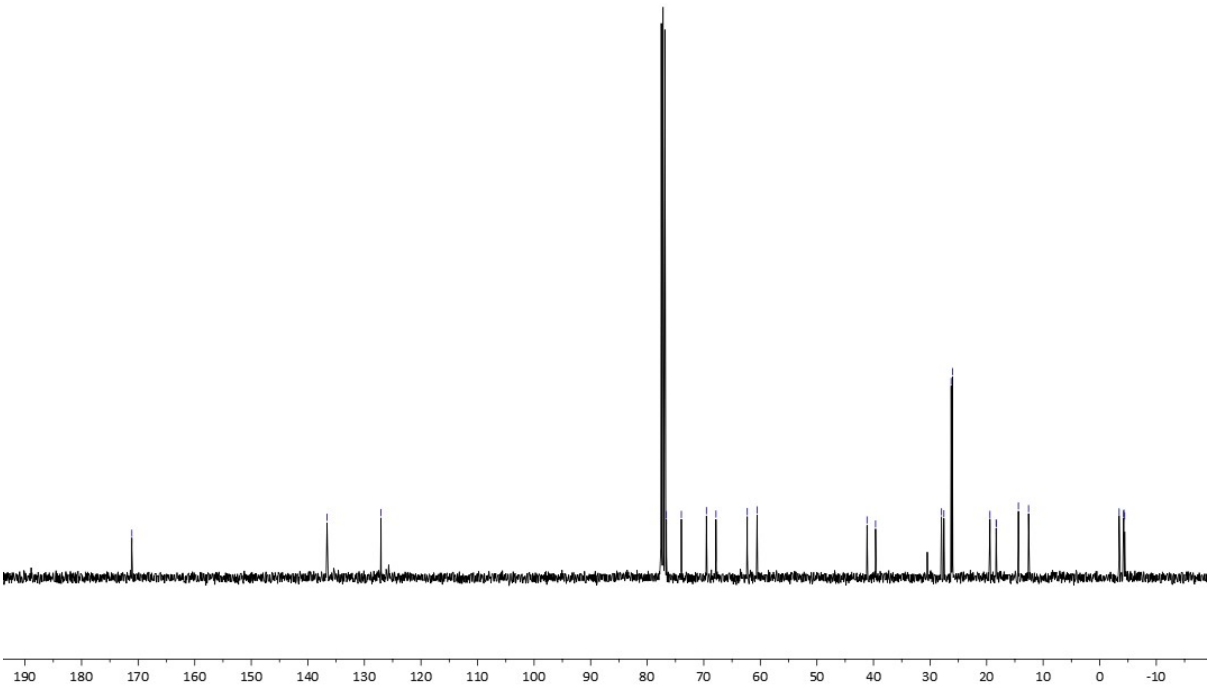

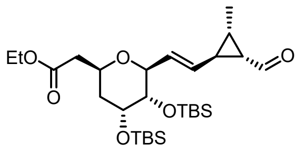

10

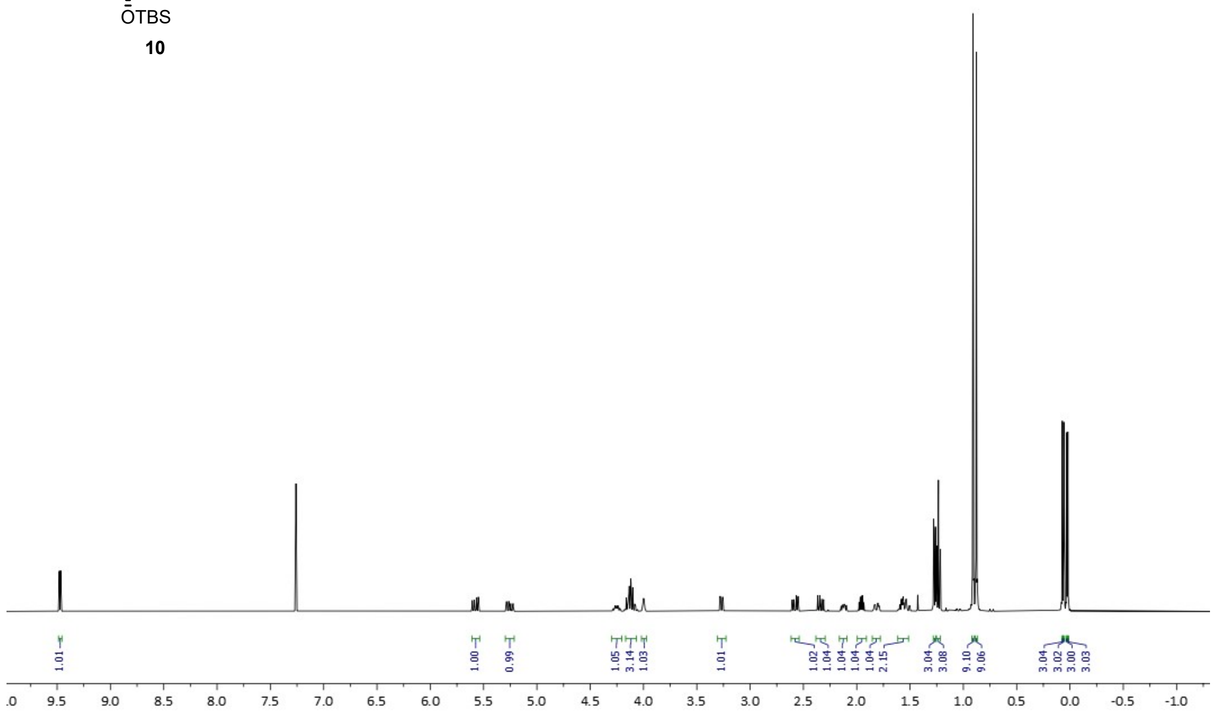

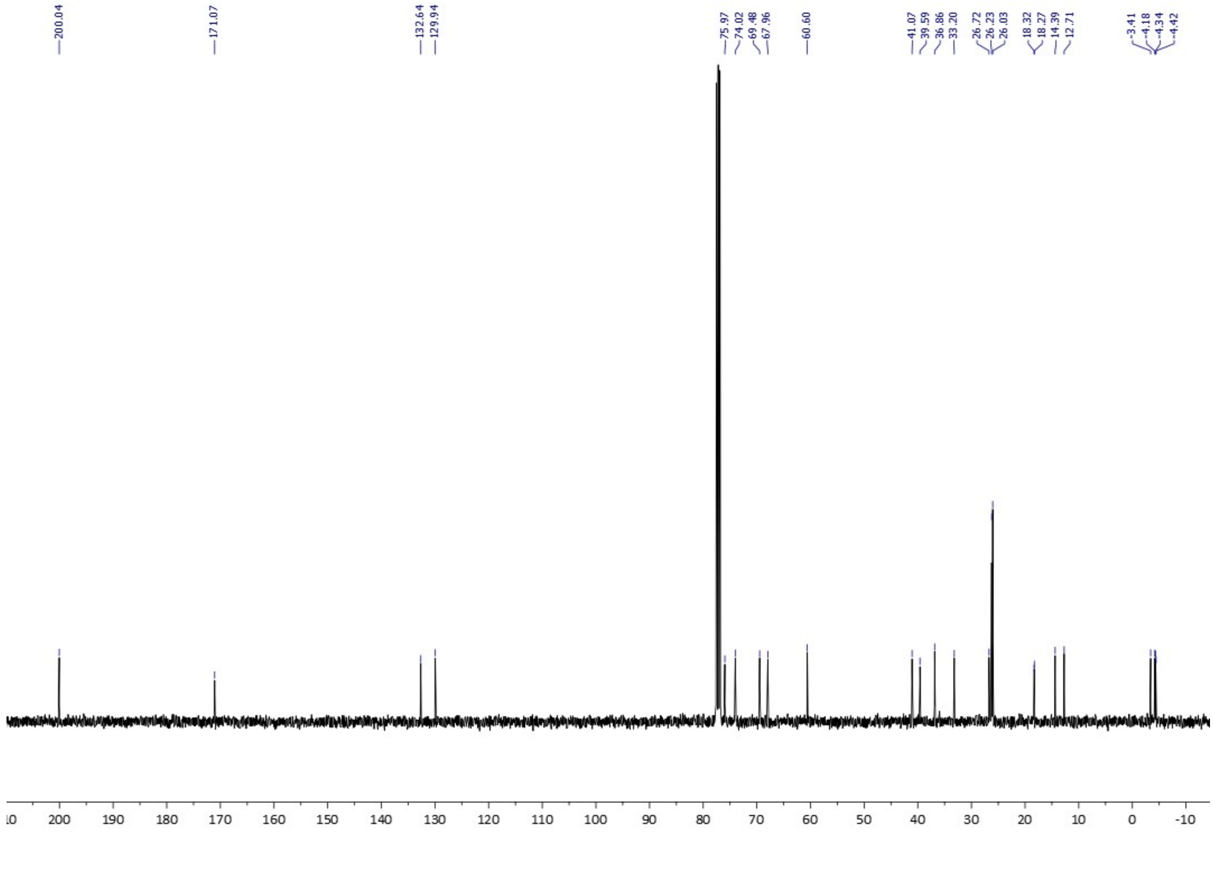

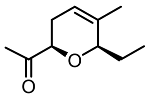

35

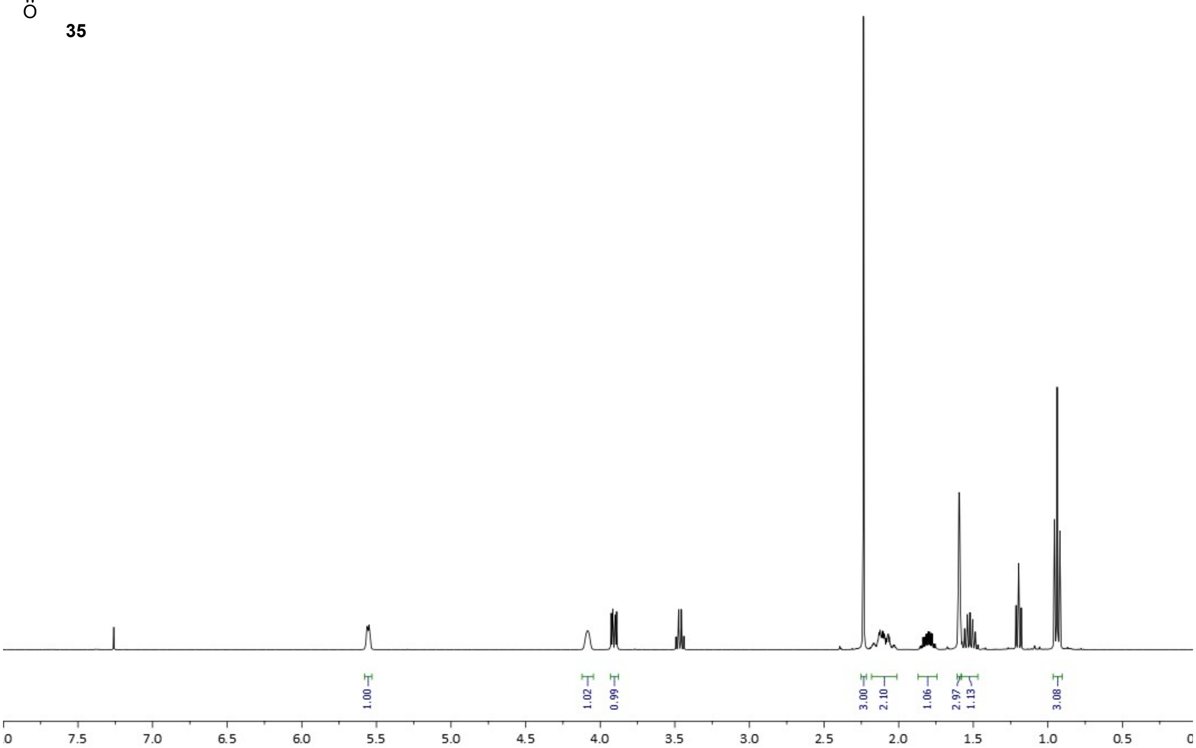
1.00
1.02
0.99
3.00
2.10
1.06
2.97
1.13
3.08

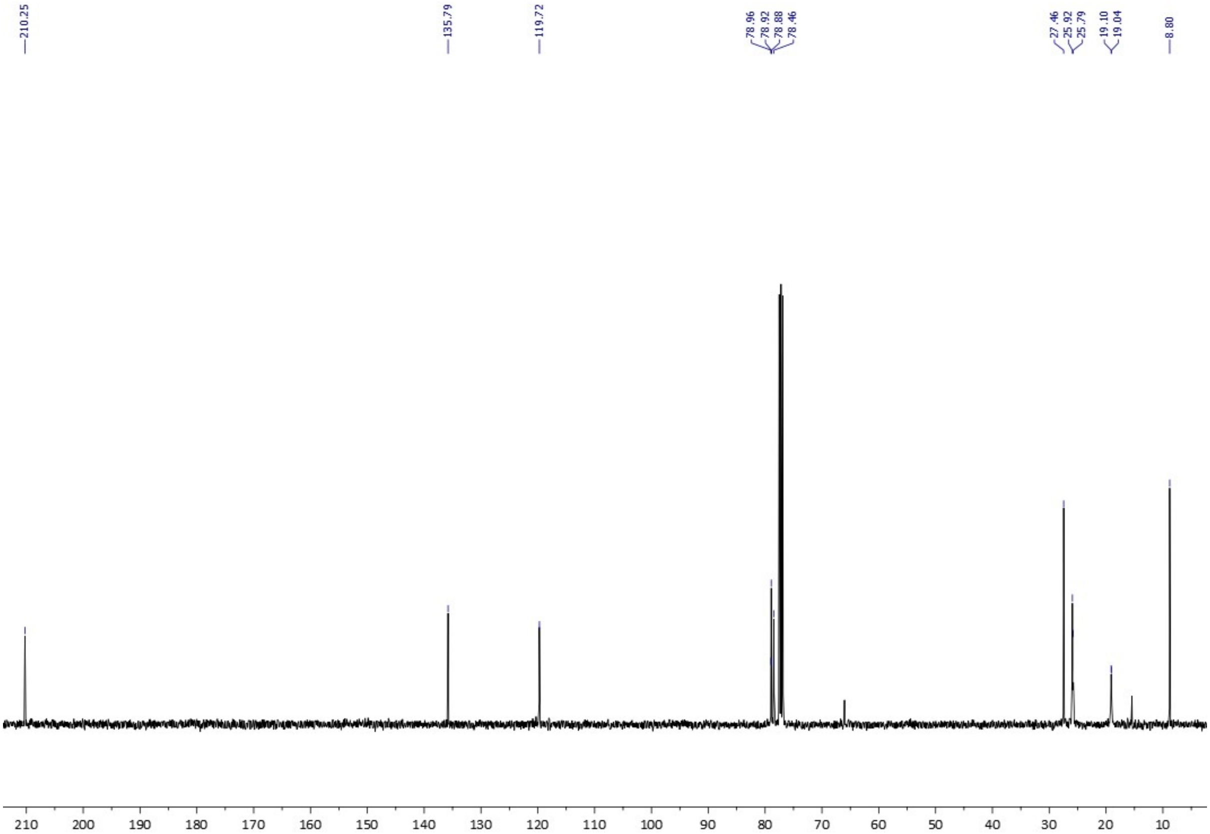
210.25
135.79
119.72
78.96
78.92
78.88
78.46
27.46
25.92
25.79
19.10
19.04
8.80

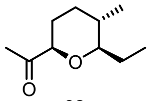
O
O

32

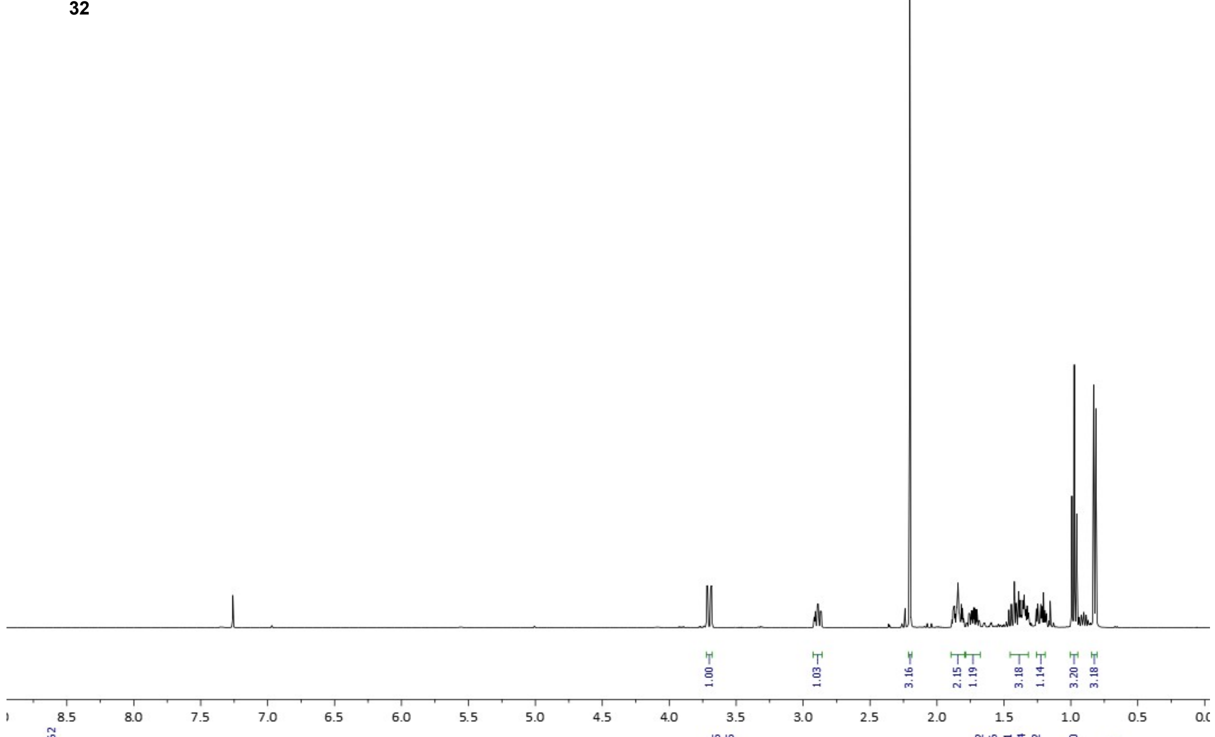
1.00
1.03
3.16
2.15
1.19
3.18
1.14
3.20
3.18
8.5
8.0
7.5
7.0
6.5
6.0
5.5
5.0
4.5
4.0
3.5
3.0
2.5
2.0
1.5
1.0
0.5

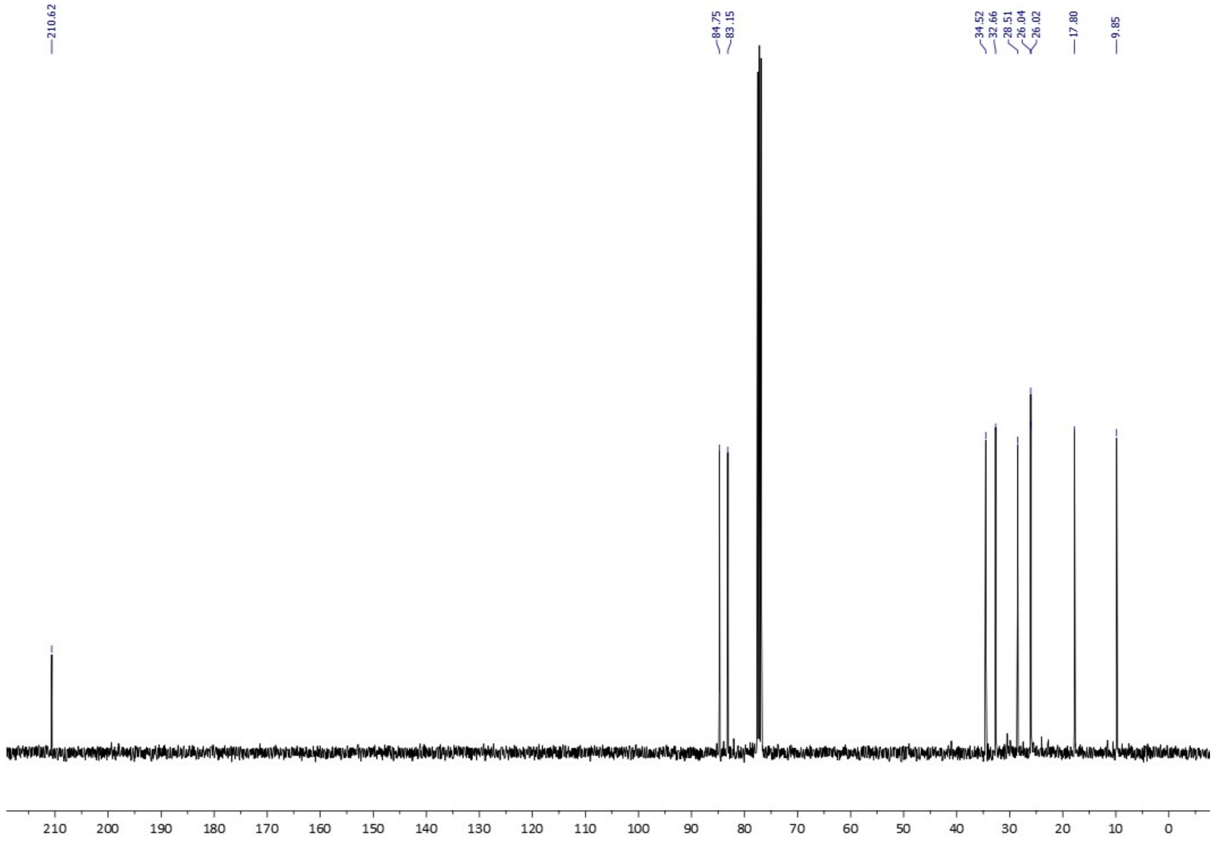
210.62
84.75
83.15
34.52
32.66
28.51
26.04
26.02
17.80
9.85
210
200
190
180
170
160
150
140
130
120
110
100
90
80
70
60
50
40
30
20
10
0

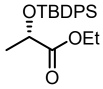
OTBDPS
OEt
O

S6

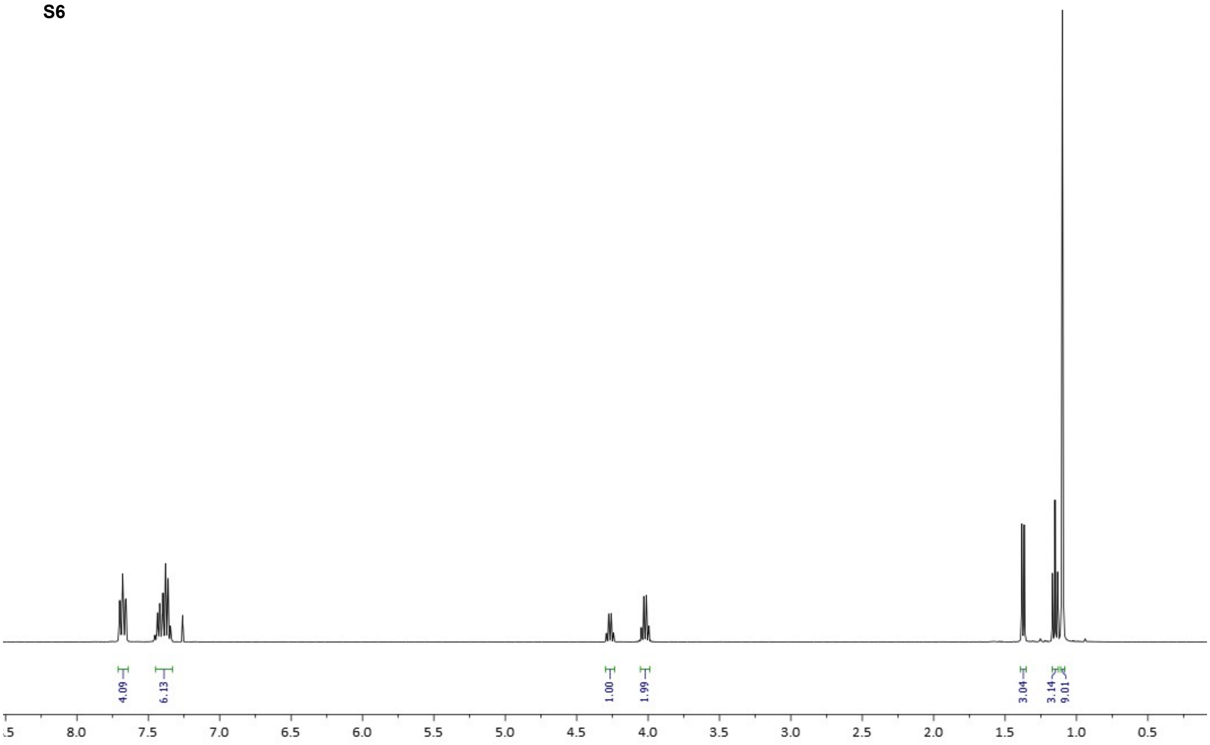
4.09
6.13
1.00
1.99
3.04
3.14
9.01
8.0 7.5 7.0 6.5 6.0 5.5 5.0 4.5 4.0 3.5 3.0 2.5 2.0 1.5 1.0 0.5

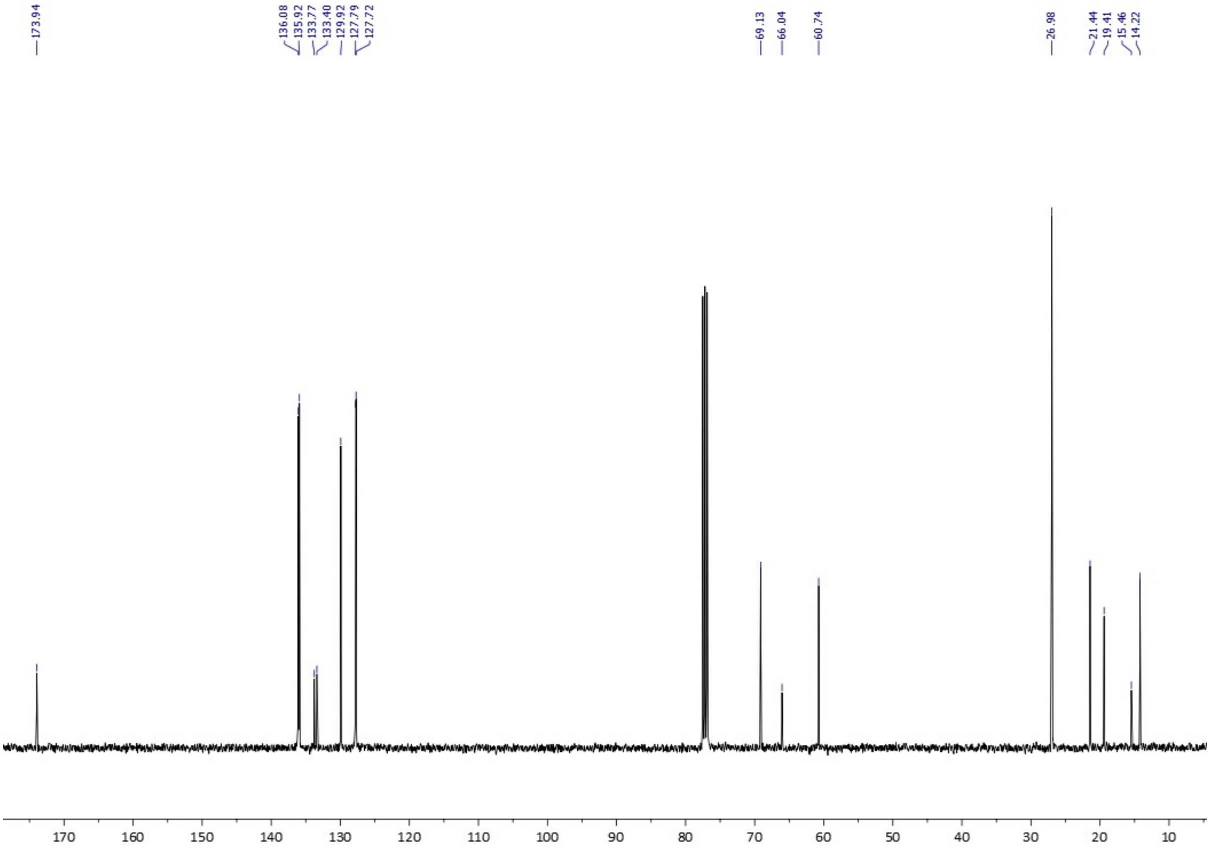
173.94
136.08
135.92
133.77
133.40
129.92
127.79
127.72
69.13
66.04
60.74
26.98
21.44
19.41
15.46
14.22
170 160 150 140 130 120 110 100 90 80 70 60 50 40 30 20 10

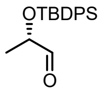
OTBDPS
O

27

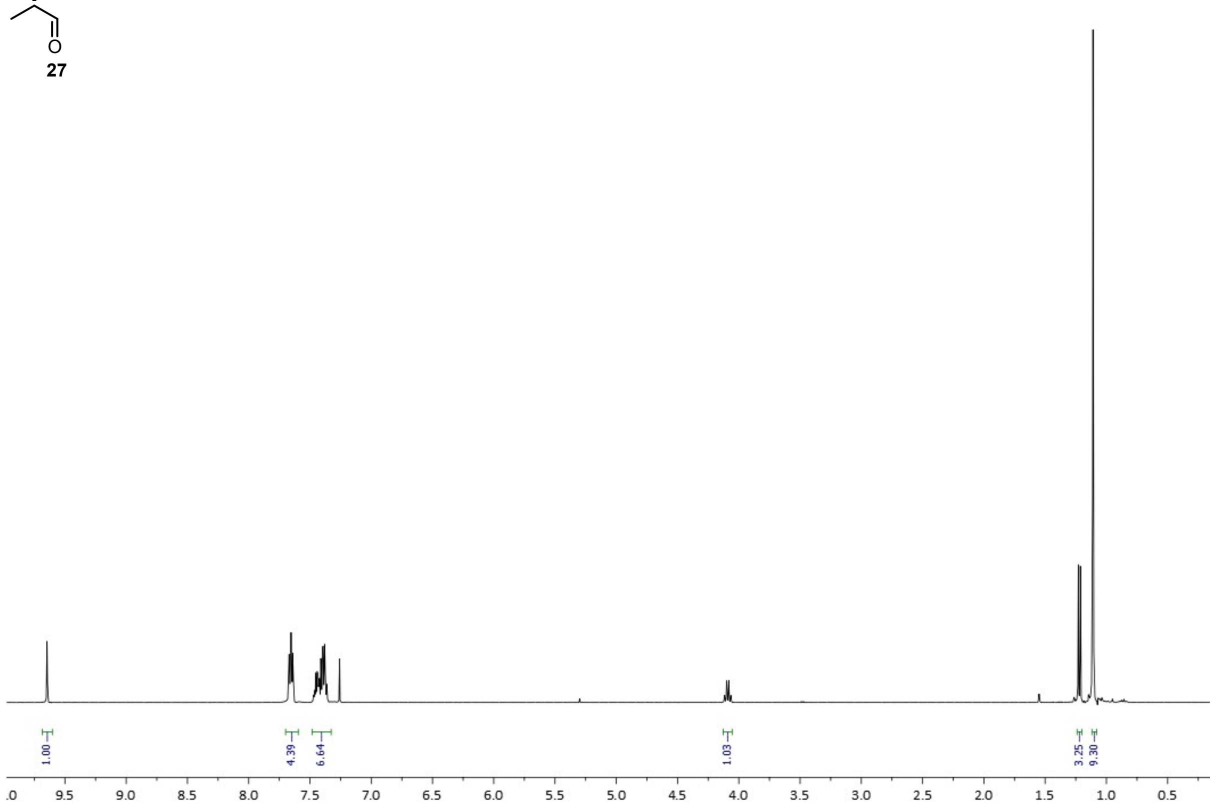
1.00
4.39
6.64
1.03
3.25
9.30
9.5 9.0 8.5 8.0 7.5 7.0 6.5 6.0 5.5 5.0 4.5 4.0 3.5 3.0 2.5 2.0 1.5 1.0 0.5

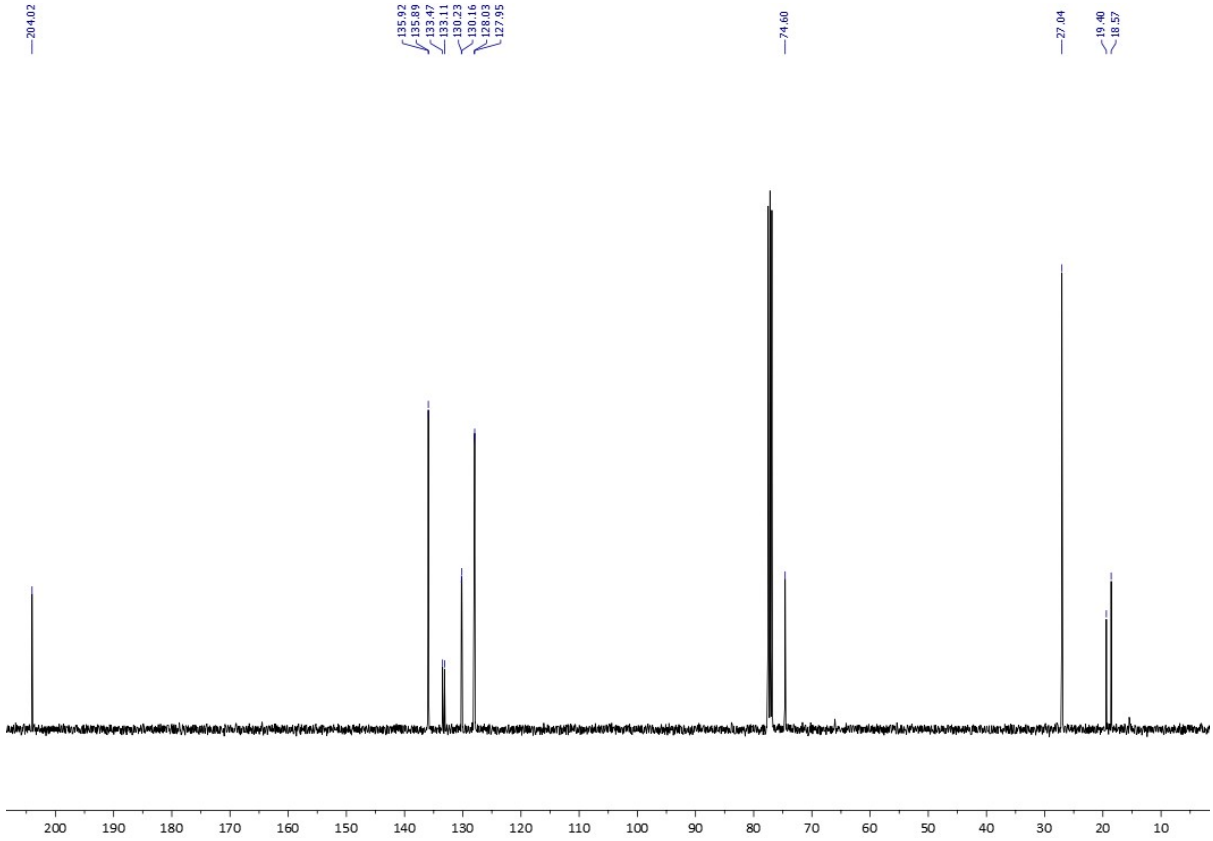
204.02
135.92
135.89
133.47
133.11
130.23
130.16
128.03
127.95
74.60
27.04
19.40
18.57
200 190 180 170 160 150 140 130 120 110 100 90 80 70 60 50 40 30 20 10

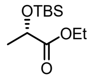

S7

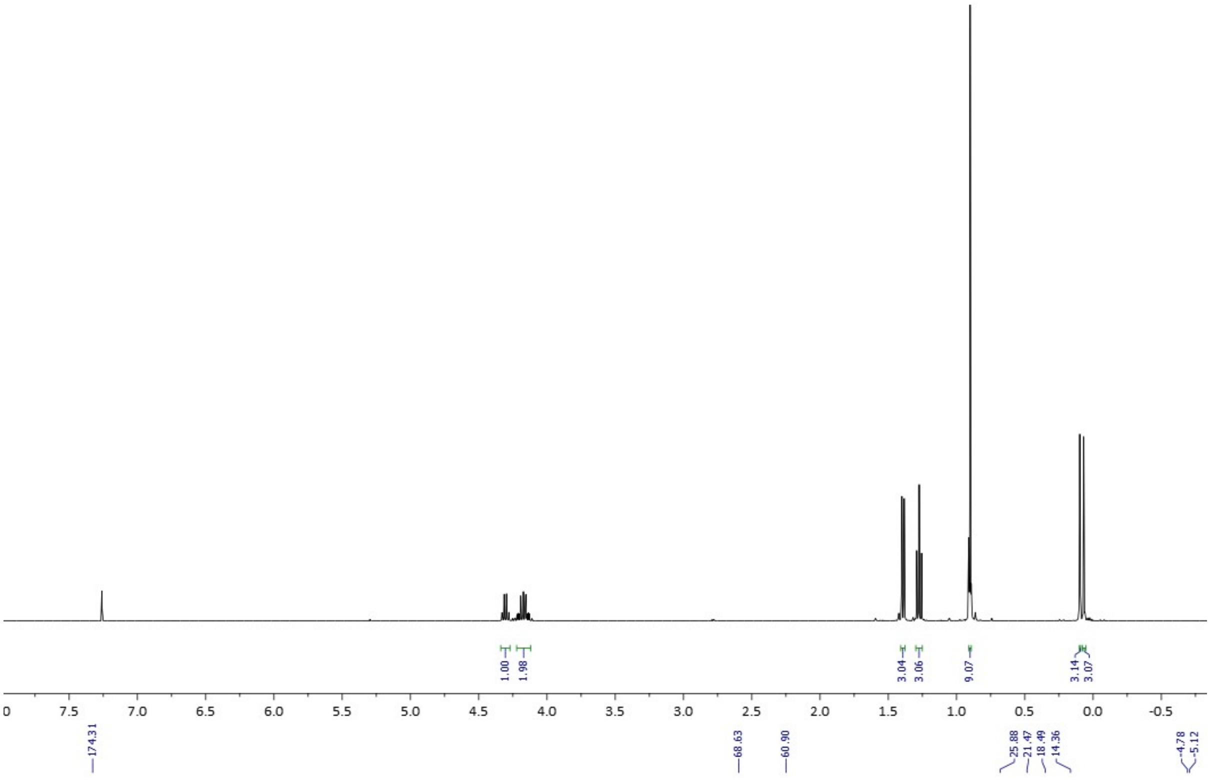

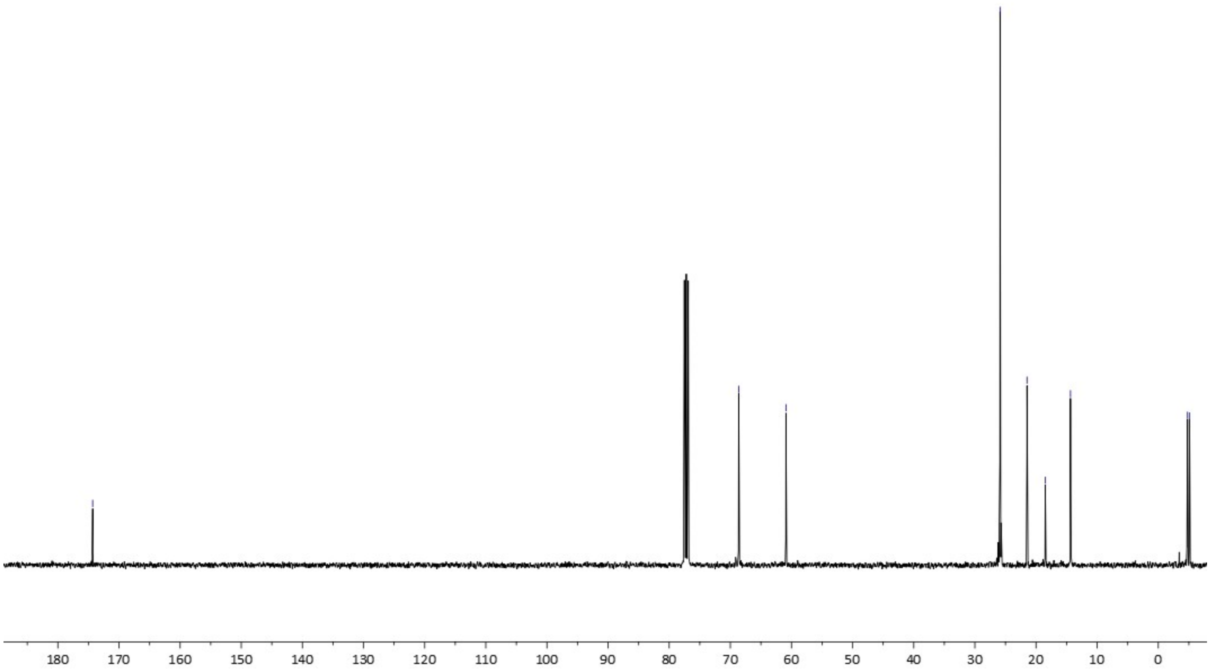

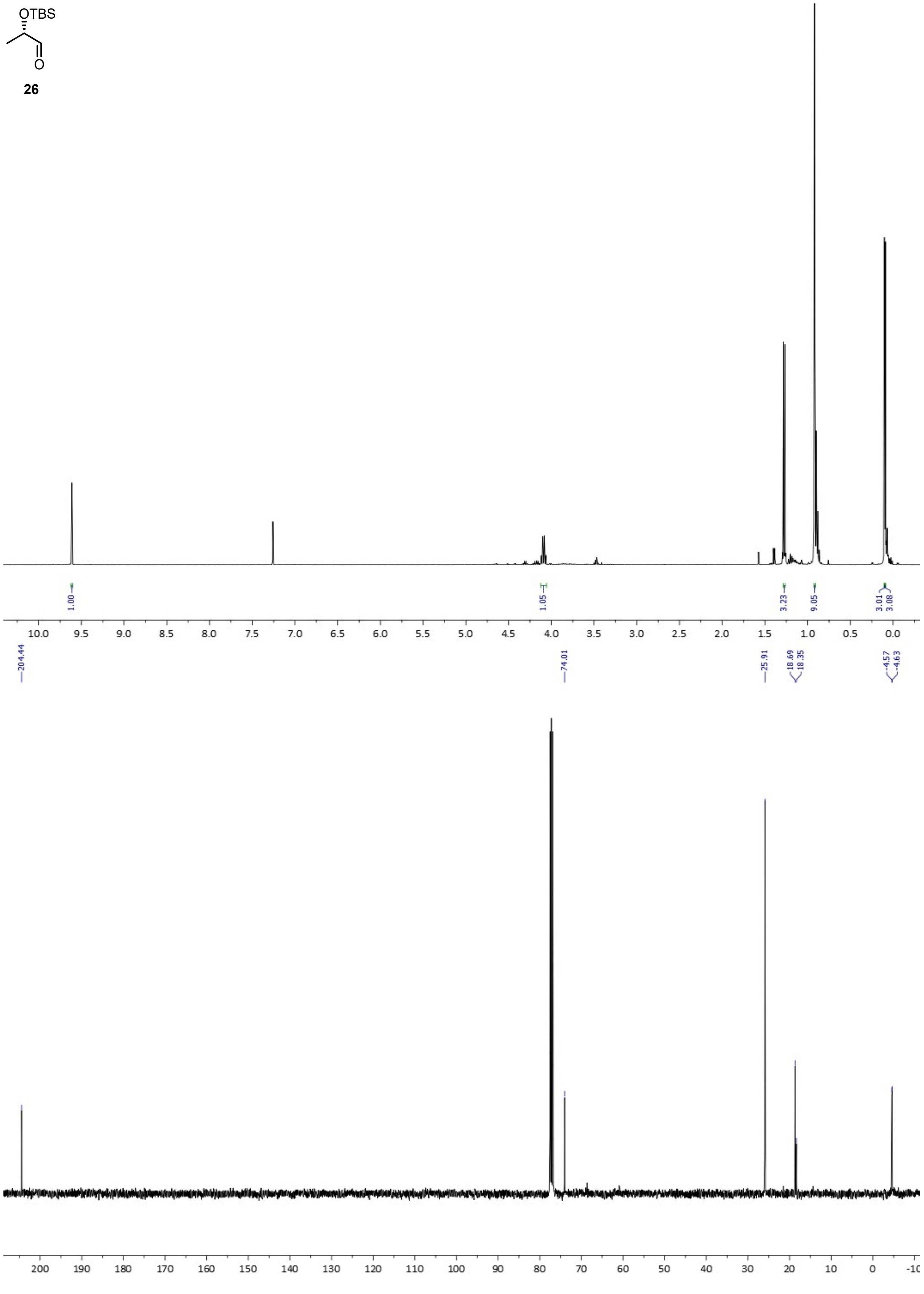
OTBS
O
1.00
1.05
3.23
9.05
3.01
3.08
204.44
74.01
25.91
18.69
18.35
-4.57
-4.63

26

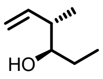
HO

25

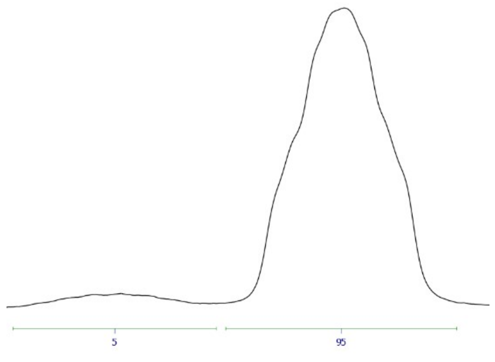
5
95

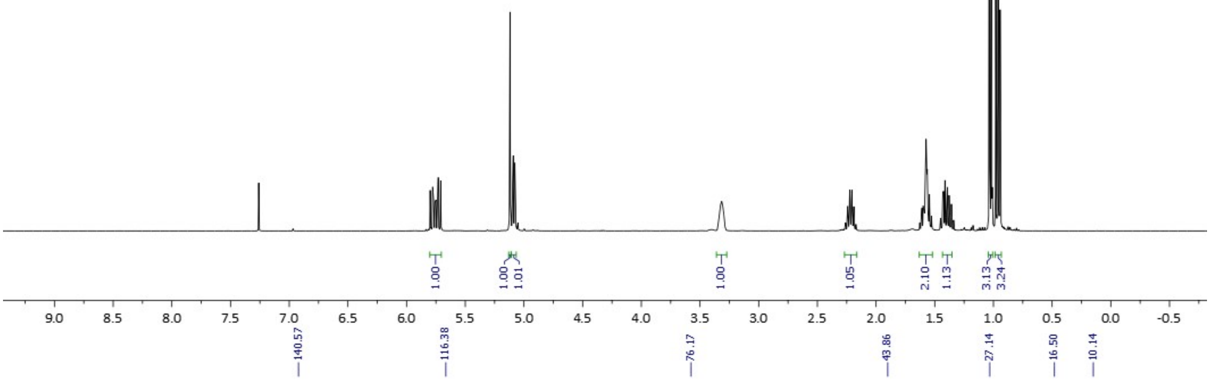
1.00
1.00
1.01
1.00
1.05
2.10
1.13
3.13
3.24
9.0 8.5 8.0 7.5 7.0 6.5 6.0 5.5 5.0 4.5 4.0 3.5 3.0 2.5 2.0 1.5 1.0 0.5 0.0 -0.5
140.57
116.38
76.17
43.86
27.14
16.50
10.14

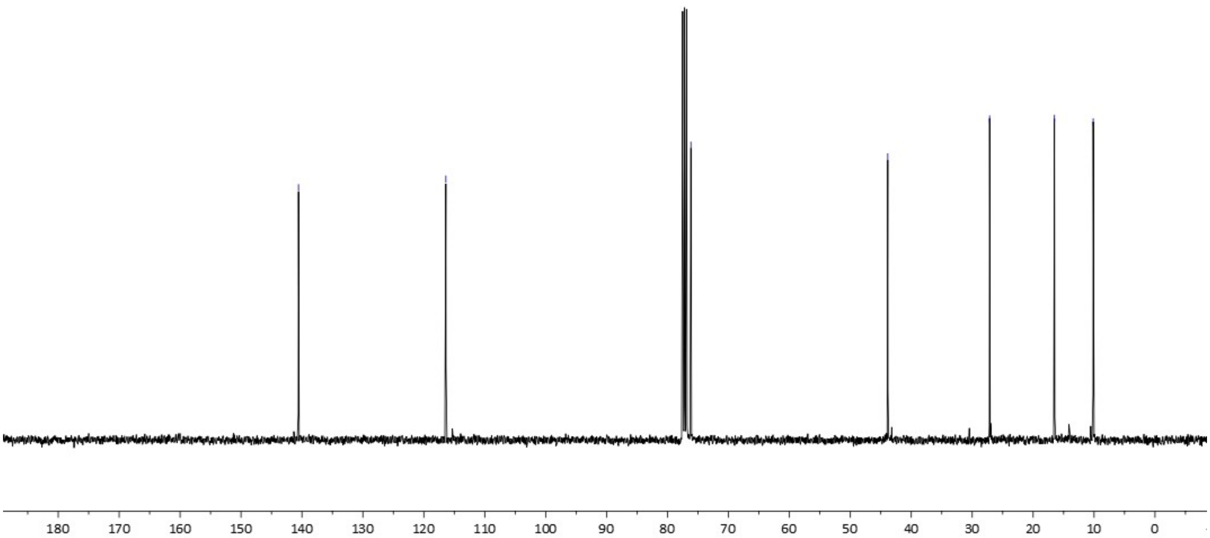
180 170 160 150 140 130 120 110 100 90 80 70 60 50 40 30 20 10 0

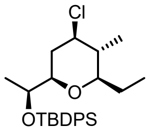
Cl
O
OTBDPS

29

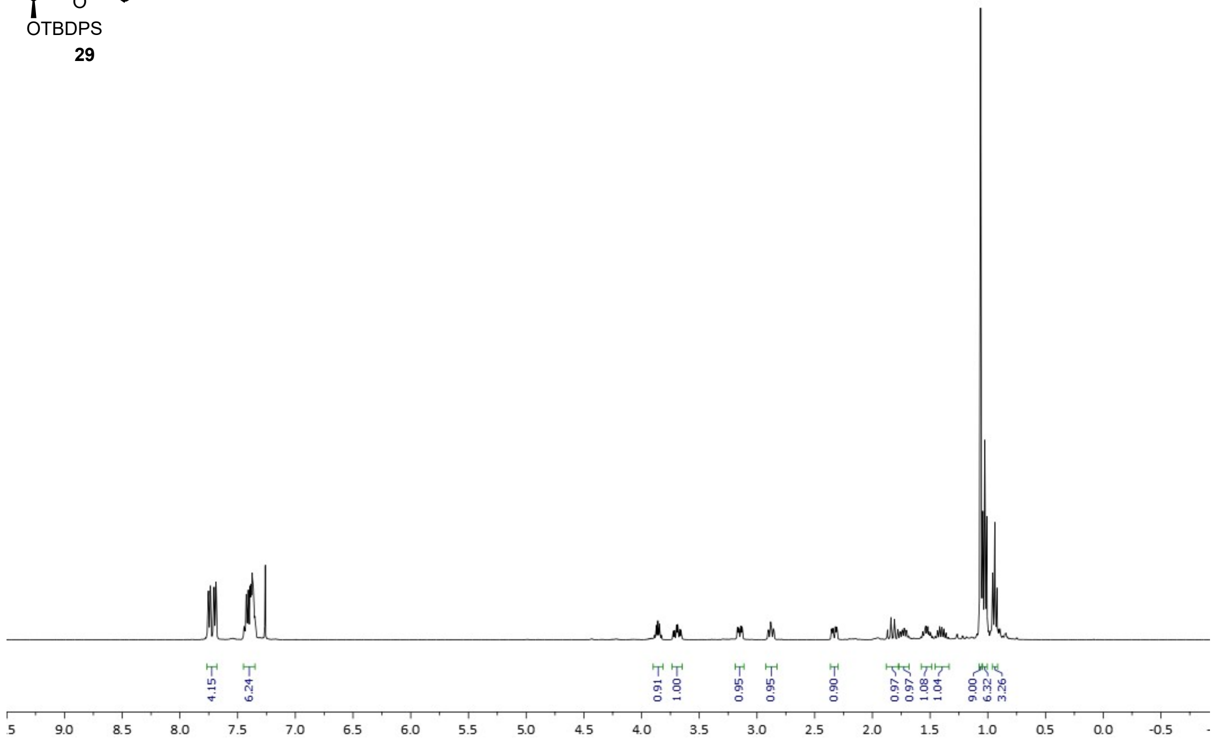
4.15
6.24
0.91
1.00
0.95
0.95
0.90
0.97
0.97
1.08
1.04
9.00
6.32
3.26
5 9.0 8.5 8.0 7.5 7.0 6.5 6.0 5.5 5.0 4.5 4.0 3.5 3.0 2.5 2.0 1.5 1.0 0.5 0.0 -0.5

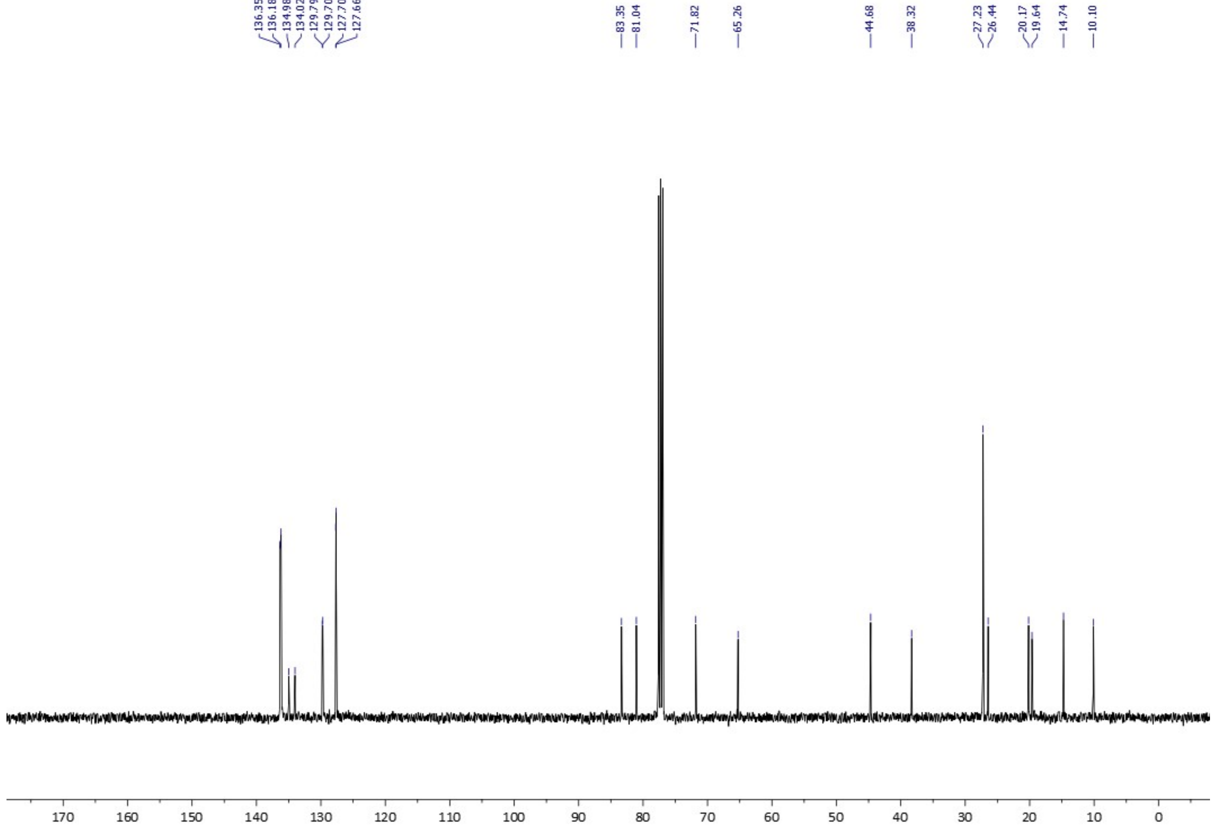
136.35
136.18
134.98
134.02
129.79
129.70
127.70
127.66
83.35
81.04
71.82
65.26
44.68
38.32
27.23
26.44
20.17
19.64
14.74
10.10
170 160 150 140 130 120 110 100 90 80 70 60 50 40 30 20 10 0

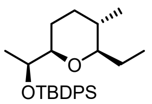
O
OTBDPS

30

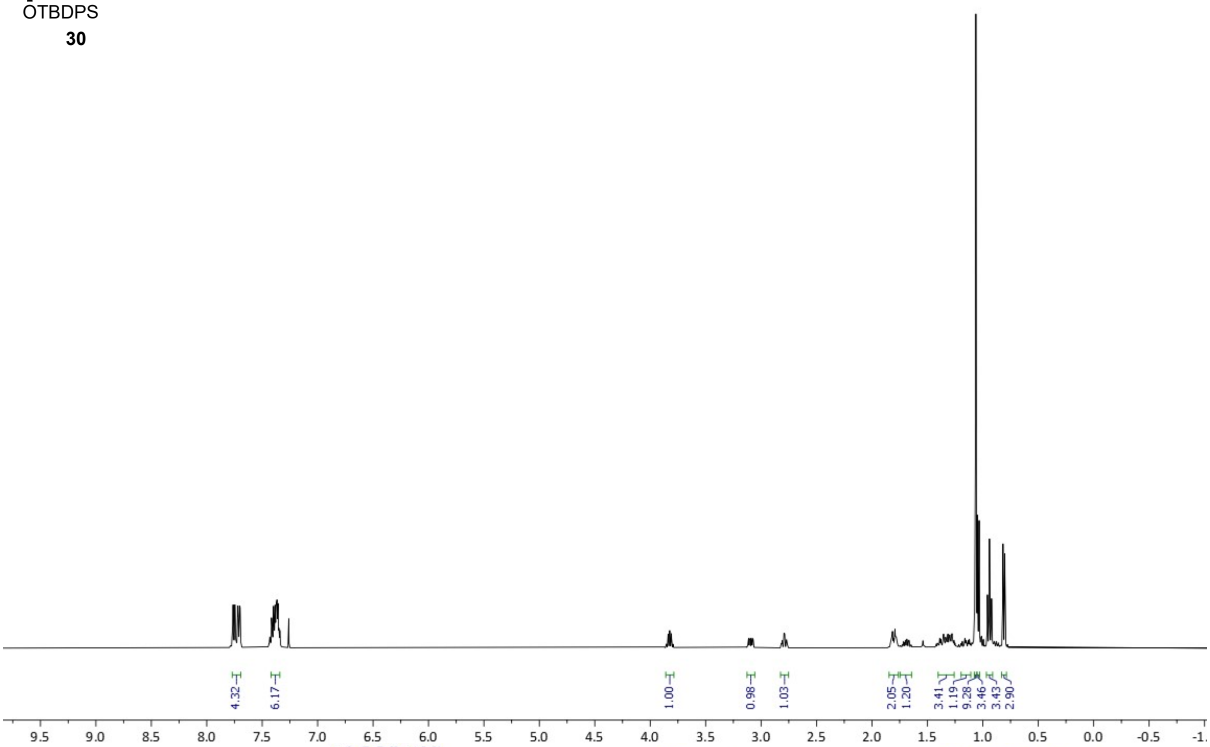
4.32
6.17
1.00
0.98
1.03
2.05
1.20
3.41
1.19
9.28
3.46
3.43
2.90
9.5 9.0 8.5 8.0 7.5 7.0 6.5 6.0 5.5 5.0 4.5 4.0 3.5 3.0 2.5 2.0 1.5 1.0 0.5 0.0 -0.5

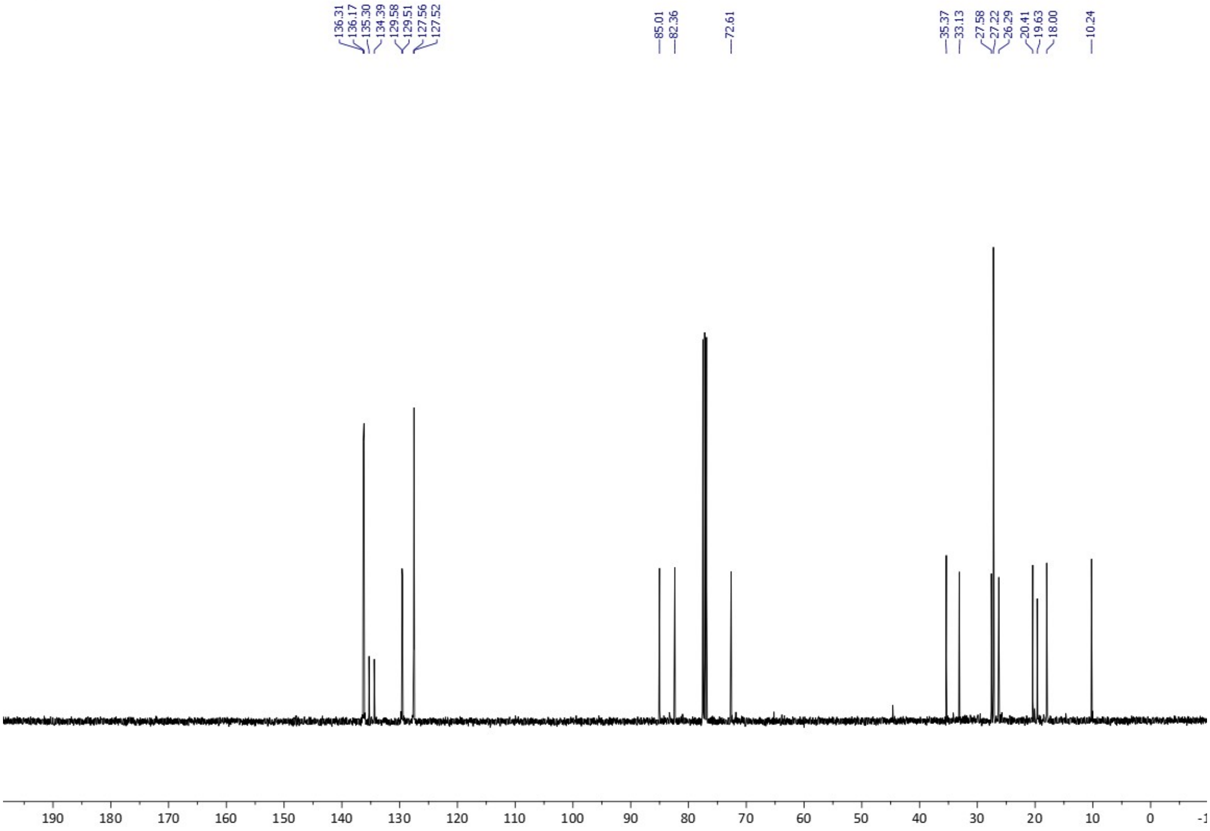
136.31
136.17
135.30
134.39
129.58
129.51
127.56
127.52
85.01
82.36
72.61
35.37
33.13
27.58
27.22
26.29
20.41
19.63
18.00
10.24
190 180 170 160 150 140 130 120 110 100 90 80 70 60 50 40 30 20 10 0

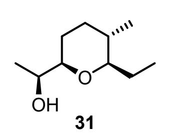
O
OH

31

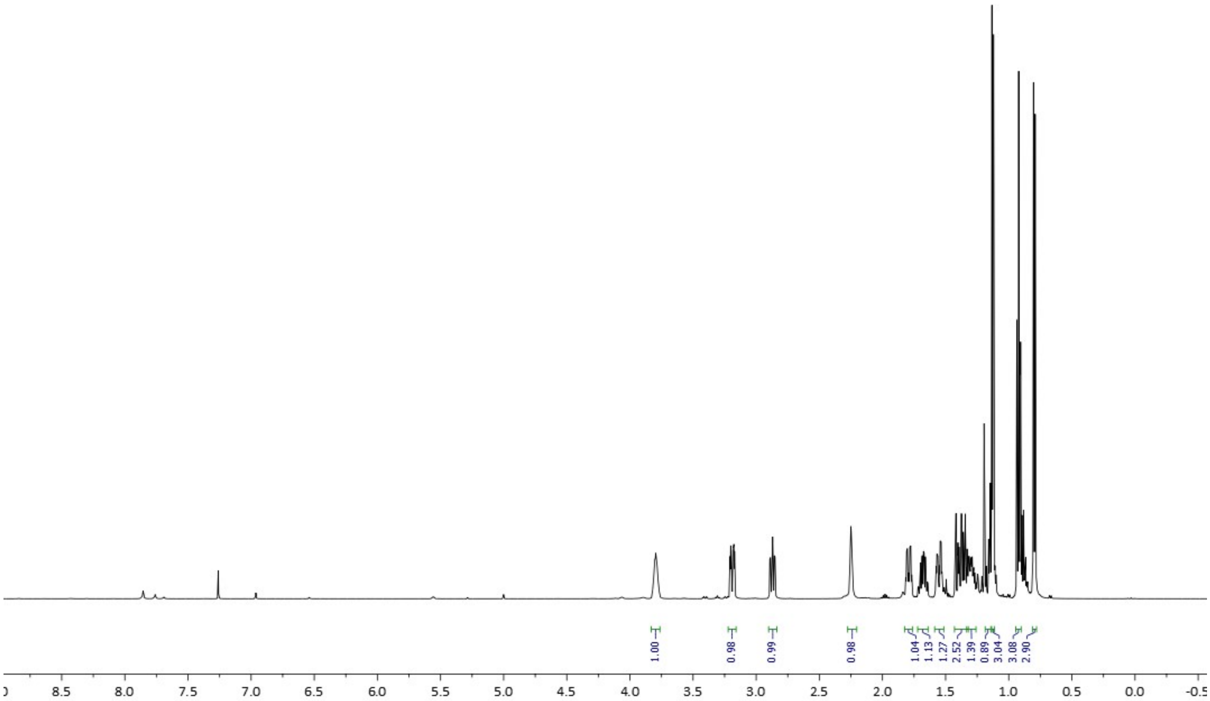
1.00
0.98
0.99
0.98
1.04
1.13
1.27
2.52
1.39
0.89
3.04
3.08
2.90
8.5 8.0 7.5 7.0 6.5 6.0 5.5 5.0 4.5 4.0 3.5 3.0 2.5 2.0 1.5 1.0 0.5 0.0 -0.5

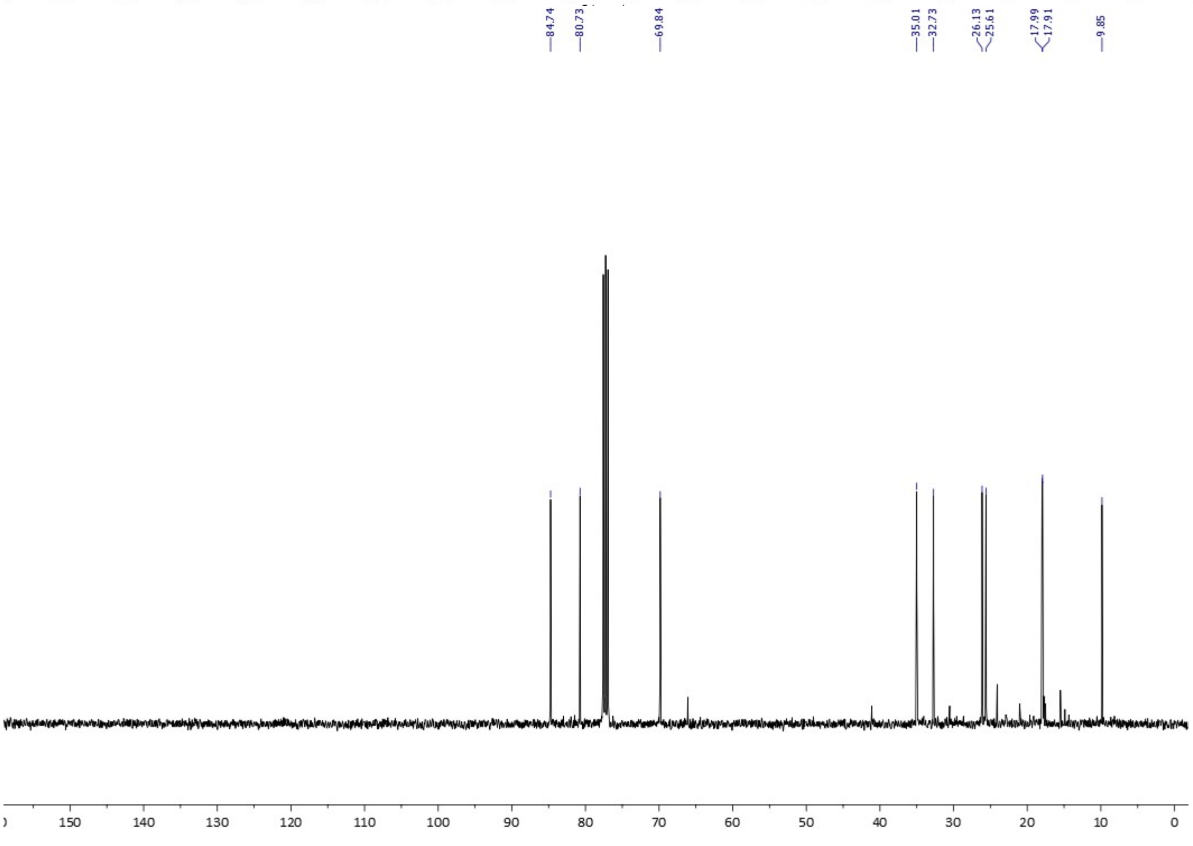
84.74
80.73
69.84
35.01
32.73
26.13
25.61
17.99
17.91
9.85
150 140 130 120 110 100 90 80 70 60 50 40 30 20 10 0

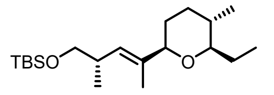
TBSO
O

37

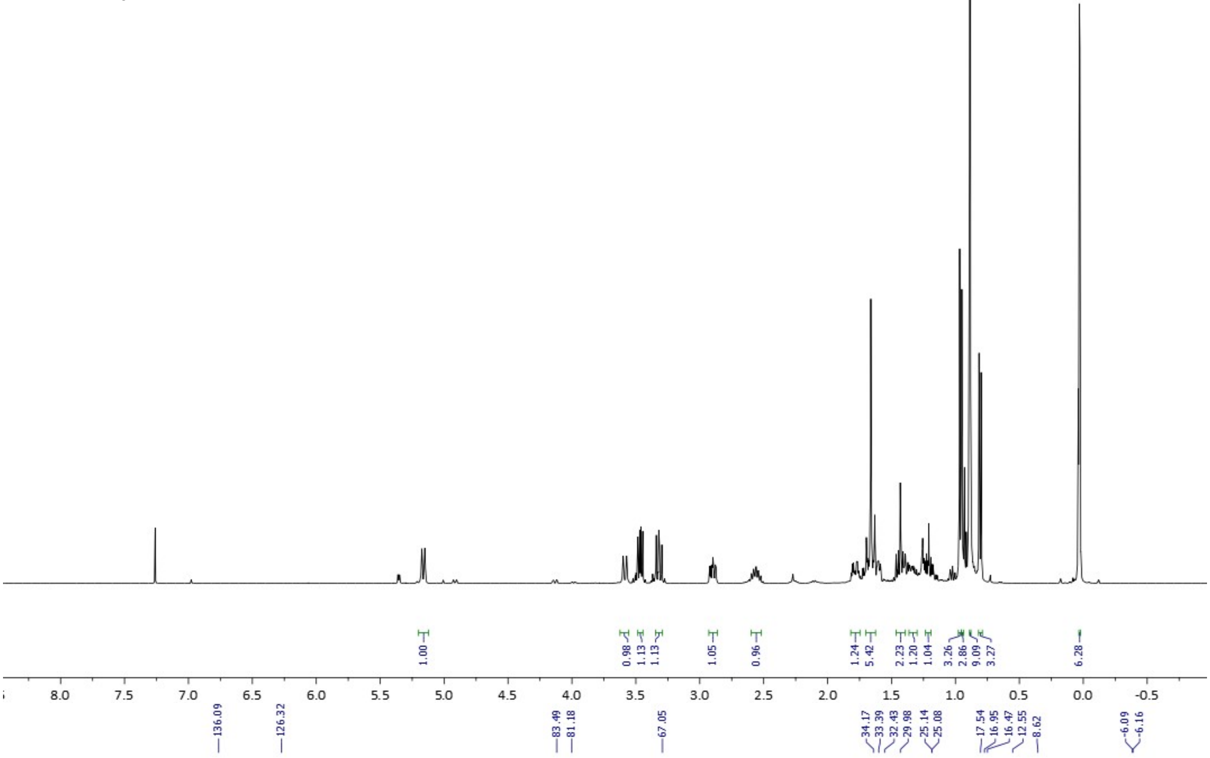
1.00
0.98
1.13
1.13
1.05
0.96
1.24
5.42
2.23
1.20
1.04
3.26
2.86
9.09
3.27
6.28
8.0 7.5 7.0 6.5 6.0 5.5 5.0 4.5 4.0 3.5 3.0 2.5 2.0 1.5 1.0 0.5 0.0 -0.5
136.09
126.32
83.49
81.18
67.05
34.17
33.39
32.43
29.98
25.14
25.08
17.54
16.95
16.47
12.55
8.62
-6.09
-6.16

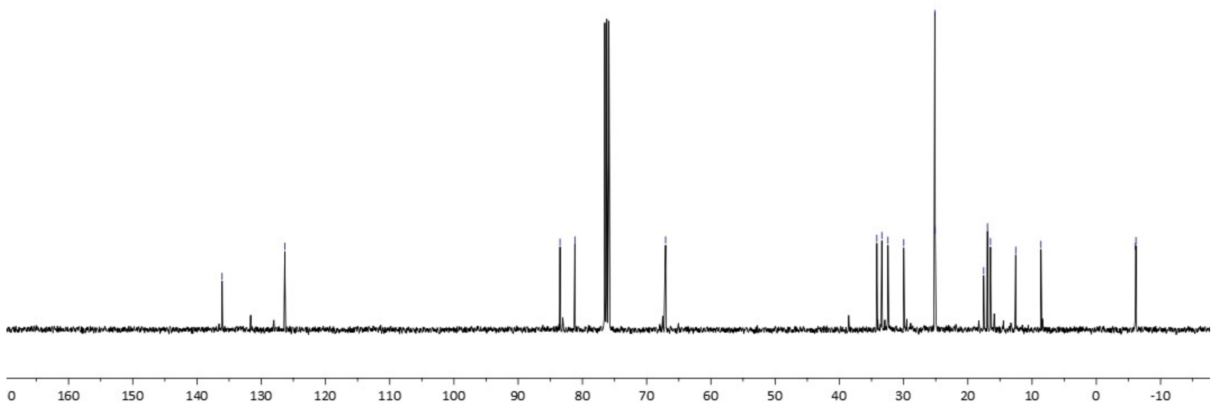
160 150 140 130 120 110 100 90 80 70 60 50 40 30 20 10 0 -10

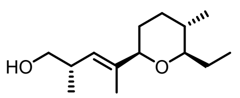
HO
O

38

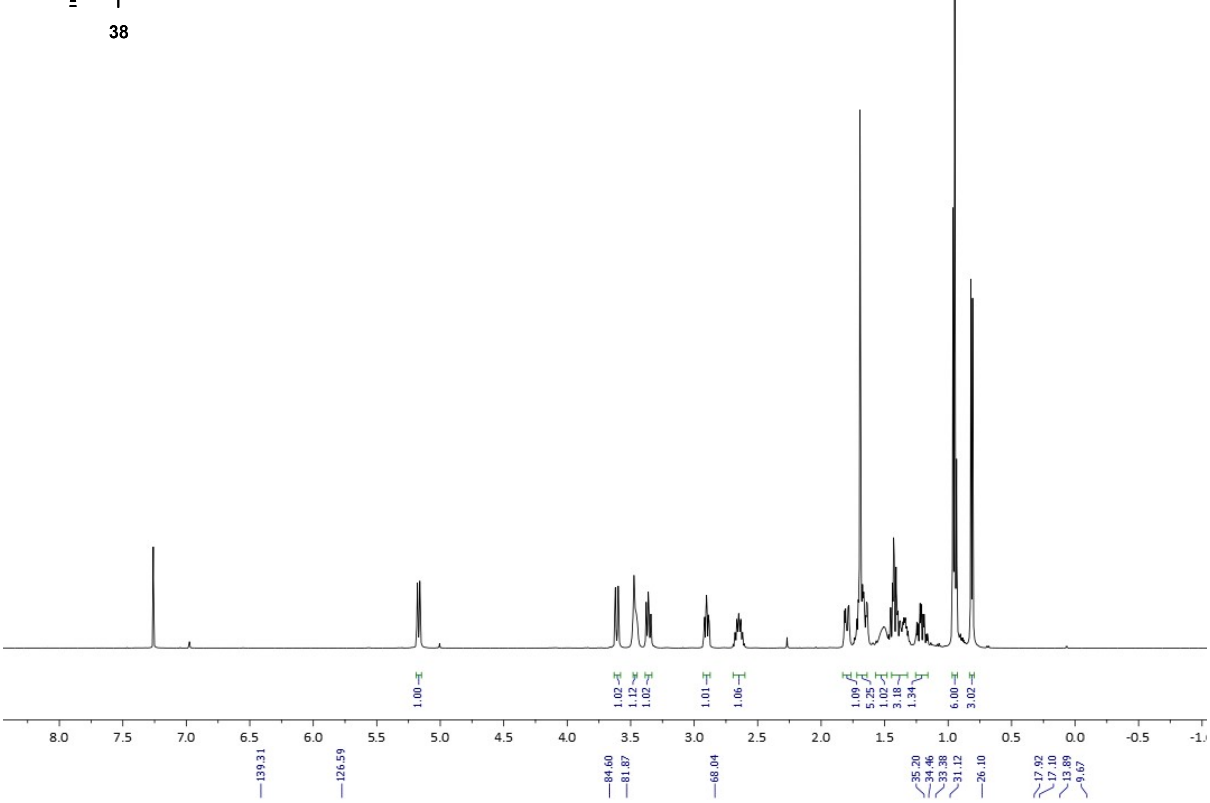
1.00
1.02
1.12
1.02
1.01
1.06
1.09
5.25
1.02
3.18
1.34
6.00
3.02
8.0 7.5 7.0 6.5 6.0 5.5 5.0 4.5 4.0 3.5 3.0 2.5 2.0 1.5 1.0 0.5 0.0 -0.5
139.31
126.59
84.60
81.87
68.04
35.20
34.46
33.38
31.12
26.10
17.92
17.10
13.89
9.67

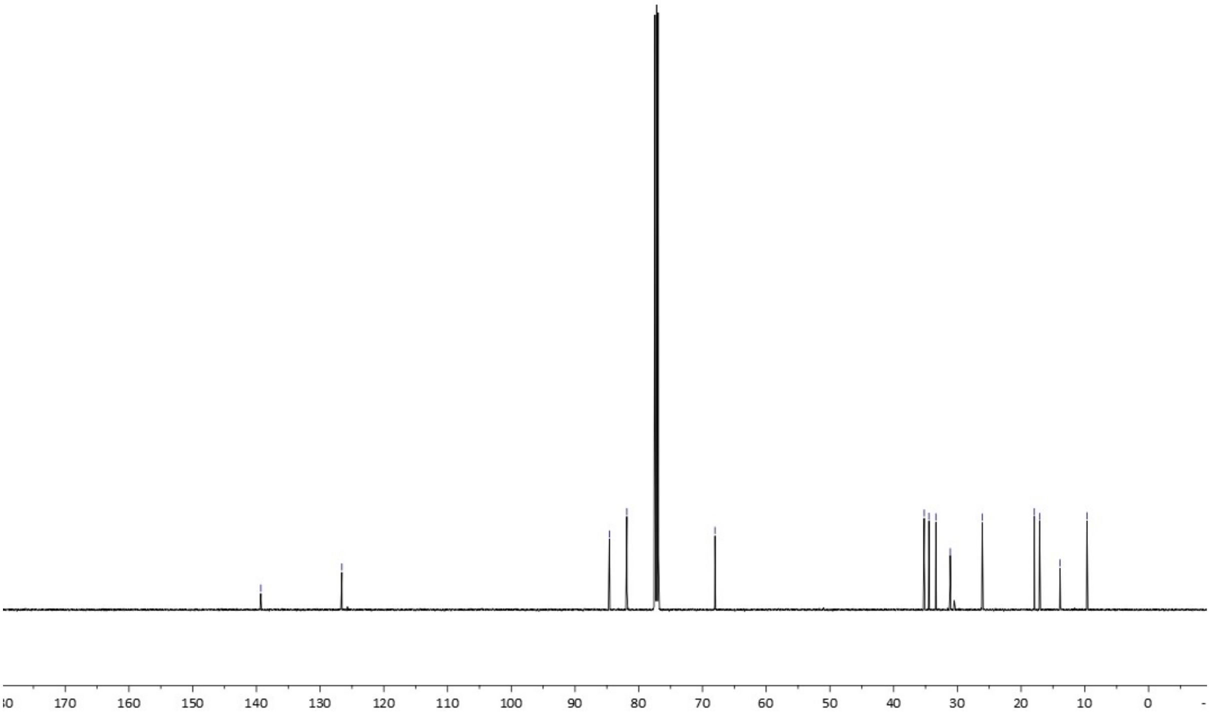
170 160 150 140 130 120 110 100 90 80 70 60 50 40 30 20 10 0

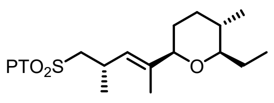

11

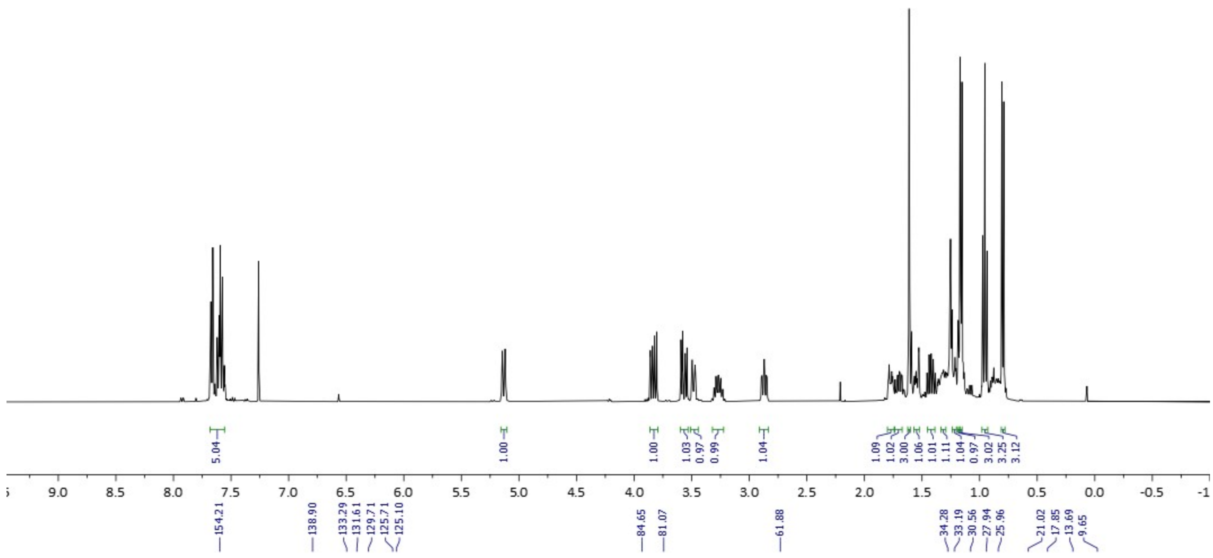

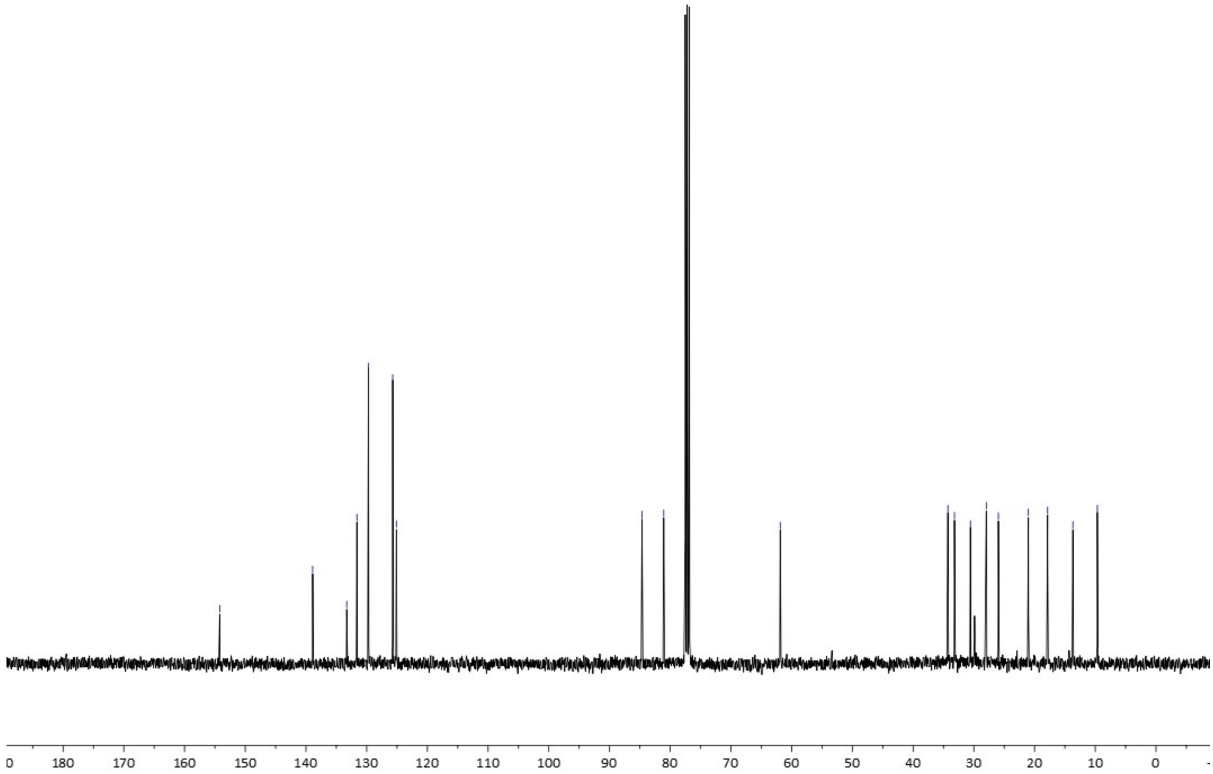

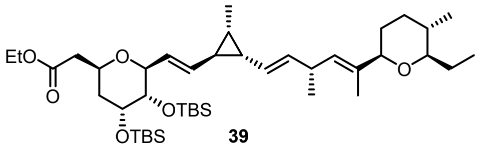
EtO
O
O
OTBS
OTBS
O

39
E/*Z* = 10:1

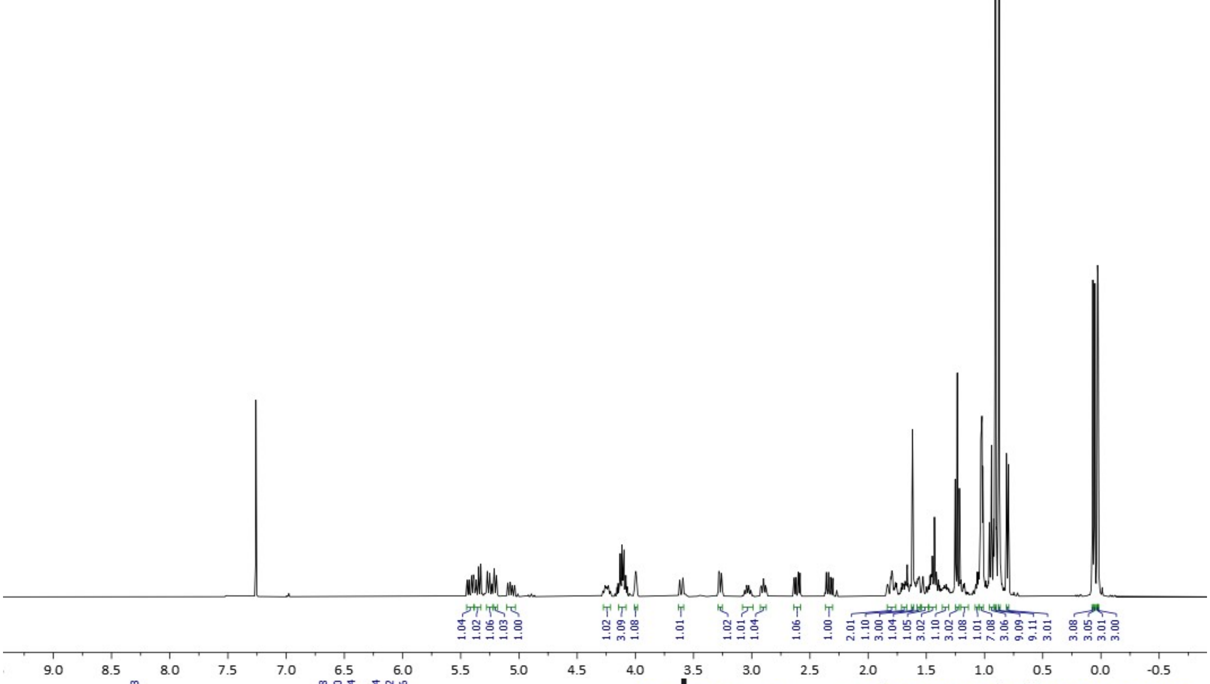
1.04
1.02
1.06
1.03
1.00
1.02
3.09
1.08
1.01
1.02
1.01
1.04
1.06
1.00
2.01
1.10
3.00
1.04
1.05
3.02
1.10
3.02
1.08
1.01
7.08
3.06
9.09
9.11
3.01
3.08
3.05
3.01
3.00
9.0 8.5 8.0 7.5 7.0 6.5 6.0 5.5 5.0 4.5 4.0 3.5 3.0 2.5 2.0 1.5 1.0 0.5 0.0 -0.5

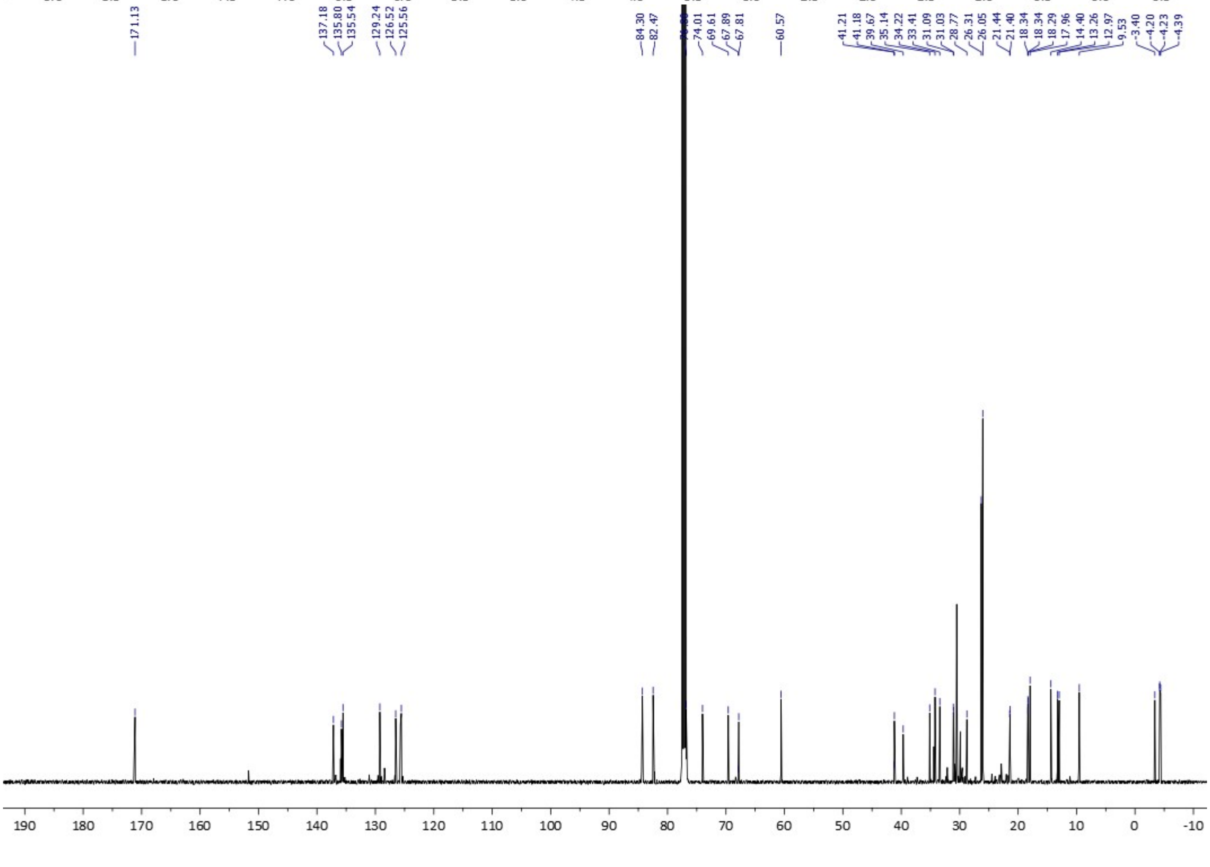
171.13
137.18
135.80
135.54
129.24
126.52
125.56
84.30
82.47
74.01
69.61
67.89
67.81
60.57
41.21
41.18
39.67
35.14
34.22
33.41
31.09
31.03
28.77
26.31
26.05
21.44
21.40
18.34
18.34
18.29
17.96
14.40
13.26
12.97
9.53
-3.40
-4.20
-4.23
-4.39
190 180 170 160 150 140 130 120 110 100 90 80 70 60 50 40 30 20 10 0 -10

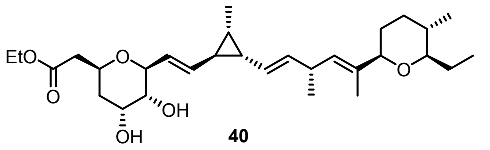
EtO
O
O
OH
OH
O

40
E/*Z* = 10:1

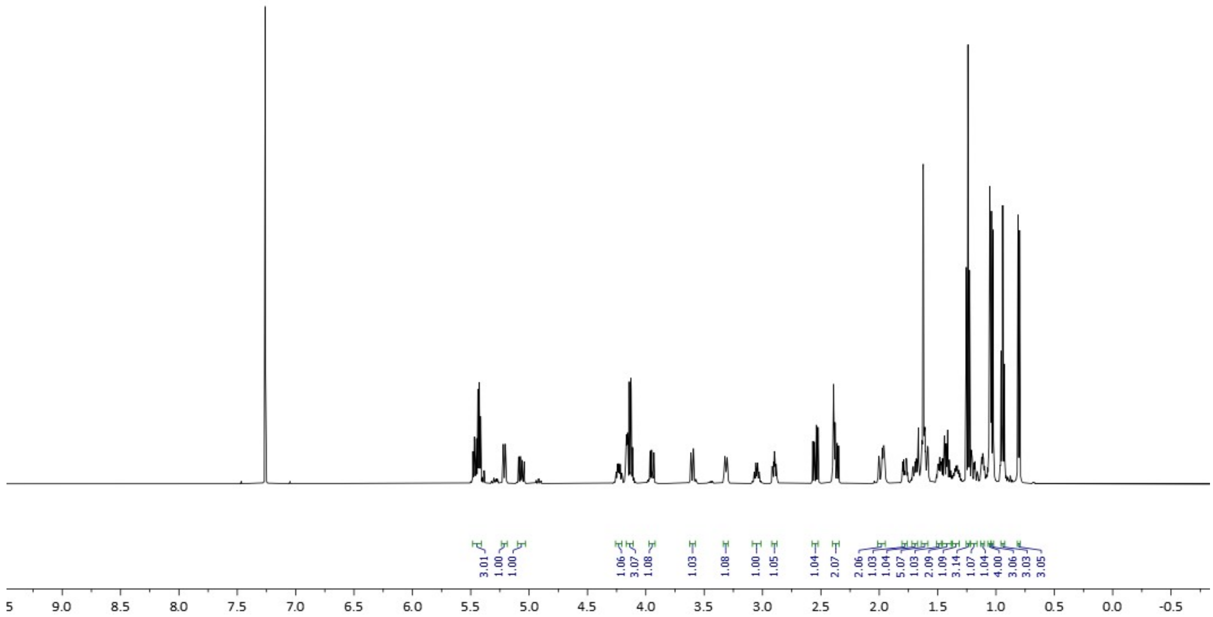
3.01
1.00
1.00
1.06
3.07
1.08
1.03
1.08
1.00
1.05
1.04
2.07
2.06
1.03
1.04
5.07
1.03
2.09
1.09
3.14
1.07
1.04
4.00
3.06
3.03
3.05
9.0 8.5 8.0 7.5 7.0 6.5 6.0 5.5 5.0 4.5 4.0 3.5 3.0 2.5 2.0 1.5 1.0 0.5 0.0 -0.5

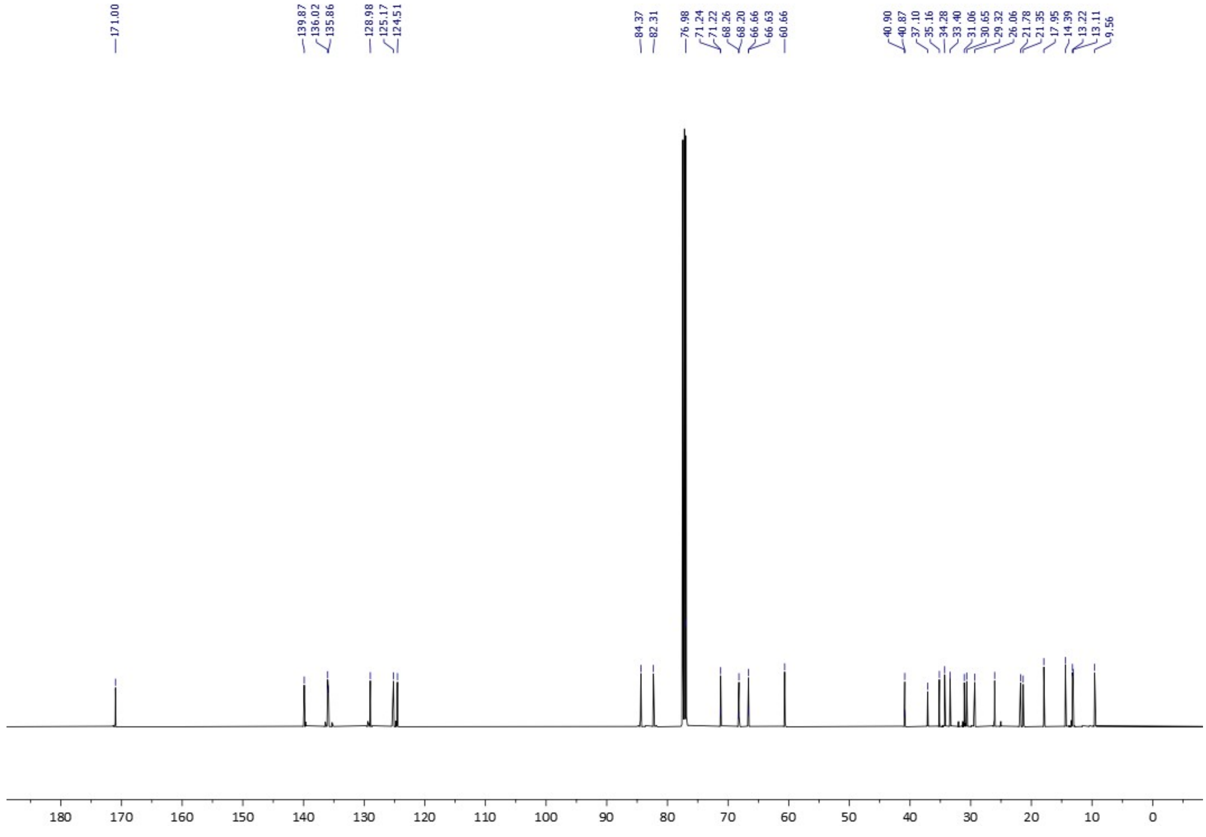
171.00
139.87
136.02
135.86
128.98
125.17
124.51
84.37
82.31
76.98
71.24
71.22
68.26
68.20
66.66
66.63
60.66
40.90
40.87
37.10
35.16
34.28
33.40
31.06
30.65
29.32
26.06
21.78
21.35
17.95
14.39
13.22
13.11
9.56
180 170 160 150 140 130 120 110 100 90 80 70 60 50 40 30 20 10 0

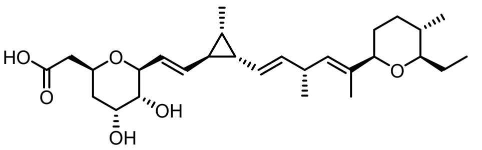

20,21-dihydroambruticin F (9)

E/*Z* = 10:1

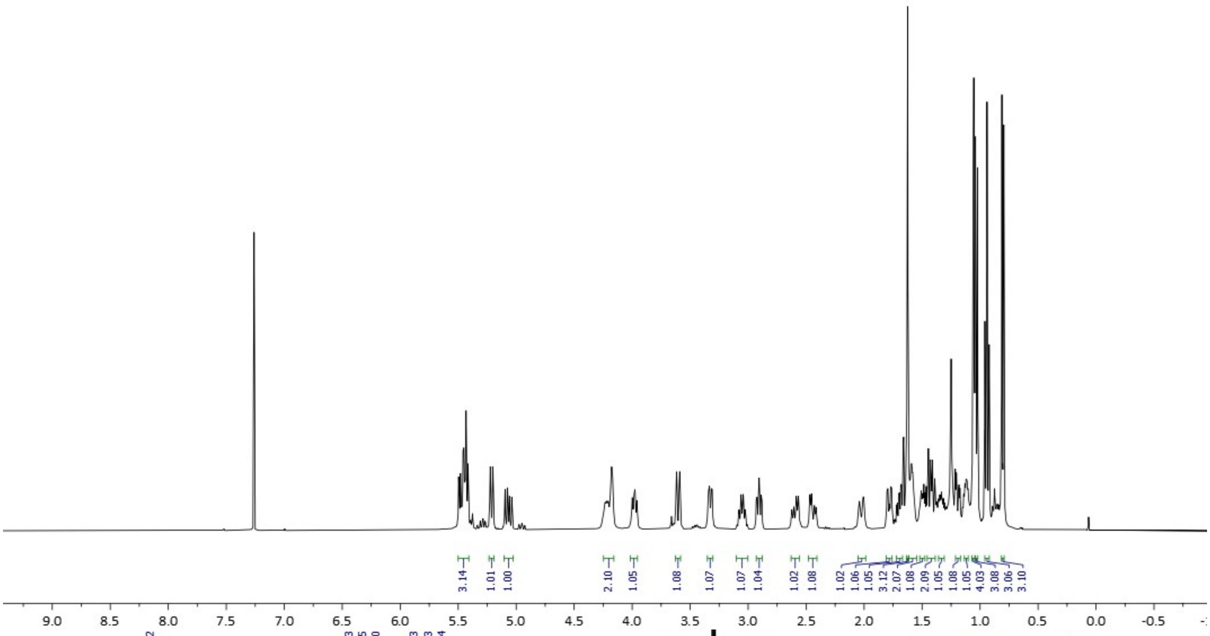

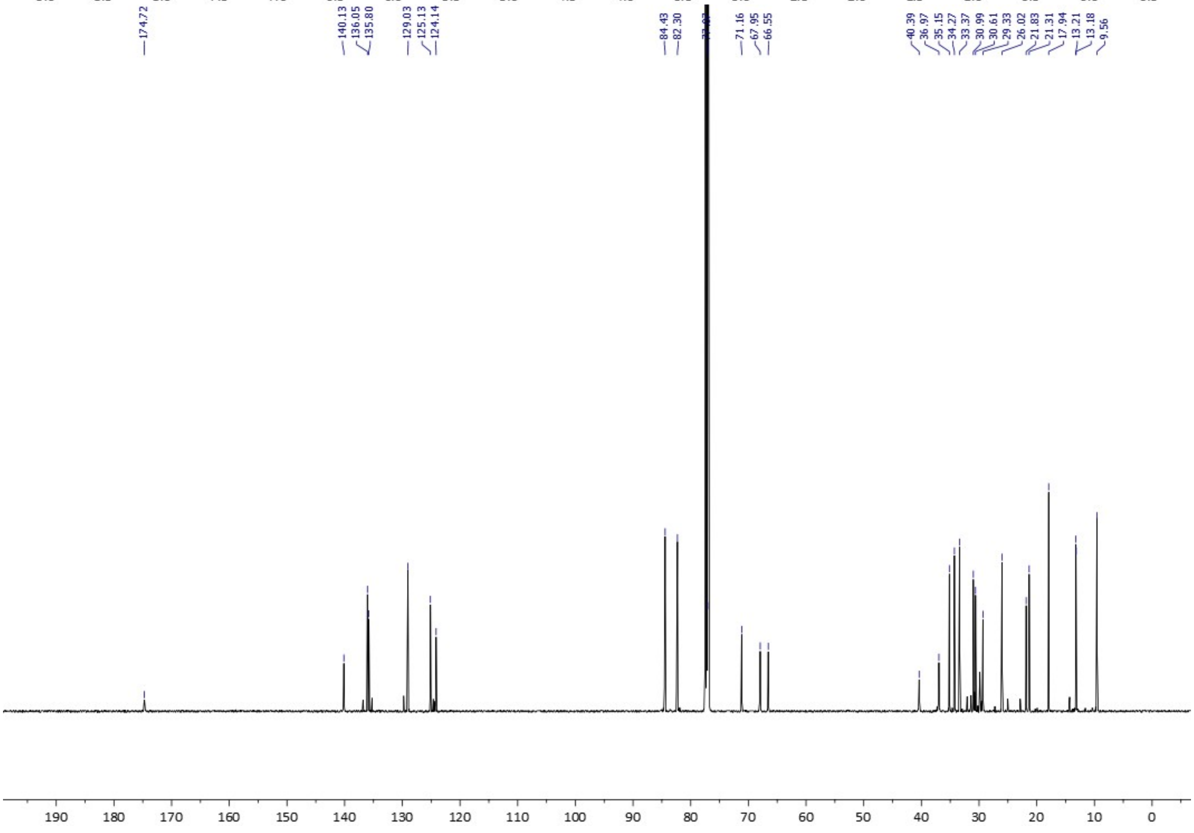

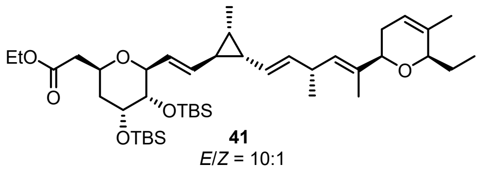
EtO
O
O
OTBS
OTBS
O

41
E/*Z* = 10:1

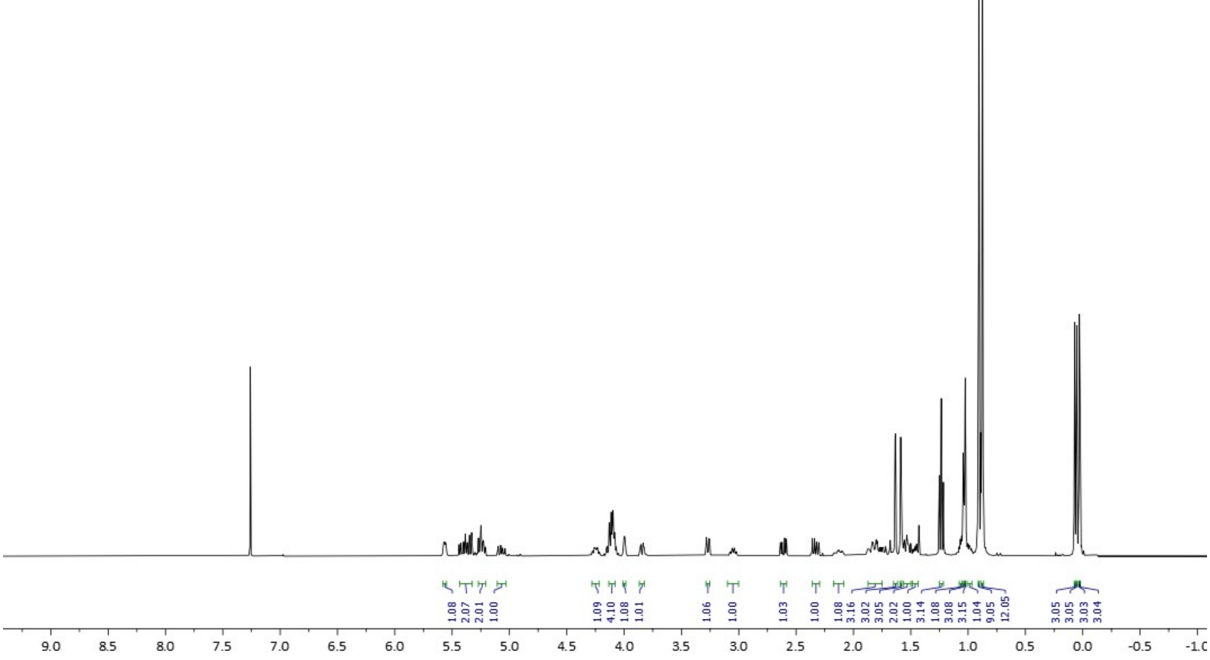
1.08
2.07
2.01
1.00
1.09
4.10
1.08
1.01
1.06
1.00
1.03
1.00
1.08
3.16
3.02
3.05
2.02
1.00
3.14
1.08
3.08
3.15
1.04
9.05
12.05
3.05
3.05
3.03
3.04
9.0
8.5
8.0
7.5
7.0
6.5
6.0
5.5
5.0
4.5
4.0
3.5
3.0
2.5
2.0
1.5
1.0
0.5
0.0
-0.5
-1.0

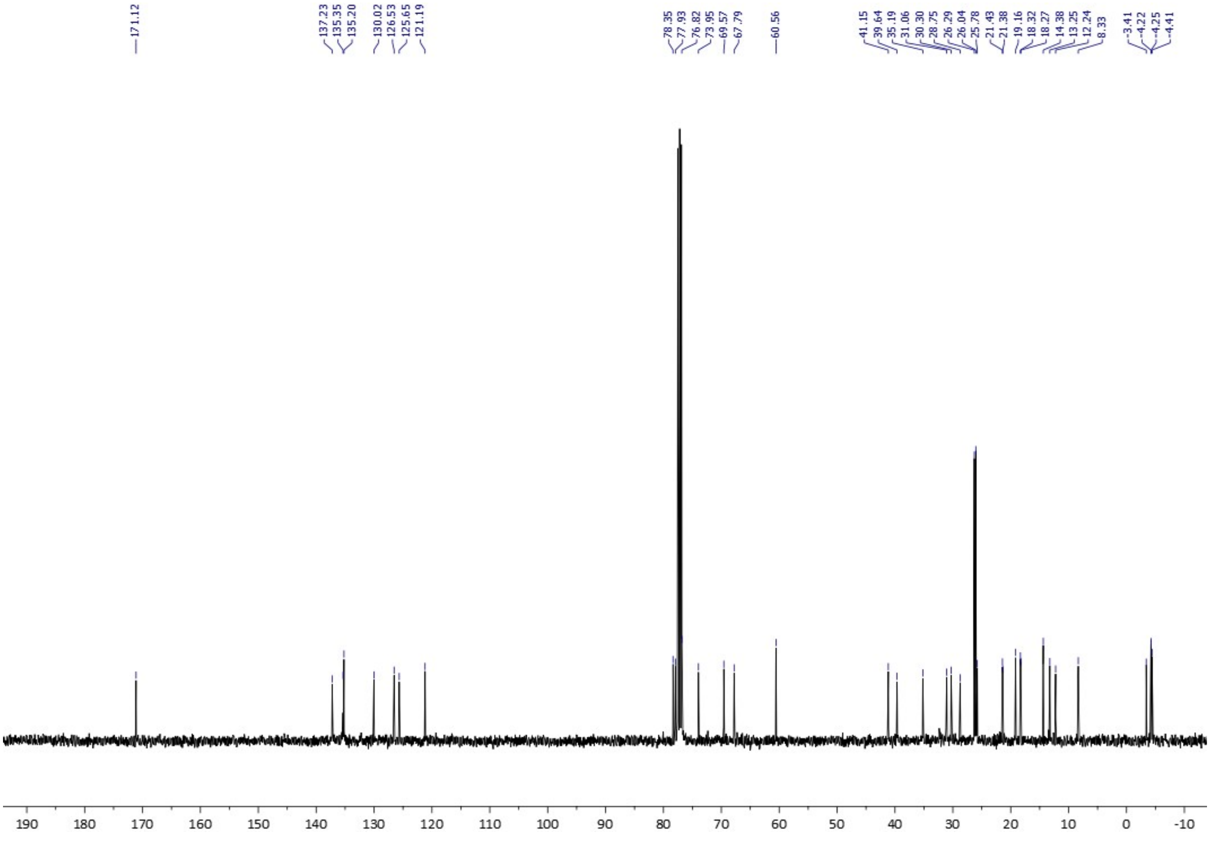
171.12
137.23
135.35
135.20
130.02
126.53
125.65
121.19
78.35
77.93
76.82
73.95
69.57
67.79
60.56
41.15
39.64
35.19
31.06
30.30
28.75
26.29
26.04
25.78
21.48
21.38
19.16
18.32
18.27
14.38
13.25
12.24
8.33
-3.41
-4.22
-4.25
-4.41
190
180
170
160
150
140
130
120
110
100
90
80
70
60
50
40
30
20
10
0
-10

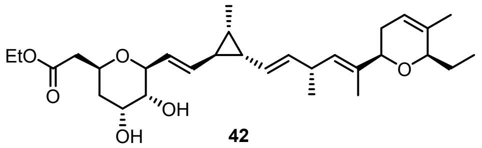
EtO
O
O
OH
OH
O

42
E/*Z* = 10:1

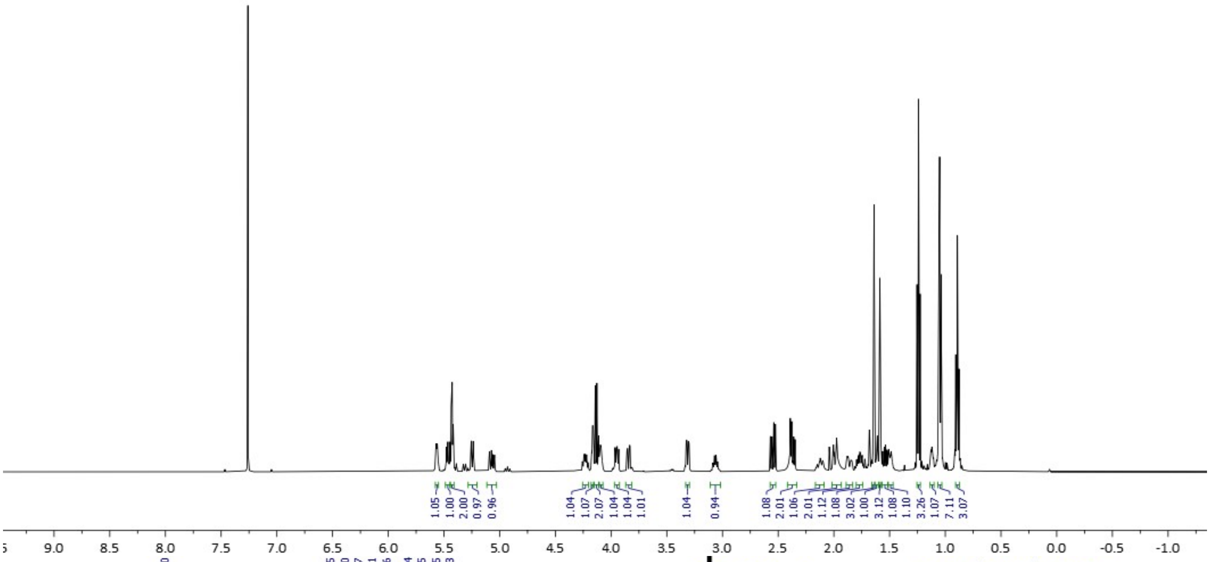
1.05
1.00
2.00
0.97
0.96
1.04
1.07
2.07
1.04
1.04
1.01
1.04
0.94
1.08
2.01
1.06
2.01
1.12
1.08
3.02
1.00
3.12
1.08
1.10
3.26
1.07
7.11
3.07
9.0
8.5
8.0
7.5
7.0
6.5
6.0
5.5
5.0
4.5
4.0
3.5
3.0
2.5
2.0
1.5
1.0
0.5
0.0
-0.5
-1.0

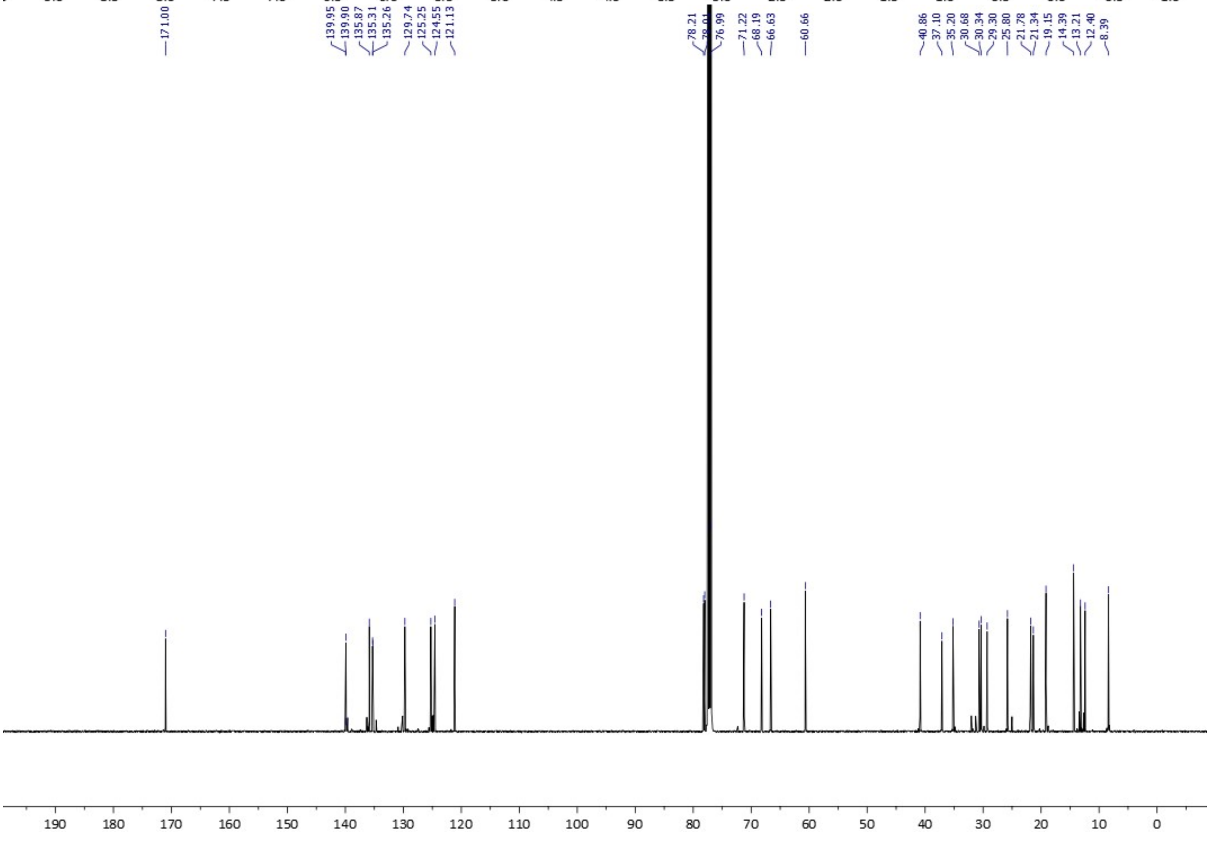
171.00
139.95
139.90
135.87
135.31
135.26
129.74
125.25
124.55
121.13
78.21
76.99
71.22
68.19
66.63
60.66
40.86
37.10
35.20
30.68
30.34
29.30
25.80
21.78
21.34
19.15
14.39
13.21
12.40
8.39
190
180
170
160
150
140
130
120
110
100
90
80
70
60
50
40
30
20
10
0

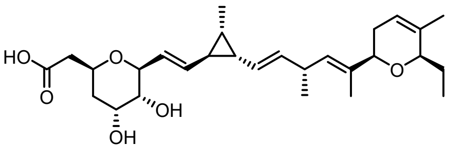

ambruticin F (2)
E/*Z* = 10:1
NMR spectra in $CDCl_3$

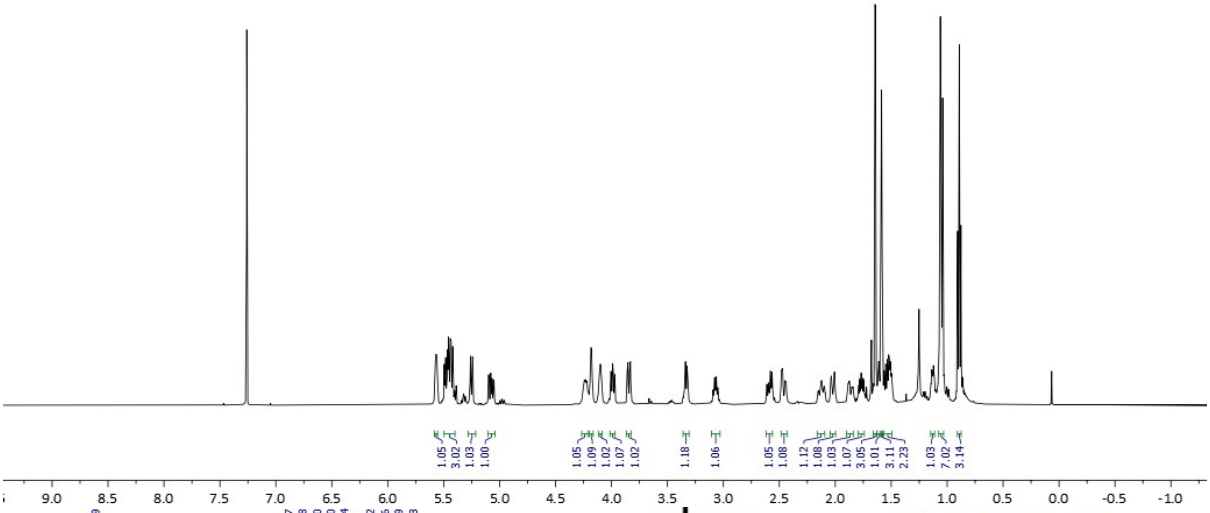

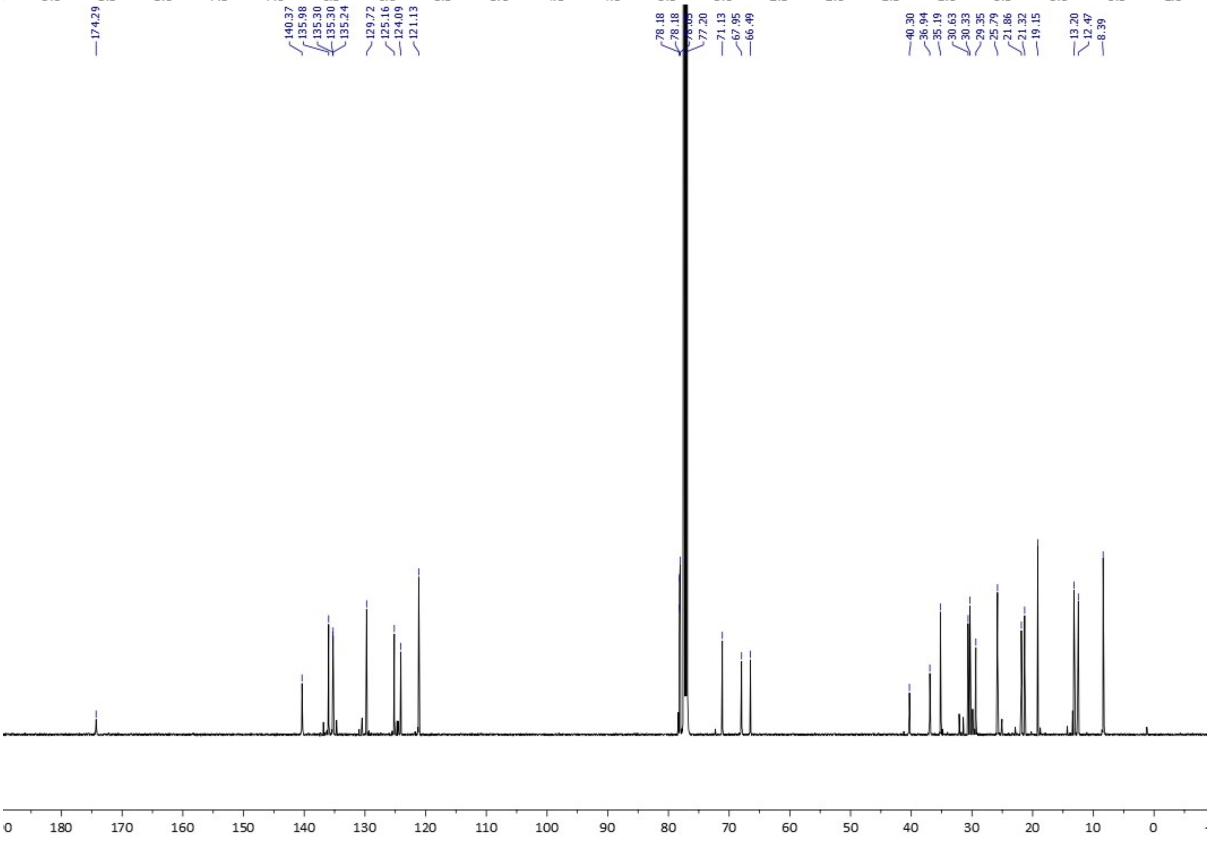

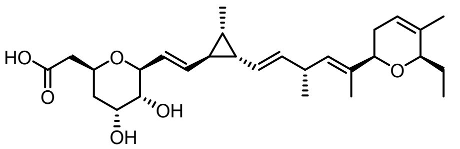

ambruticin F (2)

E/*Z* = 10:1

NMR spectra in methanol-D_4

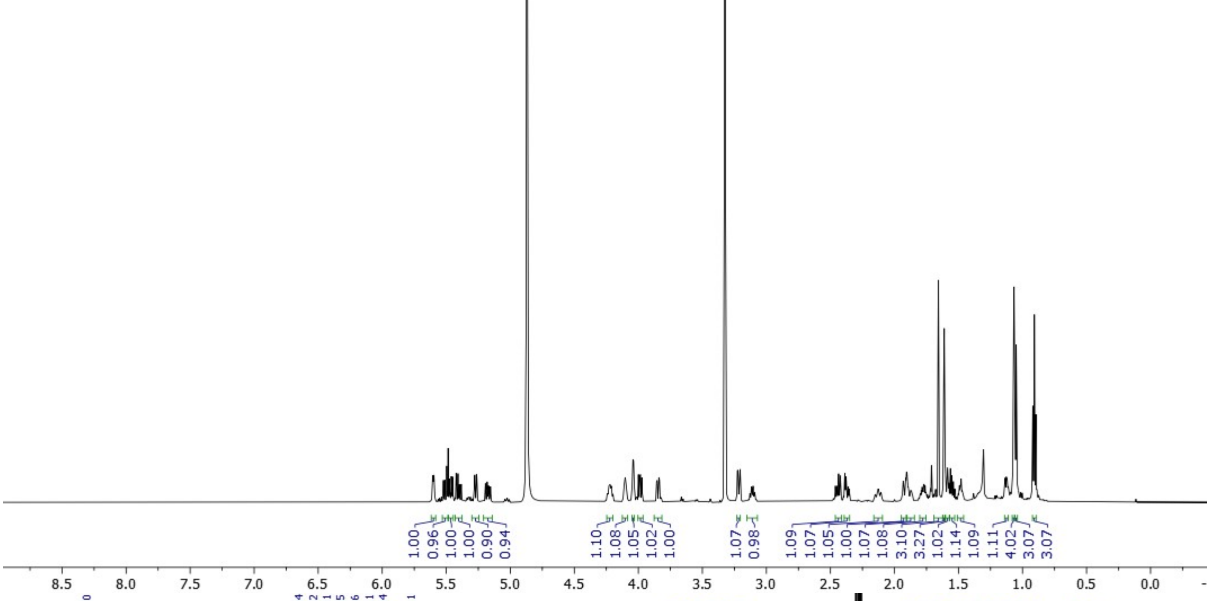

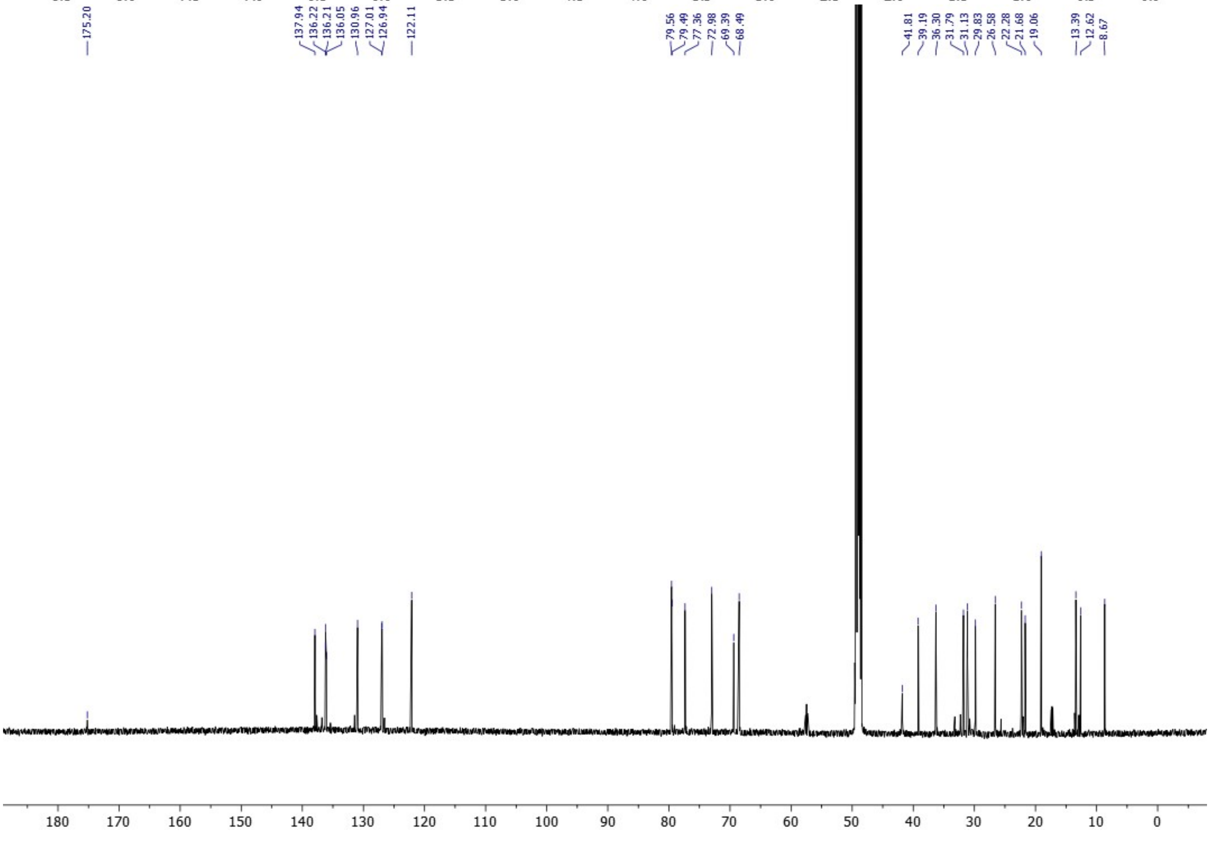

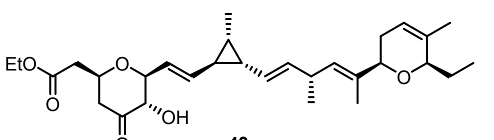
EtO
O
O
OH
O
O

43
E/*Z* = 10:1

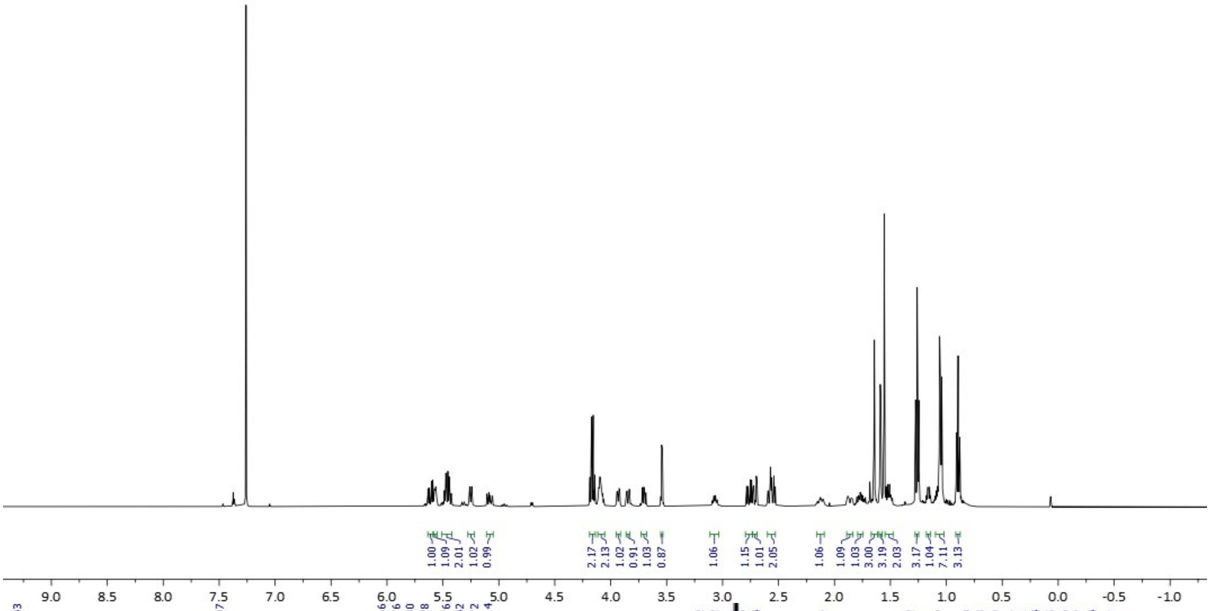
1.00
1.09
2.01
1.02
0.99
2.17
2.13
1.02
0.91
1.03
0.87
1.06
1.15
1.01
2.05
1.06
1.09
1.03
3.00
3.19
2.03
3.17
1.04
7.11
3.13
9.0 8.5 8.0 7.5 7.0 6.5 6.0 5.5 5.0 4.5 4.0 3.5 3.0 2.5 2.0 1.5 1.0 0.5 0.0 -0.5 -1.0

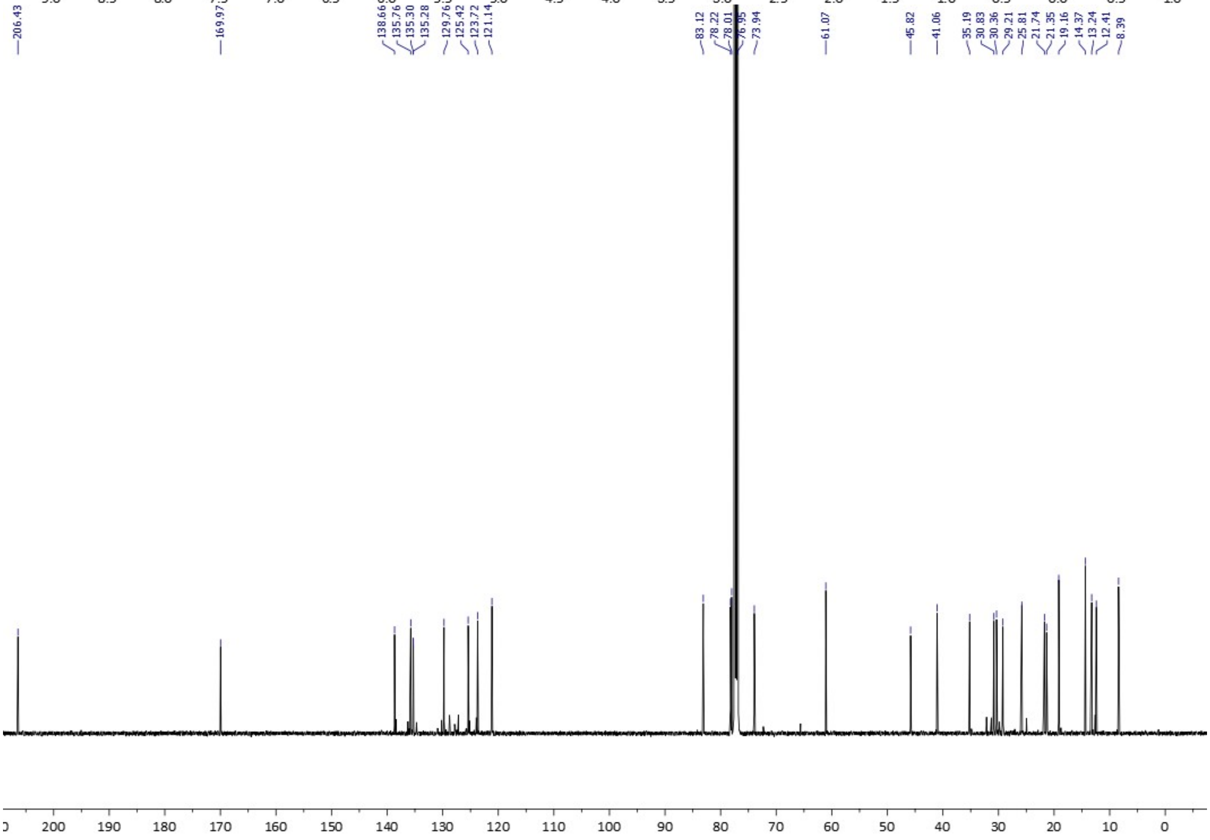
206.43
169.97
138.66
135.76
135.30
135.28
129.76
125.42
123.72
121.14
83.12
78.22
78.01
76.95
73.94
61.07
45.82
41.06
35.19
30.83
30.36
29.21
25.81
21.74
21.35
19.16
14.37
13.24
12.41
8.39
200 190 180 170 160 150 140 130 120 110 100 90 80 70 60 50 40 30 20 10 0

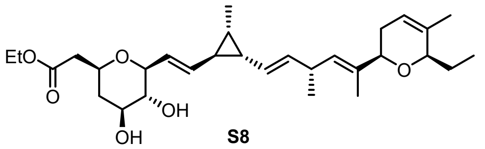

S8
E/*Z* = 10:1

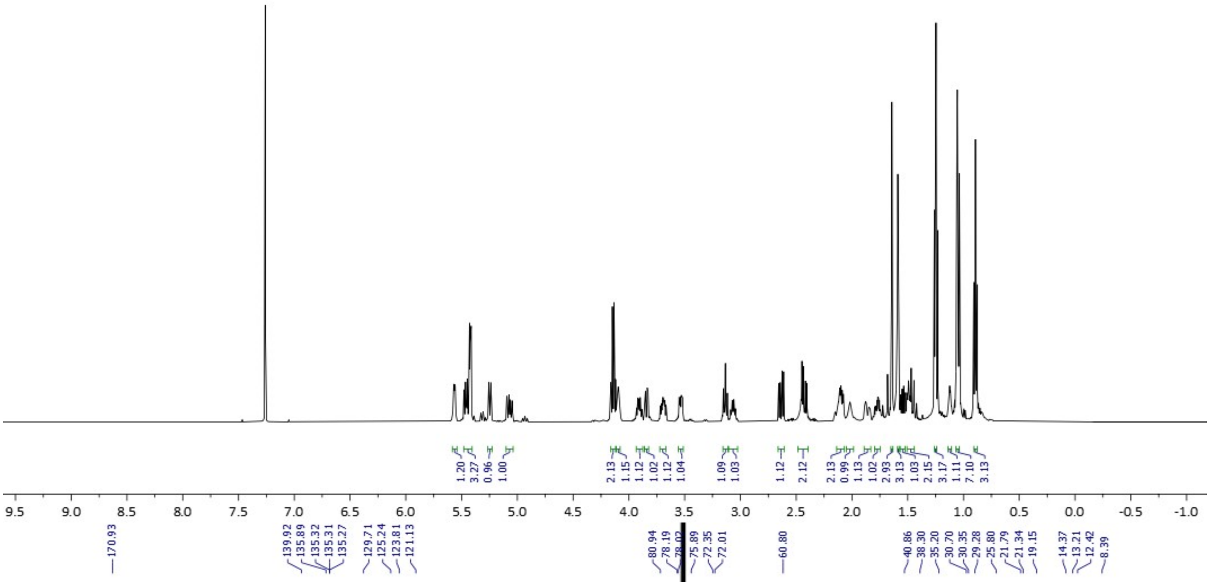

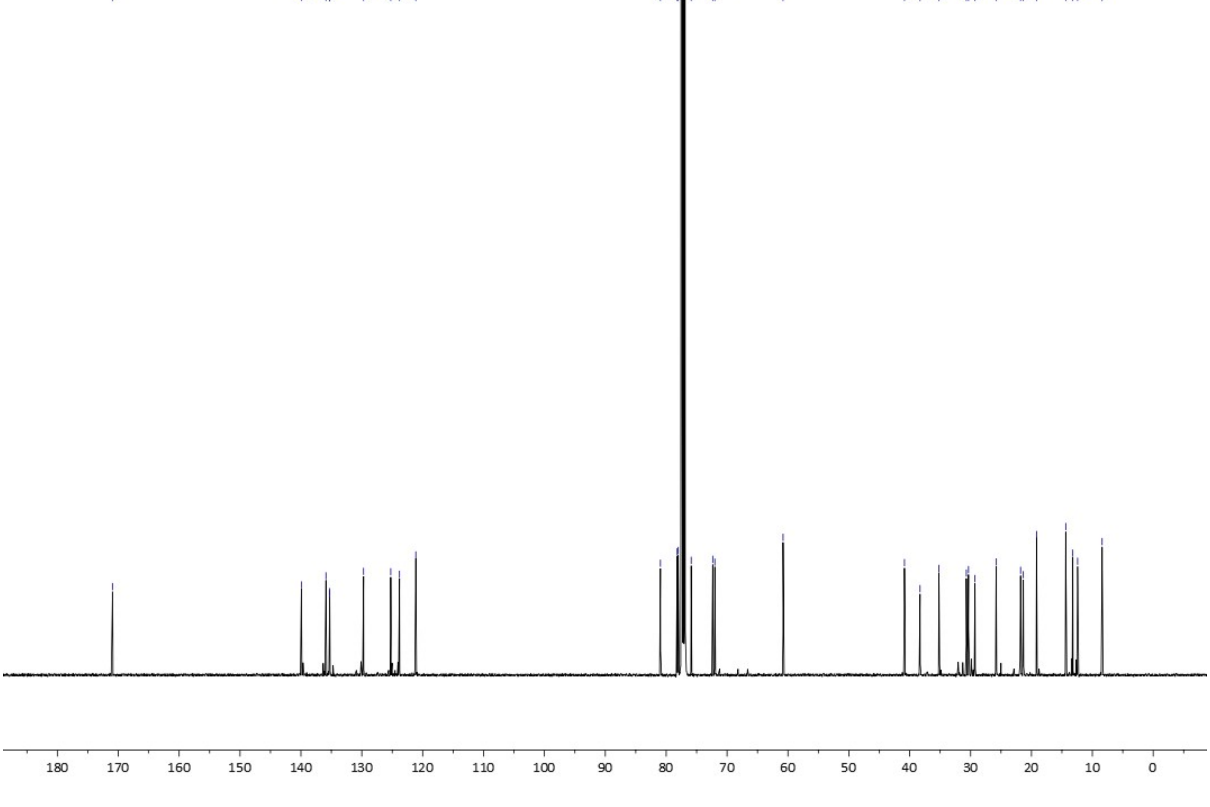

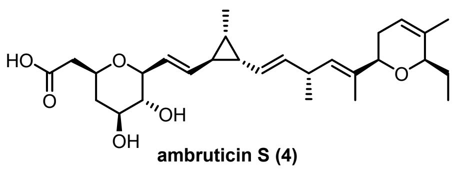

ambruticin S (4)
E/*Z* = 10:1
NMR spectra in $CDCl_3$

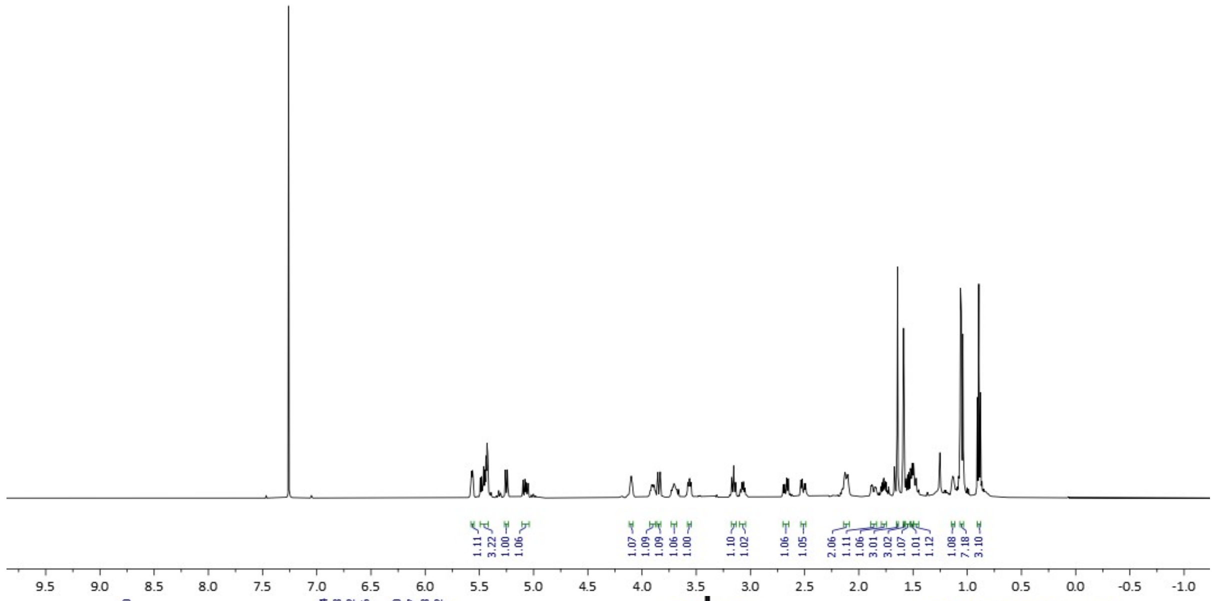

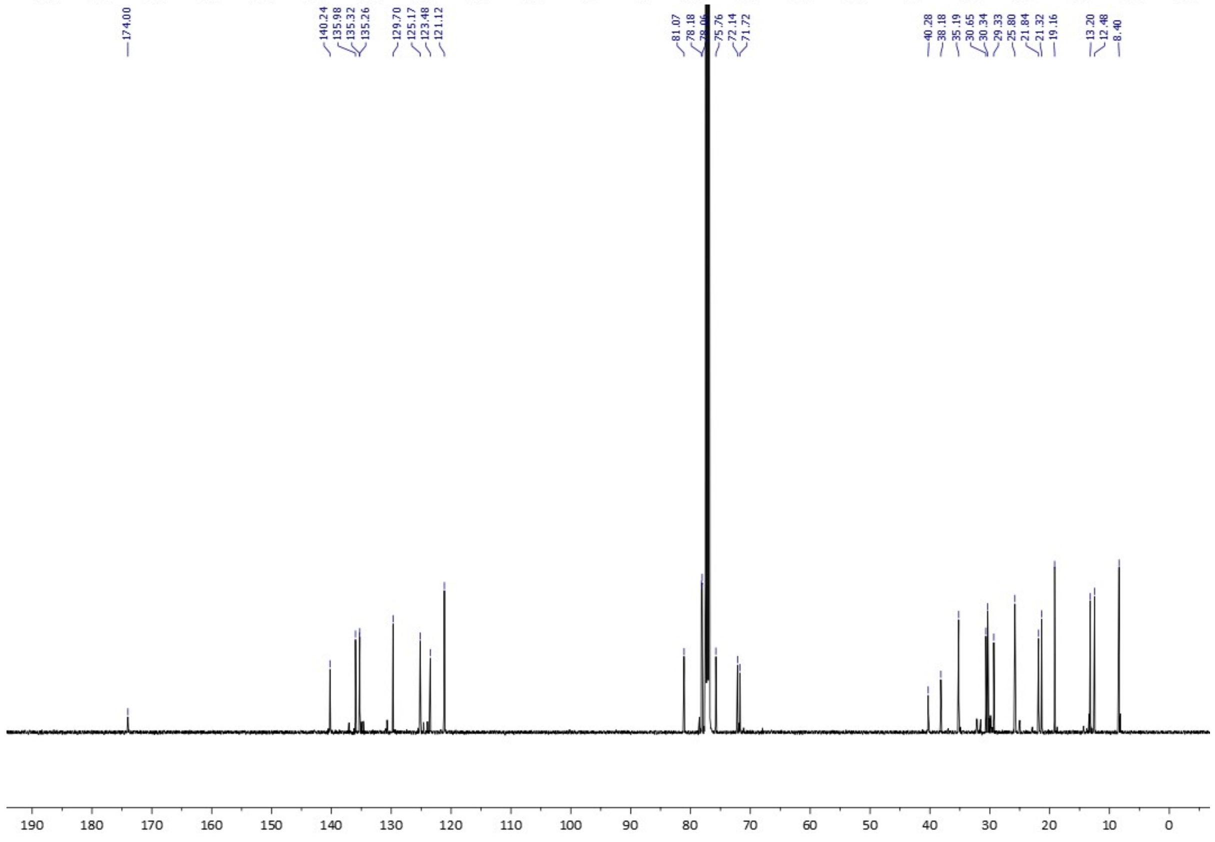

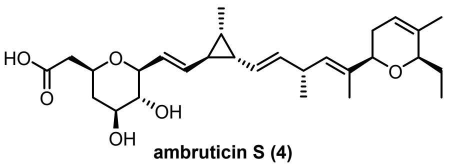

ambruticin S (4)
E/*Z* = 10:1
NMR spectra in methanol-D_4

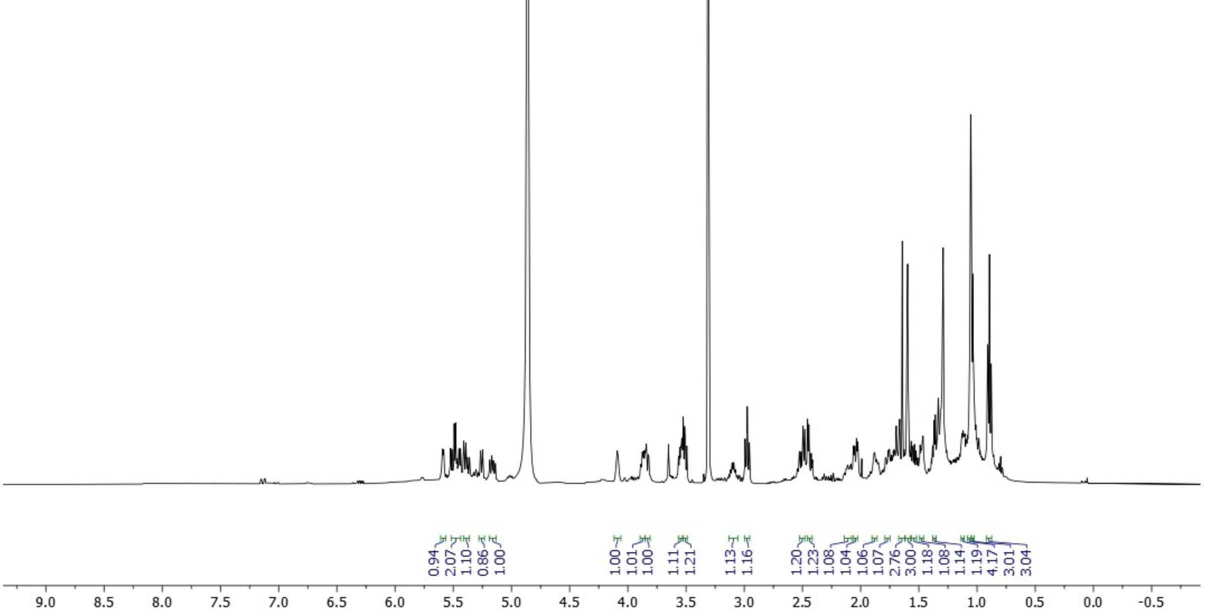

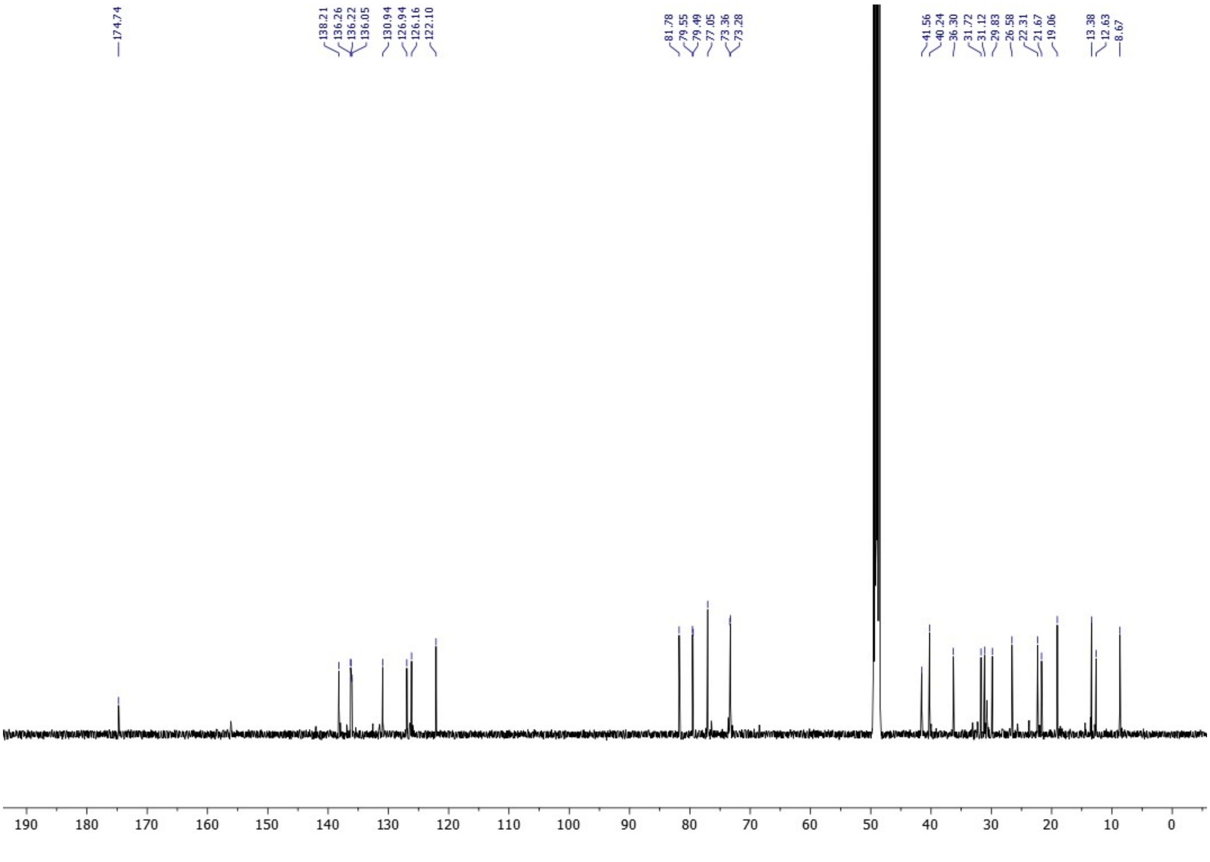

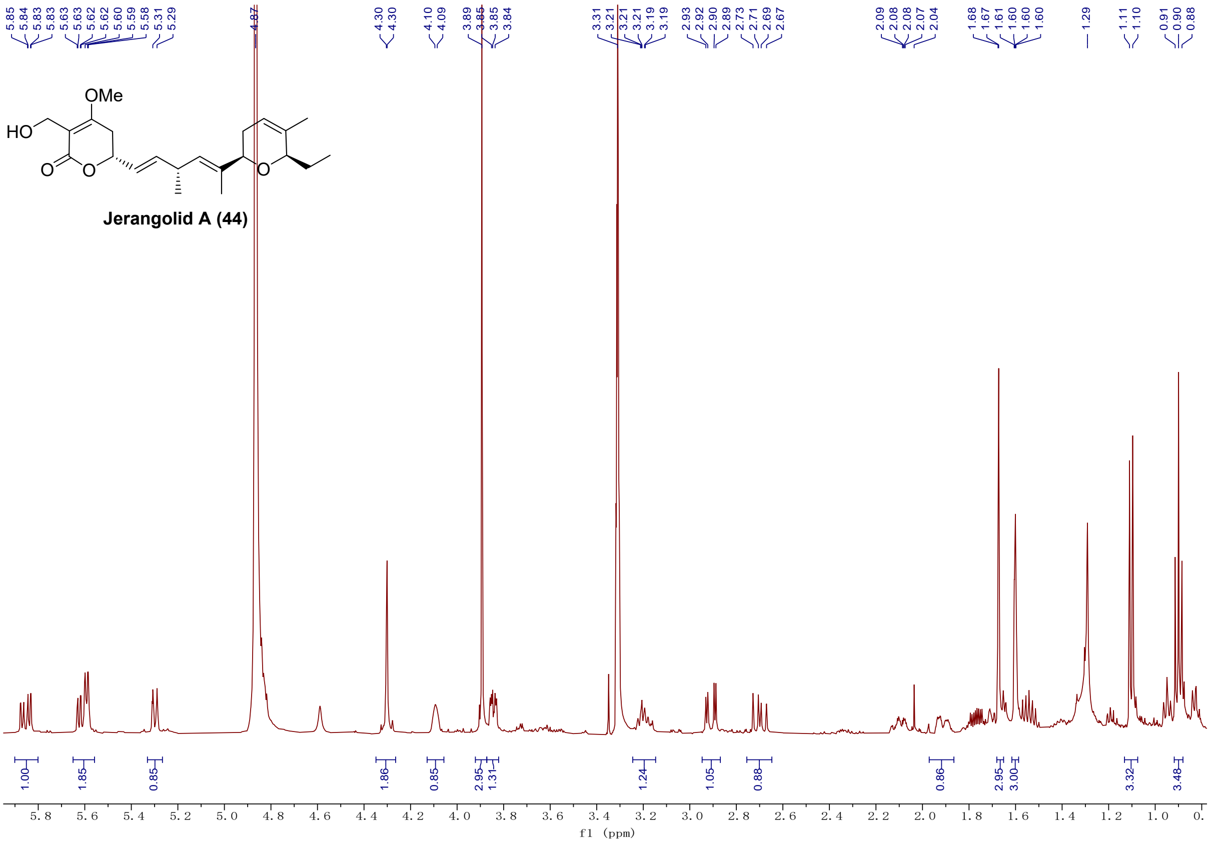
Jerangolid A (44)
f1 (ppm)

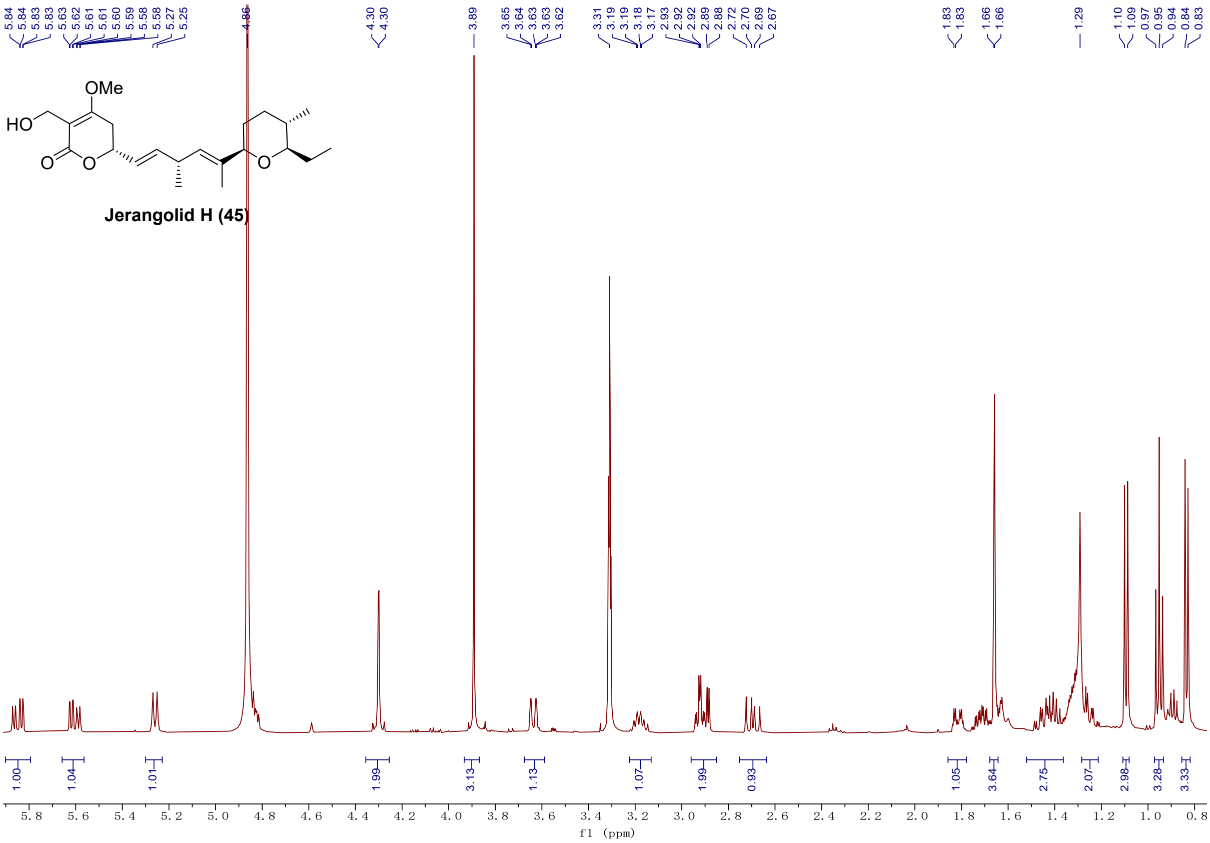
Jerangolid H (45)
f1 (ppm)
